# Redox-neutral photocatalytic cleavage and *gem*-difluoroalkenylation of lignin linkages

**DOI:** 10.1126/sciadv.ady2227

**Published:** 2025-08-15

**Authors:** Xia Hu, Loélie Ribadeau-Dumas, Cristina Nevado

**Affiliations:** Department of Chemistry, University of Zurich, Winterthurerstrasse 190, CH 8057, Zurich, Switzerland.

## Abstract

The conversion of lignin into high–value-added chemicals represents a promising strategy to enhance its valorization. However, developing catalytic systems and reagents that enable the selective activation and functionalization of specific linkages within its complex polymeric structure remains highly challenging. Here, we report a photocatalytic method for the efficient cleavage and *gem*-difluoroalkenylation of β-O-4 and β-1 lignin models, as well as native lignin biomass. By using α-trifluoromethyl alkenes as functionalization reagents, this protocol provides a wide array of valuable *gem*-difluoroalkenes through proton-coupled electron transfer (PCET), β-scission, radical addition, and β-F elimination processes. This redox-neutral method features operational simplicity, good functional group compatibility, and excellent selectivity. In addition, the derivatization of the resulting *gem*-difluorobishomoallylic alcohol highlights its synthetic potential.

## Teaser

A photocatalytic method for converting lignin into benzaldehydes and valuable *gem*-difluoroalkenes was developed.

## INTRODUCTION

**Fig. 1.**
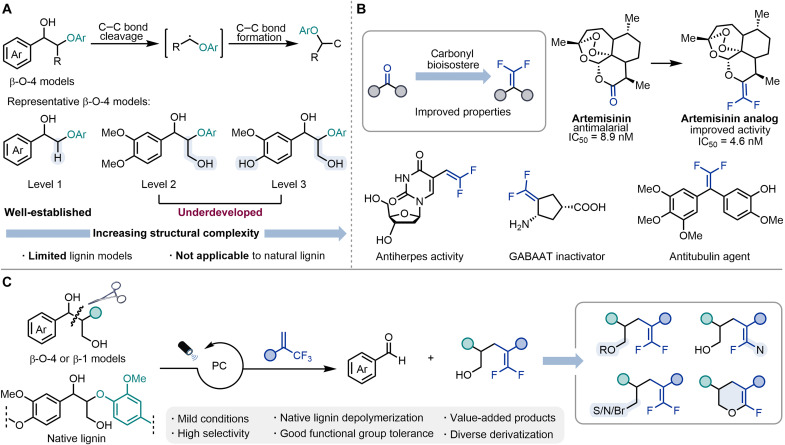
Development of photocatalytic cleavage and functionalization of lignin for the synthesis of *gem*-difluoroalkenes. (**A**) Photocatalytic depolymerization of lignin models and C─C bond formation of lignin fragments. (**B**) Representative biologically active *gem*-difluoroalkenes. IC_50_, median inhibitory concentration. (**C**) This work: Selective cleavage and functionalization of lignin models for the synthesis of *gem*-difluoroalkenes. PC, photocatalyst.

As the second largest biomass resource and the most abundant aromatic polymer in nature, lignin holds excellent potential as a renewable alternative to fossil fuels and a sustainable source for producing chemicals (*1*–*3*). Consequently, the conversion of lignin into low–molecular weight aromatic compounds is of great interest to both academia and industry alike (*4*–*11*). However, lignin is still far from being widely used due to its complex structure, which is composed of three distinct phenylpropanol subunits cross-linked through strong C─C/C─O bonds (*12*). Among them, the β-O-4 linkage has been regarded as a key target due to its prevalence. Various depolymerization strategies have been explored, which can be broadly classified as oxidative (*13*–*19*), reductive (*20*–*24*), and redox neutral in nature (*25*–*41*). Most of these transformations produce aromatic commodity chemicals such as benzaldehydes, benzoic acids, ketones, and phenols. To fully capitalize on lignin valorization, developing efficient methods to generate a wider variety of value-added products is essential. Photocatalytic approaches hold promise for lignin functionalization by triggering the generation and subsequent conversion of radical intermediates (*42*–*44*). However, the number of capturing reagents remains limited because of the challenges associated with instability of lignin fragment radicals. Up to date, only azodicarboxylates (*38*) and a handful of electron-deficient alkenes (*39*–*41*) have been adopted, with the latter being mainly applicable to simplified lignin models that contain only one hydroxyl group (Fig. 1A).

*gem*-Difluoroalkenes are an important class of organofluorine compounds with extensive applications in pharmaceuticals. As the ideal carbonyl bioisosteres, these compounds typically provide unique metabolic stability, biological activity, and target specificity (Fig. 1B) (*45*–*47*). Recently, considerable progress has been made in the catalytic defluorinative cross-coupling of α-trifluoromethyl alkenes with different precursors (*48*–*67*). However, the direct utilization of renewable carbon sources to access structurally diverse *gem*-difluoroalkenes is still underdeveloped. In this context, harnessing lignin for the synthesis of functional group–rich *gem*-difluoroalkenes not only advances biomass conversion but also opens opportunities in drug discovery due to the presence of hydroxyl groups as useful handles for further transformations.

Here, we present an efficient photocatalytic method for the selective cleavage and *gem*-difluoroalkenylation of β-O-4/β-1 lignin models and native lignin β-O-4 linkage by using α-trifluoromethyl alkenes as trapping reagents. This redox neutral reaction delivers the corresponding benzaldehydes and *gem*-difluoroalkenes in high yields at room temperature and ambient pressure. The resulting *gem*-difluoro-bishomoallylic alcohols have been successfully used to prepare a broad variety of organofluorine compounds through the functionalization of hydroxyl and/or *gem*-difluoroalkenyl groups (Fig. 1C).

## RESULTS

### Optimization of the reaction conditions

**Fig. 2.**
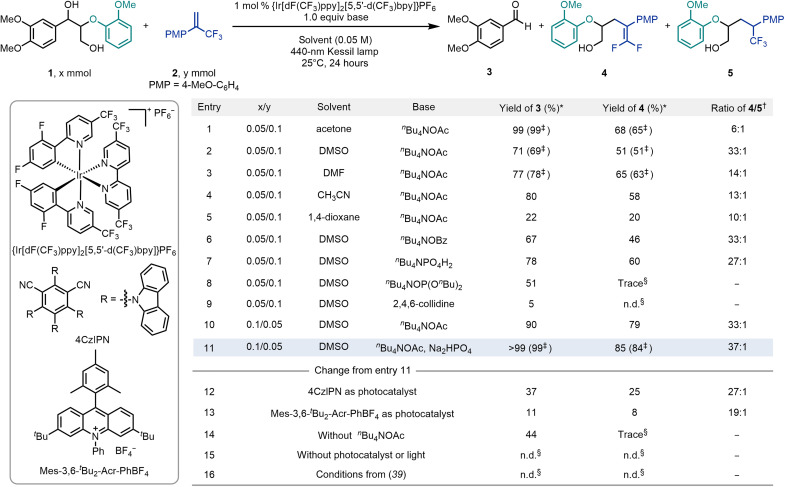
Optimization of reaction conditions. *Reaction conditions: 1-(3,4-dimethoxyphenyl)-2-(2-methoxyphenoxy)propane-1,3-diol (**1**, x mmol), 1-methoxy-4-(3,3,3-trifluoroprop-1-en-2-yl)benzene (y mmol), {Ir[dF(CF_3_)ppy]_2_[5,5′-d(CF_3_)bpy]}PF_6_ (0.0005 mmol, 1 mol %), base (0.05 mmol, 1 equiv), solvent = 1.0 ml, 40-W 440-nm Kessil lamp, N_2_, 25°C, 24 hours. Yield was determined by ^1^H nuclear magnetic resonance (NMR) by using 1,3,5-trimethoxybenzene as the internal standard. †Ratio was determined by ^19^F NMR. ‡Isolated yield. §Determined by gas chromatography–mass spectrometry. n.d., not detected; PMP, *para*-methoxyphenyl.

Our study commenced with β-O-4 lignin model **1** and 1-methoxy-4-(3,3,3-trifluoroprop-1-en-2-yl)benzene **2** as benchmark substrates (Fig. 2). After an extensive screening, we found that the reaction of 2 equivalents of alkene with 1 mol % {Ir[dF(CF_3_)ppy]_2_[5,5′-d(CF_3_)bpy]}PF_6_ under blue light irradiation (440-nm Kessil lamp) for 24 hours in the presence of 1 equivalent of tetrabutylammonium acetate (*^n^*Bu_4_NOAc) in acetone as solvent furnished the desired 3,4-dimethoxybenzaldehyde **3** and *gem*-difluoroalkenylation product **4** in 99 and 65% isolated yield, respectively (Fig. 2, entry 1). As expected, a small amount of the Giese-type hydroalkylation product (**5**) was also detected in the reaction mixture in a 6:1 ratio. The solvent has an essential impact on the selectivity of the reaction. The use of dimethyl sulfoxide (DMSO) notably improved the selectivity to 33:1 although with a slight decrease in yield (Fig. 2, entry 2). Other solvents such as *N*,*N*′-dimethylformamide (DMF) or acetonitrile (CH_3_CN) also demonstrated good reactivity, albeit with lower selectivity compared to DMSO (Fig. 2, entries 3 and 4). The use of 1,4-dioxane resulted in a substantial decrease in the reaction efficiency (Fig. 2, entry 5). The examination of bases revealed that tetrabutylammonium benzoate (*^n^*Bu_4_NOBz) and tetrabutylammonium dihydrogen phosphate (*^n^*Bu_4_NPO_4_H_2_) were also competent, yielding **4** with slightly lower yield or ratio with respect to by-product **5** (Fig. 2, entries 6 and 7). However, tetrabutylammonium di-*tert*-butylphosphate [*^n^*Bu_4_NOP(O*^n^*Bu)_2_] and 2,4,6-collidine failed to produce the desired product (Fig. 2, entries 8 and 9). When 2 equivalents of lignin model **1** were used, compounds **3** and **4** were obtained in 90 and 79% yield, respectively (Fig. 2, entry 10). The addition of 1 equivalent of Na_2_HPO_4_ further improved the reaction outcome, providing the desired product **3** in 99% yield, whereas difluorinated alkene **4** could be isolated in 84% yield with excellent selectivity with respect to trifluoromethyl adduct **5** (37:1 ratio) (Fig. 2, entry 11). Organic photosensitizers such as 4CzlPN and Mes-3,6-*^t^*Bu_2_-Acr-PhBF_4_ showed poor performances for this transformation (Fig. 2, entries 12 and 13). The essential role of the base was demonstrated by the lack of conversion to **4** when *^n^*Bu_4_NOAc was removed from the reaction mixture (Fig. 2, entry 14). Control experiments confirmed that no reaction occurs in the absence of photocatalyst or light (Fig. 2, entry 15). Notably, β-O-4 lignin model **1** did not react under previously reported methods (Fig. 2, entry 16; see table S7 in the Supplementary Materials) (*39*, *40*).

### Substrate scope

**Fig. 3.**
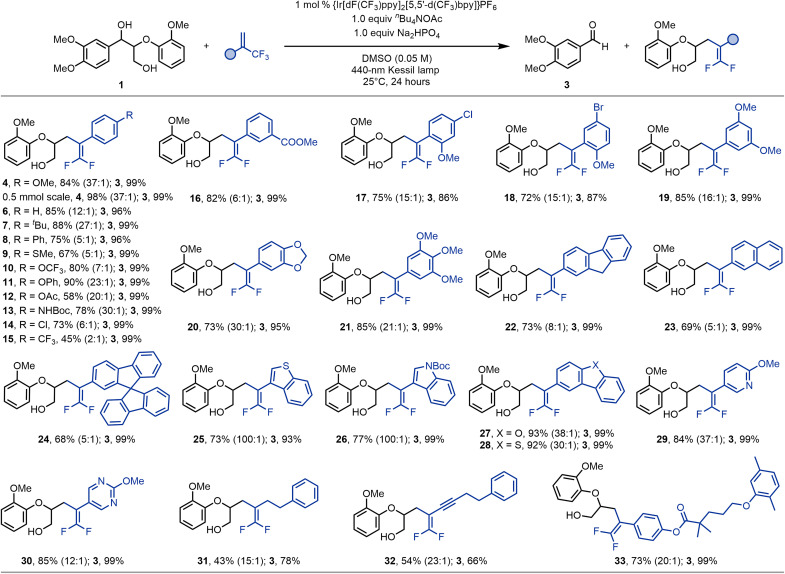
Scope of α-trifluoromethyl alkenes. Reaction conditions: 1-(3,4-Dimethoxyphenyl)-2-(2-methoxyphenoxy)propane-1,3-diol (**1**, 0.1 mmol, 2 equiv), α-trifluoromethyl alkene (0.05 mmol, 1 equiv), {Ir[dF(CF_3_)ppy]_2_[5,5′-d(CF_3_)bpy]}PF_6_ (0.0005 mmol, 1 mol %), *^n^*Bu_4_NOAc (0.05 mmol, 1 equiv), Na_2_HPO_4_ (0.05 mmol,1.0 equiv), DMSO = 1.0 ml, 40-W 440-nm Kessil lamp, N_2_, 25°C, 24 hours. Isolated yield. The ratio of *gem*-difluoroalkenylation product and Giese-type hydroalkylation by-product was determined by ^19^F NMR and is shown in parentheses.

With the optimized conditions in hand, we sought to investigate the generality of this transformation. A broad array of α-trifluoromethyl alkenes was evaluated first (Fig. 3). The yield of **4** improved to 98% when the reaction scale was increased to 0.5 mmol. The cleavage/defluorinative cross-coupling of lignin model **1** with α-trifluoromethylstyrene led to the formation of product **6** in 85% yield. A wide range of α-trifluoromethyl styrenes bearing electron-donating (-*^t^*Bu, -Ph, -SMe, -OCF_3_, -OPh, -OAc, and -NHBoc) as well as electron-withdrawing (-Cl and -CF_3_) groups in the *para* position were successfully converted to the corresponding products in good to excellent yields and selectivities over the Giese addition products (**7** to **15**). In general, electron-rich alkenes exhibited better selectivity for defluorination products, which can be ascribed to the formation of resonance stabilized, longer-lived anion intermediates in the case of electron-deficient substrates. A methyl ester substituted alkene furnished product **16** in 82% yield, demonstrating the compatibility of the method with base-sensitive functional groups. Further, the presence of Csp^2^─Cl and Csp^2^─Br bonds proved to be tolerated under the reaction conditions thus enabling subsequent functionalization of the products (**17** and **18**) via cross-coupling protocols. The reaction proceeds smoothly when substrates bearing multiple electron-donating groups were used (**19** to **21**). In addition to styrenic systems, polycyclic aryl-substituted α-CF_3_ alkenes such as 9H-fluorene (**22**), naphthalene (**23**), and 9,9′-spirobi[fluorene] (**24**) were also well-tolerated in this protocol. Furthermore, olefins containing benzothiophene (**25**), *N*-Boc protected indole (**26**), dibenzofuran (**27**), dibenzothiophene (**28**), pyridine (**29**), and pyrimidine (**30**) heterocycles could be easily transformed under the standard conditions. Alkyl-substituted trifluoromethyl alkenes were found to be suitable substrates, as exemplified by the successful isolation of compound **31**. 2-Trifluoromethyl-1,3-enynes also turned out to be compatible, delivering the corresponding 1,1-difluoro-1,3-enyne in 54% yield without concomitant addition to the alkynyl moiety (**32**). It is worth noting that a structurally complex alkene derived from gemfibrozil also furnished the desired *gem*-difluoroalkenylation product in 73% yield (**33**).

**Fig. 4.**
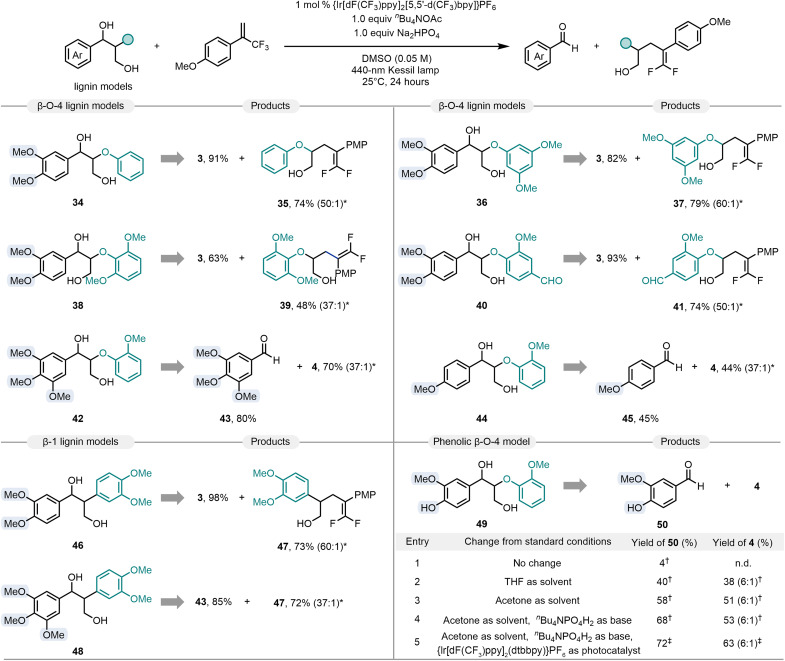
Scope of lignin models. *Reaction conditions: Lignin model (0.1 mmol, 2 equiv), 1-methoxy-4-(3,3,3-trifluoroprop-1-en-2-yl)benzene (0.05 mmol, 1 equiv), {Ir[dF(CF_3_)ppy]_2_[5,5′-d(CF_3_)bpy]}PF_6_ (0.0005 mmol, 1 mol %), *^n^*Bu_4_NOAc (0.05 mmol, 1 equiv), Na_2_HPO_4_ (0.05 mmol,1.0 equiv), DMSO = 1.0 ml, 40-W 440-nm Kessil lamp, N_2_, 25°C, 24 hours. Isolated yield. †Yield was determined by ^1^H NMR by using 1,3,5-trimethoxybenzene as the internal standard. ‡Isolated yield. The ratio of *gem*-difluoroalkenylation product and Giese-type hydroalkylation by-product was determined by ^19^F NMR and is shown in parentheses. THF, tetrahydrofuran.

The scope with respect to lignin surrogates was explored next (Fig. 4). β-O-4 lignin models containing both α- and γ-hydroxyl groups could readily participate in the consecutive fragmentation/C─C bond construction process. Specifically, model substrate **34** successfully delivered veratraldehyde (**3**) and 5,5-difluoro-4-(4-methoxyphenyl)-2-phenoxypent-4-en-1-ol (**35**) in 91 and 74% yield, respectively. Our protocol could be further applied to coniferyl-derived diols **36**, **38**, and **40**, sinapyl derivative **42**, and coumaryl lignin system **44**, thus affording the corresponding adducts (**37**, **39**, **41**, and **4**) in good yields. The moderate yield of **38** might be because of the increased steric hindrance caused by the bis-*ortho* methoxy substituents. Subsequent investigations revealed that selective C─C bond scission and functionalization could be also accomplished when β-1 lignin models **46** and **48** were subjected to the photocatalytic reaction system. However, phenolic β-O-4 lignin model **49** was not amenable to the standard reaction conditions, likely due to the weaker bond dissociation energy of phenolic hydroxyl groups compared to aliphatic hydroxyl bonds (*68*). A small rescreening of reaction conditions (see table S8 in the Supplementary Materials) revealed acetone as solvent, *^n^*Bu_4_NPO_4_H_2_ as base, and {Ir[dF(CF_3_)ppy]_2_(dtbbpy)}PF_6_ as photocatalyst for successful transformation of phenolic diol substrates. The desired products vanillin (**50**) and *gem*-difluoroalkene **4** were formed in 72 and 63% isolated yield, respectively.

**Fig. 5.**
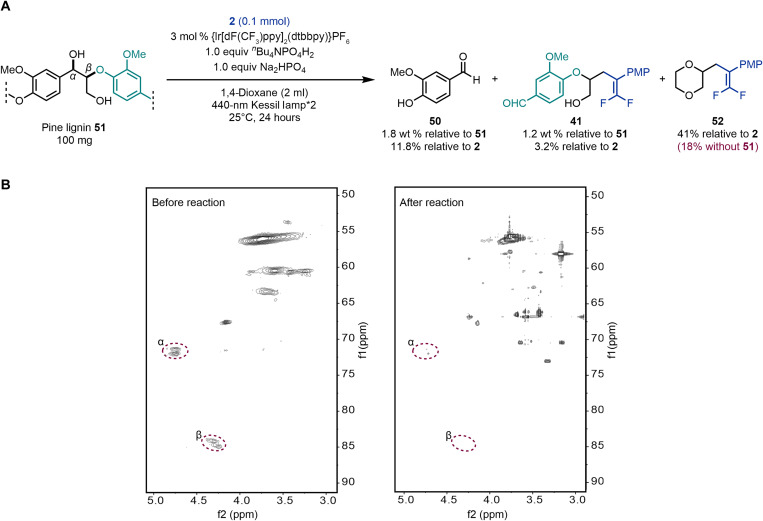
Native lignin experiments. (**A**) Photocatalytic depolymerization and *gem*-difluoroalkenylation of native pine lignin. (**B**) HSQC spectra of pine lignin before and after reaction. See figs. S1 and S7 in the Supplementary Materials for full HSQC spectra.

Encouraged by the successful outcomes in the depolymerization of lignin models, further studies were conducted to evaluate the applicability of this approach to native lignin derived from pine wood (Fig. 5). The lignin was prepared by using a previously reported dioxane/HCl extraction protocol (*14*). Building on the reaction conditions established for the phenolic β-O-4 lignin model, 1,4-dioxane was used as the solvent to ensure sufficient dissolution of pine lignin. We speculate that the reason for the success with 1,4-dioxane is the reversible proton-coupled electron transfer (PCET) activation of the phenolic hydroxyl bond in this solvent, while the reaction fails with DMSO due to its inability to serve as a good hydrogen atom donor for the phenoxy radical. Irradiation of 100 mg of pine lignin **51** and 0.1 mmol of alkene **2** for 24 hours resulted in the depolymerization of β-O-4 linkages. Gas chromatography–mass spectrometry analysis confirmed the formation of lignin degradation products vanillin **50** and *gem*-difluoroalkene **41**, with isolated yields of 1.8 and 1.2 wt %, respectively. In addition, the reaction between 1,4-dioxane and alkene **2** generated product **52**, which was isolated in 41% yield (Fig. 5A). Control experiment revealed that **52** could be obtained in 18% yield in the absence of lignin, demonstrating that 1,4-dioxane can be directly activated by the photocatalyst although the presence of lignin seems to promote this process, likely due to a hydrogen atom transfer process mediated by lignin-derived oxygen radicals. Furthermore, the depolymerization process was also investigated by two-dimensional heteronuclear single quantum coherence (HSQC) spectroscopy (Fig. 5B). The HSQC analysis revealed that the characteristic signals corresponding to C_α_-H and C_β_-H were remarkably diminished after the reaction.

### Synthetic applications

**Fig. 6.**
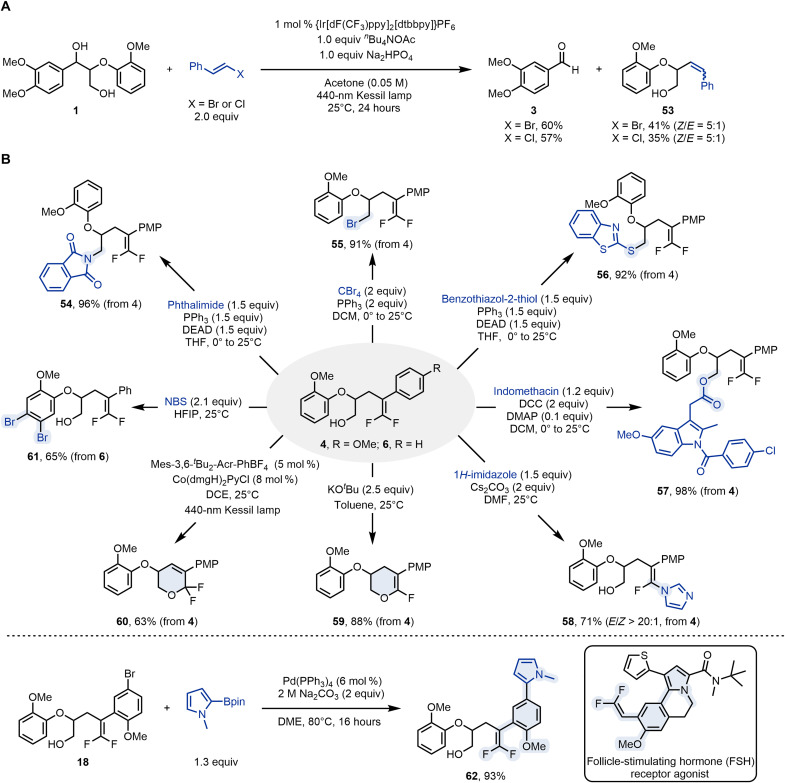
Synthetic applications. (**A**) Expansion of scope. (**B**) Derivatization of products. DCM, dichloromethane; DCC, dicyclohexylcarbodiimide; PPh_3_, triphenylphosphine; NBS, *N*-bromosuccinimide; HFIP, hexafluoro-2-propanol; DCE, 1,2-dichloroethane; DMAP, 4-dimethylaminopyridine.

Our protocol can be extended to β-halogenated alkenes. The reactions of lignin model **1** with β-bromo- and β-chlorostyrenes were successfully performed under modified reaction conditions, using acetone as solvent and 2 equivalents of the alkene. The alkenylation product **53** was obtained with a *Z*/*E* selectivity of 5:1, in 41 and 35% isolated yields, respectively (Fig. 6A). The *gem*-difluoro-bishomoallylic alcohol adducts stemming from this transformation are versatile precursors for the assembly of valuable fluorine-containing compounds (Fig. 6B) (*69*, *70*). Nucleophilic substitutions of the hydroxyl moiety converted **4** into the corresponding *gem*-difluoro-bishomoallylic phthalimide (**54**), bromide (**55**), and thioether (**56**) in high yields. In addition, indomethacin derivative (**57**) was rapidly obtained through esterification, highlighting the potential of this methodology for the late-stage modification of bioactive molecules. Nucleophilic vinylic substitution (S_N_V) of **4** furnished tetra-substituted monofluoroalkene (**58**) with excellent *E* selectivity by using imidazole as the nucleophile. Notably, monofluorinated alkenes are considered as mimics of amides due to their similar charge distributions and dipole moments, which have garnered great attention in medicinal research (*71*, *72*). The addition-elimination of **4** was readily accomplished by treatment with *^t^*BuOK in toluene, delivering 6-fluoro-3,4-dihydro-**2H**-pyran (**59**) in 88% yield. Addition of a cobalt-oxime cocatalyst (*73*) resulted in an intramolecular oxidative cyclization to deliver 6,6-difluoro-3,6-dihydro-2H-pyran (**60**). We speculate that this transformation involves a *gem*-difluoroalkene radical cation intermediate. Furthermore, the regioselective dibromination of the aromatic ring in the lignin fragment was successfully achieved, affording product **61** and offering a versatile handle for further derivatization of these scaffolds. The Suzuki-Miyaura coupling of **18** and *N*-methylpyrrole-2-boronic acid pinacol ester provided the corresponding product **62** in 93% yield, which contains the core skeleton of a follicle-stimulating hormone receptor agonists used for treating infertility (*74*).

**Fig. 7.**
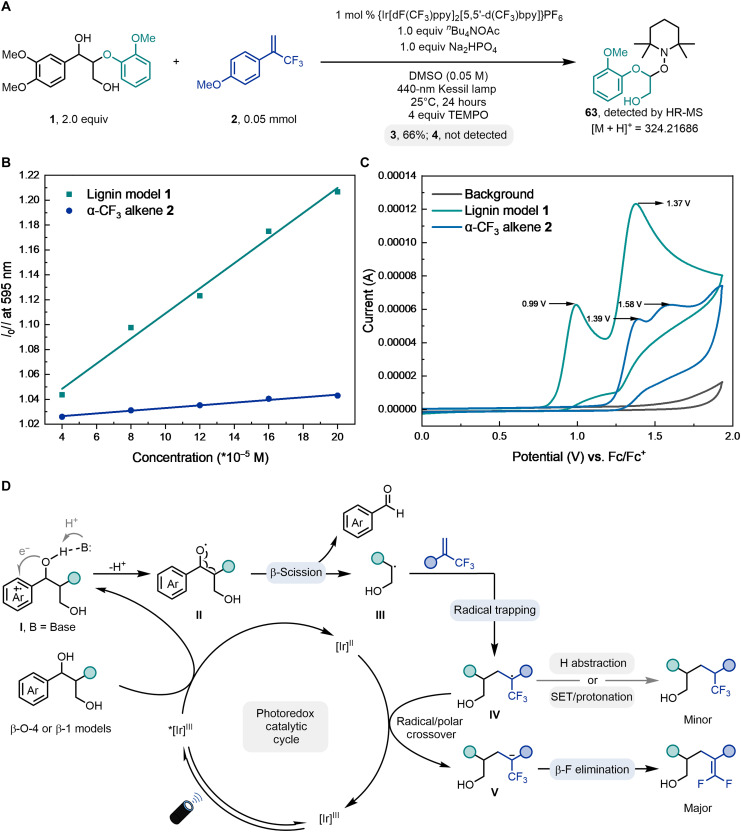
Mechanistic studies and proposed mechanism. (**A**) Radical trapping experiment. (**B**) Stern-Volmer studies. (**C**) Cyclic voltammograms studies. (**D**) Proposed mechanism. HR-MS, high-resolution mass spectrometry.

Several experiments were designed to probe the reaction mechanism of this transformation. No desired *gem*-difluoroalkenylation product **4** was detected in the presence of 2,2,6,6-tetramethyl-1-piperidinyloxy (TEMPO), and the TEMPO-containing adduct **63** could be detected by high-resolution mass spectrometry in the mixture, thus supporting the formation of the lignin-based alkyl radical via the β-scission of the C─C bond (Fig. 7A). Stern-Volmer fluorescence quenching experiments were conducted to identify potential reactants activated by the photocatalyst (Fig. 7B). A clear decrease in fluorescence intensity of the excited state of {Ir[dF(CF_3_)ppy]_2_[5,5′-d(CF_3_)bpy]}PF_6_ (Ir[III]*) was observed with increasing concentrations of lignin model **1**, exhibiting a linear Stern-Volmer behavior. In contrast, α-CF_3_ alkene **2** showed very weak quenching ability. Cyclic voltammetry studies are also consistent with these results (Fig. 7C), as the oxidative potential of α-CF_3_ alkene **2** (*E*_1/2_ = 1.30 V versus Fc/Fc^+^, oxidative peak observed at 1.39 V) is more positive than that of lignin model **1** (*E*_1/2_ = 0.91 V versus Fc/Fc^+^, oxidative peak observed at 0.99 V). These results indicate that lignin model **1** is more easily oxidized by the photocatalyst {Ir[dF(CF_3_)ppy]_2_[5,5′-d(CF_3_)bpy]}PF_6_ (*E_1/2_ = 1.30 V versus Fc/Fc^+^).

On the basis of this experimental evidence and precedents in the literature (*36*, *37*, *39*), a plausible mechanism for the lignin model cleavage and functionalization can be proposed (Fig. 7D). Upon irradiation, the excited state of the photocatalyst *[Ir]^III^ would be formed enabling the single-electron oxidation of lignin models to furnish transient radical cation **I**. An intramolecular PCET process would then take place to provide alkoxy radical intermediate **II**, in which the deprotonation of the hydroxyl group by the base occurs together with the single-electron reduction of the radical cation. Because of the substantial weakening of adjacent C─C bonds by alkoxy radicals, β-scission of **II** would deliver the alkyl radical species **III** and the corresponding aldehyde. Subsequent radical addition of **III** to α-trifluoromethyl alkene forms α-CF_3_ radical **IV**. Rather than undergoing a hydrogen abstraction process, **IV** is more likely to experience single-electron reduction by the reduced photocatalyst [Ir]^II^ to generate carbanion **V**. Although protonation is viable, the known propensity of such anions to facilitate β-fluoride elimination prevails (*75*), thus producing the desired *gem*-difluoroalkene by a radical/polar crossover process. Competitive hydrogen abstraction or single-electron transfer/protonation of **IV** would form Giese-type hydroalkylation product. This mechanistic proposal aligns with the following experimental observations: (i) The less electron-rich model **44** displayed lower reaction efficiency due to its relatively higher oxidation potential. (ii) The success of phenolic β-O-4 lignin model **49** might be attributed to the reversible PCET activation of phenolic hydroxyl bond since the hydrogen atom abstraction or back electron transfer of nonproductive phenoxy radical could regenerate the substrate. Moreover, the irreversible C─C bond cleavage of the benzylic alkoxy radical may facilitate the activation of the benzylic hydroxyl bond and subsequent functionalization processes (*36*).

## DISCUSSION

We have developed a photocatalytic protocol for the synthesis of functional group–rich *gem*-difluoroalkenes through the selective cleavage and defluorinative alkylation of lignin. This approach exhibits mild conditions, broad substrate scope, and high selectivity. (Phenolic) β-O-4 and β-1 lignin models, as well as native lignin containing distinct hydroxyl groups, can all be selectively cleaved and functionalized in a single step, forming *gem*-difluorobishomoallylic alcohols that are difficult to access using alternative synthons, thereby enhancing both structural and functional diversity. The synthetic potential of this method has been demonstrated by its compatibility with pharmacologically relevant motifs and the effective derivatization to interesting building blocks for medicinal chemistry. We believe that the insights gained here will inspire further development of methods for producing a wider range of value-added compounds from renewable feedstocks.

## MATERIALS AND METHODS

### General information

Unless otherwise stated, reactions were carried out using dry solvents under a nitrogen atmosphere. Starting materials were purchased from Aldrich, Fluka, and Tokyo Chemical Industry (TCI). Conversion was monitored by thin-layer chromatography (TLC) using Merck TLC silica gel 60 F254 and visualized by ultraviolet light at 254 nm. Flash column chromatography was performed over silica gel (230 to 400 mesh). Photochemical experiments have been performed using 40-W Kessil PR160 440-nm lamp. All nuclear magnetic resonance (NMR) spectra were recorded on AV2-400 Bruker spectrometers. Chemical shifts are given in parts per million (ppm), and the spectra are calibrated using the residual chloroform signals (7.26 ppm for ^1^H NMR and 77.0 ppm for ^13^C NMR) and the residual dichloromethane signals (2.05 ppm for ^1^H NMR and 29.84 ppm, for ^13^C NMR). Multiplicities are abbreviated as follows: singlet (s), doublet (d), triplet (t), quartet (q), doublet-doublet (dd), septet (sept), multiplet (m), and broad (b). Infrared spectra were recorded on a JASCO FT/IR-4100 spectrometer. Absorptions are reported in wave number (cm^−1^). High-resolution electrospray ionization and electron impact mass spectrometry were performed on a Finnigan MAT 900 (Thermo Finnigan, San Jose, CA, USA) double focusing magnetic sector mass spectrometer. Ten spectra were acquired. A mass accuracy of ≤2 ppm was obtained in the peak matching acquisition mode by using a solution containing two <l polyethylene glycol, molecular weight 200, two <l polypropylene glycol 450 (PPG450), and 1.5 mg of sodium acetate (NaOAc; all obtained from Sigma-Aldrich, CH-Buchs) dissolved in 100 ml of methanol [HPLC (high-performance liquid chromatography) Supra grade, Scharlau, E-Barcelona] as the internal standard.

### General procedure for the *gem*-difluoroalkenylation of lignin linkages

#### Method A

A screw cap 2-dram vial (5 ml) equipped with a stirring bar was charged with lignin model substrate (2.0 equiv, 0.1 mmol), CF_3_-substituted alkene (if solid, 1.0 equiv, 0.05 mmol), {Ir[dF(CF_3_)ppy]_2_[5,5′-d(CF_3_)bpy]}PF_6_ (0.0005 mmol, 1 mmol %), *^n^*Bu_4_NOAc (1.0 equiv, 0.05 mmol), and Na_2_HPO_4_ (1.0 equiv, 0.05 mmol). The vial was sealed, then evacuated, and refilled with N_2_ three times. Dry DMSO (0.05 M, 1.0 ml) and CF_3_-substituted alkene (if liquid, 1.0 equiv, 0.05 mmol) were added. The reaction mixture was irradiated with a 40-W Kessil PR160 440-nm lamp at 25°C with a distance of around 2 cm from the surface of the reaction vial. After 24 hours of irradiation, water (1 ml) was added, and the resulting mixture was extracted with ethyl acetate (EtOAc; 5 × 2 ml). The organic layer was washed with brine (3 × 8 ml) and dried over anhydrous MgSO_4_, filtered, and concentrated under reduced pressure. The ratio of *gem*-difluoroalkenylation product and hydroalkylation by-product was determined by crude ^19^F NMR analysis. The crude mixture was purified by chromatography on silica gel with hexane:ethyl acetate mixtures as eluent to give the corresponding products.

#### Method B

A screw cap 2-dram vial (5 ml) equipped with a stirring bar was charged with phenolic lignin model **49** (2.0 equiv, 0.1 mmol), CF_3_-substituted alkene (if solid, 1.0 equiv, 0.05 mmol), {Ir[dF(CF_3_)ppy]_2_(dtbbpy)}PF_6_ (0.0005 mmol, 1 mmol %), *^n^*Bu_4_NPO_4_H_2_ (1.0 equiv, 0.05 mmol), and Na_2_HPO_4_ (1.0 equiv, 0.05 mmol). The vial was sealed, then evacuated, and refilled with N_2_ three times. Dry acetone (0.05 M, 1.0 ml) and CF_3_-substituted alkene (if liquid, 1.0 equiv, 0.05 mmol) were added. The reaction mixture was irradiated with a 40-W Kessil PR160 440-nm lamp at 25°C with a distance of around 2 cm from the surface of the reaction vial. After 24 hours of irradiation, the resulting mixture was concentrated under reduced pressure. The ratio of *gem*-difluoroalkenylation product and hydroalkylation by-product was determined by crude ^19^F NMR analysis. The crude mixture was purified by chromatography on silica gel with hexane:ethyl acetate mixtures as eluent to give the corresponding products.

## Supplementary Materials

### This PDF file includes:

Supplementary Text Tables S1 to S9 Figs. S1 to S14 NMR spectra References
