# Supplementary material for: Redox-neutral photocatalytic cleavage and gem-difluoroalkenylation of lignin linkages
Source: Sci Adv. 2025 Aug 15;11(33):eady2227. doi: 10.1126/sciadv.ady2227 (PMC12356238; doi:10.1126/sciadv.ady2227)
Supplement: Supplementary file 1 — Supplementary Text Tables S1 to S9 Figs. S1 to S14 NMR spectra References [file sciadv.ady2227_sm.pdf]

Science Advances
AAAS

Supplementary Materials for

Redox-neutral photocatalytic cleavage and *gem*-difluoroalkenylation of lignin linkages

Xia Hu *et al.*

Corresponding author: Cristina Nevado, cristina.nevado@chem.uzh.ch

Sci. Adv. **11**, eady2227 (2025)
DOI: 10.1126/sciadv.ady2227

This PDF file includes:

Supplementary Text
Tables S1 to S9
Figs. S1 to S14
NMR spectra
References

1. General information

Unless otherwise stated, reactions were carried out using dry solvents under a nitrogen atmosphere. Starting materials were purchased from Aldrich, Fluka and TCI. Conversion was monitored by thin layer chromatography (TLC) using Merck TLC silica gel 60 F254 and visualized by UV light at 254 nm. Flash column chromatography was performed over silica gel (230-400 mesh). Photochemical experiments have been performed using 40 W Kessil® PR160-440 nm lamp. All NMR spectra were recorded on AV2-400 Bruker spectrometers. Chemical shifts are given in ppm and the spectra are calibrated using the residual chloroform signals (7.26 ppm for ^{1}H NMR and 77.0 ppm for ^{13}C NMR) and the residual dichloromethane signals (2.05 ppm for ^{1}H NMR and 29.84 ppm, for ^{13}C NMR). Multiplicities are abbreviated as follows: singlet (s), doublet (d), triplet (t), quartet (q), doublet-doublet (dd), septet (sept), multiplet (m), and broad (b). Infrared spectra were recorded on a JASCO FT/IR-4100 spectrometer. Absorptions are reported in wavenumber (cm^{-1}). High-resolution electrospray ionization and electron impact mass spectrometry was performed on a Finnigan MAT 900 (Thermo Finnigan, San Jose, CA; USA) double focusing magnetic sector mass spectrometer. Ten spectra were acquired. A mass accuracy ≤ 2 ppm was obtained in the peak matching acquisition mode by using a solution containing 2 <l PEG200, 2 <l PPG450, and 1.5 mg NaOAc (all obtained from Sigma-Aldrich, CH-Buchs) dissolved in 100 mL of MeOH (HPLC Supra grade, Scharlau, E-Barcelona) as internal standard.

2. Optimization of the reaction conditions

Table S1. Effect of solvents [a]

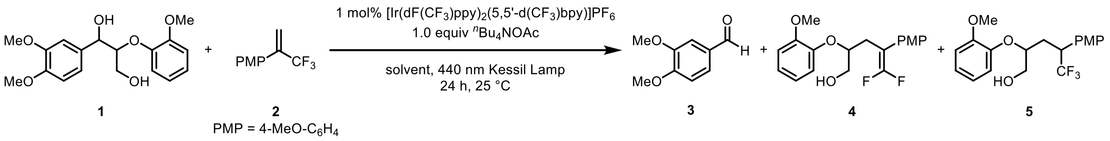

Entry	solvent	yield of **3** (%)[a]	yield of **4** (%)[a]	conversion of **1** (%)[a]	conversion of **2** (%)[a]	ratio of **4/5**[b]
1	acetone = 1mL	99 (99[c])	68 (65[c])	>99	60	6:1
2	DMSO = 1mL	71 (69[c])	51 (51[c])	92	71	33:1
3	DMF = 1mL	77 (78[c])	65 (63[c])	88	70	14:1
4	DMA = 1mL	80	65	88	65	13:1
5	THF = 1mL	65	48	77	35	5:1
6	1,4-dioxane = 1mL	22	20	34	50	10:1
7	CH_3CN = 1mL	80	58	90	53	13:1
8	DMSO/acetone = 0.5 mL/0.5 mL	80	68	88	63	17:1
9	DMSO/acetone = 0.6 mL/0.4 mL	73	58	84	85	20:1
10	DMSO/acetone = 0.4 mL/0.6 mL	81	74	84	85	14:1

[a]Reactions conditions: 1-(3,4-dimethoxyphenyl)-2-(2-methoxyphenoxy)propane-1,3-diol (**1**, 0.05 mmol, 1 equiv), 1-methoxy-4-(3,3,3-trifluoroprop-1-en-2-yl)benzene (**2**, 0.1 mmol, 2 equiv), $[Ir(dF(CF_3)ppy)_2(5,5'\text{-}d(CF_3)bpy)]PF_6$ (0.0005 mmol, 1 mol %), $^{n}Bu_4NOAc$ (0.05 mmol, 1 equiv), solvent = 1.0 mL, 40 W 440 nm Kessil Lamp, N_2, 25 °C, 24 h. Yield was determined by ^{1}H NMR by using 1,3,5-trimethoxybenzene as internal standard. [b]Ratio was determined by ^{19}F NMR. [c]Isolated yield.

Table S2. Effect of bases [a]

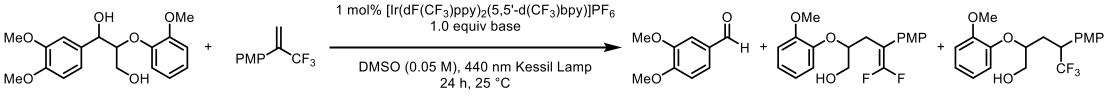

1 2 3 4 5

PMP = 4-MeO-C_6H_4

Entry	base	yield of **3** (%)[a]	yield of **4** (%)[a]	conversion of **1** (%)[a]	conversion of **2** (%)[a]	ratio of **4/5**[b]
1	^{n}Bu$_4$NOAc	71 (69[c])	51 (51[c])	92	71	33:1
2	^{n}Bu$_4$NOBz	67	46	88	69	33:1
3	^{n}Bu$_4$NPO$_4$H$_2$	78	60	96	69	27:1
4	^{n}Bu$_4$NCOOCF$_3$	13	trace	61	53	--
5	^{n}Bu$_4$NOP(O^{n}Bu)$_2$	51	trace	84	61	--
6	2,4,6-collidine	5	n.d.	37	22	--

[a]Reactions conditions: 1-(3,4-dimethoxyphenyl)-2-(2-methoxyphenoxy)propane-1,3-diol (**1**, 0.05 mmol, 1 equiv), 1-methoxy-4-(3,3,3-trifluoroprop-1-en-2-yl)benzene (**2**, 0.1 mmol, 2 equiv), [Ir(dF(CF$_3$)ppy)$_2$(5,5'-d(CF$_3$)bpy)]PF$_6$ (0.0005 mmol, 1 mol %), base (0.05 mmol, 1 equiv), DMSO = 1.0 mL, 40 W 440 nm Kessil Lamp, N_2, 25 °C, 24 h. Yield was determined by ^{1}H NMR by using 1,3,5-trimethoxybenzene as internal standard. [b]Ratio was determined by ^{19}F NMR. [c]Isolated yield.

Table S3. Effect of the ratio of starting materials [a]

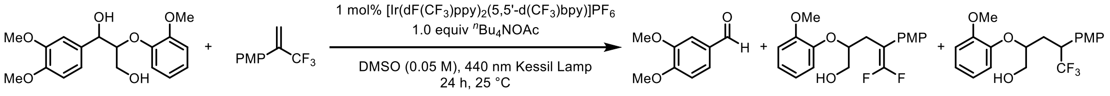

1, x mmol **2**, y mmol **3** **4** **5**

PMP = 4-MeO-C_6H_4

Entry	x/y	yield of **3** (%)[a]	yield of **4** (%)[a]	conversion of **1** (%)[a]	conversion of **2** (%)[a]	ratio of **4/5**[b]
1	0.05 / 0.1	71 (69[c])	51 (51[c])	92	71	33:1
2	0.075 / 0.05	87	68	70	97	33:1
3	0.1 / 0.05	90	79	58	90	33:1

[a]Reactions conditions: 1-(3,4-dimethoxyphenyl)-2-(2-methoxyphenoxy)propane-1,3-diol (**1**, x mmol), 1-methoxy-4-(3,3,3-trifluoroprop-1-en-2-yl)benzene (**2**, y mmol), [Ir(dF(CF$_3$)ppy)$_2$(5,5'-d(CF$_3$)bpy)]PF$_6$ (0.0005 mmol, 1 mol %), ^{n}Bu$_4$NOAc (0.05 mmol, 1 equiv), DMSO = 1.0 mL, 40 W 440 nm Kessil Lamp, N_2, 25 °C, 24 h. Yield was determined by ^{1}H NMR by using 1,3,5-trimethoxybenzene as internal standard. [b]Ratio was determined by ^{19}F NMR. cIsolated yield.

Table S4. Effect of additives [a]

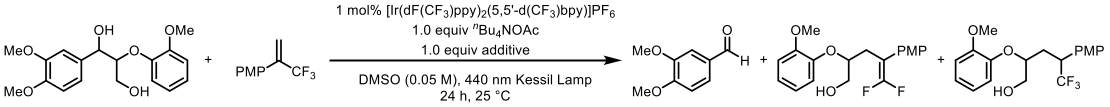

1 2 3 4 5

PMP = 4-MeO-C_6H_4

Entry	additive	yield of **3** (%)[a]	yield of **4** (%)[a]	conversion of **1** (%)[a]	conversion of **2** (%)[a]	ratio of **4/5**[b]
1	without additive	90	79	58	90	33:1
2	NaH$_2$PO$_4$	92	80	65	95	33:1
3	Na$_2$HPO$_4$	>99 (99[c])	85 (84[c])	56	>99	37:1
4	K$_2$HPO$_4$	92	74	67	>99	37:1
5	Na$_3$PO$_4$	86	77	60	>99	37:1
6	Na$_2$HPO$_4$-2H$_2$O	95 (90[c])	83 (81[c])	55	95	37:1
7	tetramethyl piperidine	66	56	51	94	33:1
8	quinuclidine	34	28	30	95	33:1

[a]Reactions conditions: 1-(3,4-dimethoxyphenyl)-2-(2-methoxyphenoxy)propane-1,3-diol (**1**, 0.1 mmol, 2 equiv), 1-methoxy-4-(3,3,3-trifluoroprop-1-en-2-yl)benzene (**2**, 0.05 mmol, 1 equiv), [Ir(dF(CF$_3$)ppy)$_2$(5,5'-d(CF$_3$)bpy)]PF$_6$ (0.0005 mmol, 1 mol %), ^{n}Bu$_4$NOAc (0.05 mmol, 1 equiv), additive (0.05 mmol, 1 equiv), DMSO = 1.0 mL, 40 W 440 nm Kessil Lamp, N_2, 25 °C, 24 h. Yield was determined by ^{1}H NMR by using 1,3,5-trimethoxybenzene as internal standard. [b]Ratio was determined by ^{19}F NMR. [c]Isolated yield.

Table S5. Effect of photocatalysts [a]

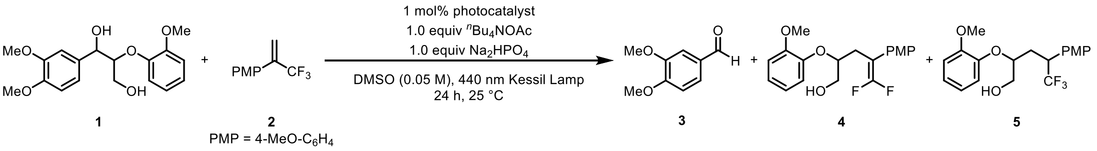

Entry	photocatalyst	yield of **3** (%)[a]	yield of **4** (%)[a]	conversion of **1** (%)[a]	conversion of **2** (%)[a]	ratio of **4/5**[b]
1	[Ir(dF(CF$_3$)ppy)$_2$(5,5'-d(CF$_3$)bpy)]PF$_6$	>99 (99[c])	85 (84[c])	56	>99	37:1
2	[Ir(dF(CF$_3$)ppy)$_2$(dtbbpy)]PF$_6$	>99	85	71	>99	37:1
3	[Ir(dF(CF$_3$)ppy)$_2$(bpy)]PF$_6$	72	62	60	>99	37:1
4	[Ir(dF(Me)ppy)$_2$(dtbbpy)]PF$_6$	>99	72	77	>99	37:1
5	[Ir(dtbbpy)(bpy)$_2$]PF$_6$	57	50	50	>99	37:1
6	4CzIPN	37	25	86	>99	27:1
7	Mes-3,6-^{t}Bu$_2$-Acr-PhBF$_4$	11	8	20	94	19:1

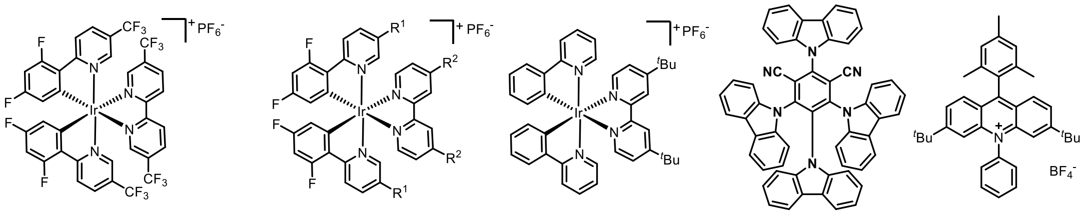

[Ir(dF(CF$_3$)ppy)$_2$(5,5'-d(CF$_3$)bpy)]PF$_6$ — R^1 = CF$_3$, R^2 = ^{t}Bu, [Ir(dF(CF$_3$)ppy)$_2$(dtbbpy)]PF$_6$; R^1 = CF$_3$, R^2 = H, [Ir(dF(CF$_3$)ppy)$_2$(bpy)]PF$_6$; R^1 = Me, R^2 = ^{t}Bu, [Ir(dF(Me)ppy)$_2$(dtbbpy)]PF$_6$ — [Ir(dtbbpy)(bpy)$_2$]PF$_6$ — 4-CzIPN — Mes-3,6-^{t}Bu$_2$-Acr-PhBF$_4$

[a]Reactions conditions: 1-(3,4-dimethoxyphenyl)-2-(2-methoxyphenoxy)propane-1,3-diol (**1**, 0.1 mmol, 2 equiv), 1-methoxy-4-(3,3,3-trifluoroprop-1-en-2-yl)benzene (**2**, 0.05 mmol, 1 equiv), photocatalyst (0.0005 mmol, 1 mol %), ^{n}Bu$_4$NOAc (0.05 mmol, 1 equiv), Na$_2$HPO$_4$ (0.05 mmol, 1 equiv), DMSO = 1.0 mL, 40 W 440 nm Kessil Lamp, N$_2$, 25 °C, 24 h. Yield was determined by ^{1}H NMR by using 1,3,5-trimethoxybenzene as internal standard. [b]Ratio was determined by ^{19}F NMR. [c]Isolated yield.

Table S6. Control experiments [a]

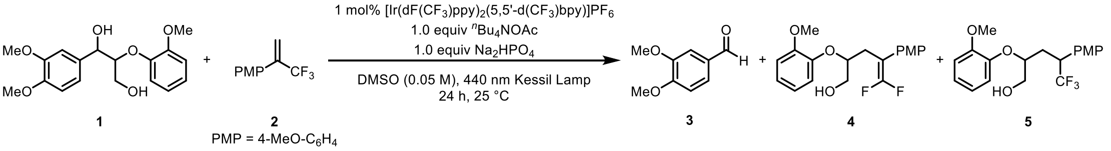

Entry	Deviation from standard conditions	yield of **1** (%)[a]	yield of **4** (%)[a]	conversion of **1** (%)[a]	conversion of **2** (%)[a]	ratio of **4/5**[b]
1	none	>99 (99[c])	85 (84[c])	56	>99	37:1
2	without photocatalyst	n.d.	n.d.	0	0	--
3	without ^{n}Bu$_4$NOAc	44	trace	42	80	--
4	without light	n.d.	n.d.	0	0	--

[a]Reactions conditions: 1-(3,4-dimethoxyphenyl)-2-(2-methoxyphenoxy)propane-1,3-diol (**1**, 0.1 mmol, 2 equiv), 1-methoxy-4-(3,3,3-trifluoroprop-1-en-2-yl)benzene (**2**, 0.05 mmol, 1 equiv), [Ir(dF(CF$_3$)ppy)$_2$(5,5'-d(CF$_3$)bpy)]PF$_6$ (0.0005 mmol, 1 mol %), ^{n}Bu$_4$NOAc (0.05 mmol, 1 equiv), Na$_2$HPO$_4$ (0.05 mmol, 1 equiv), DMSO = 1.0 mL, 40 W 440 nm Kessil Lamp, N$_2$, 25 °C, 24 h. Yield was determined by ^{1}H NMR by using 1,3,5-trimethoxybenzene as internal standard. [b]Ratio was determined by ^{19}F NMR. [c]Isolated yield.

Table S7. Compatibility of 1 under Tian's[39] or Wallentin's[40] reaction conditions

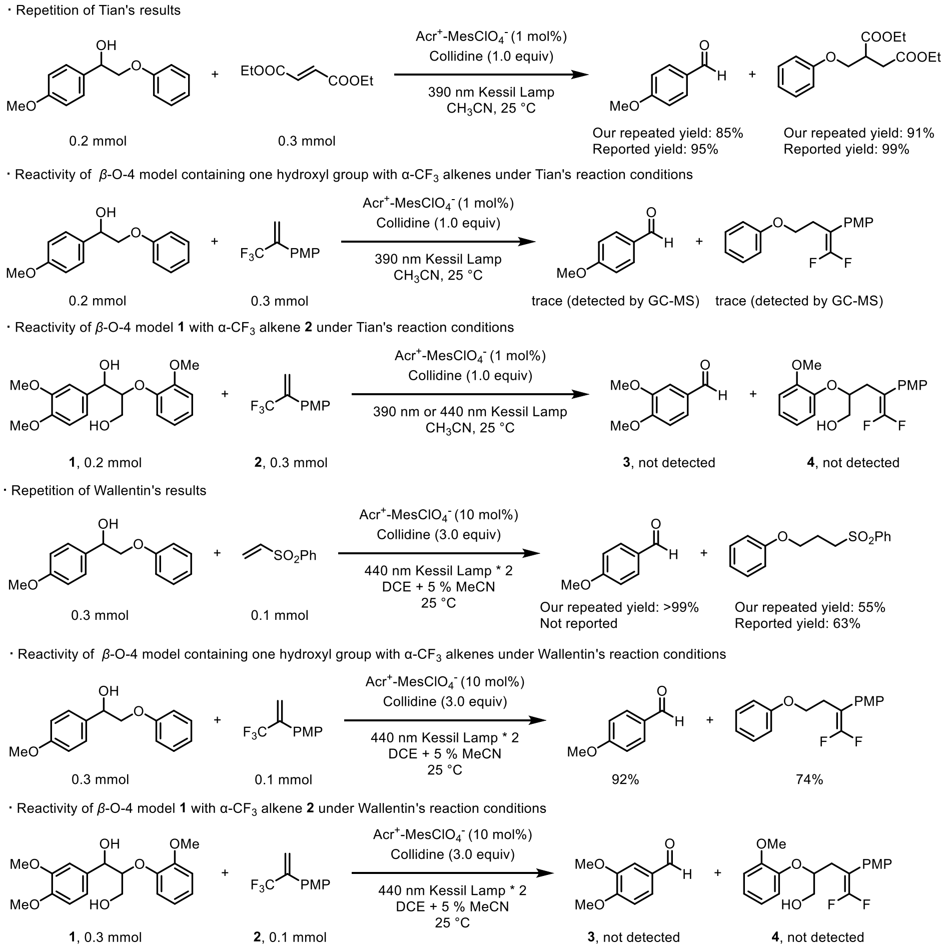

Compared to the reaction conditions reported by Tian and Wallentin, it seems that one of the key factors contributing to the success of our transformation is the use of photocatalysts with more negative reduction potentials, which are capable of reducing intermediate **IV** to **V**. This notion is supported by the reduction potentials of $[Ir(dF(CF_3)ppy)_2(5,5'\text{-}d(CF_3)bpy]PF_6$ and $[Ir(dF(CF_3)ppy)_2(dtbbpy)]PF_6$ which are -0.69V and -1.37V, respectively in contrast to the reduced form of the photocatalyst $Acr^+\text{-}MesClO_4^-$ which exhibits a less negative reduction potential of -0.49V. [76]

Table S8. Optimization of phenolic β-O-4 model [a]

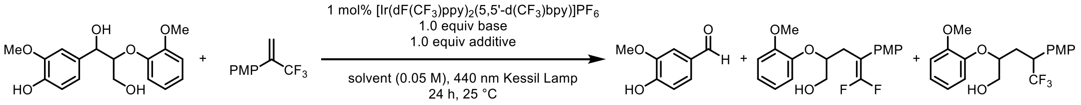

49 **2** **50** **4** **5**

PMP = 4-MeO-C$_6$H$_4$

Entry	base+additive	solvent	yield of **50** (%)[a]	yield of **4** (%)[a]	conversion of **49** (%)[a]	conversion of **2** (%)[a]	ratio of **4/5**[b]
1	1.0 equiv ^{n}Bu$_4$NOAc + 1.0 equiv. Na$_2$HPO$_4$	DMSO	4	not detected	5	10	--
2	1.0 equiv ^{n}Bu$_4$NOAc + without additive	THF	37	35	25	52	6:1
3	1.0 equiv ^{n}Bu$_4$NPO$_4$H$_2$ + without additive	THF	41	39	39	45	5:1
4	1.0 equiv ^{n}Bu$_4$NPO$_4$H$_2$ + without additive	MeCN	12	trace	12	38	--
5	1.0 equiv ^{n}Bu$_4$NPO$_4$H$_2$ + without additive	acetone	60	48	44	84	6:1
6	1.0 equiv ^{n}Bu$_4$NPO$_4$H$_2$ + 1.0 equiv. Na$_2$HPO$_4$	acetone	68 (66[c])	53 (47[c])	36	92	6:1
7	1.0 equiv ^{n}Bu$_4$NPO$_4$H$_2$ + 1.0 equiv. Na$_2$HPO$_4$	THF	51	47	31	56	5:1
8	1.0 equiv ^{n}Bu$_4$NOAc + 1.0 equiv. Na$_2$HPO$_4$	acetone	58	51	44	84	6:1
9	1.0 equiv ^{n}Bu$_4$NOAc + 1.0 equiv. Na$_2$HPO$_4$	THF	40	38	28	50	5:1
10[d]	1.0 equiv ^{n}Bu$_4$NPO$_4$H$_2$ + 1.0 equiv. Na$_2$HPO$_4$	acetone	73 (72[c])	65 (63[c])	64	>99%	6:1

[a]Reactions conditions: 1-(4-hydroxy-3-methoxyphenyl)-2-(2-methoxyphenoxy)propane-1,3-diol (**49**, 0.1 mmol, 2 equiv), 1-methoxy-4-(3,3,3-trifluoroprop-1-en-2-yl)benzene (**2**, 0.05 mmol, 1 equiv), [Ir(dF(CF$_3$)ppy)$_2$(5,5'-d(CF$_3$)bpy)]PF$_6$ (0.0005 mmol, 1 mol %), base (0.05 mmol, 1 equiv), additive (0.05 mmol, 1 equiv), solvent = 1.0 mL, 40 W 440 nm Kessil Lamp, N$_2$, 25 °C, 24 h. Yield was determined by ^{1}H NMR by using 1,3,5-trimethoxybenzene as internal standard. [b]Ratio was determined by ^{19}F NMR. [c]Isolated yield. [d][Ir(dF(CF$_3$)ppy)$_2$(dtbbpy)]PF$_6$ was used as photocatalyst.

Table S9. Optimization of β-bromostyrene [a]

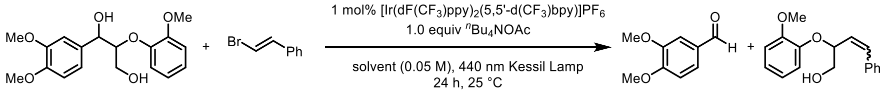

1, x mmol y mmol **3** **53**

Entry	photocatalyst	solvent	x/y	yield of **3** (%)[a]	yield of **53** (%)[a]	conversion of **1** (%)[a]
1	[Ir(dF(CF$_3$)ppy)$_2$(5,5'-d(CF$_3$)bpy)]PF$_6$	DMSO	0.1/ 0.05	50	24 (Z/E=3:1)	31
2	[Ir(dF(CF$_3$)ppy)$_2$(5,5'-d(CF$_3$)bpy)]PF$_6$	Acetone	0.1/ 0.05	64	33 (Z/E=3:1)	37
3	[Ir(dF(CF$_3$)ppy)$_2$(5,5'-d(CF$_3$)bpy)]PF$_6$	DMSO	0.05/ 0.1	40	24 (Z/E=3:1)	44
4	[Ir(dF(CF$_3$)ppy)$_2$(5,5'-d(CF$_3$)bpy)]PF$_6$	Acetone	0.05/ 0.1	61	36 (Z/E=3:1)	72
5	[Ir(dF(CF$_3$)ppy)$_2$(dtbbpy)]PF$_6$	Acetone	0.1/ 0.05	66	40 (Z/E=4:1)	39
6	[Ir(dF(CF$_3$)ppy)$_2$(dtbbpy)]PF$_6$	Acetone	0.05/ 0.1	65 (60[b])	42 (41[b], Z/E=5:1)	75

[a]Reactions conditions: 1-(3,4-dimethoxyphenyl)-2-(2-methoxyphenoxy)propane-1,3-diol (**1**, x mmol), β-bromostyrene (y mmol), photocatalyst (0.0005 mmol, 1 mol %), ^{n}Bu$_4$NOAc (0.05 mmol, 1 equiv), Na$_2$HPO$_4$ (0.05 mmol, 1 equiv), solvent = 1.0 mL, 40 W 440 nm Kessil Lamp, N$_2$, 25 °C, 24 h. Yield was determined by ^{1}H NMR by using 1,3,5-trimethoxybenzene as internal standard. [b]Isolated yield.

3. Experimental procedures for the preparation of trifluoromethyl-substituted alkenes

General Procedure:

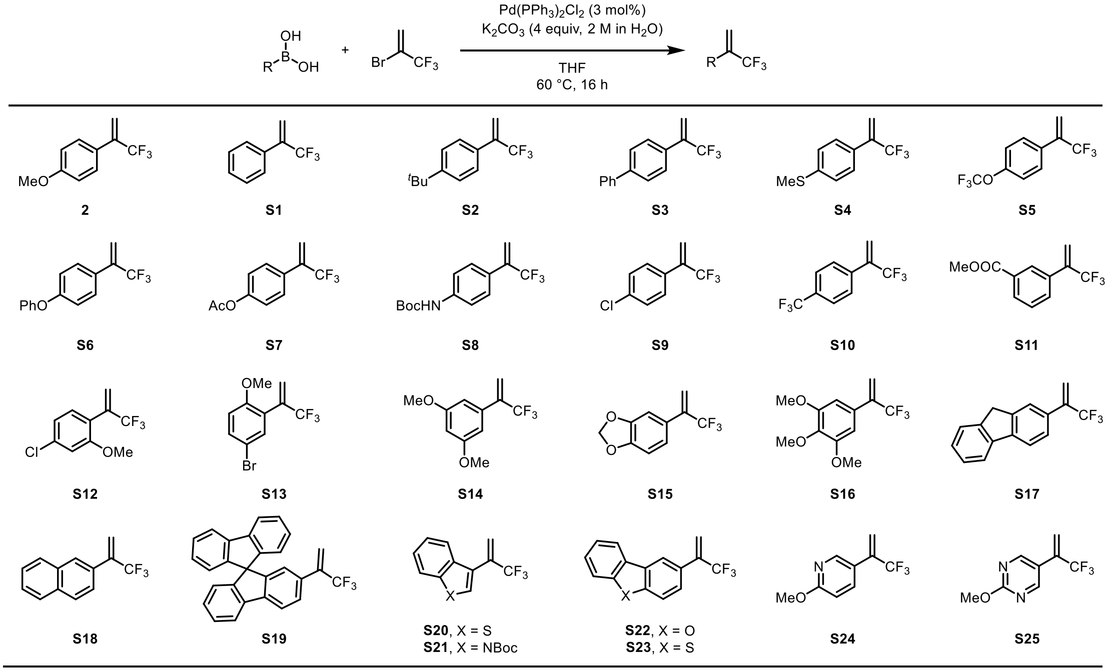

A Schlenk tube equipped with a stirring bar was charged with boronic acid (1.0 equiv) and $Pd(PPh_3)_2Cl_2$ (3 mol%). The vessel was evacuated and filled with N_2 three times, then THF (5 mL/mol boronic acid) and aqueous K_2CO_3 (4 equiv, 2.0 M in H_2O) were added. After the addition of 2-bromo-3,3,3-trifluoropropene (2.0 equiv), the reaction was stirred at 60 °C for 16 h under N_2 atmosphere. After this time, the resulting mixture was cooled to room temperature, quenched with saturated aqueous NH_4Cl, and extracted with EtOAc three times. The combined organic phases were dried over anhydrous $MgSO_4$, filtered, and concentrated under reduced pressure. The residue was purified by column chromatography on silica gel (hexane/ethyl acetate) to give the desired trifluoromethylated alkene.

2[77], **S1**[78], **S2**[60], **S3**[77], **S4**[77], **S5**[59], **S6**[77], **S8**[79], **S9**[60], **S10**[77], **S11**[79], **S13**[80], **S14**[59], **S15**[59], **S16**[81], **S18**[78], **S19**[80], **S20**[78], **S21**[60], **S22**[78], **S23**[80], **S24**[59], **S25**[59] were prepared according to the above procedure and analytical data is in agreement with previously reported values.

4-(3,3,3-Trifluoroprop-1-en-2-yl)phenyl acetate (S7)

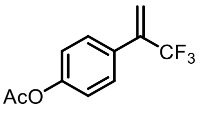

Colorless oil, 587 mg (3 mmol scale), 85% yield. ^{1}H NMR (400 MHz, $CDCl_3$) δ 7.51 – 7.44 (m, 2H), 7.15 – 7.09 (m, 2H), 5.96 (q, J = 1.3 Hz, 1H), 5.76 (q, J = 1.7 Hz, 1H), 2.31 (s, 3H); ^{13}C NMR (101 MHz, $CDCl_3$) δ 169.2, 151.2, 138.1 (q, J = 30.4 Hz), 131.2, 128.6 (d, J = 0.8 Hz), 123.2 (q, J = 275.0 Hz), 121.7, 120.7 (q, J = 5.8 Hz), 21.1; ^{19}F NMR (377 MHz, $CDCl_3$) δ -64.92. IR (film): ν (cm^{-1}) 2931, 2857, 2118, 1761, 1606, 1510, 1370, 1352, 1189, 1164, 1121, 1077, 1016, 948, 910, 859, 843, 750, 660, 609, 524; HR-MS (EI) m/z calcd for $C_{11}H_9F_3O_2^+$ [M^+] 230.05492, found 230.05507.

4-Chloro-2-methoxy-1-(3,3,3-trifluoroprop-1-en-2-yl)benzene (S12)

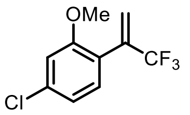

Colorless oil, 652 mg (3 mmol scale), 92% yield. ^{1}H NMR (400 MHz, $CDCl_3$) δ 7.30 (dd, *J* = 8.8, 2.7 Hz, 1H), 7.20 (d, *J* = 2.6 Hz, 1H), 6.86 (d, *J* = 8.8 Hz, 1H), 6.10 (q, *J* = 1.4 Hz, 1H), 5.65 (q, *J* = 1.1 Hz, 1H), 3.80 (s, 3H); ^{13}C NMR (101 MHz, $CDCl_3$) δ 156.1, 135.0 (q, *J* = 32.2 Hz), 130.5, 129.9, 125.3, 124.7, 124.0 (q, *J* = 5.2 Hz), 122.8 (q, *J* = 275.0 Hz), 112.4, 56.0; ^{19}F NMR (377 MHz, $CDCl_3$) δ -65.69. IR (film): ν (cm^{-1}) 3009, 2942, 2843, 1596, 1490, 1463, 1392, 1333, 1284, 1249, 1167, 1122, 1071, 1028, 954, 887, 811, 698, 642, 632, 550; HR-MS (EI) m/z calcd for $C_{10}H_8ClF_3O^+$ [M^+] 236.02103, found 236.02116.

2-(3,3,3-Trifluoroprop-1-en-2-yl)-9H-fluorene (S17)

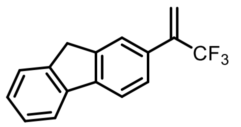

White solid, 689 mg (3 mmol scale), 88% yield. ^{1}H NMR (400 MHz, $CDCl_3$) δ 7.83 – 7.76 (m, 2H), 7.67 – 7.63 (m, 1H), 7.59 – 7.54 (m, 1H), 7.53 – 7.46 (m, 1H), 7.43 – 7.37 (m, 1H), 7.34 (td, *J* = 7.4, 1.2 Hz, 1H), 5.98 (dd, *J* = 2.6, 1.3 Hz, 1H), 5.83 (q, *J* = 1.7 Hz, 1H), 3.93 (s, 2H); ^{13}C NMR (101 MHz, $CDCl_3$) δ 143.6, 143.5, 142.5, 140.9, 139.2 (q, *J* = 3.7 Hz), 132.0, 127.2, 126.9, 126.2, 125.1, 124.0 (d, *J* = 0.7 Hz), 123.5 (q, *J* = 275.1 Hz), 120.2, 120.0 (q, *J* = 5.9 Hz), 119.8, 36.9; ^{19}F NMR (377 MHz, $CDCl_3$) δ -64.47. IR (film): ν (cm^{-1}) 3073, 3012, 1911, 1456, 1401, 1343, 1300, 1280, 1236, 1210, 1174, 1160, 1146, 1114, 1077, 952, 879, 841, 831, 774, 743, 714, 631, 619, 568, 478, 415; HR-MS (EI) m/z calcd for $C_{16}H_{11}F_3^+$ [M^+] 260.08074, found 260.08090.

<u>Synthesis of S26:</u>

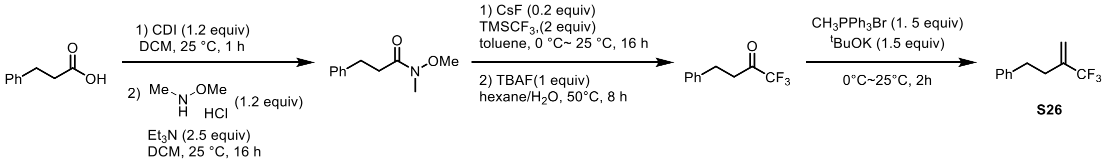

Step 1: A round bottom flask equipped with a stirring bar was charged with 3-phenylpropionic acid (50 mmol, 1.0 equiv) and DCM (150 mL). To this stirred solution was added 1,1′- carbonyldiimidazole (CDI, 1.2 equiv) in one portion. The reaction mixture was allowed to stir at 25 °C for 1 h. After this time, *N*-*O*-dimethylhydroxylamine hydrochloride (1.2 equiv) and Et_3N (2.5 equiv) were added at once, and the reaction mixture was stirred for 16 h. The reaction mixture was then quenched with 2 M HCl (125 mL) and stirred vigorously for 10 min. After this time, the solution was transferred to a separatory funnel, and the layers were separated. The aqueous phase was extracted with DCM (3 × 75 mL). The combined organic phase was washed with 2 M HCl, H_2O, saturated $NaHCO_3$, and saturated brine, dried over $MgSO_4$, filtered, and concentrated under reduced pressure. The crude was directly used into the next step without further purification.

Step 2: A round bottom flask equipped with a stirring bar was charged with *N*-methoxy-*N*-methyl-3-phenylpropanamide (30 mmol, 1 equiv), CsF (0.2 equiv) and dry toluene (60 mL). The flask was cooled to 0 °C for 15 min. TMS-CF_3 (2 equiv) was added to the reaction mixture dropwise over a period of 10 min. After completion of the addition, the reaction mixture was allowed to stir for 10 min at 0 °C. The cooling bath was removed, and the reaction mixture was allowed to stir at 25 °C for 16 h. Once complete

conversion to the silylated, tetrahedral intermediate was confirmed, toluene was removed in vacuo. Hexanes (30 mL), followed by deionized H_2O (30 mL) and TBAF (1 M in THF, 1 equiv) were added to the reaction mixture. The flask was equipped with an air-cooled reflux condenser and then heated to 50 °C for 8 h to facilitate cleavage of the silyl ether. The reaction was cooled to room temperature after it was complete. The reaction mixture was then diluted with Et_2O and H_2O, and transferred to a separatory funnel. The layers were separated, and the aqueous phase was extracted with Et_2O (3 × 60 mL). The combined organic layers were washed with 2 M HCl, H_2O, and saturated brine, dried over $MgSO_4$, filtered, and concentrated under reduced pressured. The crude material was purified by chromatography on silica gel to give the corresponding trifluoromethyl ketone.

Step 3: A three-neck round bottom flask equipped with a stirring bar was charged with ^{t}BuOK (1.5 equiv) and Ph_3PCH_3Br (1.5 equiv), THF (40 mL) was added at 0 °C under N_2 atmosphere. The mixture was stirred at 0 °C for 1 h. After this time, a solution of 1,1,1-trifluoro-4-phenylbutan-2-one (10 mmol, 1 equiv) in dry THF was added slowly to the mixture at 0 °C. The resulting mixture was warmed to room temperature and stirred for 2 h. After the reaction was complete, the resulting mixture was filtered through celite and washed with EtOAc twice. The filtrate was concentrated under reduced pressure. The residue was purified by column chromatography on silica gel (pentane) to give **S26**. Analytical data is in agreement with previously reported values. [82]

Synthesis of S27:

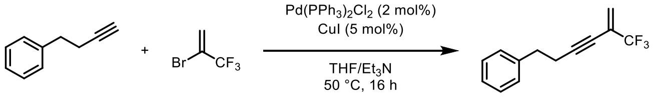

S27

A Schlenk tube equipped with a stirring bar was charged with $Pd(PPh_3)_2Cl_2$ (2 mol%) and CuI (5 mol%). The vessel was evacuated and filled with N_2 three times, then THF (10 mL) and Et_3N (10 mL) were added. After the addition of but-3-yn-1-ylbenzene (1.0 equiv) and 2-bromo-3,3,3-trifluoropropene (2.0 equiv), the reaction was stirred at 50 °C for 16 h under N_2 atmosphere. After this time, the resulting mixture was cooled to room temperature, filtered through celite and washed with EtOAc twice. The filtrate was concentrated under reduced pressure. The residue was purified by column chromatography on silica gel (hexane/ethyl acetate) to give **S27**.

(5-(Trifluoromethyl)hex-5-en-3-yn-1-yl)benzene (S27)

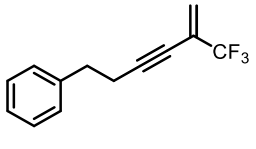

Colorless oil, 930 mg (5 mmol scale), 83% yield. ^{1}H NMR (400 MHz, $CDCl_3$) δ 7.31 – 7.23 (m, 2H), 7.22 – 7.15 (m, 3H), 5.92 (d, *J* = 1.2 Hz, 1H), 5.73 – 5.68 (m, 1H), 2.84 (t, *J* = 7.5 Hz, 2H), 2.60 (t, *J* = 7.5 Hz, 2H); ^{13}C NMR (101 MHz, $CDCl_3$) δ 140.1, 128.5, 128.4, 126.4, 125.9 (q, *J* = 4.5 Hz), 122.7 (q, *J* = 35.1 Hz), 121.4 (q, *J* = 274.4 Hz), 94.1, 74.0 (q, *J* = 1.6 Hz), 34.5, 21.5; ^{19}F NMR (377 MHz, $CDCl_3$) δ -68.29. IR (film): ν (cm^{-1}) 3064, 3030, 2930, 2242, 1878, 1497, 1454, 1400, 1360, 1336, 1175, 1158, 1136, 1057, 938, 749, 697, 628, 581, 511, 501; HR-MS (EI) m/z calcd for $C_{13}H_{11}F_3^+$ [M^+] 224.08074, found 224.08043.

Synthesis of S28:

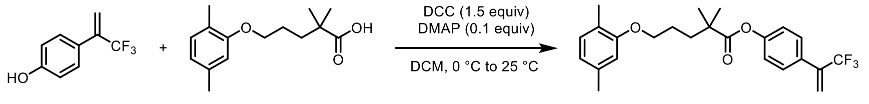

S28

A round bottom flask equipped with a stirring bar was charged with 4-(3,3,3-trifluoroprop-1-en-2-yl)phenol (2 mmol, 1 equiv), DCC (1.5 equiv), DMAP (10 mol%) and DCM (10 mL). Gemfibrozil (1.1 equiv) was added at 0 °C under N_2 atmosphere. The reaction was stirred at 25 °C for 3 h. After this time, the resulting mixture was filtered through celite and washed with DCM twice. The filtrate was concentrated under reduced pressure. The residue was purified by column chromatography on silica gel (hexane/ethyl acetate) to give **S28**. Analytical data is in agreement with previously reported values.[83]

4. Experimental procedures for the preparation of lignin models

Synthesis of *β*-O-4 lignin models:

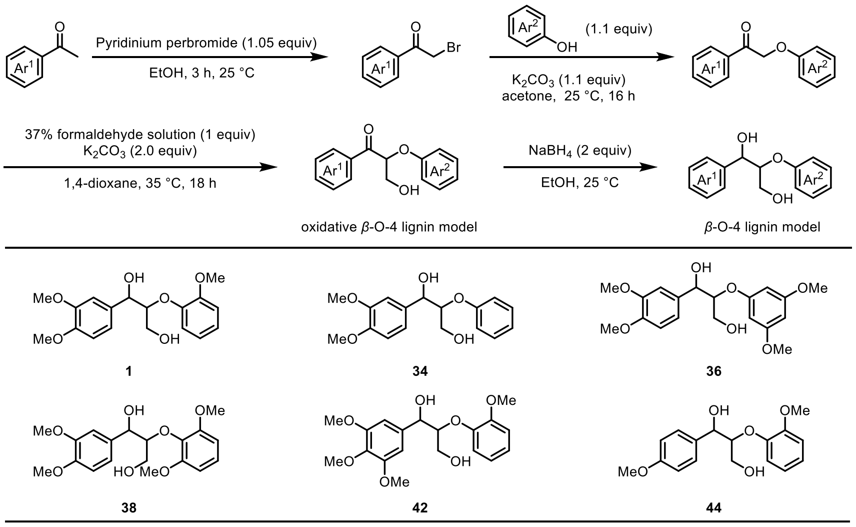

Step 1: An oven-dried flask was charged with ketone (1.0 equiv) and EtOAc (1 M), then pyridinium hydrobromide perbromide (1.05 equiv) was added. The reaction mixture was stirred for 3 h at 25 °C. After this time, the resulting mixture was filtered through celite and washed with EtOAc twice. The filtrate was concentrated, and the crude material was purified by chromatography on silica gel to give the corresponding α-bromoketone.

Step 2: An oven-dried flask was charged with α-bromoketone (1.0 equiv), phenol (1.1 equiv), K_2CO_3 (1.1 equiv) and acetone (0.5 M). The reaction mixture was stirred at 25 °C and monitored by TLC. After the reaction was complete, the resulting mixture was filtered through celite and washed with EtOAc twice. The filtrate was concentrated, and the crude material was purified by chromatography on silica gel to give the corresponding α-aryloxyketone.

Step 3: An oven-dried flask was charged with α-aryloxyketone (1.0 equiv) and dioxane (0.2 M). Then 37% formaldehyde solution (1 equiv) and K_2CO_3 (2.0 equiv) were added to the solution. The reaction mixture was stirred at 35 °C for 18 h. After this time, the

resulting mixture was filtered through celite and washed with EtOAc twice. The filtrate was concentrated and the crude material was purified by chromatography on silica gel to give the corresponding oxidative *β*-O-4 model.

Step 4: An oven-dried flask was charged with the oxidative *β*-O-4 model (1.0 equiv) and EtOH (0.1 M), then $NaBH_4$ (2.0 equiv) was added portionwise. The reaction mixture was stirred at 25 °C and monitored by TLC. After the reaction was complete, the solvent was evaporated under reduced pressure, EtOAc was added to dissolve the raw product. The corresponding organic solution was washed with saturated NH_4Cl, H_2O, and saturated brine, dried over $MgSO_4$, filtered, and concentrated under reduced pressured. The crude material was purified by chromatography on silica gel to give the corresponding *β*-O-4 lignin model.

1[84], **34**[84], **36**[85], **38**[84], **42**[86], **44**[84] were prepared according to the above procedure and analytical data is in agreement with previously reported values. **49** is commercially available from TCI.

Synthesis of lignin model 40:

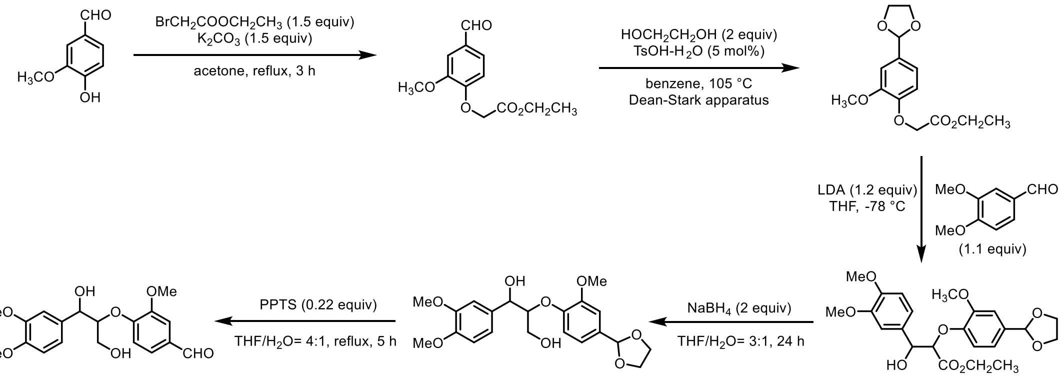

40

40 was prepared according to the literature procedure[87] and analytical data is in agreement with previously reported values.

Synthesis of *β*-1 lignin models:

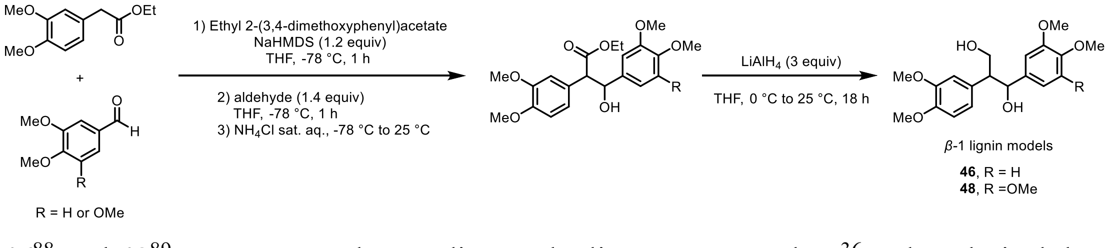

46[88] and **48**[89] were prepared according to the literature procedure[36] and analytical data is in agreement with previously reported values.

5. Experimental procedures and characterization data of products

General procedure A (GP-A):

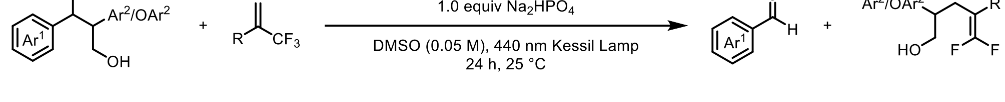

GP-A: A screw cap 2-dram vial (5 mL) equipped with a stirring bar was charged with lignin model substrate (2.0 equiv, 0.1 mmol), CF_3-substituted alkene (if solid, 1.0 equiv, 0.05 mmol), $[Ir(dF(CF_3)ppy)_2(5,5'\text{-}d(CF_3)bpy)]PF_6$ (0.0005 mmol, 1 mmol%), ${}^{n}Bu_4NOAc$ (1.0 equiv, 0.05 mmol), and Na_2HPO_4 (1.0 equiv, 0.05 mmol). The vial was sealed, then evacuated and refilled with N_2 three times. Dry DMSO (0.05M, 1.0 mL) and CF_3-substituted alkene (if liquid, 1.0 equiv, 0.05 mmol) were added. The reaction mixture was irradiated with a 40 W Kessil® PR160-440nm lamp at 25 ºC with a distance of around 2 cm from the surface of the reaction vial. After 24 h of irradiation, water (1 mL) was added, and the resulting mixture was extracted with EtOAc (5×2 mL). The organic layer was washed with brine (3×8 mL) and dried over anhydrous $MgSO_4$, filtered, and concentrated under reduced pressure. The ratio of *gem*-difluoroalkenylation product and hydroalkylation by-product was determined by crude ^{19}F NMR analysis. The crude mixture was purified by chromatography on silica gel with hexane: ethyl acetate mixtures as eluent to give the corresponding products. (Note: in some cases, the similar polarity between difluoroalkenylation products and hydroalkylation by-products made the separation difficult and weak signals from by-products were observed in the ^{19}F NMR spectra of difluoroalkylation products.)

General procedure B (GP-B):

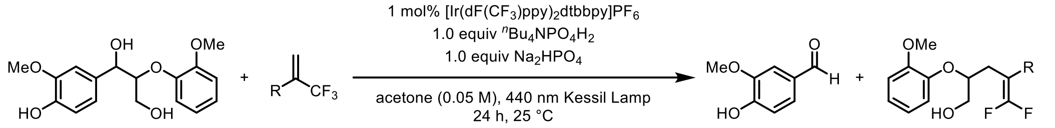

GP-B: A screw cap 2-dram vial (5 mL) equipped with a stirring bar was charged with phenolic lignin model **49** (2.0 equiv, 0.1 mmol), CF_3-substituted alkene (if solid, 1.0 equiv, 0.05 mmol), $[Ir(dF(CF_3)ppy)_2(dtbbpy)]PF_6$ (0.0005 mmol, 1 mmol%), ${}^{n}Bu_4NPO_4H_2$ (1.0 equiv, 0.05 mmol), and Na_2HPO_4 (1.0 equiv, 0.05 mmol). The vial was sealed, then evacuated and refilled with N_2 three times. Dry acetone (0.05M, 1.0 mL) and CF_3-substituted alkene (if liquid, 1.0 equiv, 0.05 mmol) were added. The reaction mixture was irradiated with a 40 W Kessil® PR160-440nm lamp at 25 ºC with a distance of around 2 cm from the surface of the reaction vial. After 24 h of irradiation, the resulting mixture was concentrated under reduced pressure. The ratio of *gem*-difluoroalkenylation product and hydroalkylation by-product was determined by crude ^{19}F NMR analysis. The crude mixture was purified by chromatography on silica gel with hexane: ethyl acetate mixtures as eluent to give the corresponding products.

General procedure C (GP-C):

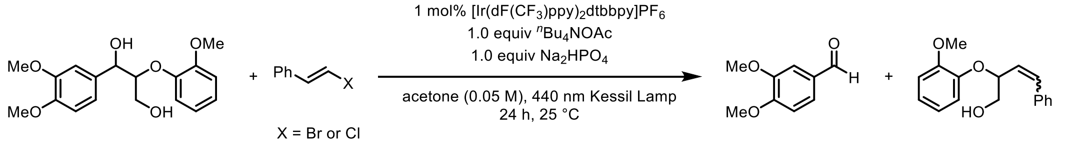

GP-C: A screw cap 2-dram vial (5 mL) equipped with a stirring bar was charged with phenolic lignin model **1** (1.0 equiv, 0.05 mmol), $[Ir(dF(CF_3)ppy)_2(dtbbpy)]PF_6$ (0.0005 mmol, 1 mmol%), ${}^{n}Bu_4NOAc$ (1.0 equiv, 0.05 mmol), and Na_2HPO_4 (1.0 equiv, 0.05 mmol). The vial was sealed, then evacuated and refilled with N_2 three times. Dry acetone (0.05M, 1.0 mL) and β-bromo/chloro-styrene (2.0 equiv, 1.0 mmol) were

added. The reaction mixture was irradiated with a 40 W Kessil® PR160-440nm lamp at 25 ºC with a distance of around 2 cm from the surface of the reaction vial. After 24 h of irradiation, the resulting mixture was concentrated under reduced pressure. The crude mixture was purified by chromatography on silica gel with hexane: ethyl acetate mixtures as eluent to give the corresponding products.

5,5-Difluoro-2-(2-methoxyphenoxy)-4-(4-methoxyphenyl)pent-4-en-1-ol (4)

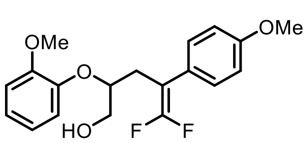

Prepared by **GP-A**. The ratio of *gem*-difluoroalkenylation product and hydroalkylation by-product is 37:1. Colorless oil, 14.7 mg, 84% yield. ^{1}H NMR (400 MHz, $CDCl_3$) δ 7.25 – 7.19 (m, 2H), 7.03 – 6.97 (m, 1H), 6.92 – 6.86 (m, 3H), 6.86 – 6.81 (m, 1H), 6.79 (dd, *J* = 8.0, 1.8 Hz, 1H), 4.08 (qd, *J* = 7.0, 3.0 Hz, 1H), 3.83 (s, 3H), 3.81 (s, 3H), 3.67 (dd, *J* = 12.1, 2.9 Hz, 1H), 3.56 (dd, *J* = 12.2, 5.1 Hz, 1H), 2.95 (ddt, *J* = 14.4, 6.8, 2.2 Hz, 1H), 2.72 (ddt, *J* = 14.7, 6.9, 2.3 Hz, 1H), 2.63 (br s, 1H); ^{13}C NMR (101 MHz, $CDCl_3$) δ 158.8, 154.1 (dd, *J* = 291.1, 289.1 Hz), 151.2, 147.0, 129.4 (t, *J* = 3.2 Hz), 125.1 (t, *J* =3.6 Hz), 123.45, 121.2, 119.8, 114.0, 112.1, 88.6 (dd, *J* = 21.4, 15.6 Hz), 81.0 (t, *J* = 2.9 Hz), 63.3, 55.7, 55.2, 29.8; ^{19}F NMR (377 MHz, $CDCl_3$) δ -90.14 (d, *J* = 41.5 Hz, 1F), -90.68 (d, *J* = 41.5 Hz, 1F). IR (film): ν (cm^{-1}) 3490, 3065, 3000, 2937, 2917, 2838, 1730, 1609, 1592, 1513, 1499, 1456, 1441, 1292, 1178, 1122, 1116, 1105, 1028, 981, 952, 906, 832, 798, 745, 696, 611, 565, 526, 456; HR-MS (ESI) m/z calcd for $C_{19}H_{20}F_2NaO_4^+$ [M+Na$^+$] 373.12219, found 373.12259.

5,5-Difluoro-2-(2-methoxyphenoxy)-4-phenylpent-4-en-1-ol (6)

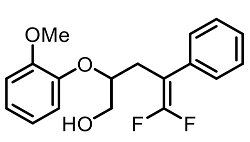

Prepared by **GP-A**. The ratio of *gem*-difluoroalkenylation product and hydroalkylation by-product is 12:1. Colorless oil, 13.6 mg, 85% yield. ^{1}H NMR (400 MHz, $CDCl_3$) δ 7.38 – 7.32 (m, 2H), 7.32 – 7.25 (m, 3H), 7.04 – 6.97 (m, 1H), 6.89 (dd, *J* = 8.2, 1.4 Hz, 1H), 6.83 (td, *J* = 7.6, 1.5 Hz, 1H), 6.77 (dd, *J* = 8.0, 1.7 Hz, 1H), 4.08 (qd, *J* = 7.0, 3.0 Hz, 1H), 3.82 (s, 3H), 3.67 (dd, *J* = 12.2, 2.9 Hz, 1H), 3.56 (dd, *J* = 12.2, 5.0 Hz, 1H), 3.01 (ddt, *J* = 14.8, 6.8, 2.4 Hz, 1H), 2.76 (ddt, *J* = 14.7, 6.8, 2.3 Hz, 1H), 2.61 (br s, 1H); ^{13}C NMR (101 MHz, $CDCl_3$) δ 154.3 (dd, *J* = 292.2, 289.6 Hz), 151.3, 147.0, 133.1 (t, *J* = 3.8 Hz), 128.5, 128.3 (t, *J* = 3.1 Hz), 127.5, 123.6, 121.3, 120.0, 112.1, 89.1 (dd, *J* = 21.3, 15.4 Hz), 81.0 (t, *J* = 2.9 Hz), 63.3, 55.8, 29.7 (d, *J* = 1.4 Hz); ^{19}F NMR (377 MHz, $CDCl_3$) δ -89.17 (d, *J* = 39.2 Hz, 1F), -89.77 (d, *J* = 38.8 Hz, 1F). IR (film): ν (cm^{-1}) 3479, 3063, 2938, 2838, 1730, 1592, 1498, 1456, 1446, 1305, 1249, 1224, 1177, 1123, 1073, 1042, 1026, 981, 954, 914, 807, 766, 742, 698, 611, 578; HR-MS (ESI) m/z calcd for $C_{18}H_{18}F_2NaO_3^+$ [M+Na$^+$] 343.11162, found 343.11171.

4-(4-(*tert*-Butyl)phenyl)-5,5-difluoro-2-(2-methoxyphenoxy)pent-4-en-1-ol (7)

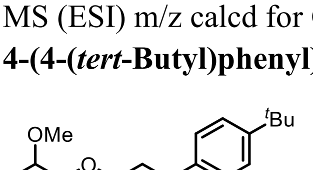

Prepared by **GP-A**. The ratio of *gem*-difluoroalkenylation product and hydroalkylation by-product is 27:1. Colorless oil, 16.5 mg, 88% yield. ^{1}H NMR (400 MHz, $CDCl_3$) δ 7.39 – 7.33 (m, 2H), 7.25 – 7.19 (m, 2H), 7.00 (ddd, *J* = 8.2, 7.4, 1.6 Hz, 1H), 6.88 (dd, *J* = 8.2, 1.4 Hz, 1H), 6.85 – 6.79 (m, 1H), 6.75 (dd, *J* = 8.0, 1.6 Hz, 1H), 4.10 (qd, *J* = 7.0, 3.0 Hz, 1H), 3.82 (s, 3H), 3.66 (dd, *J* = 12.2, 2.9 Hz, 1H), 3.57 (dd, *J* = 12.2 Hz, 5.1 Hz, 1H), 3.08 – 2.89 (m, 2H), 2.75 (ddt, *J* = 14.7, 6.9, 2.3 Hz, 1H),

1.33 (s, 9H); ^{13}C NMR (101 MHz, $CDCl_3$) δ 154.2 (dd, J = 292.0, 289.3 Hz), 151.3, 150.7, 147.0, 129.9 (t, J =3.8 Hz), 127.9 (t, J =3.2 Hz), 125.4, 123.6, 121.2, 120.1, 112.1, 89.8 (dd, J = 21.3, 15.2 Hz) , 81.1 (t, J = 2.8 Hz), 63.4, 55.8, 34.5, 31.2, 29.7; ^{19}F NMR (377 MHz, $CDCl_3$) δ -89.35 (d, J= 40.0 Hz, 1F), -89.92 (d, J = 39.6 Hz, 1F). IR (film): ν (cm^{-1}) 3479, 2960, 2926, 2868, 2838, 1591, 1499, 1457, 1440, 1405, 1364, 1306, 1251, 1178, 1113, 1042, 1026, 983, 956, 905, 836, 805, 742, 591, 580, 554, 542, 454; HR-MS (ESI) m/z calcd for $C_{22}H_{26}F_2NaO_3^+$ [M+Na$^+$] 399.17422, found 399.17428.

4-([1,1'-Biphenyl]-4-yl)-5,5-difluoro-2-(2-methoxyphenoxy)pent-4-en-1-ol (8)

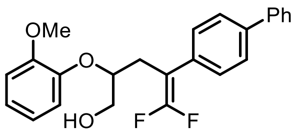

Prepared by **GP-A**. The ratio of *gem*-difluoroalkenylation product and hydroalkylation by-product is 5:1. Colorless oil, 14.8 mg, 75% yield. ^1H NMR (400 MHz, $CDCl_3$) δ 7.65 – 7.54 (m, 4H), 7.50 – 7.42 (m, 2H), 7.41 – 7.33 (m, 3H), 7.01 (ddd, J = 8.2, 6.8, 2.2 Hz, 1H), 6.89 (dd, J = 8.2, 1.2 Hz, 1H), 6.87 – 6.79 (m, 2H), 4.14 (qd, J = 7.0, 3.0 Hz, 1H), 3.83 (s, 3H), 3.71 (dd, J = 12.2, 2.9 Hz, 1H), 3.60 (dd, J = 12.2, 5.0 Hz, 1H), 3.05 (ddt, J = 14.8, 7.2, 2.4 Hz, 1H), 2.80 (ddt, J = 14.8, 6.8, 2.3 Hz, 1H); ^{13}C NMR (101 MHz, $CDCl_3$) δ 154.4 (dd, J = 292.7, 290.1 Hz), 151.3, 147.0, 140.4, 140.3, 132.0 (t, J = 3.9 Hz), 128.8, 128.6 (t, J = 3.2 Hz), 127.5, 127.2, 127.0, 123.6, 121.3, 120.0, 112.2, 88.9 (dd, J = 21.5, 15.0 Hz), 81.1 (t, J = 2.9 Hz), 63.4, 55.8, 29.7; ^{19}F NMR (377 MHz, $CDCl_3$) δ -89.52 (d, J = 38.1 Hz, 1F), -89.18 (d, J = 38.1 Hz, 1F). IR (film): ν (cm^{-1}) 3487, 3061, 3031, 2952, 2932, 2836, 1725, 1592, 1498, 1455, 1440, 1406, 1322, 1306, 1249, 1222, 1177, 1122, 1114, 1043, 1025, 1008, 982, 954, 912, 842, 763, 744, 735, 697, 583, 551, 507; HR-MS (ESI) m/z calcd for $C_{24}H_{22}F_2NaO_3^+$ [M+Na$^+$] 419.14292, found 419.14304.

5,5-Difluoro-2-(2-methoxyphenoxy)-4-(4-(methylthio)phenyl)pent-4-en-1-ol (9)

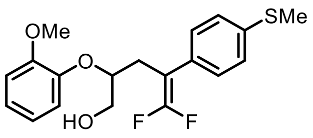

Prepared by **GP-A**. The ratio of *gem*-difluoroalkenylation product and hydroalkylation by-product is 5:1. Colorless oil, 12.2 mg, 67% yield. ^1H NMR (400 MHz, $CDCl_3$) δ 7.25 – 7.17 (m, 4H), 7.04 – 6.96 (m, 1H), 6.92 – 6.87 (m, 1H), 6.87 – 6.81 (m, 1H), 6.79 (dd, J = 7.9, 1.6 Hz, 1H), 4.11 – 4.03 (m, 1H), 3.82 (s, 3H), 3.66 (dd, J = 12.2, 2.9 Hz, 1H), 3.55 (dd, J = 12.2, 5.0 Hz, 1H), 2.98 (ddt, J = 14.8, 6.8, 2.0 Hz, 1H), 2.73 (ddt, J = 14.3, 6.7, 2.2 Hz, 1H), 2.49 (s, 3H); ^{13}C NMR (101 MHz, $CDCl_3$) δ 154.3 (dd, J = 292.4, 289.8 Hz), 151.3, 147.0, 137.9, 129.7 (dd, J = 3.9, 3.4 Hz), 128.6 (t, J = 3.3 Hz), 126.4, 123.6, 121.3, 120.0, 112.1, 88.7 (dd, J = 21.7, 15.2 Hz), 81.1 (t, J = 2.9 Hz), 63.3, 55.8, 29.6, 15.6; ^{19}F NMR (377 MHz, $CDCl_3$) δ -88.84 (d, J = 38.8 Hz, 1F), -89.46 (d, J = 38.8 Hz, 1F). IR (film): ν (cm^{-1}) 3475, 3063, 2922, 2836, 1727, 1592, 1498, 1455, 1439, 1399, 1313, 1302, 1249, 1178, 1121, 1115, 1092, 1041, 1027, 982, 954, 907, 882, 822, 745, 577, 511, 484; HR-MS (ESI) m/z calcd for $C_{19}H_{20}F_2NaO_3S^+$ [M+Na$^+$] 389.09934, found 389.09952.

5,5-Difluoro-2-(2-methoxyphenoxy)-4-(4-(trifluoromethoxy)phenyl)pent-4-en-1-ol (10)

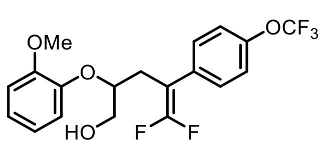

Prepared by **GP-A**. The ratio of *gem*-difluoroalkenylation product and hydroalkylation by-product is 7:1. Colorless oil, 16.2 mg, 80% yield. ^{1}H NMR (400 MHz, $CDCl_3$) δ 7.35 – 7.28 (m, 2H), 7.22 – 7.15 (m, 2H), 7.04 – 6.98 (m, 1H), 6.91 – 6.87 (m, 1H), 6.86 – 6.81 (m, 1H), 6.74 (dd, *J* = 7.9, 1.4 Hz, 1H), 4.12 – 4.04 (m, 1H), 3.81 (s, 3H), 3.68 (dd, *J* = 12.2, 3.0 Hz, 1H), 3.56 (dd, *J* = 12.2, 4.8 Hz, 1H), 2.99 (ddt, *J* = 14.8, 7.3, 2.0 Hz, 1H), 2.74 (ddt, *J* = 15.2, 6.4, 2.8 Hz, 1H), 2.62 (br s, 1H); ^{13}C NMR (101 MHz, $CDCl_3$) δ 154.4 (dd, *J* = 292.8, 290.3 Hz), 151.3, 148.3, 146.8, 131.9 (dd, *J* = 4.5, 3.2 Hz), 129.8 (t, *J* = 3.3 Hz), 123.8, 121.3, 121.0, 120.4 (q, *J* = 258.4 Hz), 120.0, 112.2, 88.4 (dd, *J* = 22.4, 15.0 Hz), 80.8 (t, *J* = 2.8 Hz), 63.2, 55.7, 29.8; ^{19}F NMR (377 MHz, $CDCl_3$) δ -57.85, -88.14 (d, *J* = 37.3 Hz, 1F), -88.98 (d, *J* = 37.3 Hz, 1F). IR (film): ν (cm^{-1}) 3483, 3068, 2936, 2875, 2841, 1730, 1592, 1500, 1457, 1440, 1412, 1305, 1249, 1220, 1160, 1121, 1115, 1103, 1043, 1027, 984, 956, 922, 851, 807, 743, 680, 580, 537, 511; HR-MS (ESI) m/z calcd for $C_{19}H_{17}F_5NaO_4^+$ [M+Na$^+$] 427.09392, found 427.09417.

5,5-Difluoro-2-(2-methoxyphenoxy)-4-(4-phenoxyphenyl)pent-4-en-1-ol (11)

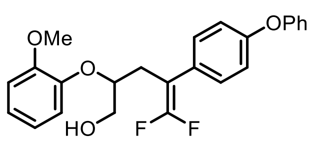

Prepared by **GP-A**. The ratio of *gem*-difluoroalkenylation product and hydroalkylation by-product is 23:1. Colorless oil, 18.7 mg, 90% yield. ^{1}H NMR (400 MHz, $CDCl_3$) δ 7.38 – 7.31 (m, 2H), 7.28 – 7.21 (m, 2H), 7.15 – 7.09 (m, 1H), 7.05 – 6.94 (m, 5H), 6.88 (dd, *J* = 8.2, 1.4 Hz, 1H), 6.86 – 6.81 (m, 1H), 6.79 (dd, *J* = 8.0, 1.7 Hz, 1H), 4.09 (qd, *J* = 7.0, 3.0 Hz, 1H), 3.83 (s, 3H), 3.68 (dd, *J* = 12.2, 3.0 Hz, 1H), 3.57 (dd, *J* = 12.2, 5.0 Hz, 1H), 2.98 (ddt, *J* = 14.8, 6.8, 2.4 Hz, 1H), 2.73 (ddt, *J* = 14.7, 6.7, 2.4 Hz, 1H); ^{13}C NMR (101 MHz, $CDCl_3$) δ 156.74, 156.66, 154.2 (dd, *J* = 291.2, 289.4 Hz), 151.3, 147.0, 129.8, 129.7 (t, *J* = 3.2 Hz), 127.7 (dd, *J* = 4.1, 3.7 Hz), 123.6, 123.6, 121.3, 120.0, 119.1, 118.6, 112.2, 88.6 (dd, *J* = 21.6, 15.4 Hz), 81.1 (t, *J* = 2.9 Hz), 63.4, 55.8, 29.8; ^{19}F NMR (377 MHz, $CDCl_3$) δ -89.37 (d, *J* = 40.0 Hz, 1F), -89.96 (d, *J* = 39.6 Hz, 1F). IR (film): ν (cm^{-1}) 3490, 3067, 3040, 2939, 2907, 2839, 2360, 1728, 1589, 1500, 1489, 1465, 1456, 1440, 1409, 1302, 1278, 1236, 1175, 1123, 1101, 1041, 1025, 982, 956, 906, 869, 840, 799, 743, 691, 572, 514, 493; HR-MS (ESI) m/z calcd for $C_{24}H_{22}F_2NaO_4^+$ [M+Na$^+$] 435.13784, found 435.13813.

4-(1,1-Difluoro-5-hydroxy-4-(2-methoxyphenoxy)pent-1-en-2-yl)phenyl acetate (12)

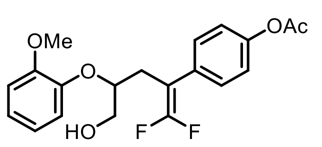

Prepared by **GP-A**. The ratio of *gem*-difluoroalkenylation product and hydroalkylation by-product is 20:1. Colorless oil, 11 mg, 58% yield. ^{1}H NMR (400 MHz, $CDCl_3$) δ 7.33 – 7.26 (m, 2H), 7.11 – 7.04 (m, 2H), 7.03 – 6.96 (m, 1H), 6.88 (dd, *J* = 8.2, 1.4 Hz, 1H), 6.86 – 6.80 (m, 1H), 6.76 (dd, *J* = 7.9, 1.6 Hz, 1H), 4.13 – 4.05 (m, 1H), 3.82 (s, 3H), 3.67 (dd, *J* = 12.2, 3.0 Hz, 1H), 3.55 (dd, *J* = 12.2, 4.8 Hz, 1H), 2.99 (ddt, *J* = 14.8, 6.8, 2.2 Hz, 1H), 2.74 (ddt, *J* = 14.8, 6.5, 2.4 Hz, 1H), 2.30 (s, 3H); ^{13}C NMR (101 MHz, $CDCl_3$) δ 169.3, 154.3 (dd, *J* = 292.5, 289.8 Hz), 151.3, 149.8, 146.9, 130.7 (dd, *J* = 4.2, 3.4 Hz), 129.3 (t, *J* = 3.3 Hz), 123.7, 121.7, 121.3, 120.2, 112.1, 88.6 (dd, *J* = 21.9, 15.2 Hz), 80.9 (t, *J* = 2.8 Hz), 63.2, 55.7, 29.8, 21.1; ^{19}F NMR (377 MHz, $CDCl_3$) δ -88.65 (d, *J* = 37.7 Hz, 1F), -89.28 (d, *J* = 38.1 Hz, 1F).

IR (film): ν (cm^{-1}) 3498, 3067, 2942, 2932, 2919, 2839, 1769, 1746, 1735, 1592, 1499, 1456, 1440, 1410, 1315, 1303, 1250, 1195, 1171, 1123, 1101, 1042, 1018, 983, 956, 910, 848, 802, 745, 665, 591, 576, 522; HR-MS (ESI) m/z calcd for $C_{20}H_{20}F_2NaO_5^+$ [M+Na$^+$] 401.11710, found 401.11709.

tert-Butyl (4-(1,1-difluoro-5-hydroxy-4-(2-methoxyphenoxy)pent-1-en-2-yl)phenyl)carbamate (13)

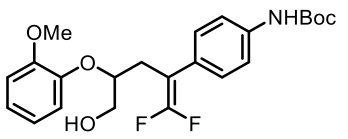

Prepared by **GP-A**. The ratio of *gem*-difluoroalkenylation product and hydroalkylation by-product is 30:1. Colorless oil, 17.1 mg, 78% yield. ^{1}H NMR (400 MHz, $CDCl_3$) δ 7.34 (d, *J* = 8.6 Hz, 2H), 7.21 (d, *J* = 7.9 Hz, 2H), 7.03 – 6.96 (m, 1H), 6.88 (dd, *J* = 8.1, 1.3 Hz, 1H), 6.86 – 6.80 (m, 1H), 6.79 (dd, *J* = 7.9, 1.8 Hz, 1H), 6.55 (s, 1H), 4.06 (qd, *J* = 6.9, 3.0 Hz, 1H), 3.82 (s, 3H), 3.65 (dd, *J* = 12.2, 2.9 Hz, 1H), 3.54 (dd, *J* = 12.2, 5.0 Hz, 1H), 2.97 (ddt, *J* = 14.6, 6.8, 2.2 Hz, 1H), 2.72 (ddt, *J* = 14.7, 6.8, 2.2 Hz, 1H), 2.62 (br s, 1H), 1.52 (s, 9H); ^{13}C NMR (101 MHz, $CDCl_3$) δ 154.2 (dd, *J* = 291.7, 289.3 Hz), 152.6, 151.3, 147.0, 137.6, 128.8 (t, *J* = 3.2 Hz), 127.4 (t, *J* = 3.7 Hz), 123.6, 121.3, 120.0, 118.4, 112.1, 88.6 (dd, *J* = 21.5, 15.4 Hz), 81.1 (t, *J* = 2.8 Hz), 80.7, 63.3, 55.8, 29.7, 28.3; ^{19}F NMR (377 MHz, $CDCl_3$) δ -89.47 (d, *J* = 40.0 Hz, 1F), -90.01 (d, *J* = 40.0 Hz, 1F). IR (film): ν (cm^{-1}) 3487, 3328, 2977, 2934, 2838, 1725, 1708, 1612, 1591, 1524, 1499, 1455, 1440, 1407, 1392, 1366, 1318, 1293, 1236, 1156, 1123, 1114, 1050, 1027, 982, 953, 904, 838, 766, 735, 701, 648, 580, 524, 458; HR-MS (ESI) m/z calcd for $C_{23}H_{27}F_2NNaO_5^+$ [M+Na$^+$] 458.17495, found 458.17519.

4-(4-Chlorophenyl)-5,5-difluoro-2-(2-methoxyphenoxy)pent-4-en-1-ol (14)

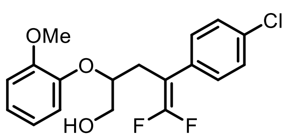

Prepared by **GP-A**. The ratio of *gem*-difluoroalkenylation product and hydroalkylation by-product is 6:1. Colorless oil, 13 mg, 73% yield. ^{1}H NMR (400 MHz, $CDCl_3$) δ 7.34 – 7.28 (m, 2H), 7.25 – 7.18 (m, 2H), 7.04 – 6.98 (m, 1H), 6.89 (dd, *J* = 8.2, 1.5 Hz, 1H), 6.87 – 6.81 (m, 1H), 6.77 (dd, *J* = 7.9, 1.7 Hz, 1H), 4.06 (qd, *J* = 7.0, 3.1 Hz, 1H), 3.82 (s, 3H), 3.67 (dd, *J* = 12.2, 3.0 Hz, 1H), 3.55 (dd, *J* = 12.2, 4.8 Hz, 1H), 2.97 (ddt, *J* = 14.8, 7.2, 2.2 Hz, 1H), 2.73 (ddt, *J* = 14.8, 6.4, 2.5 Hz, 1H), 2.39 (br s, 1H); ^{13}C NMR (101 MHz, $CDCl_3$) δ 154.3 (dd, *J* = 292.9, 290.2 Hz), 151.3, 146.9, 133.3, 131.6 (dd, *J* = 4.4, 3.5 Hz), 129.6 (t, *J* = 3.3 Hz), 128.7, 123.7, 121.3, 120.0, 112.2, 88.5 (dd, *J* = 22.1, 15.0 Hz), 81.0 (t, *J* = 2.8 Hz), 63.2, 55.8, 29.7; ^{19}F NMR (377 MHz, $CDCl_3$) δ -88.21 (d, *J* = 37.3 Hz, 1F), -88.93 (d, *J* = 37.3 Hz, 1F). IR (film): ν (cm^{-1}) 3479, 3066, 2934, 2919, 2838, 2363, 1728, 1593, 1496, 1456, 1439, 1400, 1315, 1300, 1248, 1221, 1178, 1122, 1093, 1042, 1027, 1015, 982, 954, 908, 831, 743, 580, 506, 488; HR-MS (ESI) m/z calcd for $C_{18}H_{17}ClF_2NaO_3^+$ [M+Na$^+$] 377.07265, found 377.07273.

5,5-Difluoro-2-(2-methoxyphenoxy)-4-(4-(trifluoromethyl)phenyl)pent-4-en-1-ol (15)

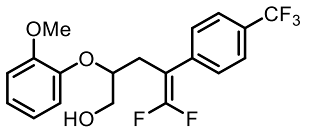

Prepared by **GP-A**. The ratio of *gem*-difluoroalkenylation product and hydroalkylation by-product is 2:1. Colorless oil, 8.8 mg, 45% yield. ^{1}H NMR (400 MHz, $CDCl_3$) δ 7.59 (d, *J* = 8.3 Hz, 2H), 7.41 (d, *J* = 8.2 Hz, 2H), 7.05 – 6.97 (m, 1H),

6.89 (dd, *J* = 8.2, 1.4 Hz, 1H), 6.86 – 6.81 (m, 1H), 6.76 (dd, *J* = 7.9, 1.6 Hz, 1H), 4.12 – 4.03 (m, 1H), 3.81 (s, 3H), 3.68 (dd, *J* = 12.2, 3.0 Hz, 1H), 3.55 (dd, *J* = 12.2, 4.7 Hz, 1H), 3.02 (ddt, *J* = 15.2, 7.6, 2.0 Hz, 1H), 2.79 (ddt, *J* = 14.9, 6.1, 2.5 Hz, 1H); ^{13}C NMR (101 MHz, $CDCl_3$) δ 154.6 (dd, *J* = 294.1, 291.1 Hz), 151.3, 146.8, 137.1 (t, *J* = 4.7 Hz), 129.5 (q, *J* = 32.9 Hz), 128.6 (t, *J* = 3.3 Hz), 125.4 (q, *J* = 3.7 Hz), 124.0 (q, *J* = 273.1 Hz), 123.8, 121.3, 120.0, 112.2, 88.8 (dd, *J* = 22.3, 14.4 Hz), 80.9 (t, *J* = 2.8 Hz), 63.2, 55.7, 29.6; ^{19}F NMR (377 MHz, $CDCl_3$) δ -62.69, -86.88 (d, *J* = 34.3 Hz, 1F), -87.94 (d, *J* = 34.3 Hz, 1F). IR (film): ν (cm^{-1}) 3479, 3071, 2943, 2839, 1726, 1618, 1592, 1500, 1456, 1441, 1410, 1325, 1250, 1222, 1166, 1120, 1043, 1027, 1018, 984, 956, 906, 844, 768, 743, 721, 600, 509, 448, 441; HR-MS (ESI) m/z calcd for $C_{19}H_{17}F_5NaO_3^+$ [M+Na$^+$] 411.09901, found 411.09926.

Methyl 3-(1,1-difluoro-5-hydroxy-4-(2-methoxyphenoxy)pent-1-en-2-yl)benzoate (16)

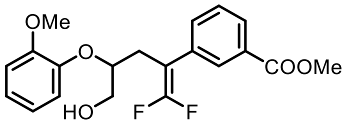

Prepared by **GP-A**. The ratio of *gem*-difluoroalkenylation product and hydroalkylation by-product is 6:1. Colorless oil, 15.5 mg, 82% yield. ^{1}H NMR (400 MHz, $CDCl_3$) δ 7.99 – 7.91 (m, 2H), 7.49 – 7.38 (m, 2H), 7.02 – 6.95 (m, 1H), 6.89 – 6.85 (m, 1H), 6.84 – 6.78 (m, 1H), 6.76 (dd, *J* = 7.9, 1.6 Hz, 1H), 4.10 – 4.03 (m, 1H), 3.91 (s, 3H), 3.80 (s, 3H), 3.67 (dd, *J* = 12.2, 2.9 Hz, 1H), 3.56 (dd, *J* = 12.2, 4.9 Hz, 1H), 3.03 (ddt, *J* = 14.8, 7.2, 2.0 Hz, 1H), 2.78 (ddt, *J* = 14.8, 6.4, 2.3 Hz, 1H), 2.64 (br s, 1H); ^{13}C NMR (101 MHz, $CDCl_3$) δ 166.6, 154.5 (dd, *J* = 292.9, 290.4 Hz), 151.3, 146.9, 133.6 (dd, *J* = 4.4, 3.2 Hz), 132.8 (t, *J* = 3.2 Hz), 130.6, 129.3 (t, *J* = 6.5 Hz), 128.7, 123.7, 121.3, 119.9, 112.2, 88.7 (dd, *J* = 22.1, 15.0 Hz), 80.8 (t, *J* = 2.8 Hz), 63.3, 55.7, 52.2, 29.7; ^{19}F NMR (377 MHz, $CDCl_3$) δ -88.12 (d, *J* = 36.9 Hz, 1F), -88.90 (d, *J* = 36.9 Hz, 1F). IR (film): ν (cm^{-1}) 3490, 3067, 2999, 2952, 2839, 1722, 1592, 1501, 1454, 1439, 1252, 1228, 1179, 1122, 1115, 1082, 1043, 1027, 997, 956, 910, 744, 698, 586; HR-MS (ESI) m/z calcd for $C_{20}H_{20}F_2NaO_5^+$ [M+Na$^+$] 401.11710, found 401.11721.

4-(4-Chloro-2-methoxyphenyl)-5,5-difluoro-2-(2-methoxyphenoxy)pent-4-en-1-ol (17)

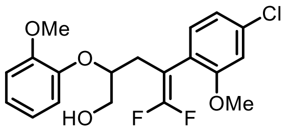

Prepared by **GP-A**. The ratio of *gem*-difluoroalkenylation product and hydroalkylation by-product is 15:1. Colorless oil, 14.5 mg, 75% yield. ^{1}H NMR (400 MHz, $CDCl_3$) δ 7.24 (dd, *J* = 8.8, 2.6 Hz, 1H), 7.04 – 6.95 (m, 2H), 6.88 (dd, *J* = 8.2, 1.5 Hz, 1H), 6.86 – 6.79 (m, 2H), 6.77 (dd, *J* = 8.0, 1.6 Hz, 1H), 4.01 (qd, *J* = 6.7, 3.3 Hz, 1H), 3.82 (s, 3H), 3.76 (s, 3H), 3.68 (dd, *J* = 12.1, 3.0 Hz, 1H), 3.59 (dd, *J* = 12.1, 5.3 Hz, 1H), 2.93 (ddt, *J* = 14.4, 6.4, 2.2 Hz, 1H), 2.71 (ddt, *J* = 14.8, 6.8, 2.0 Hz, 1H); ^{13}C NMR (101 MHz, $CDCl_3$) δ 155.9 (dd, *J* = 2.5, 0.6 Hz), 153.8 (t, *J* = 290.2 Hz), 151.2, 147.0, 130.9 (t, *J* = 2.3 Hz), 129.0, 125.4, 123.4 (t, *J* = 2.0 Hz), 123.4, 121.2, 119.4, 112.12, 112.10, 85.4 (dd, *J* = 24.1, 18.1 Hz), 80.6 (t, *J* = 2.9Hz), 63.6, 55.74, 55.69, 29.6; ^{19}F NMR (377 MHz, $CDCl_3$) δ -87.04 (d, *J* = 36.9 Hz, 1F), -90.59 (d, *J* = 36.6 Hz, 1F). IR (film): ν (cm^{-1}) 3490, 3068, 3003, 2940, 2837, 1740, 1592, 1492, 1456, 1442, 1401, 1280, 1250, 1223, 1178, 1120, 1025, 988, 967, 905, 887, 809, 743, 723,

643, 531, 472, 450; HR-MS (ESI) m/z calcd for $C_{19}H_{19}ClF_2NaO_4^+$ [M+Na$^+$] 407.08321, found 407.08344.

4-(5-Bromo-2-methoxyphenyl)-5,5-difluoro-2-(2-methoxyphenoxy)pent-4-en-1-ol (18)

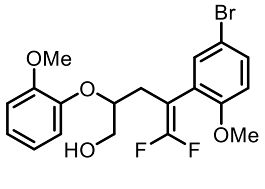

Prepared by **GP-A**. The ratio of *gem*-difluoroalkenylation product and hydroalkylation by-product is 15:1. Colorless oil, 15.5 mg, 72% yield. ^{1}H NMR (400 MHz, $CDCl_3$) δ 7.38 (dd, *J* = 8.8, 2.5 Hz, 1H), 7.14 (d, *J* = 2.4 Hz, 1H), 7.04 – 6.96 (m, 1H), 6.91 – 6.81 (m, 2H), 6.80 – 6.73 (m, 2H), 4.00 (qd, *J* = 6.7, 3.0 Hz, 1H), 3.82 (s, 3H), 3.75 (s, 3H), 3.68 (dd, *J* = 12.1, 3.0 Hz, 1H), 3.58 (dd, *J* = 12.1, 5.3 Hz, 1H), 2.92 (ddt, *J* = 14.8, 6.8, 2.0 Hz, 1H), 2.70 (ddt, *J* = 14.7, 6.7, 2.1 Hz, 1H), 2.56 (br s, 1H); ^{13}C NMR (101 MHz, $CDCl_3$) δ 156.4 (d, *J* = 2.5 Hz), 153.8 (t, *J* = 290.1 Hz), 151.1, 147.0, 133.7 (t, *J* = 2.4 Hz), 132.0, 123.9 (dd, *J* = 4.9, 2.0 Hz), 123.4, 121.2, 119.4, 112.6, 112.1, 85.4 (dd, *J* = 24.1, 18.2 Hz), 80.6 (t, *J* = 2.9 Hz), 63.6, 55.7, 55.6, 29.6; ^{19}F NMR (377 MHz, $CDCl_3$) δ -86.96 (d, *J* = 36.6 Hz, 1F), -90.55 (d, *J* = 36.6 Hz, 1F). IR (film): ν (cm^{-1}) 3487, 3067, 3002, 2967, 2943, 2909, 2837, 1741, 1592, 1499, 1490, 1456, 1441, 1395, 1303, 1280, 1250, 1232, 1223, 1178, 1120, 1041, 1025, 964, 809, 740, 623, 531, 449, 432; HR-MS (ESI) m/z calcd for $C_{19}H_{19}BrF_2NaO_4^+$ [M+Na$^+$] 451.03270, found 451.03309.

4-(3,5-Dimethoxyphenyl)-5,5-difluoro-2-(2-methoxyphenoxy)pent-4-en-1-ol (19)

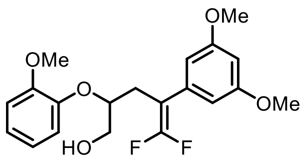

Prepared by **GP-A**. The ratio of *gem*-difluoroalkenylation product and hydroalkylation by-product is 16:1. Colorless oil, 16.2 mg, 85% yield. ^{1}H NMR (400 MHz, $CDCl_3$) δ 7.03 – 6.96 (m, 1H), 6.91 – 6.87 (m, 1H), 6.86 – 6.81 (m, 2H), 6.45 – 6.41 (m, 2H), 6.40 – 6.37 (m, 1H), 4.10 (qd, *J* = 7.0, 3.0 Hz, 1H), 3.83 (s, 3H), 3.75 (s, 6H), 3.68 (dd, *J* = 12.2, 2.9 Hz, 1H), 3.58 (dd, *J* = 12.2, 5.1 Hz, 1H), 2.97 (dddd, *J* = 14.7, 6.8, 2.5, 2.0 Hz, 1H), 2.71 (ddt, *J* = 14.7, 6.8, 2.3 Hz, 1H), 2.41 (br s, 1H); ^{13}C NMR (101 MHz, $CDCl_3$) δ 160.8, 154.3 (dd, *J* = 292.4, 289.3 Hz), 151.3, 147.0, 135.0 (dd, *J* = 4.3, 3.1 Hz), 123.5, 121.2, 119.8, 112.1, 106.5 (t, *J* = 3.2 Hz), 99.7, 89.2 (dd, *J* = 21.8, 15.2 Hz), 80.9 (t, *J* = 2.9 Hz), 63.4, 55.8, 55.3, 29.8; ^{19}F NMR (377 MHz, $CDCl_3$) δ -88.34 (d, *J* = 38.1 Hz, 1F), -88.78 (d, *J* = 38.1 Hz, 1F). IR (film): ν (cm^{-1}) 3490, 3003, 2958, 2941, 2840, 1730, 1591, 1499, 1455, 1423, 1353, 1252, 1204, 1178, 1154, 1121, 1060, 1042, 1027, 1003, 992, 927, 835, 818, 744, 702, 536, 469; HR-MS (ESI) m/z calcd for $C_{20}H_{22}F_2NaO_5^+$ [M+Na$^+$] 403.13275, found 403.13274.

4-(Benzo[d][1,3]dioxol-5-yl)-5,5-difluoro-2-(2-methoxyphenoxy)pent-4-en-1-ol (20)

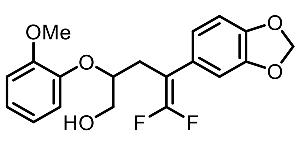

Prepared by **GP-A**. The ratio of *gem*-difluoroalkenylation product and hydroalkylation by-product is 30:1. Colorless oil, 13.4 mg, 73% yield. ^{1}H NMR (400 MHz, $CDCl_3$) δ 7.04 – 6.97 (m, 1H), 6.92 – 6.70 (m, 6H), 5.97 (s, 2H), 4.08 (qd, *J* = 7.0, 3.2 Hz, 1H), 3.84 (s, 3H), 3.67 (dd, *J* = 12.2, 2.9 Hz, 1H), 3.56 (dd, *J* = 12.2, 5.0

Hz, 1H), 2.94 (ddt, *J* = 14.7, 6.8, 2.2 Hz, 1H), 2.69 (ddt, *J* = 14.7, 6.7, 2.3 Hz, 1H); ^{13}C NMR (101 MHz, $CDCl_3$) δ 154.2 (dd, *J* = 291.1, 289.6 Hz), 151.3, 147.8, 147.0, 146.9, 126.7 (dd, *J* = 3.9, 2.7 Hz), 123.6, 121.9 (t, *J* = 3.1 Hz), 121.3, 120.0, 112.1, 108.8 (t, *J* = 3.3 Hz), 108.3, 101.2, 88.9 (dd, *J* = 21.6, 16.0 Hz), 81.0 (t, *J* = 2.9 Hz), 63.3, 55.8, 30.1; ^{19}F NMR (377 MHz, $CDCl_3$) δ -89.70 (d, *J* = 40.0 Hz, 1F), -89.92 (d, *J* = 40.3 Hz, 1F). IR (film): ν (cm^{-1}) 3490, 3068, 2913, 2839, 2358, 1729, 1592, 1499, 1493, 1455, 1440, 1341, 1305, 1240, 1222, 1179, 1116, 1034, 987, 971, 931, 863, 811, 743, 600, 559, 470; HR-MS (ESI) m/z calcd for $C_{19}H_{18}F_2NaO_5^+$ [M+Na$^+$] 387.10145, found 387.10157.

5,5-Difluoro-2-(2-methoxyphenoxy)-4-(3,4,5-trimethoxyphenyl)pent-4-en-1-ol (21)

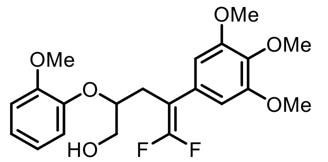

Prepared by **GP-A**. The ratio of *gem*-difluoroalkenylation product and hydroalkylation by-product is 21:1. Colorless oil, 17.4 mg, 85% yield. ^{1}H NMR (400 MHz, $CDCl_3$) δ 7.04 – 6.95 (m, 1H), 6.89 (d, *J* = 8.0 Hz, 1H), 6.83 (d, *J* = 4.0 Hz, 2H), 6.47 (s, 2H), 4.11 (qd, *J* = 6.8, 3.1 Hz, 1H), 3.85 (s, 3H), 3.82 (s, 3H), 3.77 (s, 6H), 3.70 (dd, *J* = 12.1, 3.0 Hz, 1H), 3.60 (dd, *J* = 12.1, 5.2 Hz, 1H), 2.95 (ddt, *J* = 14.7, 6.7, 2.3 Hz, 1H), 2.70 (ddt, *J* = 14.7, 6.8, 2.3 Hz, 1H), 2.60 (br s, 1H); ^{13}C NMR (101 MHz, $CDCl_3$) δ 154.2 (dd, *J* = 291.7, 289.6 Hz), 153.1, 151.2, 147.0, 137.4, 128.4 (dd, *J* = 4.1, 2.9 Hz), 123.5, 121.2, 119.6, 112.1, 105.6 (t, *J* = 3.2 Hz), 89.2 (dd, *J* = 21.2, 15.0 Hz), 80.8 (t, *J* = 2.8 Hz), 63.5, 60.8, 56.0, 55.7, 29.9; ^{19}F NMR (377 MHz, $CDCl_3$) δ -88.97 (d, *J* = 39.6 Hz, 1F), -89.27 (d, *J* = 39.6 Hz, 1F). IR (film): ν (cm^{-1}) 3502, 3001, 2936, 2838, 1732, 1584, 1501, 1455, 1414, 1352, 1268, 1252, 1220, 1176, 1118, 1042, 1026, 1004, 910, 830, 768, 744, 688, 599, 530, 446; HR-MS (ESI) m/z calcd for $C_{21}H_{24}F_2NaO_6^+$ [M+Na$^+$] 433.14322, found 433.14318.

4-(9H-Fluoren-2-yl)-5,5-difluoro-2-(2-methoxyphenoxy)pent-4-en-1-ol (22)

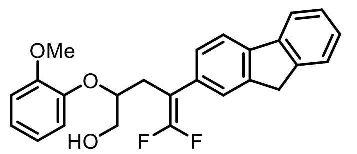

Prepared by **GP-A**. The ratio of *gem*-difluoroalkenylation product and hydroalkylation by-product is 8:1. Colorless oil, 14.9 mg, 73% yield. ^{1}H NMR (400 MHz, $CDCl_3$) δ 7.78 (d, *J* = 7.5 Hz, 1H), 7.75 (d, *J* = 8.0 Hz, 1H), 7.55 (d, *J* = 7.3 Hz, 1H), 7.45 – 7.36 (m, 2H), 7.35 – 7.28 (m, 2H), 7.03 – 6.97 (m, 1H), 6.90 – 6.86 (m, 1H), 6.84 – 6.77 (m, 2H), 4.13 (qd, *J* = 6.8, 3.0 Hz, 1H), 3.88 (s, 2H), 3.81 (s, 3H), 3.70 (dd, *J* = 12.2, 2.9 Hz, 1H), 3.61 (dd, *J* = 12.2, 5.1 Hz, 1H), 3.05 (ddt, *J* = 14.8, 6.8, 2.4 Hz, 1H), 2.81 (ddt, *J* = 14.7, 6.8, 2.3 Hz, 1H), 2.33 (br s, 1H); ^{13}C NMR (101 MHz, $CDCl_3$) δ 154.3 (dd, *J* = 291.6, 289.7 Hz), 151.3, 147.0, 143.5, 143.3, 141.1, 131.4 (t, *J* = 3.5 Hz), 126.94 (t, *J* = 3.3 Hz), 126.92, 126.8, 125.0 (t, *J* = 3.1 Hz), 124.9, 123.5, 121.2, 120.0, 119.9, 119.8, 112.1, 89.4 (dd, *J* = 21.2, 15.4 Hz), 81.0 (t, *J* = 2.9 Hz), 63.4, 55.8, 36.9, 30.0; ^{19}F NMR (377 MHz, $CDCl_3$) δ -89.22 (d, *J* = 39.6 Hz, 1F), -89.64 (d, *J* = 39.6 Hz, 1F). IR (film): ν (cm^{-1}) 3490, 3057, 2923, 2837, 2360, 1726, 1592, 1499, 1455, 1439, 1402, 1298, 1247, 1222, 1178, 1114, 1043, 1025, 991, 974, 908, 831, 770, 736, 593, 470, 419; HR-MS (ESI) m/z calcd for $C_{25}H_{22}F_2NaO_3^+$ [M+Na$^+$] 431.14292, found 431.14317.

5,5-Difluoro-2-(2-methoxyphenoxy)-4-(naphthalen-2-yl)pent-4-en-1-ol (23)

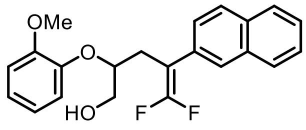

Prepared by **GP-A**. The ratio of *gem*-difluoroalkenylation product and hydroalkylation by-product is 5:1. Colorless oil, 12.7 mg, 69% yield. ^{1}H NMR (400 MHz, $CDCl_3$) δ 7.86 – 7.80 (m, 2H), 7.79 – 7.74 (m, 1H), 7.71 (s, 1H), 7.52 – 7.46 (m, 2H), 7.42 (dt, *J* = 8.6, 1.7 Hz, 1H), 7.03 – 6.96 (m, 1H), 6.90 – 6.85 (m, 1H), 6.83 – 6.76 (m, 2H), 4.13 (qd, *J* = 6.9, 3.0 Hz, 1H), 3.78 (s, 3H), 3.70 (dd, *J* = 12.2, 3.0 Hz, 1H), 3.61 (dd, *J* = 12.2, 5.1 Hz, 1H), 3.11 (ddt, *J* = 14.8, 6.8, 2.4 Hz, 1H), 2.86 (ddt, *J* = 14.7, 6.8, 2.3 Hz, 1H), 2.61 (br s, 1H); ^{13}C NMR (101 MHz, $CDCl_3$) δ 154.5 (dd, *J* = 292.6, 290.0 Hz), 151.3, 147.0, 133.2, 132.5, 130.4 (t, *J* = 3.8 Hz), 128.2, 127.9, 127.6, 127.4 (t, *J* = 3.2 Hz), 126.34, 126.26, 126.0 (t, *J* = 3.1 Hz), 123.6, 121.2, 119.9, 112.1, 89.2 (dd, *J* = 21.6, 15.2 Hz), 81.0 (t, *J* = 2.9 Hz), 63.4, 55.7, 29.8; ^{19}F NMR (377 MHz, $CDCl_3$) δ -88.56 (d, *J* = 37.7 Hz, 1F), -89.32 (d, *J* = 38.1 Hz, 1F). IR (film): ν (cm^{-1}) 3471, 3061, 2924, 2910, 2835, 2362, 1719, 1592, 1500, 1467, 1453, 1439, 1360, 1310, 1292, 1249, 1226, 1182, 1122, 1048, 1026, 999, 949, 938, 907, 894, 864, 847, 826, 776, 743, 642, 618, 551, 477; HR-MS (ESI) m/z calcd for $C_{22}H_{20}F_2NaO_3^+$ [M+Na$^+$] 393.12727, found 393. 12705.

4-(9,9'-Spirobi[fluoren]-2-yl)-5,5-difluoro-2-(2-methoxyphenoxy)pent-4-en-1-ol (24)

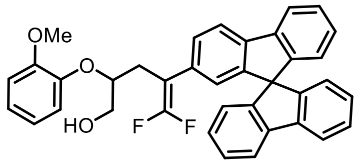

Prepared by **GP-A**. The ratio of *gem*-difluoroalkenylation product and hydroalkylation by-product is 5:1. Colorless oil, 19.1 mg, 68% yield. ^{1}H NMR (400 MHz, $CDCl_3$) δ 7.89 – 7.80(m, 4H), 7.42 – 7.31 (m, 4H), 7.15 – 7.07 (m, 3H), 6.97 – 6.90 (m, 1H), 6.82 (dd, *J* = 8.2, 1.4 Hz, 1H), 6.77 – 6.64 (m, 5H), 6.48 (dd, *J* = 8.0, 1.5 Hz, 1H), 3.88 (qd, *J* = 6.9, 3.1 Hz, 1H), 3.77 (s, 3H), 3.51 (dd, *J* = 12.2, 2.9 Hz, 1H), 3.39 (dd, *J* = 12.2, 4.9 Hz, 1H), 2.84 (ddt, *J* = 14.7, 6.8, 2.1 Hz, 1H), 2.59 (ddt, *J* = 14.7, 6.8, 2.3 Hz, 1H); ^{13}C NMR (101 MHz, $CDCl_3$) δ 154.2 (dd, *J* = 291.7, 290.7 Hz), 151.2, 149.2, 149.1, 148.5, 148.4, 146.9, 141.8, 141.7, 141.3, 141.0, 132.5 (t, *J* = 1.7 Hz), 128.2 (t, *J* = 3.3 Hz), 128.0, 127.88, 127.86, 127.82, 127.80, 127.77, 124.0, 123.94, 123.91, 123.5 (t, *J* = 3.1 Hz), 123.4, 121.3, 120.1 (t, *J* = 2.5 Hz), 119.8, 112.0, 89.1 (dd, *J* = 20.1, 16.7 Hz), 81.0 (t, *J* = 2.8 Hz), 65.9, 63.0, 55.8, 29.5; ^{19}F NMR (377 MHz, $CDCl_3$) δ -88.97 (d, *J* = 38.1 Hz, 1F), -89.09 (d, *J* = 38.1 Hz, 1F). IR (film): ν (cm^{-1}) 3494, 3062, 2929, 2839, 1725, 1592, 1498, 1461, 1447, 1416, 1251, 1220, 1177, 1116, 1043, 1027, 947, 908, 887, 834, 751, 729, 636, 620, 594; HR-MS (ESI) m/z calcd for $C_{37}H_{28}F_2NaO_3^+$ [M+Na$^+$] 581.18987, found 581.19019.

4-(Benzo[b]thiophen-3-yl)-5,5-difluoro-2-(2-methoxyphenoxy)pent-4-en-1-ol (25)

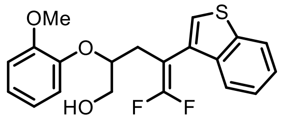

Prepared by **GP-A**. The ratio of *gem*-difluoroalkenylation product and hydroalkylation by-product is 100:1. Colorless oil, 13.7 mg, 73% yield. ^{1}H NMR (400 MHz, $CDCl_3$) δ 7.91 – 7.84 (m, 1H), 7.68 – 7.61 (m, 1H), 7.42 – 7.34 (m, 2H), 7.25 (s, 1H), 7.02 – 6.94 (m, 1H), 6.87 (dd, *J* = 8.2, 1.4 Hz, 1H), 6.81 – 6.74 (m, 1H), 6.70 (dd, *J* = 8.0, 1.6 Hz, 1H), 4.10 – 4.02 (m, 1H), 3.79 (s, 3H), 3.67 (dd, *J* = 12.2, 3.0 Hz, 1H), 3.56 (dd, *J* = 12.2, 4.8 Hz, 1H), 3.08 (dddd, *J* = 14.6, 7.2, 2.4, 1.4 Hz, 1H), 2.79 (ddt, *J* = 14.6, 6.2, 2.4 Hz, 1H), 2.57 (br s, 1H); ^{13}C NMR (101 MHz, $CDCl_3$) δ 154.4 (t,*J* =

291.4 Hz), 151.2, 146.9, 139.9, 137.6 (dd, J = 2.0, 1.2 Hz), 128.4 (dd, J = 4.7, 1.9 Hz), 125.7 (dd, J = 3.9, 1.3 Hz), 124.6, 124.4, 123.5, 122.8, 122.5 (d, J = 2.1 Hz), 121.2, 119.7, 112.1, 83.7 (dd, J = 24.0, 18.4 Hz), 80.7 (t, J = 2.8 Hz), 63.3, 55.7, 30.7 (d, J = 1.5 Hz); ^{19}F NMR (377 MHz, $CDCl_3$) δ -84.74 (d, J = 35.4 Hz, 1F), -88.91 (d, J = 35.4 Hz, 1F). IR (film): ν (cm^{-1}) 3487, 3066, 3003, 2927, 2836, 1733, 1591, 1498, 1456, 1439, 1428, 1354, 1274, 1250, 1218, 1177, 1116, 1043, 1023, 959, 928, 822, 802, 762, 734, 710, 628, 534, 493, 424; HR-MS (ESI) m/z calcd for $C_{20}H_{18}F_2NaO_3S^+$ [M+Na$^+$] 399.08369, found 399.08390.

***tert*-Butyl 3-(1,1-difluoro-5-hydroxy-4-(2-methoxyphenoxy)pent-1-en-2-yl)-1H-indole-1-carboxylate (26)**

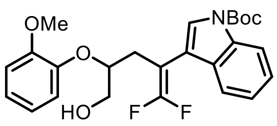

Prepared by **GP-A**. The ratio of *gem*-difluoroalkenylation product and hydroalkylation by-product is 100:1. Colorless oil, 17.8 mg, 77% yield. ^{1}H NMR (400 MHz, $CDCl_3$) δ 8.17 (d, J = 8.1 Hz, 1H), 7.49 (s, 1H), 7.47 – 7.42 (m, 1H), 7.38 – 7.31 (m, 1H), 7.26 – 7.20 (m, 1H), 7.01 – 6.95 (m, 1H), 6.89 – 6.84 (m, 1H), 6.82 – 6.75 (m, 2H), 4.17 – 4.09 (m, 1H), 3.79 (s, 3H), 3.69 (dd, J = 12.2, 3.0 Hz, 1H), 3.59 (dd, J = 12.2, 5.1 Hz, 1H), 3.05 (dddd, J = 14.5, 6.8, 2.4, 1.8 Hz, 1H), 2.78 (ddt, J = 14.5, 6.7, 2.2 Hz, 1H), 2.66 (br s, 1H), 1.68 (s, 9H); ^{13}C NMR (101 MHz, $CDCl_3$) δ 154.3 (t, J = 291.0 Hz), 151.2, 149.4, 146.9, 135.3, 128.8, 124.7 (dd, J = 4.3, 1.8 Hz), 124.7, 123.5, 122.8, 121.2, 119.9 (d, J = 3.3 Hz), 119.8, 115.3, 113.0 (dd, J = 4.8, 2.3 Hz), 112.08, 84.0, 81.6 (dd, J = 25.1, 17.9 Hz), 80.9 (t, J = 2.9 Hz), 63.4, 55.7, 30.2, 28.1; ^{19}F NMR (377 MHz, $CDCl_3$) δ -85.31 (d, J = 36.2 Hz, 1F), -89.03 (d, J = 36.2 Hz, 1F). IR (film): ν (cm^{-1}) 3494, 3058, 2977, 2933, 2839, 1732, 1592, 1500, 1452, 1374, 1336, 1309, 1286, 1251, 1223, 1176, 1153, 1114, 1083, 1066, 1048, 1023, 926, 851, 765, 742, 587, 475, 462, 425; HR-MS (ESI) m/z calcd for $C_{25}H_{27}F_2NaO_5^+$ [M+Na$^+$] 482.17495, found 482.17550.

4-(Dibenzo[b,d]furan-2-yl)-5,5-difluoro-2-(2-methoxyphenoxy)pent-4-en-1-ol (27)

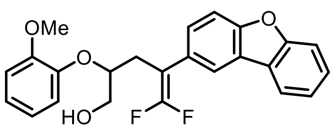

Prepared by **GP-A**. The ratio of *gem*-difluoroalkenylation product and hydroalkylation by-product is 38:1. Colorless oil, 19.1 mg, 93% yield. ^{1}H NMR (400 MHz, $CDCl_3$) δ 7.94 – 7.87 (m, 1H), 7.81 – 7.76 (m, 1H), 7.61 – 7.51 (m, 2H), 7.51 – 7.44 (m, 1H), 7.39 – 7.32 (m, 2H), 7.03 – 6.96 (m, 1H), 6.87 (d, J = 7.9 Hz, 1H), 6.82 – 6.77 (m, 2H), 4.14 (qd, J = 6.9, 3.1 Hz, 1H), 3.79 (s, 3H), 3.71 (dd, J = 12.2, 2.9 Hz, 1H), 3.63 (dd, J = 12.2, 5.1 Hz, 1H), 3.14 – 3.04 (m, 1H), 2.88 – 2.79 (m, 1H), 2.62 (br s, 1H); ^{13}C NMR (101 MHz, $CDCl_3$) δ 156.5, 155.3, 154.3 (dd, J = 290.7, 289.8 Hz), 151.2, 147.0, 127.7 (dd, J = 4.2, 2.9 Hz), 127.45, 127.42 (t, J = 3.0 Hz), 124.5, 123.8, 123.5, 122.8, 121.2, 120.71, 120.69 (t, J = 3.0 Hz), 119.8, 112.1, 111.73, 111.70, 89.1 (dd, J = 21.7, 16.0 Hz), 80.8 (t, J = 2.9 Hz), 63.4, 55.7, 30.4; ^{19}F NMR (377 MHz, $CDCl_3$) δ -89.72 (d, J = 40.3 Hz, 1F), -90.28 (d, J = 40.3 Hz, 1F). IR (film): ν (cm^{-1}) 3479, 3066, 2926, 2836, 1731, 1591, 1499, 1480, 1451, 1341, 1320, 1300, 1289, 1248, 1230, 1198, 1179, 1116, 1042, 1023, 984, 960, 907, 892, 842, 818, 775, 767, 745, 735, 606, 566, 460; HR-MS (ESI) m/z calcd for $C_{24}H_{20}F_2NaO_4^+$ [M+Na$^+$] 433.12219, found 433.12258.

4-(Dibenzo[b,d]thiophen-2-yl)-5,5-difluoro-2-(2-methoxyphenoxy)pent-4-en-1-ol (28)

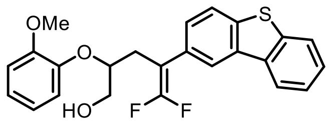

Prepared by **GP-A**. The ratio of *gem*-difluoroalkenylation product and hydroalkylation by-product is 30:1. Colorless oil, 19.6 mg, 92% yield. ^{1}H NMR (400 MHz, $CDCl_3$) δ 8.11 – 8.04 (m, 1H), 8.01 (s, 1H), 7.89 – 7.84 (m, 1H), 7.82 (d, *J* = 8.3 Hz, 1H), 7.50 – 7.43 (m, 2H), 7.41 – 7.36 (m, 1H), 6.99 (ddd, *J* = 8.2, 6.6, 2.4 Hz, 1H), 6.89 – 6.85 (m, 1H), 6.84 – 6.77 (m, 2H), 4.16 (qd, *J* = 6.9, 3.1 Hz, 1H), 3.78 (s, 3H), 3.72 (dd, *J* = 12.1, 3.0 Hz, 1H), 3.63 (dd, *J* = 12.2, 5.1 Hz, 1H), 3.11 (dddd, *J* = 14.7, 6.9, 2.5, 1.9 Hz, 1H), 2.86 (ddt, *J* = 14.8, 6.6, 2.4 Hz, 1H), 2.66 (s, 1H); ^{13}C NMR (101 MHz, $CDCl_3$) δ 154.4 (dd, *J* = 291.8, 290.0 Hz), 151.2, 146.9, 139.8, 138.6, 135.8, 135.1, 129.4 (dd, *J* = 4.2, 3.2 Hz), 127.0, 126.8 (t, *J* = 3.1 Hz), 124.4, 123.5, 122.8, 121.7, 121.4 (t, *J* = 3.1 Hz), 121.2, 119.7, 112.1, 89.1 (dd, *J* = 21.7, 15.4 Hz), 80.7 (t, *J* = 2.8 Hz), 63.4, 55.7, 30.1; ^{19}F NMR (377 MHz, $CDCl_3$) δ -88.93 (d, *J* = 39.2 Hz, 1F), -89.56 (d, *J* = 39.2 Hz, 1F). IR (film): ν (cm^{-1}) 3479, 3064, 2931, 2838, 2361, 2340, 1728, 1591, 1498, 1466, 1456, 1433, 1414, 1288, 1251, 1238, 1220, 1177, 1121, 1079, 1042, 1025, 985, 962, 907, 886, 809, 763, 732, 628, 514, 418; HR-MS (ESI) m/z calcd for $C_{24}H_{20}F_2NaO_3S^+$ [M+Na$^+$] 449.09934, found 449.09970.

5,5-Difluoro-2-(2-methoxyphenoxy)-4-(6-methoxypyridin-3-yl)pent-4-en-1-ol (29)

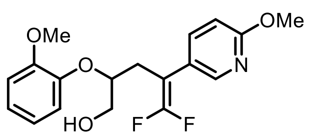

Prepared by **GP-A**. The ratio of *gem*-difluoroalkenylation product and hydroalkylation by-product is 37:1. Colorless oil, 14.7 mg, 84% yield. ^{1}H NMR (400 MHz, $CDCl_3$) δ 8.13 – 8.08 (m, 1H), 7.52 – 7.45 (m, 1H), 7.04 – 6.97 (m, 1H), 6.90 – 6.78 (m, 3H), 6.72 (d, *J* = 8.6 Hz, 1H), 4.13 – 4.05 (m, 1H), 3.94 (s, 3H), 3.82 (s, 3H), 3.68 (dd, *J* = 12.2, 3.0 Hz, 1H), 3.55 (dd, *J* = 12.2, 4.7 Hz, 1H), 2.95 (ddt, *J* = 14.8, 7.2, 2.2 Hz, 1H), 2.84 (br s, 1H), 2.72 (ddt, *J* = 14.8, 6.0, 2.4 Hz, 1H); ^{13}C NMR (101 MHz, $CDCl_3$) δ 163.2, 154.3 (dd, *J* = 291.9, 290.4 Hz), 151.3, 146.8, 146.3 (t, *J* = 3.6 Hz), 138.5 (t, *J* = 3.1 Hz), 123.8, 122.1 (t, *J* = 4.0 Hz), 121.3, 120.0, 112.2, 110.7, 86.3 (dd, *J* = 22.9, 15.7 Hz), 81.0 (t, *J* = 2.8 Hz), 63.2, 55.7, 53.6, 29.7; ^{19}F NMR (377 MHz, $CDCl_3$) δ -88.42 (d, *J* = 38.8 Hz, 1F), -89.50 (d, *J* = 38.8 Hz, 1F). IR (film): ν (cm^{-1}) 3471, 2947, 2839, 1731, 1603, 1563, 1496, 1457, 1440, 1378, 1313, 1248, 1178, 1044, 981, 953, 926, 909, 832, 645, 592, 581, 534, 470; HR-MS (ESI) m/z calcd for $C_{18}H_{19}F_2NNaO_4^+$ [M+Na$^+$] 374.11744, found 374.11761.

5,5-Difluoro-2-(2-methoxyphenoxy)-4-(2-methoxypyrimidin-5-yl)pent-4-en-1-ol (30)

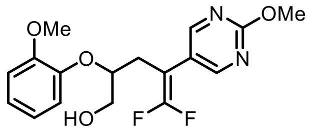

Prepared by **GP-A**. The ratio of *gem*-difluoroalkenylation product and hydroalkylation by-product is 12:1. Colorless oil, 14.9 mg, 85% yield. ^{1}H NMR (400 MHz, $CDCl_3$) δ 8.44 (s, 2H), 7.04 – 6.97 (m, 1H), 6.90 – 6.79 (m, 3H), 4.18 – 4.10 (m, 1H), 4.00 (s, 3H), 3.80 (s, 3H), 3.71 (dd, *J* = 12.2, 3.2 Hz, 1H), 3.56 (dd, *J* = 12.2, 4.3 Hz, 1H), 2.94 (ddt, *J* = 14.9, 8.0, 2.1 Hz, 1H), 2.85 (br s, 1H), 2.73 (ddt, *J* = 15.0, 5.2, 2.5 Hz, 1H); ^{13}C NMR (101 MHz, $CDCl_3$) δ 164.6, 158.5 (t, *J* = 3.6 Hz), 154.4 (dd, *J* = 293.2, 291.8 Hz), 151.2, 146.5, 123.9, 121.3, 121.2 (t, *J* = 4.3 Hz), 119.8, 112.2, 84.1 (dd, *J* = 24.5, 15.6 Hz), 80.6 (t, *J* = 2.7 Hz), 63.0, 55.7, 55.0, 29.4 (d, *J* = 1.3 Hz);

^{19}F NMR (377 MHz, $CDCl_3$) δ -86.58 (d, *J* = 35.8 Hz, 1F), -87.96 (d, *J* = 35.8 Hz, 1F). IR (film): ν (cm^{-1}) 3377, 3001, 2957, 2935, 2870, 2840, 1731, 1595, 1550, 1500, 1474, 1414, 1391, 1330, 1249, 1220, 1179, 1121, 1026, 980, 946, 907, 803, 744, 642, 594, 523, 418; HR-MS (ESI) m/z calcd for $C_{17}H_{18}F_2NaO_4^+$ [M+Na^+] 375.11268, found 375.11301.

4-(Difluoromethylene)-2-(2-methoxyphenoxy)-6-phenylhexan-1-ol (31)

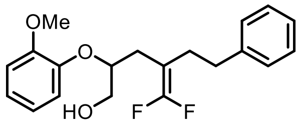

Prepared by **GP-A**. The ratio of *gem*-difluoroalkenylation product and hydroalkylation by-product is 15:1. Colorless oil, 15.0 mg, 43% yield. ^{1}H NMR (400 MHz, $CDCl_3$) δ 7.30 – 7.25 (m, 2H), 7.22 – 7.12 (m, 3H), 7.07 – 7.01 (m, 1H), 7.00 – 6.96 (m, 1H), 6.95 – 6.89 (m, 2H), 4.23 – 4.15 (m, 1H), 3.86 (s, 3H), 3.70 (dd, *J* = 12.1, 3.0 Hz, 1H), 3.60 (dd, *J* = 12.1, 5.4 Hz, 1H), 2.75 – 2.67 (m, 2H), 2.51 (ddt, *J* = 14.5, 6.7, 2.4 Hz, 1H), 2.40 – 2.27 (m, 3H); ^{13}C NMR (101 MHz, $CDCl_3$) δ 154.3 (t, *J* = 286.6 Hz), 151.2, 147.1, 141.0, 128.4, 128.3, 126.1, 123.6, 121.4, 119.8, 112.2, 85.5 (dd, *J* = 18.3, 17.0 Hz), 81.8 (t, *J* = 3.2 Hz), 63.6, 55.8, 33.9 (t, *J* = 2.8 Hz), 28.8 (d, *J* = 1.8 Hz), 28.2 (t, *J* = 2.2 Hz); ^{19}F NMR (377 MHz, $CDCl_3$) δ -92.71 (d, *J* = 50.5 Hz, 1F), -92.89 (d, *J* = 50.9 Hz, 1F). IR (film): ν (cm^{-1}) 3487, 3027, 2955, 2929, 2856, 2838, 1746, 1682, 1592, 1498, 1455, 1440, 1252, 1219, 1208, 1178, 1153, 1121, 1116, 1044, 1024, 908, 809, 742, 699, 583, 526, 498; HR-MS (ESI) m/z calcd for $C_{20}H_{22}F_2NaO_3^+$ [M+Na^+] 371.14292, found 371.14311.

4-(Difluoromethylene)-2-(2-methoxyphenoxy)-8-phenyloct-5-yn-1-ol (32)

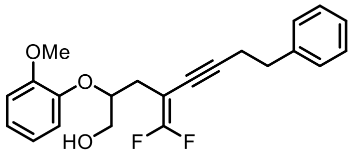

Prepared by **GP-A**. The ratio of *gem*-difluoroalkenylation product and hydroalkylation by-product is 23:1. Colorless oil, 10.0 mg, 54% yield. ^{1}H NMR (400 MHz, $CDCl_3$) δ 7.30 – 7.24 (m, 2H), 7.23 – 7.16 (m, 3H), 7.06 – 6.99 (m, 2H), 6.94 – 6.87 (m, 2H), 4.24 – 4.15 (m, 1H), 3.86 (s, 3H), 3.72 (dd, *J* = 12.2, 2.9 Hz, 1H), 3.57 (dd, *J* = 12.2, 5.0 Hz, 1H), 2.87 – 2.80 (m, 2H), 2.67 – 2.58 (m, 3H), 2.31 (ddt, *J* = 14.4, 6.4, 2.5 Hz, 1H); ^{13}C NMR (101 MHz, $CDCl_3$) δ 159.7 (dd, *J* = 295.5, 294.3 Hz), 151.3, 147.2, 140.3, 128.42, 128.37, 126.4, 123.6, 121.4, 120.2, 112.2, 94.3 (t, *J* = 5.7 Hz), 81.4 (t, *J* = 2.8 Hz), 75.1 (dd, *J* = 34.9, 17.9 Hz), 72.6 (dd, *J* = 7.7, 3.7 Hz), 63.3, 55.8, 34.8, 29.4, 21.6; ^{19}F NMR (377 MHz, $CDCl_3$) δ -80.19 (d, *J* = 17.3 Hz, 1F), -84.92 (d, *J* = 17.3 Hz, 1F). IR (film): ν (cm^{-1}) 3490, 3063, 3028, 2931, 2836, 1720, 1592, 1499, 1455, 1440, 1305, 1252, 1218, 1178, 1121, 1115, 1028, 974, 911, 942, 698, 577, 511, 466; HR-MS (ESI) m/z calcd for $C_{22}H_{22}F_2NaO_3^+$ [M+Na^+] 395.14292, found 395.14312.

4-(1,1-Difluoro-5-hydroxy-4-(2-methoxyphenoxy)pent-1-en-2-yl)phenyl 5-(2,5-dimethylphenoxy)-2,2-dimethylpentanoate (33)

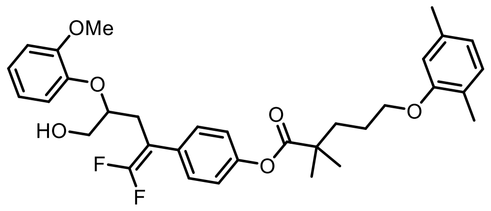

Prepared by **GP-A**. The ratio of *gem*-difluoroalkenylation product and hydroalkylation by-product is 20:1. Colorless oil, 20.7 mg, 73% yield. ^{1}H NMR (400 MHz, $CDCl_3$) ^{1}H NMR (400 MHz, $CDCl_3$) δ 7.34 – 7.28 (m, 2H), 7.07 – 6.97 (m, 4H), 6.93 – 6.82 (m, 2H), 6.78 (dd, *J* = 7.9, 1.5 Hz, 1H), 6.67 (d, *J* = 7.5 Hz, 1H), 6.64 (s, 1H), 4.13 – 4.04 (m, 1H), 4.05 – 3.97 (m, 2H), 3.83 (s, 3H), 3.68 (dd, *J* = 12.2, 2.9 Hz, 1H), 3.55 (dd, *J* = 12.2, 4.8 Hz, 1H), 3.00 (ddt, *J* = 14.8, 7.2, 1.8 Hz, 1H), 2.74 (ddt, *J* = 14.8, 6.3, 2.3 Hz, 1H), 2.55 (br s, 1H), 2.31 (s, 3H), 2.18 (s, 3H), 1.95 – 1.82 (m, 4H), 1.38 (s, 6H); ^{13}C NMR (101 MHz, $CDCl_3$) δ 176.2, 156.8, 154.3 (dd, *J* = 292.2, 289.6 Hz), 151.3, 150.2, 146.9, 136.5, 130.5 (dd, *J* = 4.0, 3.5 Hz), 130.3, 129.3 (t, *J* = 3.2 Hz), 123.7, 123.6, 121.7, 121.4, 120.8, 120.2, 112.1, 111.9, 88.6 (dd, *J* = 22.6, 15.8 Hz), 81.0 (t, *J* = 2.7 Hz), 67.7, 63.2, 55.8, 42.4, 37.1, 29.8, 25.2, 25.1, 21.4, 15.8; ^{19}F NMR (377 MHz, $CDCl_3$) δ -88.75 (d, *J* = 38.1 Hz, 1F), -89.36 (d, *J* = 38.1 Hz, 1F). IR (film): ν (cm^{-1}) 3475, 3285, 2930, 2856, 1749, 1701, 1643, 1587, 1501, 1453, 1411, 1388, 1308, 1251, 1206, 1167, 1157, 1113, 1043, 1031, 984, 958, 909, 893, 856, 846, 805, 735, 587, 522, 454, 426; HR-MS (ESI) m/z calcd for $C_{33}H_{38}F_2NaO_6^+$ [M+Na$^+$] 591.25287, found 591.25351.

5,5-Difluoro-4-(4-methoxyphenyl)-2-phenoxypent-4-en-1-ol (35)

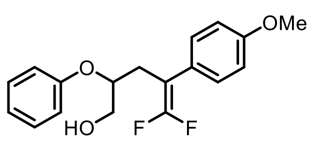

Prepared by **GP-A**. The ratio of *gem*-difluoroalkenylation product and hydroalkylation by-product is 50:1. Colorless oil, 11.8 mg, 74% yield. ^{1}H NMR (400 MHz, $CDCl_3$) δ 7.26 – 7.20 (m, 2H), 7.19 – 7.12 (m, 2H), 6.98 – 6.92 (m, 1H), 6.91 – 6.85 (m, 2H), 6.80 (d, *J* = 7.9 Hz, 2H), 4.32 (qd, *J* = 6.3, 3.4 Hz, 1H), 3.82 (s, 3H), 3.78 (dd, *J* = 11.9, 3.3 Hz, 1H), 3.68 (dd, *J* = 11.9, 5.8 Hz, 1H), 2.83 (ddt, *J* = 14.6, 5.9, 2.4 Hz, 1H), 2.67 (ddt, *J* = 14.7, 7.2, 2.1 Hz, 1H); ^{13}C NMR (101 MHz, $CDCl_3$) 158.9, 157.7, 154.1 (dd, *J* = 290.8, 289.2 Hz), 129.5, 129.5 (t, *J* = 3.1 Hz), 125.0 (dd, *J* = 3.4, 2.2 Hz), 121.5, 116.1, 114.0, 88.3 (dd, *J* = 20.9, 16.6 Hz), 63.9, 55.3, 29.4; ^{19}F NMR (377 MHz, $CDCl_3$) δ -95.73 (d, *J* = 41.1 Hz, 1F), -95.90 (d, *J* = 41.5 Hz, 1F). IR (film): ν (cm^{-1}) 3426, 2957, 2933, 2838, 1730, 1610, 1597, 1587, 1514, 1492, 1455, 1442, 1290, 1233, 1179, 1128, 1104, 1180, 1032, 981, 952, 909, 887, 832, 798, 692, 591, 564, 526, 506; HR-MS (ESI) m/z calcd for $C_{18}H_{18}F_2NaO_3^+$ [M+Na$^+$] 343.11162, found 343.11185.

2-(3,5-Dimethoxyphenoxy)-5,5-difluoro-4-(4-methoxyphenyl)pent-4-en-1-ol (37)

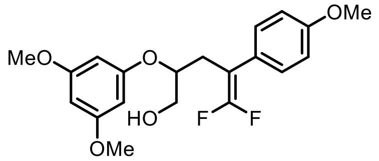

Prepared by **GP-A**. The ratio of *gem*-difluoroalkenylation product and hydroalkylation by-product is 60:1. Colorless oil, 15 mg, 79% yield. ^{1}H NMR (400 MHz, $CDCl_3$) δ 7.22 – 7.15 (m, 2H), 6.92 – 6.84 (m, 2H), 6.12 – 6.06 (m, 1H), 5.97 (d, *J* = 2.2 Hz, 2H), 4.28 (qd, *J* = 6.1, 3.4 Hz, 1H), 3.81 (s, 3H), 3.77 (dd, *J* = 11.9, 3.3 Hz, 1H), 3.71 (s, 6H), 3.66 (dd, *J* = 11.9, 5.8 Hz, 1H), 2.82 (ddt, *J* = 14.8, 6.0, 2.6 Hz, 1H), 2.66 (ddt, *J* = 14.8, 7.2, 2.2 Hz, 1H); ^{13}C NMR (101 MHz, $CDCl_3$) δ 161.5, 159.5, 159.0, 154.1 (dd, *J* = 291.1, 289.2 Hz), 129.5 (t, *J* = 3.1 Hz), 125.0 (dd, *J* = 3.4, 2.4 Hz), 114.1, 94.7,

94.0, 88.3 (dd, J = 21.2, 16.7 Hz), 63.8, 55.3, 55.2, 29.3; ^{19}F NMR (377 MHz, $CDCl_3$) δ -90.33 (d, J = 41.1 Hz, 1F), -90.52 (d, J = 41.5 Hz, 1F). IR (film): ν (cm^{-1}) 3487, 3004, 2958, 2937, 2907, 2839, 1732, 1594, 1514, 1472, 1460, 1444, 1427, 1290, 1243, 1203, 1192, 1104, 1060, 1032, 982, 947, 930, 830, 822, 798, 684, 591, 565, 527; HR-MS (ESI) m/z calcd for $C_{20}H_{22}F_2NaO_5^+$ [M+Na$^+$] 403.13275, found 403.13274.

2-(2,6-Dimethoxyphenoxy)-5,5-difluoro-4-(4-methoxyphenyl)pent-4-en-1-ol (39)

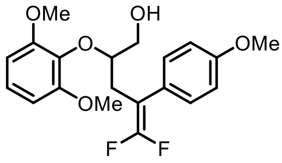

Prepared by **GP-A**. The ratio of *gem*-difluoroalkenylation product and hydroalkylation by-product is 37:1. Colorless oil, 9.2 mg, 48% yield. ^{1}H NMR (400 MHz, $CDCl_3$) δ 7.35 – 7.28 (m, 2H), 7.02 – 6.95 (m, 1H), 6.92 – 6.85 (m, 2H), 6.55 (d, J = 8.4 Hz, 2H), 4.08 – 4.01 (m, 1H), 3.80 (s, 3H), 3.78 (s, 6H), 3.61 – 3.54 (m, 1H), 3.46 – 3.39 (m, 1H), 3.07 (ddt, J = 14.4, 6.0, 3.1 Hz, 1H), 2.87 (ddd, J = 14.4, 8.4, 2.4 Hz, 1H); ^{13}C NMR (101 MHz, $CDCl_3$) δ 158.6, 154.1 (dd, J = 291.2, 288.9 Hz), 153.4, 135.4, 129.3 (t, J = 3.4 Hz), 125.6 (t, J = 3.8 Hz), 123.9, 113.8, 105.2, 88.7 (dd, J = 21.6, 14.8 Hz), 81.0 (dd, J = 3.4, 2.8 Hz), 62.5, 55.9, 55.2, 29.6; ^{19}F NMR (377 MHz, $CDCl_3$) δ -89.94 (d, J = 41.5 Hz, 1F), -90.76 (d, J = 41.5 Hz, 1F). IR (film): ν (cm^{-1}) 3506, 3004, 2938, 2838, 1727, 1609, 1594, 1514, 1492, 1477, 1464, 1444, 1293, 1249, 1183, 1107, 1028, 982, 954, 895, 833, 770, 731, 562, 526; HR-MS (ESI) m/z calcd for $C_{20}H_{22}F_2NaO_5^+$ [M+Na$^+$] 403.13275, found 403.13288.

4-((5,5-Difluoro-1-hydroxy-4-(4-methoxyphenyl)pent-4-en-2-yl)oxy)-3-methoxybenzaldehyde (41)

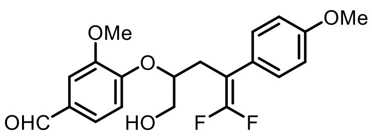

Prepared by **GP-A**. The ratio of *gem*-difluoroalkenylation product and hydroalkylation by-product is 50:1. Colorless oil, 14.0 mg, 74% yield. ^{1}H NMR (400 MHz, $CDCl_3$) δ 9.84 (s, 1H), 7.39 (d, J = 1.9 Hz, 1H), 7.34 (dd, J = 8.2, 1.9 Hz, 1H), 7.20 – 7.14 (m, 2H), 6.90 – 6.85 (m, 2H), 6.83 (d, J = 8.2 Hz, 1H), 4.35 (qd, J = 6.5, 3.4 Hz, 1H), 3.88 (s, 3H), 3.81 (s, 3H), 3.78 – 3.67 (m, 2H), 2.95 – 2.84 (m, 1H), 2.75 (ddt, J = 14.7, 7.0, 2.2 Hz, 1H), 2.11 (br s, 1H); ^{13}C NMR (101 MHz, $CDCl_3$) δ 190.8, 159.0, 154.2 (dd, J = 291.6, 289.5 Hz), 152.8, 151.0, 131.2, 129.4 (t, J = 3.1 Hz), 126.2, 124.8 (dd, J = 3.9, 3.2 Hz), 115.9, 114.1, 110.0, 88.1 (dd, J = 21.4, 16.4 Hz), 79.6 (t, J = 3.0 Hz), 63.7, 55.9, 55.3, 29.5; ^{19}F NMR (377 MHz, $CDCl_3$) δ -89.88 (d, J = 40.3 Hz, 1F), -90.24 (d, J = 40.7 Hz, 1F). IR (film): ν (cm^{-1}) 3403, 2967, 2933, 2916, 2837, 1729, 1680, 1609, 1593, 1584, 1512, 1506, 1465, 1422, 1392, 1266, 1238, 1180, 1159, 1133, 1104, 1028, 954, 902, 868, 833, 812, 781, 730, 656, 589, 566, 528; HR-MS (ESI) m/z calcd for $C_{20}H_{20}F_2NaO_5^+$ [M+Na$^+$] 401.11710, found 401.11720.

2-(3,4-Dimethoxyphenyl)-5,5-difluoro-4-(4-methoxyphenyl)pent-4-en-1-ol (47)

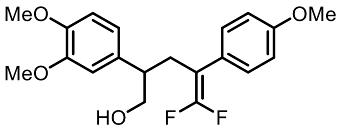

Prepared by **GP-A**. The ratio of *gem*-difluoroalkenylation product and hydroalkylation by-product is 60:1. Colorless oil, 13.3 mg, 73% yield. ^{1}H NMR (400 MHz, $CDCl_3$) δ 7.14 – 7.07 (m, 2H), 6.90 – 6.84 (m, 2H), 6.81 (d, J = 8.2 Hz, 1H), 6.65 (dd, J = 8.2, 2.0 Hz, 1H), 6.60 (d, J = 1.9 Hz, 1H), 3.87 (s, 3H), 3.83 (s, 3H), 3.81 (s, 3H), 3.74 – 3.67 (m, 2H), 2.80 – 2.61 (m, 3H); ^{13}C NMR (101 MHz, $CDCl_3$) δ 158.8, 153.7 (dd, J = 290.0, 287.1 Hz), 148.9, 148.0, 133.3, 129.5 (t, J = 3.0

Hz), 125.2 (dd, J = 4.0, 2.6 Hz), 120.1, 113.9, 111.2, 111.1, 90.1 (dd, J = 21.5, 14.9 Hz), 66.7, 55.84, 55.78, 55.2, 45.7, 30.3; ^{19}F NMR (377 MHz, $CDCl_3$) δ -91.88 (d, J = 45.2 Hz, 1F), -92.22 (d, J = 45.2 Hz, 1F). IR (film): ν (cm^{-1}) 3490, 3002, 2936, 2912, 2838, 1729, 1609, 1592, 1575, 1513, 1463, 1442, 1419, 1303, 1288, 1233, 1180, 1155, 1142, 1124, 1104, 1060, 1026, 994, 950, 833, 807, 799, 764, 731, 686, 643, 586, 563, 524; HR-MS (ESI) m/z calcd for $C_{20}H_{22}F_2NaO_4^+$ [M+Na$^+$] 387.13784, found 387.13790.

2-(3,3-Difluoro-2-(4-methoxyphenyl)allyl)-1,4-dioxane (52)

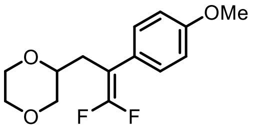

Colorless oil, 11 mg, 41% yield. ^{1}H NMR (400 MHz, $CDCl_3$) δ 7.26 – 7.21 (m, 2H), 6.93 – 6.87 (m, 2H), 3.82 (s, 3H), 3.78 – 3.72 (m, 1H), 3.71 – 3.56 (m, 4H), 3.55 – 3.48 (m, 1H), 3.27 (dd, J = 11.4, 9.9 Hz, 1H), 2.55 (dddd, J = 14.5, 6.8, 2.7, 2.2 Hz, 1H), 2.38 (dddd, J = 14.5, 6.8, 2.8, 1.9 Hz, 1H); ^{13}C NMR (126 MHz, $CDCl_3$) δ 158.9, 154.0 (dd, J = 290.2, 288.0 Hz), 129.4 (t, J = 3.2 Hz), 125.2 (t, J = 3.5 Hz), 114.0, 88.1 (dd, J = 21.5, 16.0 Hz), 73.1 (t, J = 2.8 Hz), 70.8, 66.8, 66.4, 55.2, 30.6; ^{19}F NMR (377 MHz, $CDCl_3$) ^{19}F NMR (377 MHz, $CDCl_3$) δ -90.56 (d, J = 42.6 Hz, 1F), -90.89 (d, J = 42.6 Hz, 1F). IR (film): ν (cm^{-1}) 2978, 2957, 2926, 2900, 2869, 2859, 2840, 1736, 1612, 1513, 1456, 1449, 1365, 1291, 1283, 1254, 1231, 1176, 1124, 1118, 1105, 1080, 1062, 1027, 960, 914, 878, 853, 836, 818, 806, 796, 734, 694, 659, 608, 586, 563, 526; HR-MS (EI) m/z calcd for $C_{14}H_{16}F_2O_3$ [M] 370.10675, found 370.10586.

2-(2-Methoxyphenoxy)-4-phenylbut-3-en-1-ol (53)

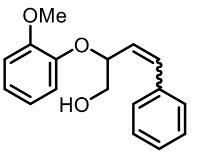

Prepared by **GP-C**. Colorless oil, 5.6 mg, 41% yield, Z/E = 5:1 from β-bromostyrene; 4.8 mg, 35% yield, Z/E = 5:1 from β-chlorostyrene. ^{1}H NMR (400 MHz, $CDCl_3$) δ Z/E mixture 7.41 – 7.31 (m, 2H), 7.31 – 7.25 (m, 1H), 7.23 – 7.02 (m, 2H), 7.01 – 6.92 (m, 1H), 6.92 – 6.66 (m, 4H), 6.29 (dd, J = 16.1, 6.8 Hz, 0.17H), 5.77 (dd, J = 11.9, 9.2 Hz, 0.83H), 5.12 (dddd, J = 9.0, 7.8, 3.5, 1.1 Hz, 0.83H), 4.72 (dddd, J = 8.1, 6.9, 3.5, 1.3 Hz, 0.17H), 3.98 – 3.78 (m, 5H), 3.14 (s, 0.17H), 3.03 (s, 0.83H); ^{13}C NMR (101 MHz, $CDCl_3$) δ major (Z) 150.48, 146.98, 136.15, 133.80, 128.56, 128.50, 128.44, 127.58, 122.72, 120.88, 118.24, 111.76, 78.63, 64.76, 55.65; minor (E) 150.68, 147.54, 136.13, 133.33, 128.57, 128.01, 126.57, 125.55, 123.09, 121.04, 119.05, 111.89, 84.13, 65.49, 55.76. IR (film): ν (cm^{-1}) 3464, 3060, 3023, 2933, 2836, 1725, 1591, 1499, 1455, 1249, 1219, 1178, 1122, 1023, 971, 912, 797, 767, 740, 699, 555, 458; HR-MS (ESI) m/z calcd for $C_{17}H_{18}NaO_3^+$ [M+Na$^+$] 293.11482, found 393.11433.

6. Extraction and reaction of native lignin

6-1. Extraction and HSQC spectra of native pine lignin

Pine wood sawdust was dried at 80 ºC for 24 h before extraction. The dried wood sawdust (50 g), 1,4-dioxane (400 mL), and 2 mol/L HCl aqueous solution (40 mL) were added to a flask. The mixture was heated to reflux under N_2 atmosphere for 1 hour at 110 °C. Then the mixture was filtered, washed with dioxane and concentrated by rotary-evaporation (40 °C) to afford a brown liquid-gel. The concentrated liquid was dissolved in a mixture of acetone and H_2O (v/v = 9:1, 250 mL). Crude lignin was obtained by the precipitation of the solution in H_2O (2 L). The precipitated lignin was isolated by

centrifugation (30 min, 4 °C), washed with deionized water, centrifuged again and dried by using lyophilizer. The resulting crude lignin was dissolved in a mixture of acetone and methanol (v/v = 9:1). The lignin was regenerated by precipitation in diethyl ether (2 L), filtered and dried under vacuum to give the purified lignin. The 2D HSQC spectra of native lignin sample is shown below (Fig. S1).

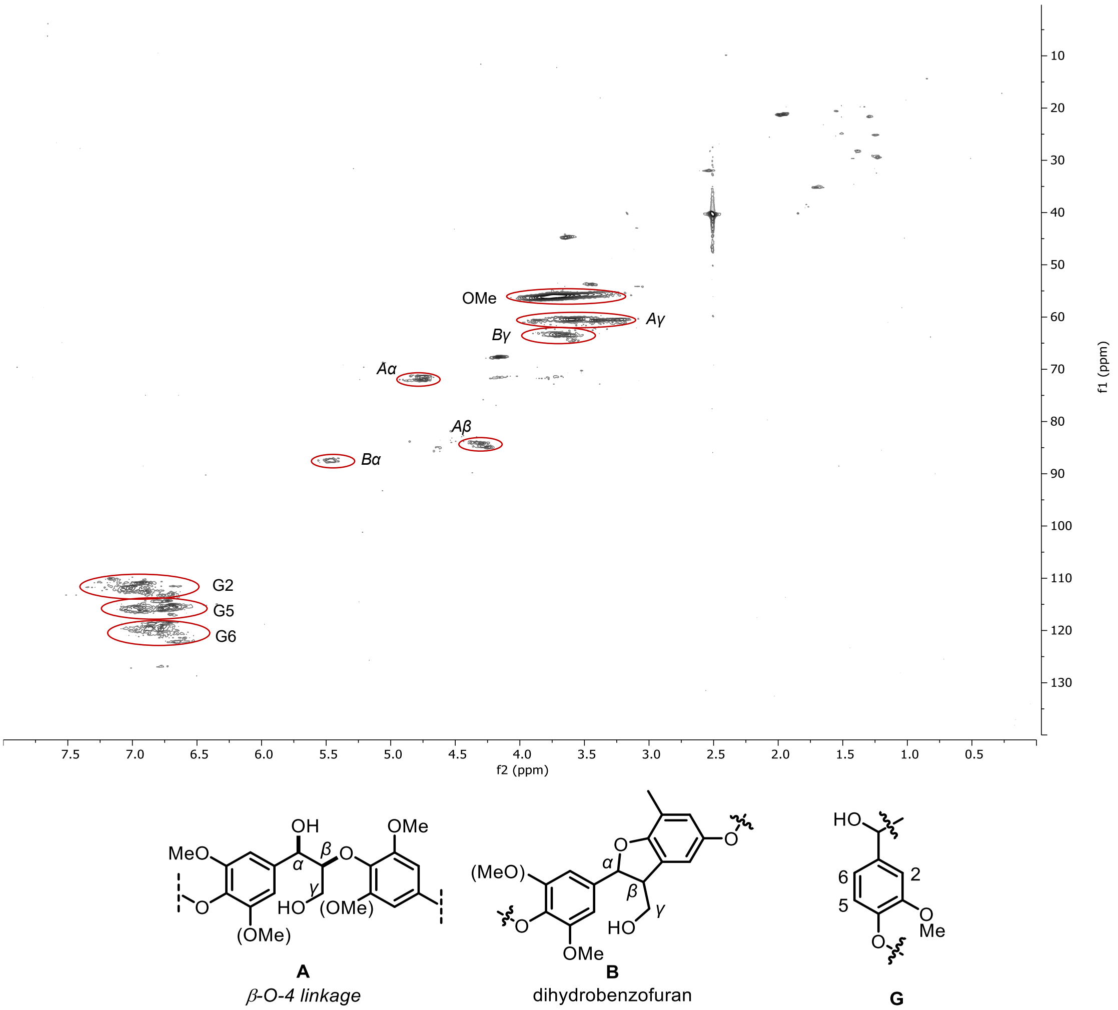

Fig S1. **Native lignin 2D HSQC.** 2D HSQC spectrum of native pine lignin before reaction.

6-2. Depolymerization and *gem*-difluoroalkenylation of native pine lignin

<u>Experimental procedure and identification of products</u>

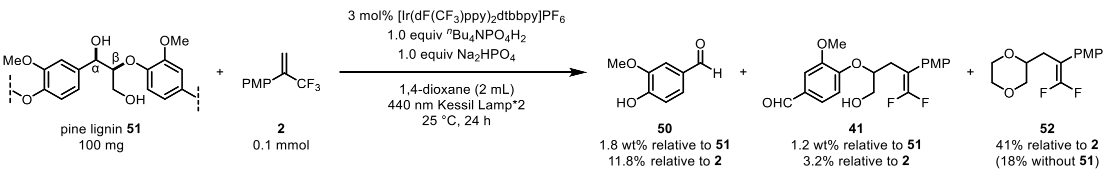

A screw cap 2-dram vial (5 mL) equipped with a stirring bar was charged with native pine lignin **51** (100 mg), $[Ir(dF(CF_3)ppy)_2(dtbbpy)]PF_6$ (0.003 mmol, 3 mmol%), $^nBu_4NPO_4H_2$ (1.0 equiv, 0.1 mmol), and Na_2HPO_4 (1.0 equiv, 0.1 mmol). The vial was sealed, then evacuated and refilled with N_2 three times. Dry 1,4-dioxane (0.05 M, 2.0 mL) and CF_3-substituted alkene **2** (1.0 equiv, 0.1 mmol) were added. The reaction mixture was irradiated with two 40 W Kessil® PR160-440nm lamps at 25 ºC with a

distance of around 2 cm from the surface of the reaction vial. After 24 h of irradiation, the resulting mixture was concentrated under reduced pressure. Ethyl acetate was then added, and the mixture was filtered through a pipette plug of silica gel and eluted with EtOAc (three times). The organic soluble fraction was concentrated under reduced pressure. The low-molecular weight products can be detected by TLC and were analyzed by GC-MS (Fig. S2). The generation of products **50**, **52**, and **41** was confirmed by comparing their mass spectra with those of the corresponding standard compounds prepared independently (Figs. S4, S5, and S6). The isolated yields were obtained after purification by flash column chromatography. A control experiment in the absence of pine lignin **51** was also carried out, and the yield of **52** was determined by ^{1}H NMR by using 1,3,5-trimethoxybenzene as internal standard.

Note: *gem*-Difluoroalkenylation product **41** shows a low peak on GC-MS, likely due to its weak responsiveness at low concentrations. This was also the case for an independently synthesized compound **41** (Fig. S6-a).

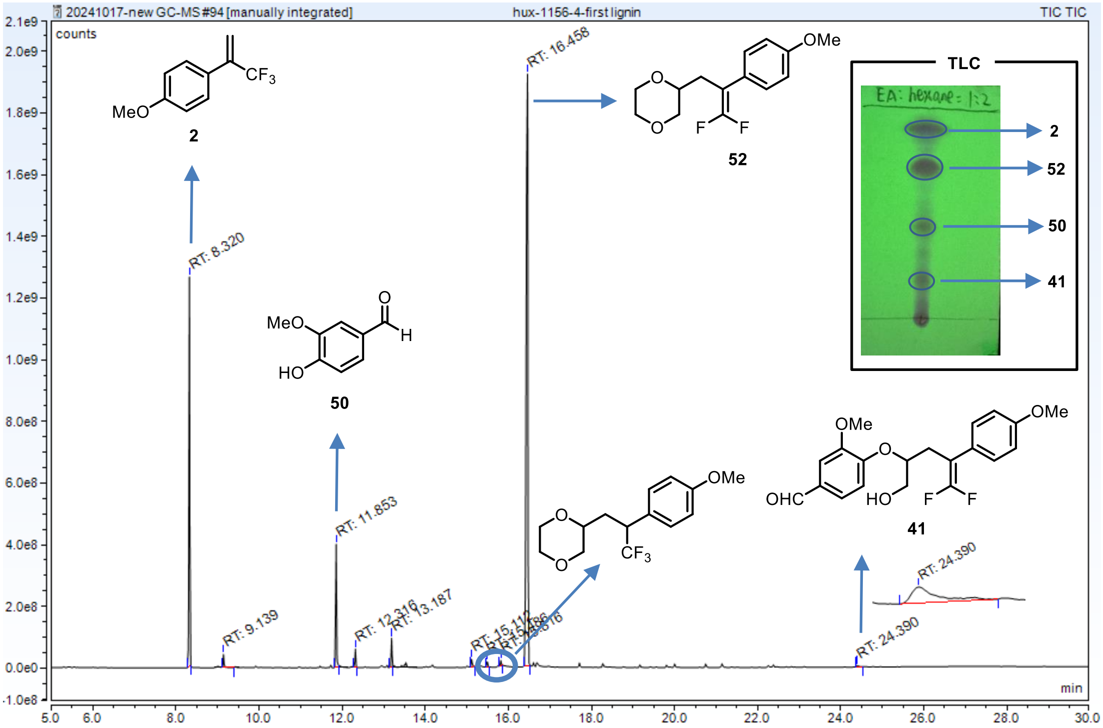

Fig. S2. **TLC and GC-MS traces.** TLC and GC-MS traces for the reaction mixture after depolymerization and *gem*-difluoroalkenylation of pine lignin.

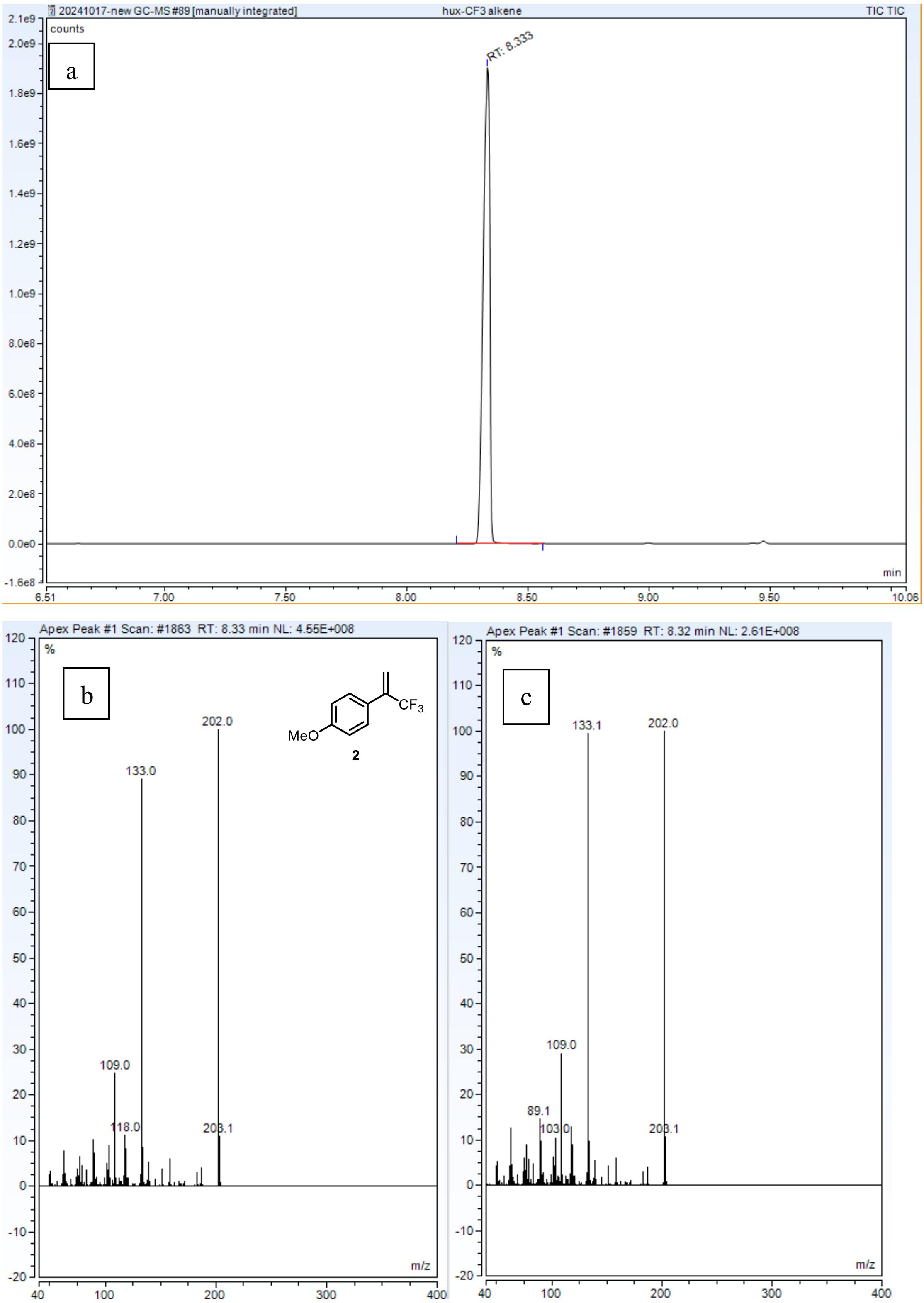

Fig. S3. **GC-MS traces of compound 2.** a. GC-MS traces of standard **2**; b. Mass spectrum of standard **2**; c. Mass spectrum of starting marterial **2** from reaction mixture.

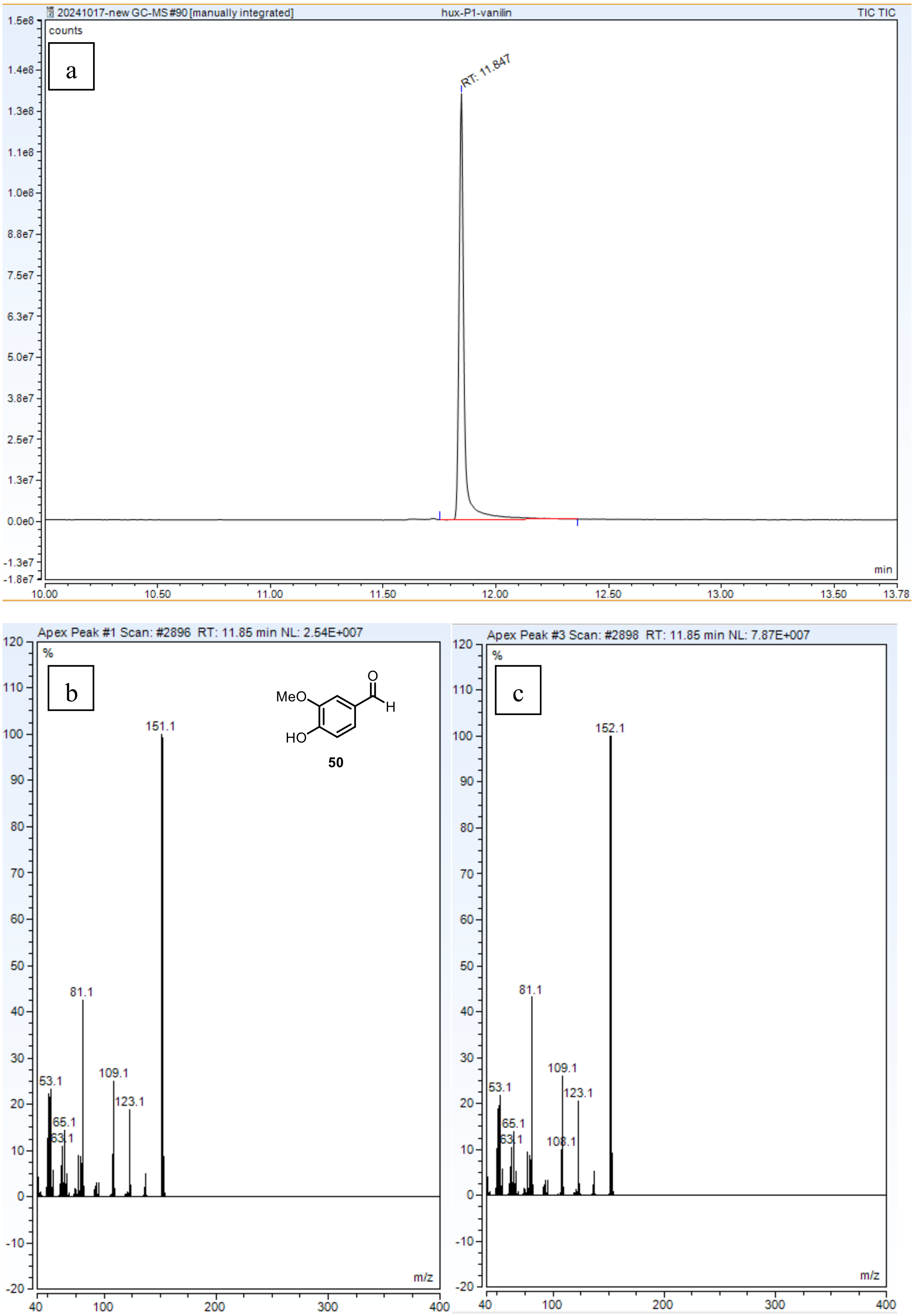

Fig. S4. **GC-MS traces of compound 50.** a. GC-MS traces of standard **50**; b. Mass spectrum of standard **50**; c. Mass spectrum of product **50** from reaction mixture.

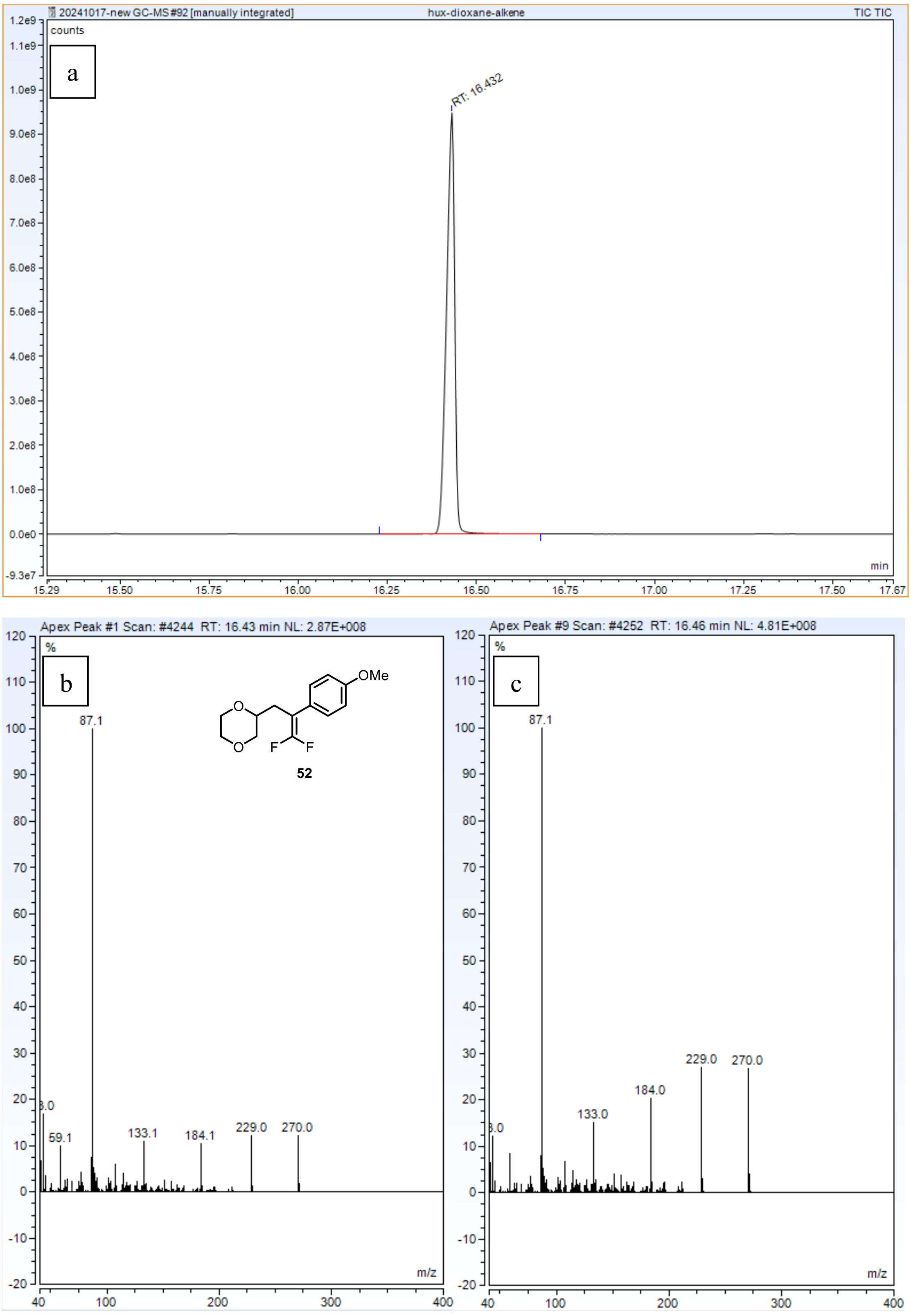

Fig. S5. **GC-MS traces of compound 52.** a. GC-MS traces of standard **52**; b. Mass spectrum of standard **52**; c. Mass spectrum of product **52** from reaction mixture.

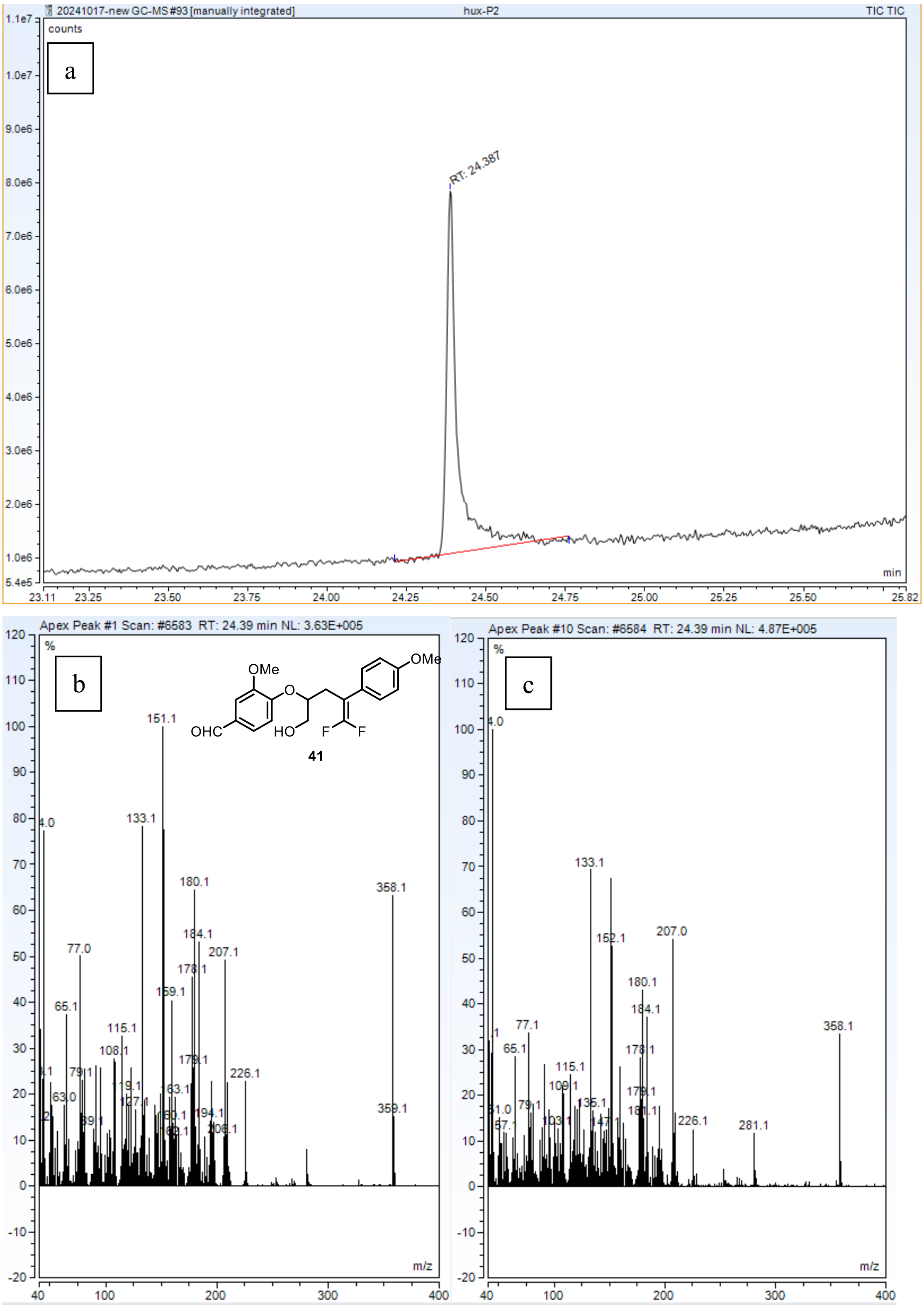

Fig. S6. **GC-MS traces of compound 41.** a. GC-MS traces of standard **41**; b. Mass spectrum of standard **41**; c. Mass spectrum of product **41** from reaction mixture.

<u>HSQC analysis for depolymerized lignin</u>

A screw cap 2-dram vial (5 mL) equipped with a stirring bar was charged with native pine lignin **51** (100 mg), $[Ir(dF(CF_3)ppy)_2(dtbbpy)]PF_6$ (0.003 mmol, 3 mmol%), $^{n}Bu_4NPO_4H_2$ (1.0 equiv, 0.1 mmol), and Na_2HPO_4 (1.0 equiv, 0.1 mmol). The vial was

sealed, then evacuated and refilled with N_2 three times. Dry 1,4-dioxane (0.05 M, 2.0 mL) and CF_3-substituted alkene **2** (1.0 equiv, 0.1 mmol) were added. The reaction mixture was irradiated with two 40 W Kessil® PR160-440nm lamps at 25 °C with a distance of around 2 cm from the surface of the reaction vial. After 24 h of irradiation, the resulting mixture was concentrated under reduced pressure. Then the mixture was subjected to HSQC analysis using DMSO-d_6 as the solvent (Fig. S7).

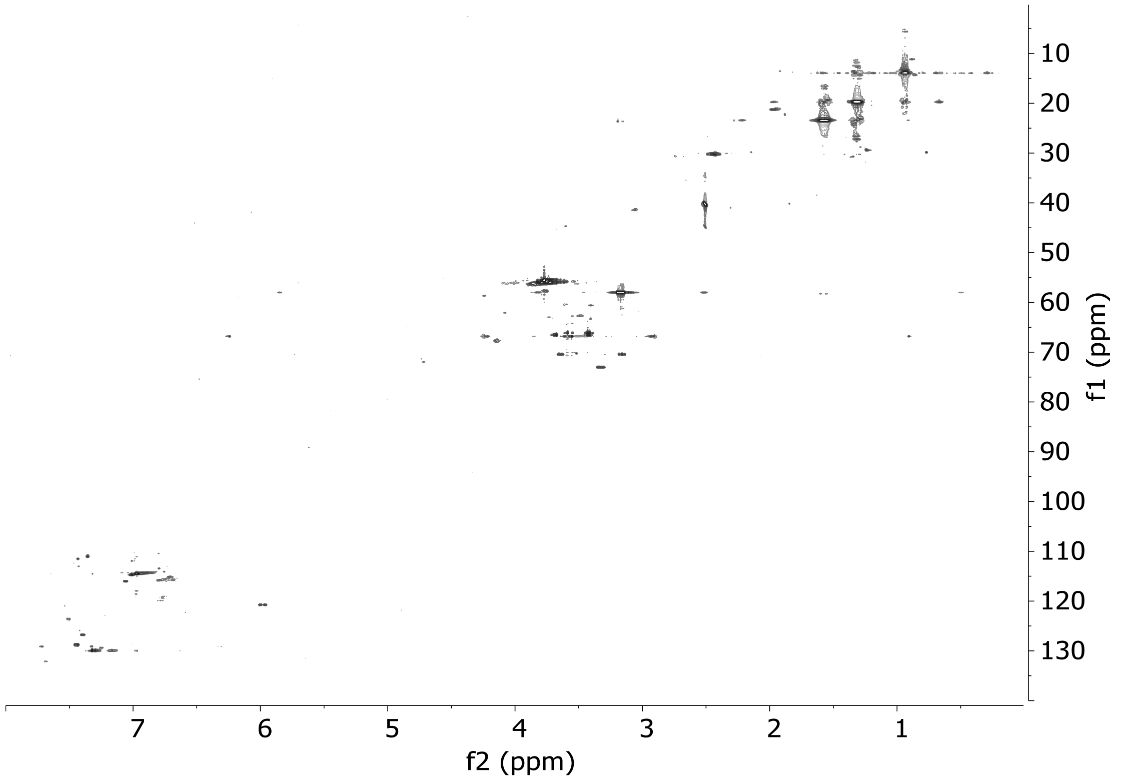

Fig. S7. **2D HSQC.** 2D HSQC spectrum of reaction mixture after reaction.

7. Derivatization of products

2-(5,5-Difluoro-2-(2-methoxyphenoxy)-4-(4-methoxyphenyl)pent-4-en-1-yl)isoindoline-1,3-dione (54)

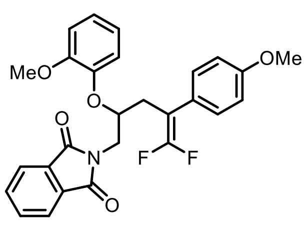

An oven-dried schlenk tube was charged with 5,5-difluoro-2-(2-methoxyphenoxy)-4-(4-methoxyphenyl)pent-4-en-1-ol (**4**, 0.05 mmol, 1.0 equiv), PPh_3 (0.075 mmol, 1.5 equiv), isoindoline-1,3-dione (0.075 mmol, 1.5 equiv), and THF (1 mL). DEAD (0.075 mmol, 1.5 equiv) was added slowly at 0 °C under a N_2 atmosphere. The reaction mixture was stirred at 25 °C for 16 h. The solvent was evaporated under reduced pressure, and the crude oil was purified by chromatography on silica gel to give the title compound as a colorless oil in 96% yield (23.0 mg). ^{1}H NMR (400 MHz, $CDCl_3$) δ 7.81 – 7.76 (m, 2H), 7.71 – 7.64 (m, 2H), 7.14 – 7.06 (m, 2H), 6.88 – 6.77 (m, 4H), 6.75 – 6.67 (m, 2H), 4.64 – 4.54 (m, 1H), 4.06 (dd, J = 14.0, 8.2 Hz, 1H), 3.84 – 3.74 (m, 4H), 3.54 (s, 3H), 2.93 (ddt, J = 14.8, 6.8, 2.1 Hz, 1H), 2.70 (ddt, J = 14.7, 6.4, 2.3 Hz, 1H); ^{13}C NMR (101 MHz, $CDCl_3$) δ 168.1, 158.8, 154.1 (dd, J = 290.8, 289.2 Hz), 150.6, 146.9, 133.8, 132.0, 129.5 (t, J = 3.0 Hz), 125.0 (dd, J = 3.9, 3.0 Hz), 123.1, 122.7, 120.7, 118.4,

113.9, 112.2, 88.5 (dd, *J* = 21.1, 16.4 Hz), 75.1 (t, *J* = 3.0 Hz), 55.4, 55.2, 41.6, 32.0; ^{19}F NMR (377 MHz, $CDCl_3$) δ -90.09 (d, *J* = 41.1 Hz, 1F), -90.40 (d, *J* = 41.1 Hz, 1F). IR (film): ν (cm^{-1}) 3063, 2939, 2837, 2363, 2337, 1775, 1711, 1610, 1593, 1514, 1498, 1466, 1457, 1427, 1395, 1372, 1292, 1248, 1223, 1179, 1123, 1112, 1023, 905, 832, 797, 746, 722, 714, 694, 567, 528; HR-MS (ESI) m/z calcd for $C_{27}H_{23}F_2NNaO_5^+$ [M+Na$^+$] 502.14365, found 502.14372.

1-((1-Bromo-5,5-difluoro-4-(4-methoxyphenyl)pent-4-en-2-yl)oxy)-2-methoxybenzene (55)

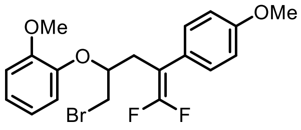

An oven-dried schlenk tube was charged with 5,5-difluoro-2-(2-methoxyphenoxy)-4-(4-methoxyphenyl)pent-4-en-1-ol (**4**, 0.05 mmol, 1.0 equiv), PPh_3 (0.06 mmol, 1.2 equiv), and CBr_4 (0.055 mmol, 1.1 equiv). CH_2Cl_2 (1 mL) was added at 0 °C under a N_2 atmosphere. The reaction mixture was stirred at 0 °C for 2 h. The solvent was evaporated under reduced pressure, and the residue was purified by chromatography on silica gel to give the title compound as colorless oil in 91% yield (18.8 mg). ^{1}H NMR (400 MHz, $CDCl_3$) δ 7.24 – 7.18 (dd, *J* = 8.8, 1.0 Hz, 2H), 7.02 – 6.95 (m, 1H), 6.91 – 6.79 (m, 5H), 4.36 – 4.25 (m, 1H), 3.81 (s, 3H), 3.77 (s, 3H), 3.53 – 3.44 (m, 2H), 3.00 – 2.86 (m, 2H); ^{13}C NMR (101 MHz, $CDCl_3$) δ 158.8, 154.3 (dd, *J* = 291.1, 289.7 Hz), 151.1, 146.5, 129.4 (t, *J* = 3.2 Hz), 124.9 (t, *J* = 3.6 Hz), 123.4, 120.9, 119.2, 113.9, 112.5, 88.3 (dd, *J* = 21.4, 16.1 Hz), 77.4 (dd, *J* = 6.0, 3.1 Hz), 55.7, 55.2, 33.8, 31.9 (d, *J* = 1.7 Hz); ^{19}F NMR (377 MHz, $CDCl_3$) δ -89.64 (d, *J* = 40.0 Hz, 1F), -90.18 (d, *J* = 40.0 Hz, 1F). IR (film): ν (cm^{-1}) 3004, 2959, 2938, 2912, 2836, 1729, 1610, 1591, 1513, 1498, 1456, 1438, 1418, 1293, 1248, 1178, 1112, 1026, 912, 832, 798, 746, 566, 527; HR-MS (ESI) m/z calcd for $C_{19}H_{19}BrF_2NaO_3^+$ [M+Na$^+$] 435.03778, found 435.03804.

2-((5,5-Difluoro-2-(2-methoxyphenoxy)-4-(4-methoxyphenyl)pent-4-en-1-yl)thio)benzo[d]thiazole (56)

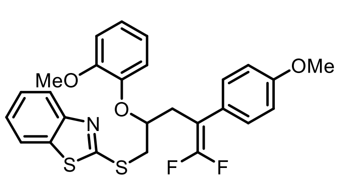

An oven-dried schlenk tube was charged with 5,5-difluoro-2-(2-methoxyphenoxy)-4-(4-methoxyphenyl)pent-4-en-1-ol (**4**, 0.05 mmol, 1.0 equiv), PPh_3 (0.06 mmol, 1.2 equiv), 2(3H)-benzothiazolethione (0.055 mmol, 1.1 equiv), and THF (1 mL). DEAD (0.06 mmol, 1.2 equiv) was added slowly at 0 °C under a N_2 atmosphere. The reaction mixture was stirred at 25 °C for 16 h. The solvent was evaporated under reduced pressure, and the residue was purified by chromatography on silica gel to give the title compound as a colorless oil in 92% yield (22.9 mg). ^{1}H NMR (400 MHz, $CDCl_3$) δ 7.83 – 7.78 (m, 1H), 7.77 – 7.71 (m, 1H), 7.42 (ddd, *J* = 8.2, 7.3, 1.2 Hz, 1H), 7.30 (ddd, *J* = 8.4, 7.4, 1.2 Hz, 1H), 7.22 – 7.14 (m, 2H), 6.96 (ddd, *J* = 8.0, 7.3, 1.6 Hz, 1H), 6.91 (dd, *J* = 8.0, 1.6 Hz, 1H), 6.86 (dd, *J* = 8.1, 1.5 Hz, 1H), 6.79 (ddt, *J* = 11.9, 5.1, 2.3 Hz, 3H), 4.62 (p, *J* = 6.0 Hz, 1H), 3.75 (s, 3H), 3.73 (s, 3H), 3.68 (dd, *J* = 5.7, 2.8 Hz, 2H), 3.04 – 2.87 (m, 2H); ^{13}C NMR (101 MHz, $CDCl_3$) δ 166.3, 158.7, 154.2 (dd, *J* = 291.0, 289.1 Hz), 153.0, 150.7, 147.0, 135.4, 129.5 (t, *J* = 3.2 Hz), 126.0, 125.0 (t, *J* = 3.7 Hz), 124.2, 122.6, 121.4, 121.0, 120.8, 117.8, 113.8, 112.2, 88.5 (dd, *J* = 21.3, 16.0 Hz), 76.8 (t, *J* = 3.0 Hz), 55.7, 55.1, 36.7, 32.6; ^{19}F NMR (377 MHz, $CDCl_3$) δ -89.93 (d, *J* = 41.1 Hz, 1F), -90.43 (d, *J* =

41.1 Hz, 1F). IR (film): ν (cm^{-1}) 3062, 3000, 2954, 2932, 2835, 1729, 1609, 1591, 1512, 1498, 1456, 1427, 1290, 1250, 1221, 1177, 1123, 1077, 1028, 995, 943, 887, 831, 798, 753, 726, 672, 566, 526; HR-MS (ESI) m/z calcd for $C_{26}H_{23}F_2NNaO_3S_2^+$ [M+Na$^+$] 522.09796, found 522.09798.

5,5-Difluoro-2-(2-methoxyphenoxy)-4-(4-methoxyphenyl)pent-4-en-1-yl 2-(1-(4-chlorobenzoyl)-5-methoxy-2-methyl-1H-indol-3-yl)acetate (57)

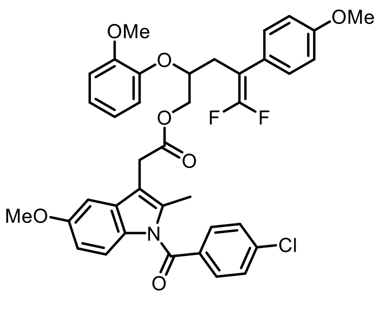

An oven-dried schlenk tube was charged with 5,5-difluoro-2-(2-methoxyphenoxy)-4-(4-methoxyphenyl)pent-4-en-1-ol (**4**, 0.07 mmol, 1.0 equiv), DCC (0.14 mmol, 2.0 equiv), and DMAP (0.007 mmol, 0.1 equiv). CH_2Cl_2 (2 mL) and indomethacin (0.084 mmol, 1.2 equiv) were added under N_2 atmosphere. The reaction mixture was stirred for 16 h at 25 ºC. After this time, water was added, and the aqueous phase was extracted with CH_2Cl_2 three times. The combined organic layers were dried (anhydrous $MgSO_4$), filtered, and the solvent was evaporated under reduced pressure. The crude mixture was purified by chromatography on silica gel to give the title compound as a colorless oil in 98% yield (47.3 mg). ^{1}H NMR (400 MHz, $CDCl_3$) δ 7.66 – 7.57 (m, 2H), 7.47 – 7.39 (m, 2H), 7.12 – 7.09 (m, 2H), 6.98 – 6.92 (m, 2H), 6.90 (d, J = 9.0 Hz, 1H), 6.85 (dd, J = 8.1, 1.1 Hz, 1H), 6.83 – 6.78 (m, 2H), 6.77 – 6.71 (m, 1H), 6.71 – 6.65 (m, 2H), 4.36 – 4.18 (m, 3H), 3.79 (s, 3H), 3.79 (s, 3H), 3.74 (s, 3H), 3.65 (s, 2H), 2.90 – 2.81 (m, 1H), 2.77 – 2.66 (m, 1H), 2.33 (s, 3H); ^{13}C NMR (101 MHz, $CDCl_3$) δ 170.5, 168.2, 158.8, 156.1, 154.1 (dd, J = 291.1, 289.3 Hz), 150.9, 146.8, 139.2, 135.9, 133.9, 131.1, 130.7, 130.6, 129.3 (t, J = 3.1 Hz), 129.0, 124.7 (t, J = 3.0 Hz), 123.1, 120.8, 118.7, 114.9, 113.9, 112.3, 112.2, 111.8, 101.1, 88.2 (dd, J = 20.8, 16.4 Hz), 76.0 (t, J = 2.9 Hz), 65.2, 55.59, 55.57, 55.2, 30.2, 30.1, 13.3; ^{19}F NMR (377 MHz, $CDCl_3$) δ -90.13 (d, J = 41.1 Hz, 1F), -90.32 (d, J = 40.7 Hz, 1F). IR (film): ν (cm^{-1}) 3002, 2954, 2931, 2836, 2366, 2341, 1735, 1681, 1609, 1592, 1514, 1499, 1477, 1456, 1439, 1357, 1315, 1290, 1250, 1223, 1177, 1166, 1140, 1189, 1066, 1030, 1014, 925, 910, 832, 808, 799, 752, 732, 689, 648, 565, 482; HR-MS (ESI) m/z calcd for $C_{38}H_{34}ClF_2NNaO_7^+$ [M+Na$^+$] 712.18841, found 712.18926.

(*E*)-5-Fluoro-5-(1H-imidazol-1-yl)-2-(2-methoxyphenoxy)-4-(4-methoxyphenyl)pent-4-en-1-ol (58)

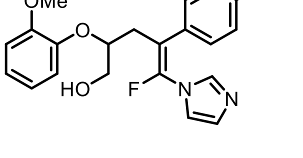

An oven-dried schlenk tube was charged with 5,5-difluoro-2-(2-methoxyphenoxy)-4-(4-methoxyphenyl)pent-4-en-1-ol (**4**, 0.05 mmol, 1.0 equiv), 1H-imidazole (0.075 mmol, 1.5 equiv), anhydrous Cs_2CO_3 (0.1 mmol, 2.0 equiv), anhydrous DMF (2 mL) was added at 25 °C under a N_2 atmosphere. The resulting mixture was stirred at 25 ºC for 20 h. After this time, the reaction mixture was quenched with saturated NH_4Cl solution. The aqueous layer was extracted with EtOAc three times. The combined organic layers were washed with brine and dried over anhydrous magnesium sulfate, filtered, and concentrated under reduced pressure. The residue was purified by chromatography on silica gel to give the title compound as a colorless oil in

71% yield (14.1 mg). ^{1}H NMR (400 MHz, $CDCl_3$) δ 7.31 (s, 1H), 7.00 (ddd, *J* = 8.2, 7.2, 1.8 Hz, 1H), 6.96 (s, 1H), 6.93 – 6.87 (m, 3H), 6.86 – 6.81 (m, 2H), 6.81 – 6.74 (m, 3H), 4.21 – 4.14 (m, 1H), 3.82 (s, 3H), 3.76 (s, 3H), 3.70 (dd, *J* = 12.5, 2.7 Hz, 1H), 3.61 (dd, *J* = 12.1, 5.0 Hz, 1H), 3.23 (ddd, *J* = 14.3, 7.3, 2.9 Hz, 1H), 2.89 (ddd, *J* = 14.4, 6.3, 3.1 Hz, 1H); ^{13}C NMR (101 MHz, $CDCl_3$) δ 159.3, 151.2, 146.9, 143.2 (d, *J* = 260.8 Hz), 137.2, 129.4, 129.3 (d, *J* = 3.0 Hz), 126.4 (d, *J* = 4.3 Hz), 123.6, 121.2, 119.7, 118.7, 114.4, 112.2, 110.9 (d, *J* = 23.6 Hz), 80.4 (d, *J* = 3.0 Hz), 63.6, 55.8, 55.2, 32.4; ^{19}F NMR (377 MHz, $CDCl_3$) δ -92.24. IR (film): ν (cm^{-1}) 3253, 3122, 2932, 2838, 2121, 1703, 1607, 1593, 1512, 1498, 1457, 1441, 1380, 1293, 1249, 1221, 1178, 1142, 1123, 1077, 1026, 972, 911, 833, 740, 657, 610, 578, 533; HR-MS (EI) m/z calcd for $C_{22}H_{24}FN_2O_4^+$ [M+H$^+$] 399.17146, found 399.17109.

6-Fluoro-3-(2-methoxyphenoxy)-5-(4-methoxyphenyl)-3,4-dihydro-2H-pyran (59)

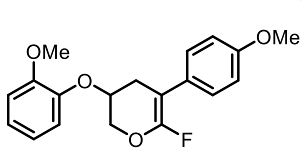

An oven-dried schlenk tube was charged with 5,5-difluoro-2-(2-methoxyphenoxy)-4-(4-methoxyphenyl)pent-4-en-1-ol (**4**, 0.05 mmol, 1.0 equiv), *^{t}*BuOK (0.125 mmol, 2.5 equiv.), dry toluene (1.0 mL) was added at 25 °C under a N_2 atmosphere. The resulting mixture was stirred at 25 °C for 12 h. After this time, the reaction mixture was filtered through a silica gel plug with EA (10 mL) as the eluent. The organic phase was concentrated under reduced pressure, and the residue was purified by chromatography on silica gel to give the title compound as a colorless oil in 88% yield (14.5 mg). ^{1}H NMR (400 MHz, $CDCl_3$) δ 7.30 (d, *J* = 8.6 Hz, 2H), 7.09 – 7.00 (m, 2H), 6.98 – 6.83 (m, 4H), 4.77 – 4.67 (m, 1H), 4.41 – 4.26 (m, 2H), 3.86 (s, 3H), 3.80 (s, 3H), 2.88 – 2.65 (m, 2H); ^{13}C NMR (101 MHz, $CDCl_3$) δ 157.7 (d, *J* = 1.2 Hz), 154.1 (d, *J* = 258.4 Hz), 151.5, 145.9, 128.1 (d, *J* = 4.1 Hz), 127.9 (d, *J* = 5.2 Hz), 123.7, 121.0, 119.9, 113.7, 112.6, 82.2 (d, *J* = 16.3 Hz), 71.0 (d, *J* = 1.2 Hz), 69.0 (d, *J* = 1.6 Hz), 55.9, 55.2, 29.9 (d, *J* = 3.8 Hz); ^{19}F NMR (377 MHz, $CDCl_3$) δ -91.29. IR (film): ν (cm^{-1}) 2999, 2934, 2837, 2362, 2339, 1734, 1611, 1591, 1512, 1499, 1456, 1441, 1303, 1250,1220, 1176, 1146, 1122, 1114, 1027, 948, 909, 827, 744, 569, 554, 536; HR-MS (EI) m/z calcd for $C_{19}H_{19}FO_4$ [M] 330.12674, found 330.12892.

6,6-Difluoro-3-(2-methoxyphenoxy)-5-(4-methoxyphenyl)-3,6-dihydro-2H-pyran (60)

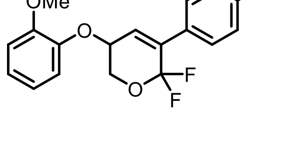

A screw cap 2-dram vial (5 mL) equipped with a stirring bar was charged with 5,5-difluoro-2-(2-methoxyphenoxy)-4-(4-methoxyphenyl)pent-4-en-1-ol (**4**, 0.05 mmol, 1.0 equiv), 3,6-di-tert-butyl-9-mesityl-phenylacridin-10-ium tetrafluoroborate (Mes-3,6-*^{t}*Bu_2-Acr-$PhBF_4$, 0.0025 mmol, 5 mol%), chloro(pyridine)bis(dimethylglyoximato)cobalt ($Co(dmgH)_2PyCl$, 0.004 mmol, 8 mol%). The vial was sealed, then evacuated and refilled with N_2 three times. Dry DCE (1.0 mL) was added. The reaction mixture was irradiated with a 40 W Kessil® PR160-440nm lamp at 25 °C for 24 h with a distance of around 2 cm from the surface of the reaction vial. The solvent was evaporated under reduced pressure, and the residue was purified by chromatography on silica gel to give the title compound as a colorless oil in 63% yield (11 mg). ^{1}H NMR (400 MHz, Acetone-d_6) δ 7.51 (d, *J* = 9.0 Hz, 2H), 7.13 (d, *J* = 7.8 Hz, 1H), 7.05 (d, *J* = 5.2 Hz, 2H), 7.01 – 6.95 (m, 2H), 6.95 – 6.85 (m,

1H), 6.62 (dd, J = 6.4, 2.8 Hz, 1H), 4.99 – 4.90 (m, 1H), 4.47 (dd, J = 12.4, 3.8 Hz, 1H), 4.38 (ddd, J = 12.3, 4.0, 0.9 Hz, 1H), 3.86 (s, 3H), 3.83 (s, 3H); ^{13}C NMR (101 MHz, Acetone-d_6) δ 161.2, 152.3, 147.5, 135.6 (t, J = 33.4 Hz), 129.5 (t, J = 1.5 Hz), 129.2 (t, J = 5.8 Hz), 126.7 (t, J = 2.0 Hz), 124.6, 121.8 (dd, J = 251.9, 244.9 Hz), 120.2, 119.4, 114.8, 113.8, 69.8 (t, J = 1.4 Hz), 67.4 (dd, J = 3.2, 1.8 Hz), 56.2, 55.6; ^{19}F NMR (376 MHz, Acetone-d_6) δ -65.88 (d, J = 176.3 Hz), -70.98 (d, J = 176.7 Hz). IR (film): ν (cm^{-1}) 3089, 3011, 2946, 2901, 2837, 2384, 2286, 2118, 1874, 1607, 1595, 1515, 1503, 1468, 1455, 1378, 1291, 1252, 1218, 1193, 1171, 1123, 1113, 1054, 1031, 1024, 1007, 976, 925, 824, 796, 744, 728, 675, 604, 573, 548, 502, 448, 420, 411; HR-MS (EI) m/z calcd for $C_{19}H_{18}F_2NaO_4^+$ [M+Na$^+$] 371.10654, found 371.10696.

2-(4,5-Dibromo-2-methoxyphenoxy)-5,5-difluoro-4-phenylpent-4-en-1-ol (61)

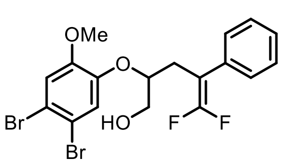

A screw cap 2-dram vial (5 mL) equipped with a stirring bar was charged with 5, 5-difluoro-2-(2-methoxyphenoxy)-4-(4-methoxyphenyl)pent-4-en-1-ol (**4**, 0.03 mmol, 1.0 equiv) and HFIP (1 mL), NBS (0.063 mmol, 2.1 equiv) was added under air. The reaction mixture was stirred at 25 ºC for 12 h. The solvent was evaporated under reduced pressure, and the residue was purified by chromatography on silica gel to give the title compound as a colorless oil in 65% yield (18.6 mg). ^1H NMR (400 MHz, $CDCl_3$) δ 7.43 – 7.34 (m, 2H), 7.34 – 7.23 (m, 3H), 7.07 (s, 1H), 6.88 (s, 1H), 4.08 (qd, J = 6.7, 3.1 Hz, 1H), 3.79 (s, 3H), 3.70 (dd, J = 12.2, 3.1 Hz, 1H), 3.62 (dd, J = 12.3, 5.2 Hz, 1H), 2.93 (dddd, J = 14.8, 6.8, 2.7, 1.7 Hz, 1H), 2.73 (ddt, J = 14.8, 6.7, 2.4 Hz, 1H); ^{13}C NMR (101 MHz, $CDCl_3$) δ 154.3 (dd, J = 292.7, 289.9 Hz), 150.7, 146.8, 132.6 (t, J = 3.6 Hz), 128.8, 128.2 (t, J = 3.0 Hz), 127.8, 123.1, 117.3, 116.7, 114.9, 88.8 (dd, J = 21.2, 15.9 Hz), 80.8 (t, J = 3.0 Hz), 63.4, 56.2, 29.3; ^{19}F NMR (377 MHz, $CDCl_3$) δ -89.08 (d, J = 38.4 Hz, 1F), -89.43 (d, J = 38.1 Hz, 1F). IR (film): ν (cm^{-1}) 3494, 2924, 2844, 2360, 2342, 1731, 1582, 1486, 1439, 1351, 1305, 1244, 1200, 1178, 1126, 1109, 1072, 1025, 982, 954, 869, 848, 796, 770, 728, 697, 651, 580, 495; HR-MS (ESI) m/z calcd for $C_{18}H_{16}Br_2F_2NaO_3^+$ [M+Na$^+$] 498.93265, found 498.93285.

5,5-Difluoro-4-(2-methoxy-5-(1-methyl-1H-pyrrol-2-yl)phenyl)-2-(2-methoxyphenoxy)pent-4-en-1-ol (62)

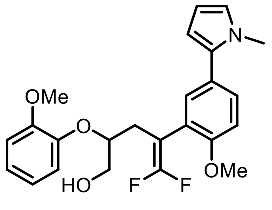

An oven-dried schlenk tube was charged with 5,5-difluoro-2-(2-methoxyphenoxy)-4-(4-methoxyphenyl)pent-4-en-1-ol (**4**, 0.03 mmol, 1.0 equiv). Then DME (1 mL), 1-methyl-2-(4,4,5,5-tetramethyl-1,3,2-dioxaborolan-2-yl)-1H-pyrrole (0.039 mmol, 1.3 equiv), $Pd(PPh_3)_4$ (0.0018 mmol, 0.06 equiv), and 2 M Na_2CO_3 (0.03 mL, 2.0 equiv) were added 25 °C under a N_2 atmosphere. The resulting mixture was stirred at 80 °C for 16 h. After this time, the solvent was evaporated under reduced pressure, and the residue was purified by chromatography on silica gel to give the title compound as colorless oil in 93% yield (12 mg). ^1H NMR (400 MHz, $CDCl_3$) δ 7.33 (dd, J = 8.5, 2.3 Hz, 1H), 7.10 (d, J = 2.0 Hz, 1H), 6.98 – 6.91 (m, 2H), 6.84 (dd, J = 8.2, 1.5 Hz, 1H), 6.81 – 6.75 (m, 1H), 6.74 – 6.64 (m, 2H), 6.21 – 6.08 (m, 2H), 4.04 (ddd, J = 12.1, 6.8, 2.9 Hz, 1H), 3.82 (s, 3H), 3.79 (s, 3H), 3.70 (dd, J = 12.1, 2.9

Hz, 1H), 3.60 (dd, J = 12.1, 5.3 Hz, 1H), 3.55 (s, 3H), 3.03 – 2.94 (m, 1H), 2.82 – 2.72 (m, 1H), 2.13 (br s, 1H); ^{13}C NMR (101 MHz, $CDCl_3$) δ 156.2 (d, J = 2.3 Hz), 153.9 (t, J = 289.4 Hz), 151.1, 147.1, 133.7, 131.6 (t, J = 2.2 Hz), 129.7, 125.9, 123.23, 123.16, 121.5 (dd, J = 4.8, 2.1 Hz), 121.1, 119.3, 112.0, 110.9, 108.2, 107.6, 86.1 (dd, J = 23.3, 18.1 Hz), 80.9 (t, J = 2.8 Hz), 63.6, 55.7, 55.5, 34.8, 29.6; ^{19}F NMR (377 MHz, $CDCl_3$) δ -87.99 (d, J = 38.8 Hz, 1F), -91.13 (d, J = 38.5 Hz, 1F). IR (film): ν (cm^{-1}) 3403, 2943, 2917, 2841, 2361, 2336, 2059, 1740, 1684, 1594, 1498, 1456, 1440, 1397, 1277, 1250, 1222, 1177, 1121, 1023, 906, 818, 809, 746, 727, 710; HR-MS (EI) m/z calcd for $C_{24}H_{25}F_2NNaO_4^+$ [M+Na^+] 452.16439, found 452.16470.

8. Mechanistic investigations

8-1. Radical trapping experiment

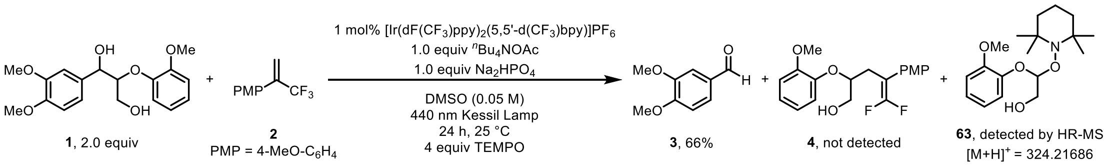

A screw cap 2-dram vial (5 mL) equipped with a stirring bar was charged with lignin model substrate **1** (2.0 equiv, 0.1 mmol), [Ir(dF(CF_3)ppy)$_2$(5,5'-d(CF_3)bpy)]PF_6 (0.0005 mmol, 1 mmol%), ^{n}Bu_4NOAc (1.0 equiv, 0.05 mmol), Na_2HPO_4 (1.0 equiv, 0.05 mmol), and 2,2,6,6-tetramethyl-1-piperidinyloxy (TEMPO, 0.2 mmol, 4 equiv). The vial was sealed, then evacuated and refilled with N_2 three times. Dry DMSO (0.05M, 1.0 mL) and CF_3-substituted alkene **2** (1.0 equiv, 0.05 mmol) was added. The reaction mixture was irradiated with a 40 W Kessil® PR160-440nm lamp at 25 ºC with a distance of around 2 cm from the surface of the reaction vial. After 24 h of irradiation, water (1 mL) was added, and the resulting mixture was extracted with EtOAc (5×2 mL). The organic layer was washed with brine (3×8 mL) and dried over anhydrous magnesium sulfate, filtered, and concentrated under reduced pressure. The mixture was analyzed by high-resolution mass spectrometry (HR-MS). It was found that TEMPO-trapped product **63** could be detected by HR-MS (Fig. S8), which suggests that the lignin-based alkyl radical is generated via the β-scission of the C-C bond under the applied conditions.

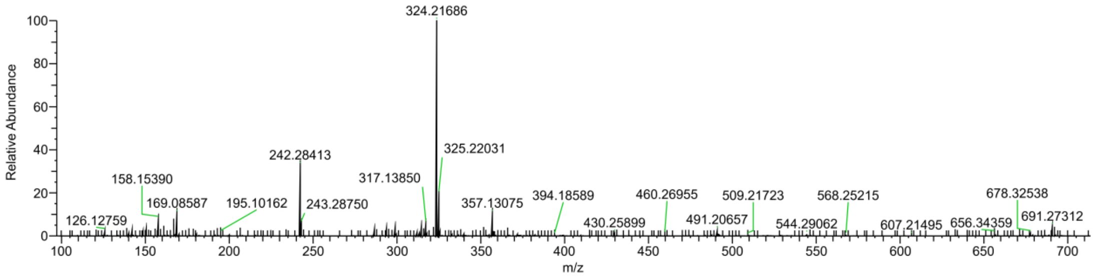

Fig. S8. **HR-MS trace.** HR-MS for radical trapping experiment under standard reaction conditions.

8-2. Stern-Volmer quenching experiments

Fluorescence quenching of [Ir(dF(CF_3)ppy)$_2$(5,5'-d(CF_3)bpy)]PF_6 was recorded with a SpectroFluorometer FS5 using Fluoracle software. Samples consisting of noted

concentration of quencher in anhydrous degassed CH_3CN were prepared under N_2 atmosphere. The solutions were irradiated at 400 nm and luminescence was measured at 595 nm. [Ir] = $4x10^{-5}$ M.

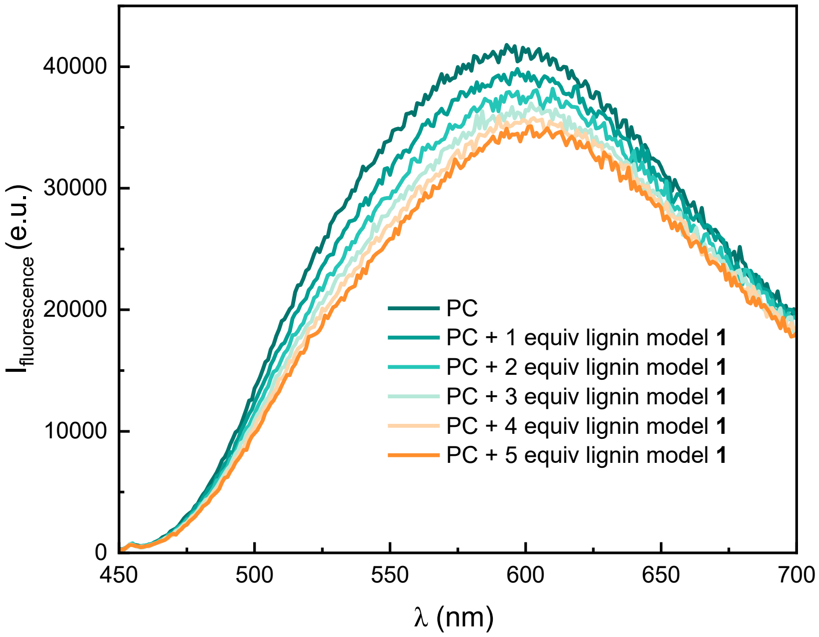

Fig. S9. **Fluorescence quenching of PC in presence of compound 1.** Evolution of fluorescence in presence of lignin model **1**.

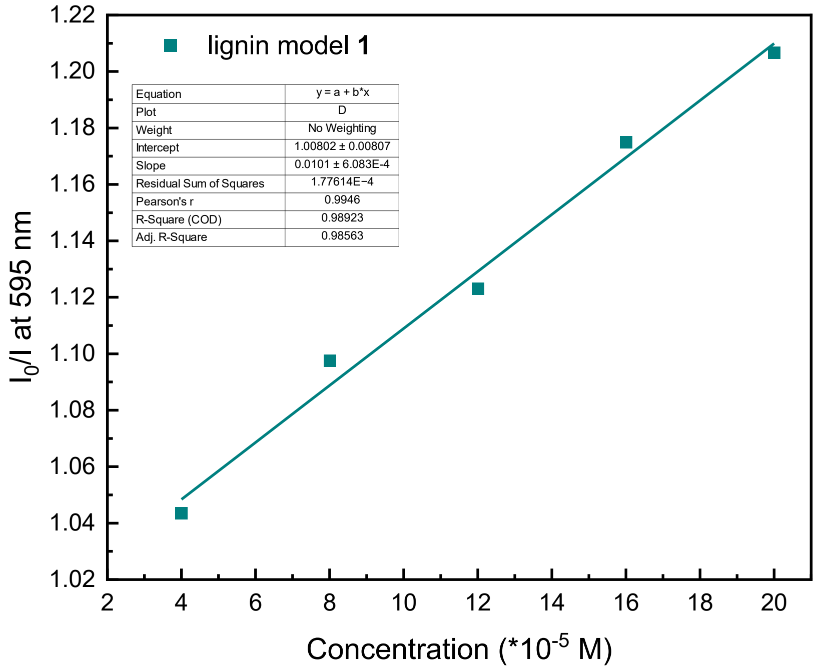

Fig. S10. **Stern-Volmer experiment for compound 1.** Plotted ratio of fluorescence intensity with concentration of lignin model **1**.

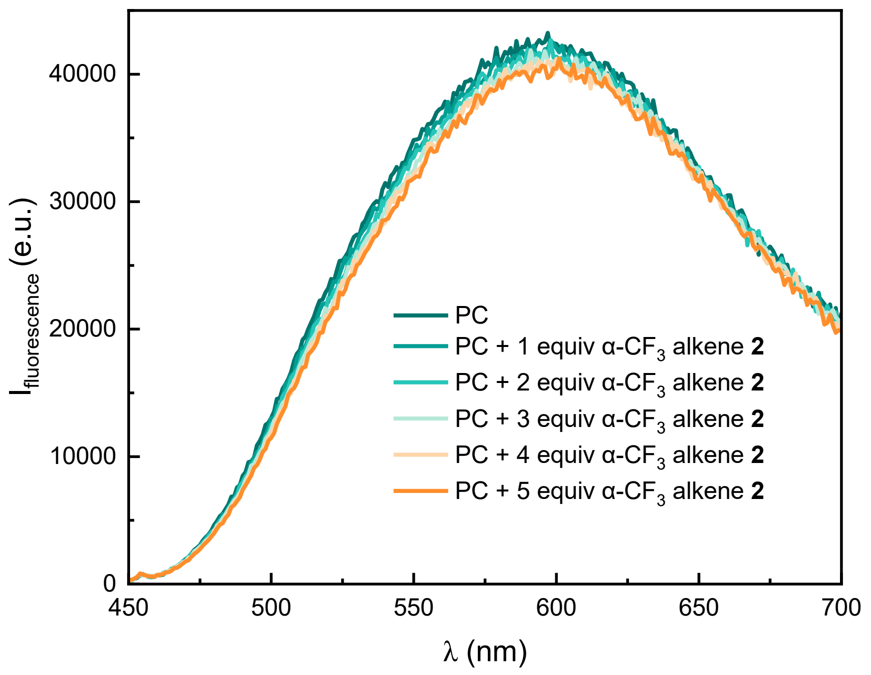

Fig. S11. **Fluorescence quenching of PC in presence of compound 2.** Evolution of fluorescence in presence of α-CF_3 alkene **2**.

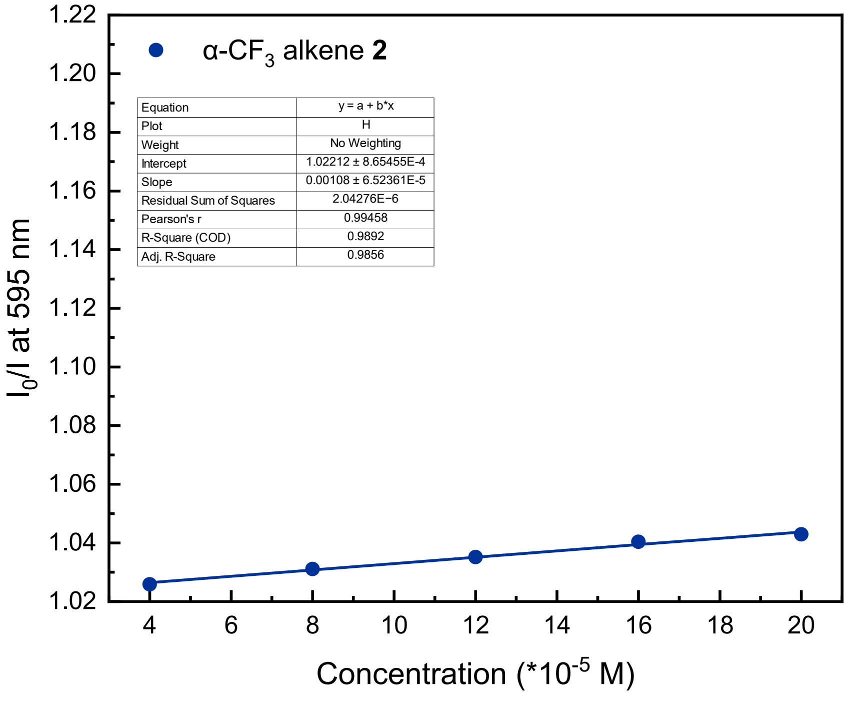

Fig. S12. **Stern-Volmer experiment for compound 2.** Plotted ratio of fluorescence intensity with concentration of α-CF_3 alkene **2**.

8-3. Cyclic voltammetry studies

Cyclic voltammetry experiments were performed in a three-electrode cell connected to N_2 at room temperature. A working glass carbon electrode, platinum wire counter electrode, and Ag|AgCl reference electrode were employed. Anhydrous degassed CH_3CN (5 mL) containing 1.0 mmol $^nBu_4NBF_4$ were poured into the electrochemical cell in all experiments. The concentration of compounds is 3 mM. The scan rate is 100 mV/s. All cyclic voltammograms were normalized by adding 1.0 equiv of freshly

sublimed ferrocene and collecting a new voltammogram. The ½ wave potential of the Fc/Fc^+ peak was identified and set to 0.0 V. Data was analyzed by subtracting a background current prior to identifying the maximum current (Cp) and determining the potential (Ep/2) at half this value (Cp/2).[90] As shown in Fig. S14, the oxidative peak of lignin model **1** was observed at 0.99 V and the oxidative half-peak potential of **1** was measured as 0.91 V. The oxidative peak of α-CF_3 alkene **2** was observed at 1.39 V and the oxidative half-peak potential of **2** was measured as 1.30 V.

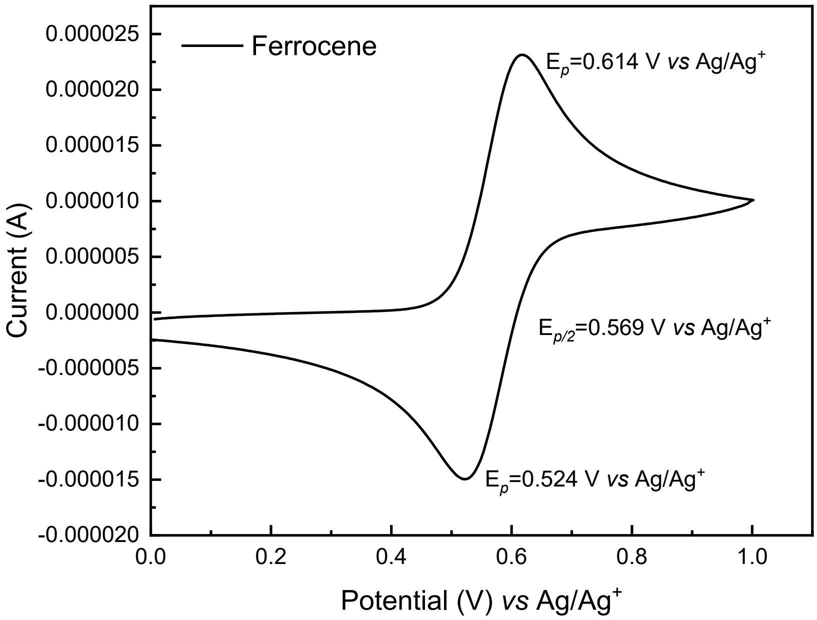

Fig. S13. **Cyclic voltammograms of ferrocene.** Cyclic voltammograms of ferrocene vs. Ag/Ag^+.

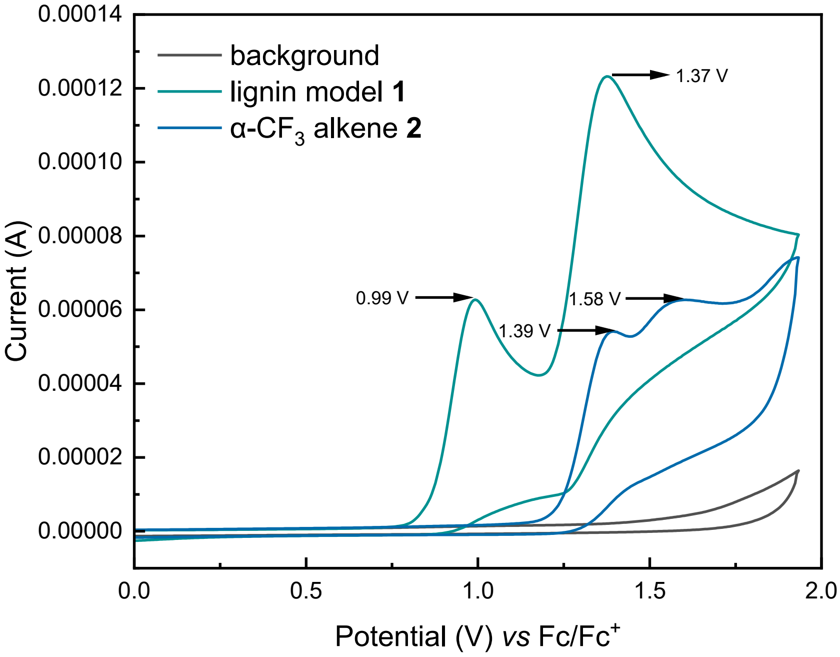

Fig. S14. **Cyclic voltammograms of compound 1 and 2**. Cyclic voltammograms of lignin model **1** and α-CF_3 alkene **2** vs. Fc/Fc^+.

10. NMR spectra

Compound **S7**

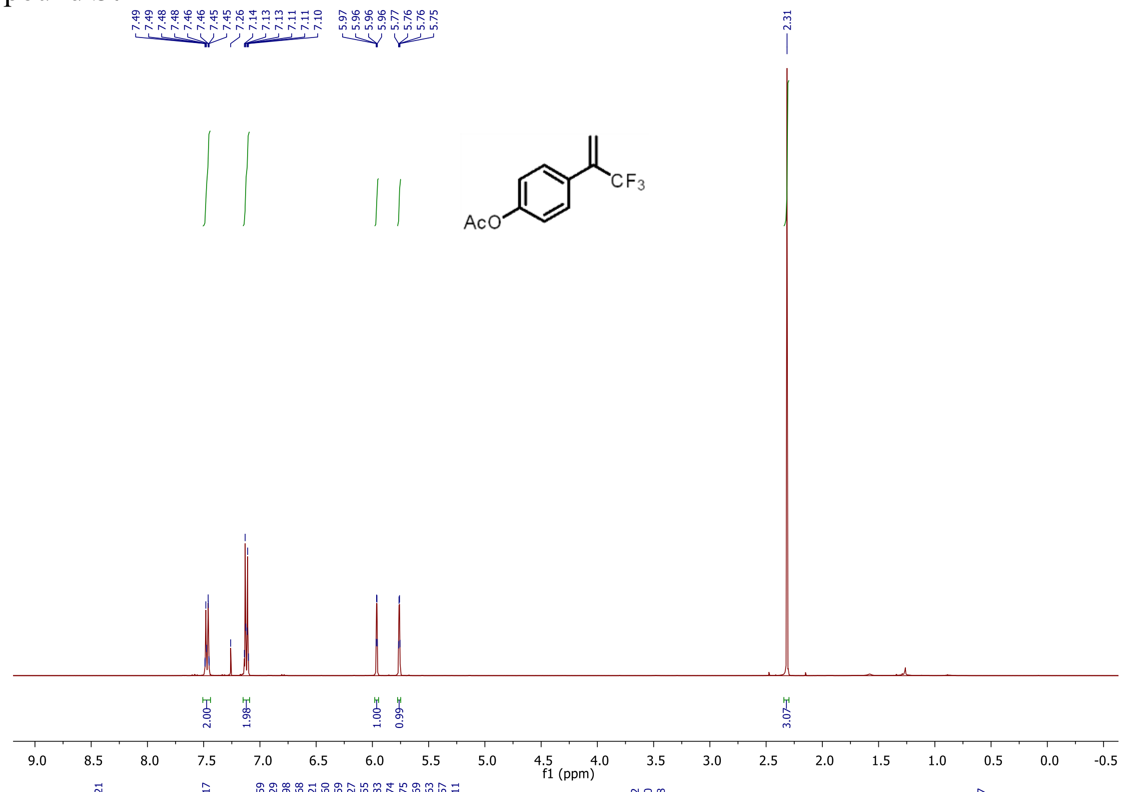

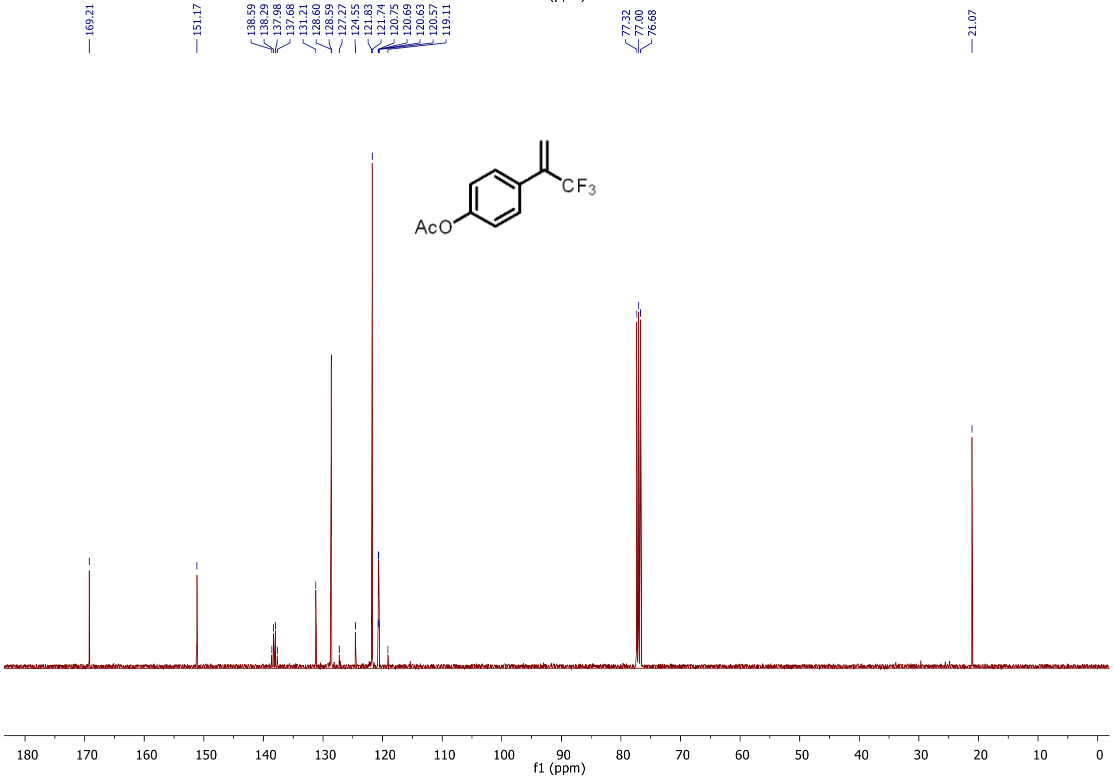

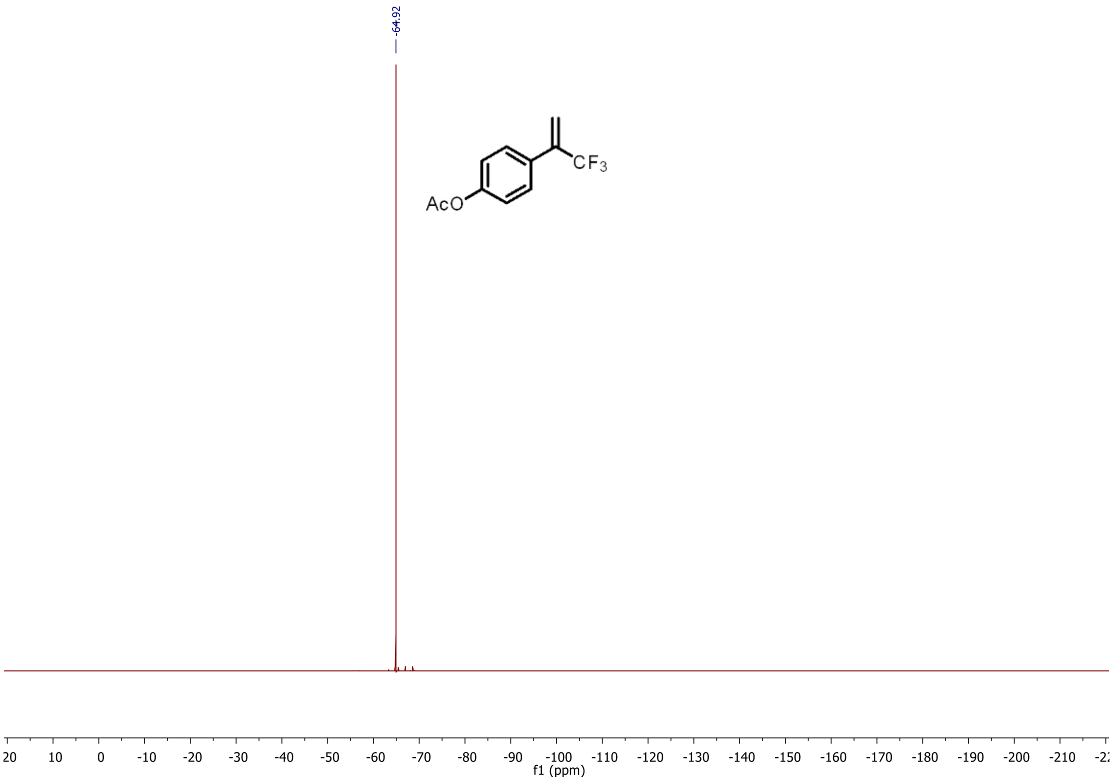

Compound **S12**

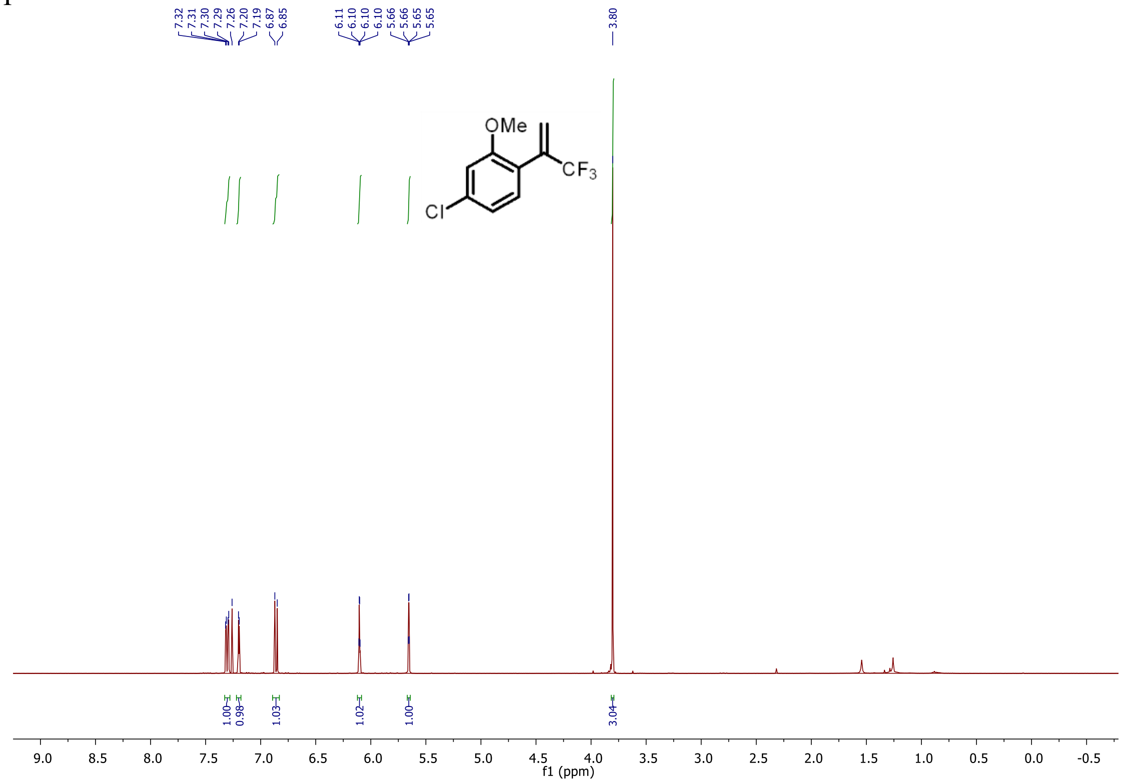

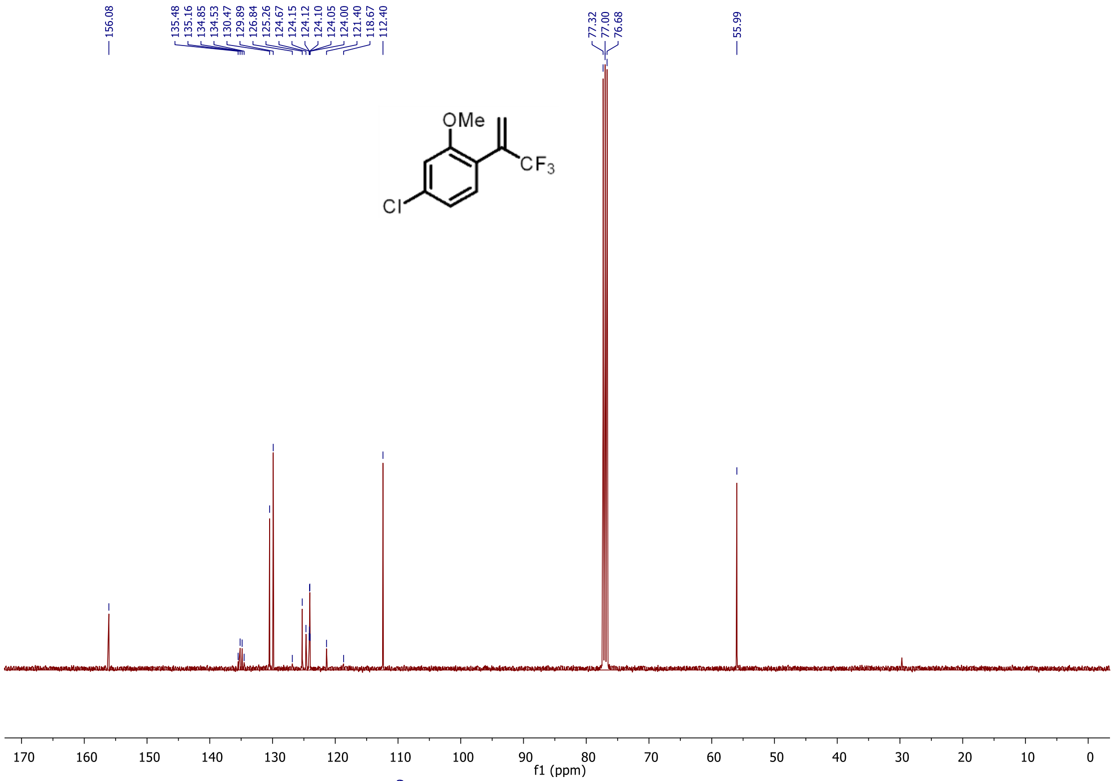
156.08
135.48
135.16
134.85
134.53
130.47
129.89
126.84
125.26
124.67
124.15
124.12
124.10
124.05
124.00
121.40
118.67
112.40
77.32
77.00
76.68
55.99
OMe
CF3
Cl
f1 (ppm)

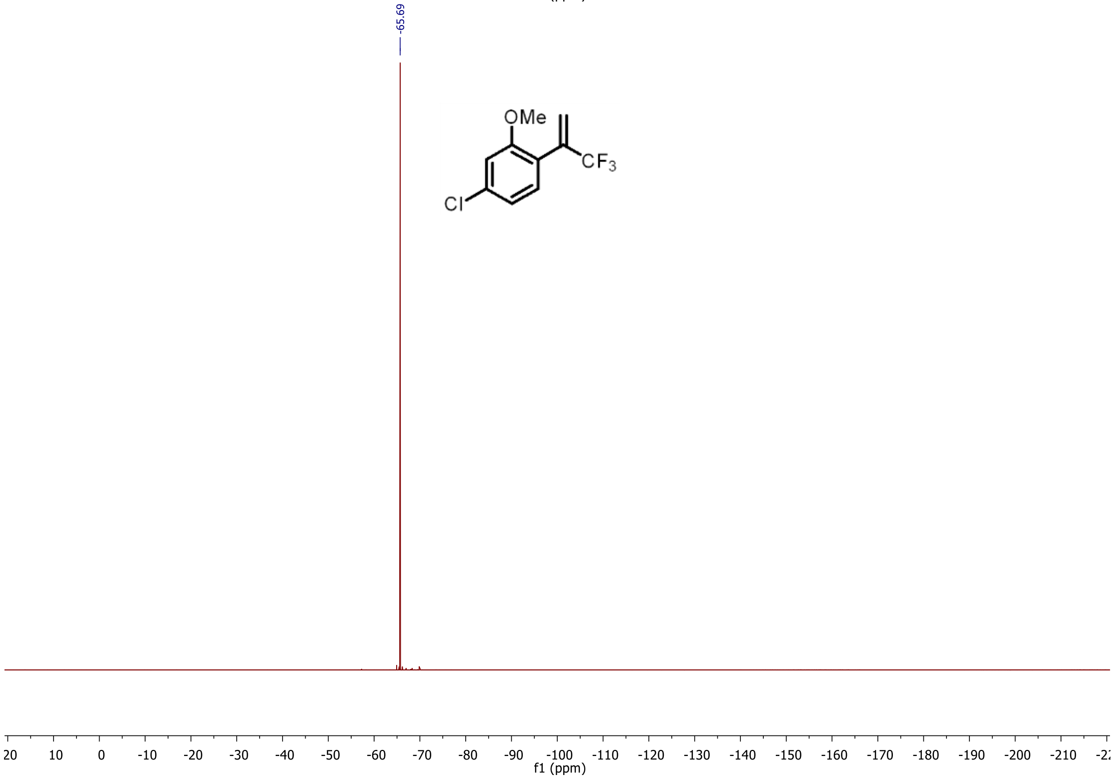
-65.69
OMe
CF3
Cl
f1 (ppm)

Compound **S17**

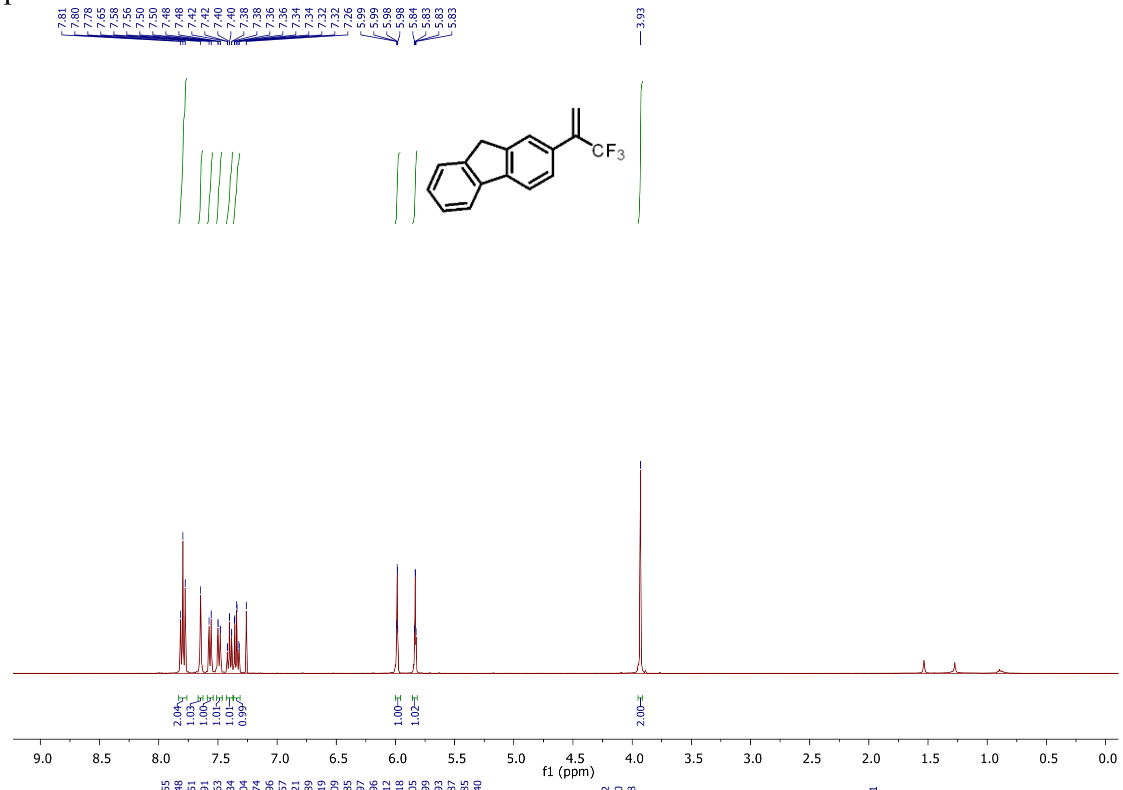

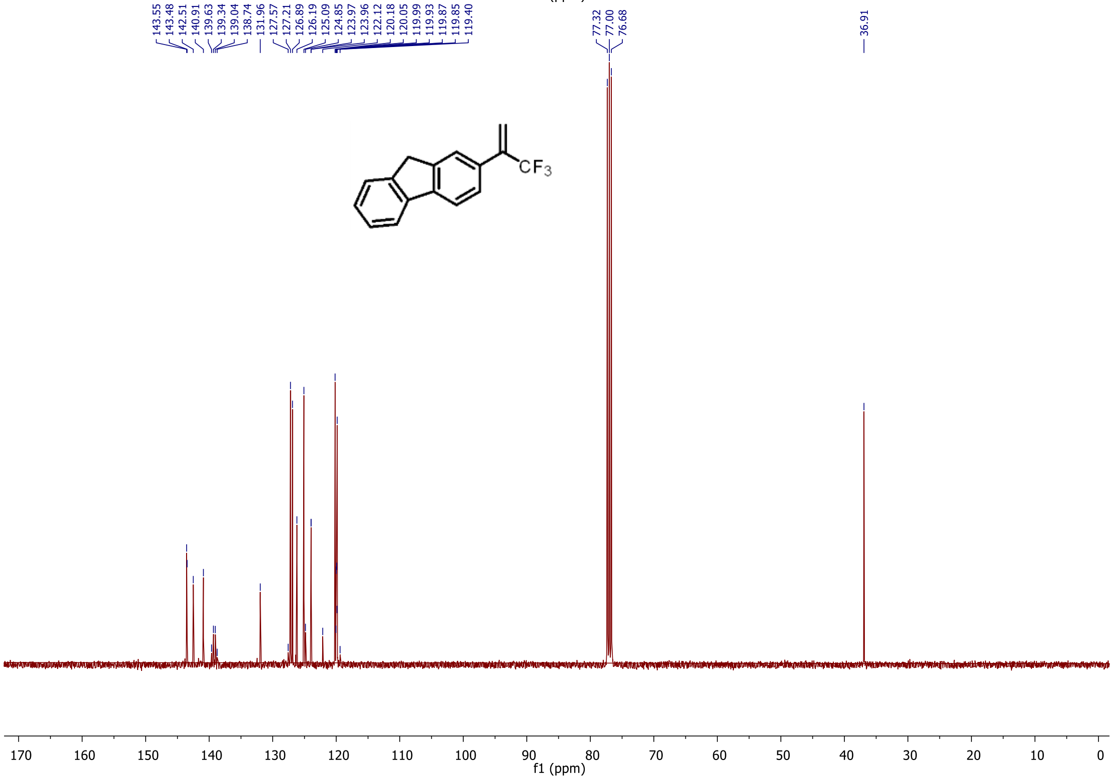

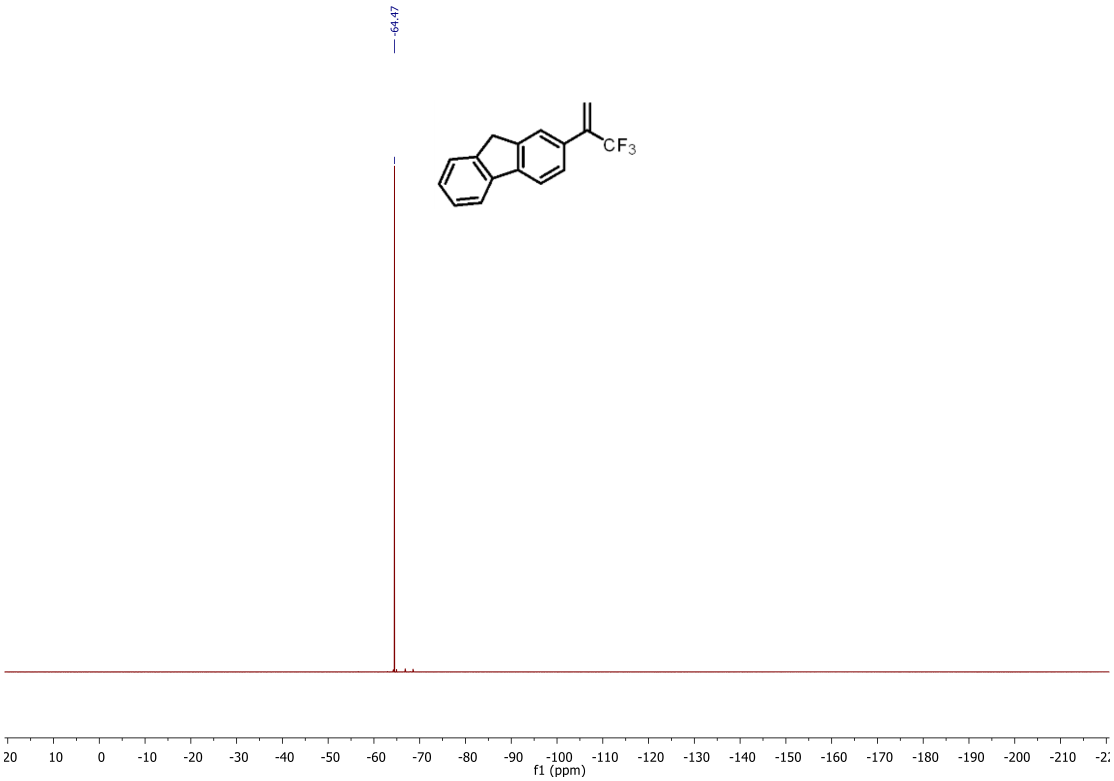

Compound **S27**

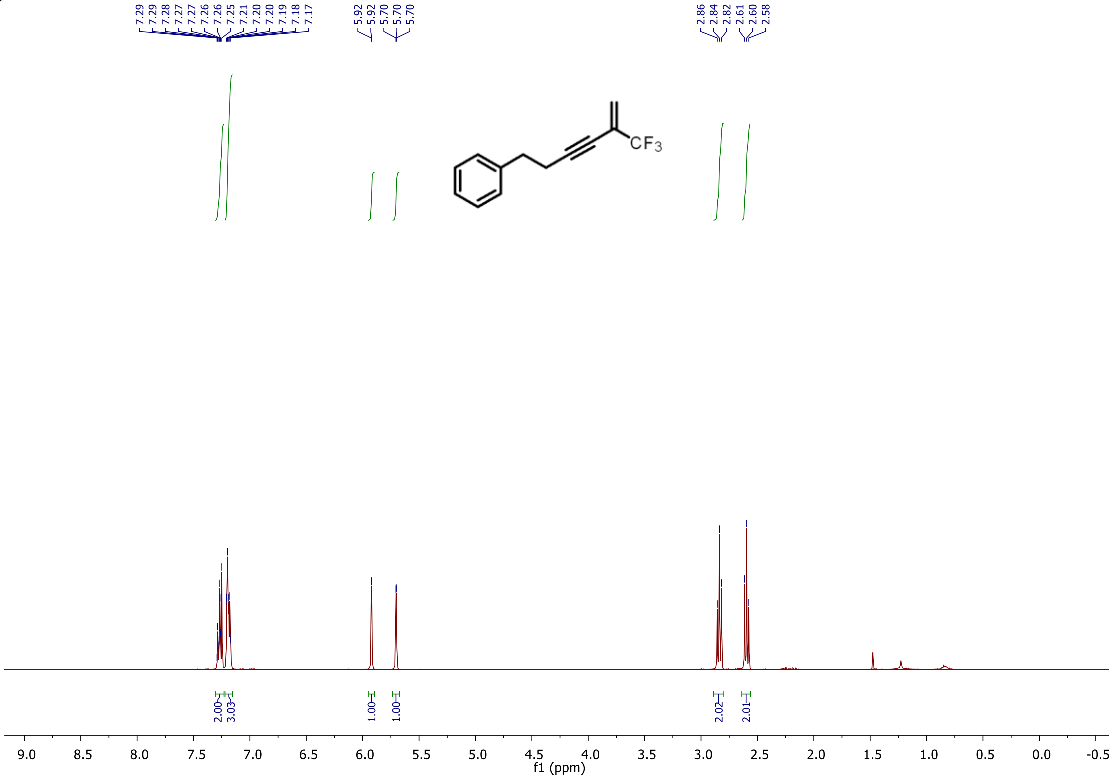

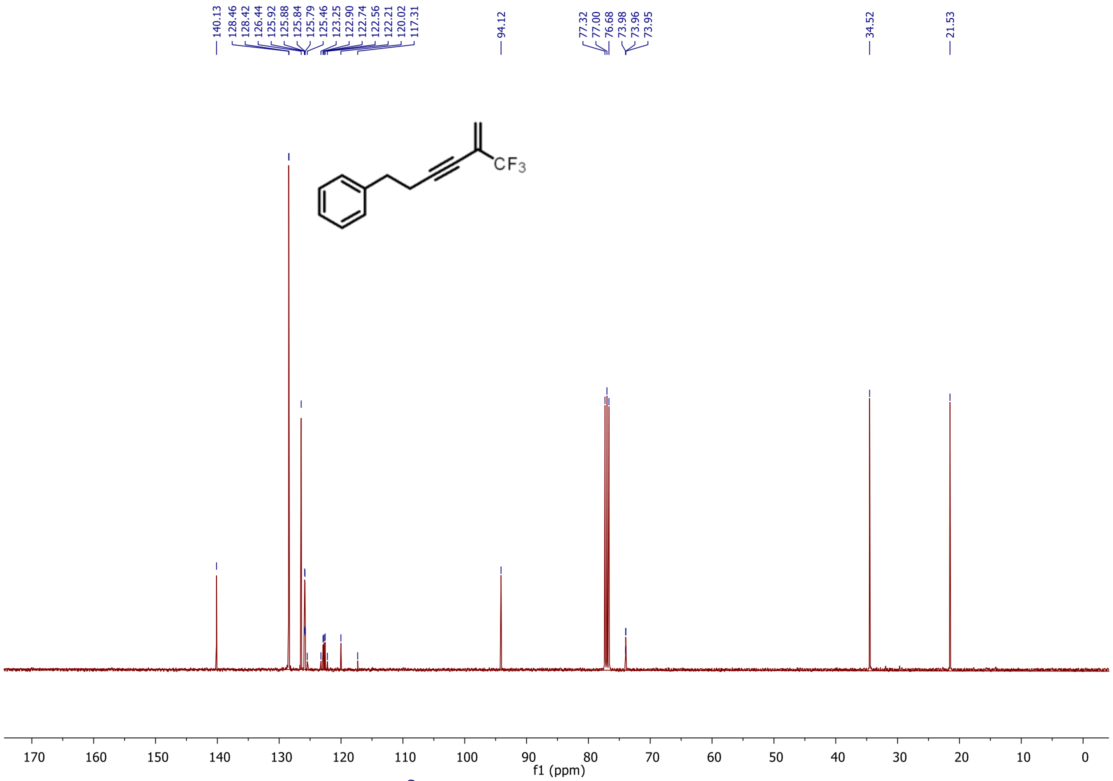

140.13
128.46
128.42
126.44
125.92
125.88
125.84
125.79
125.46
123.25
122.90
122.74
122.56
122.21
120.02
117.31
94.12
77.32
77.00
76.68
73.98
73.96
73.95
34.52
21.53
CF3
170 160 150 140 130 120 110 100 90 80 70 60 50 40 30 20 10 0
f1 (ppm)

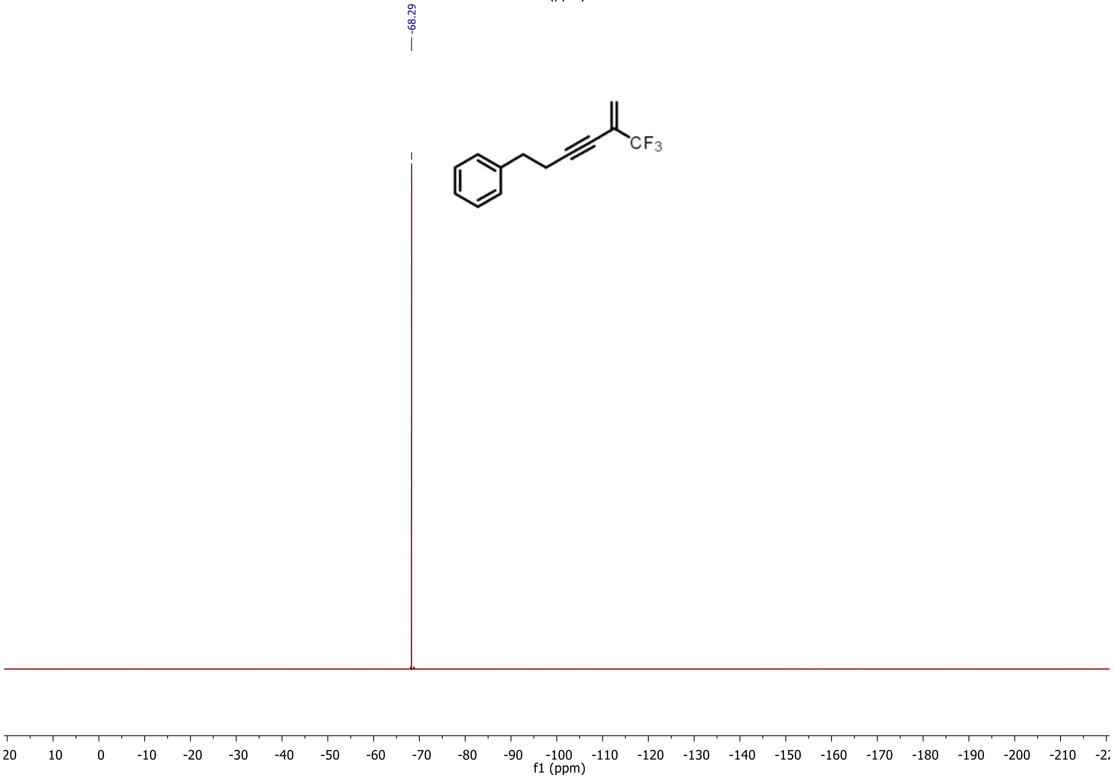

-68.29
CF3
20 10 0 -10 -20 -30 -40 -50 -60 -70 -80 -90 -100 -110 -120 -130 -140 -150 -160 -170 -180 -190 -200 -210
f1 (ppm)

Compound **4**

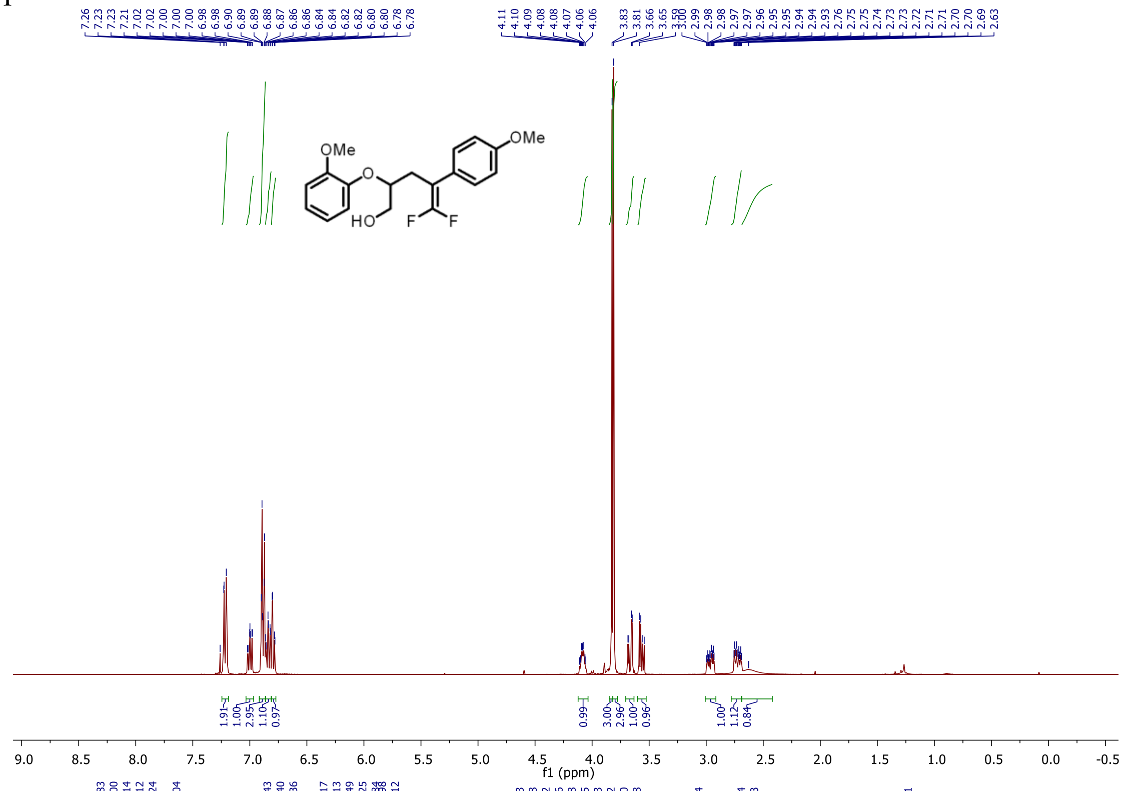

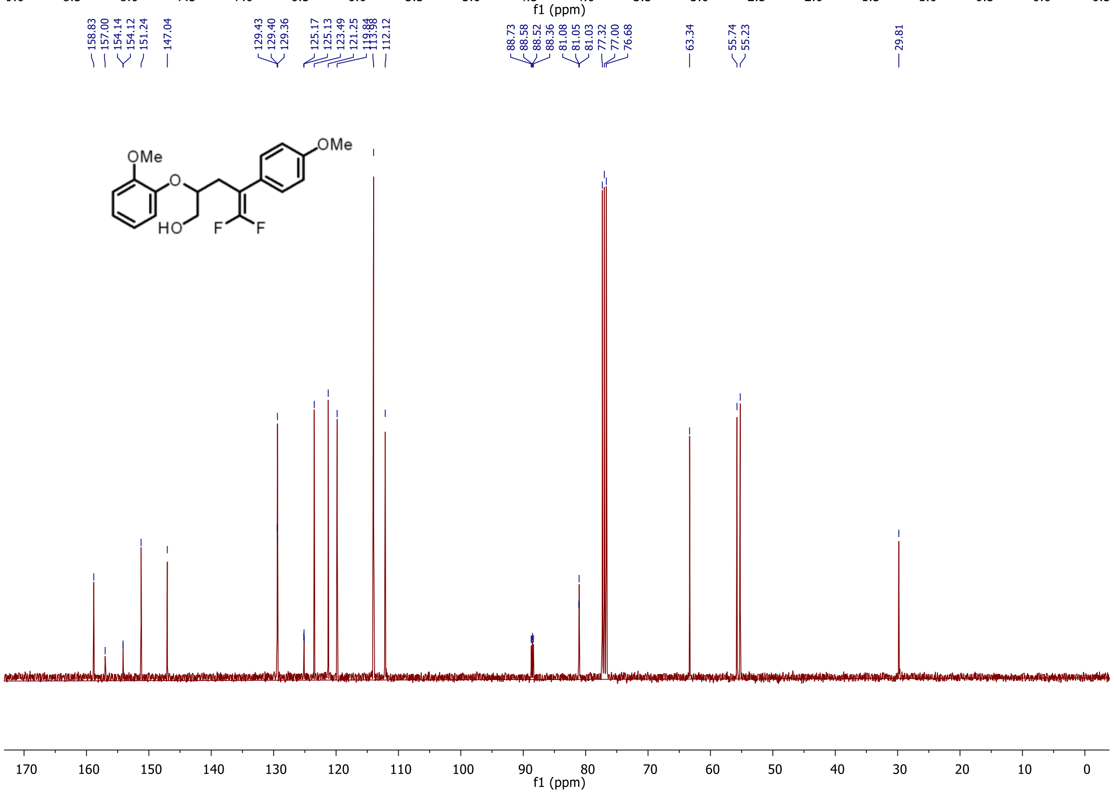

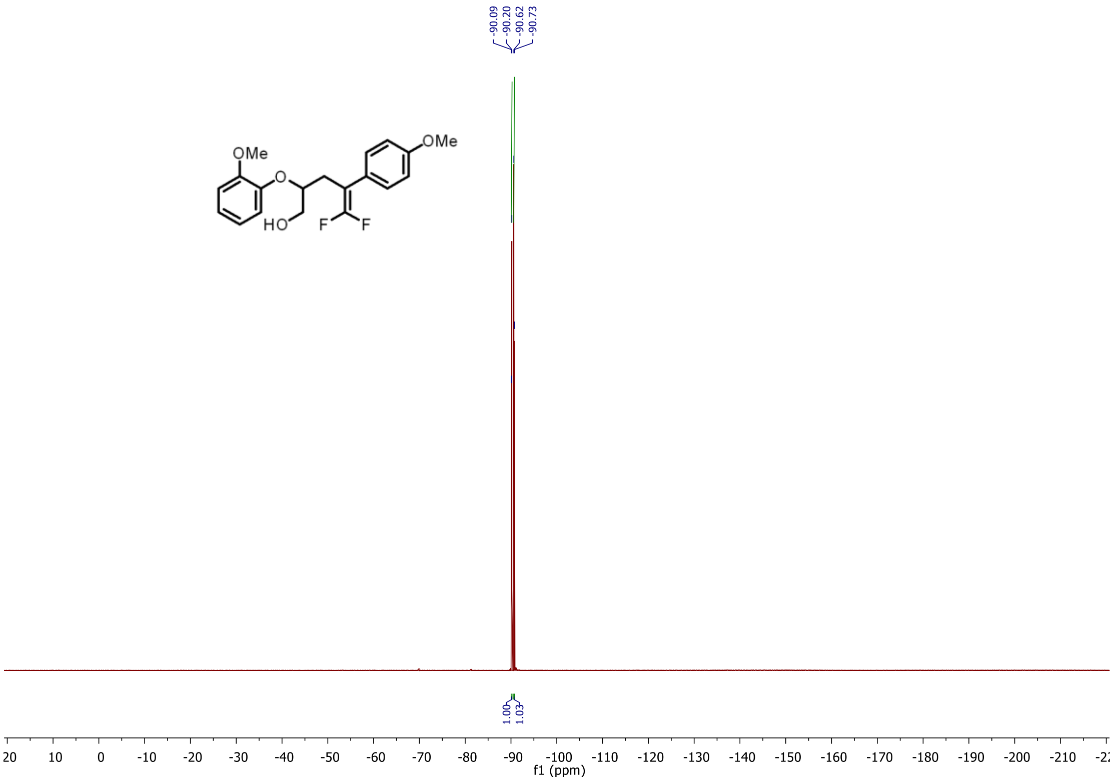
-90.09
-90.20
-90.62
-90.73
OMe
O
OMe
HO
F
F
1.00
1.03
f1 (ppm)

Compound **6**

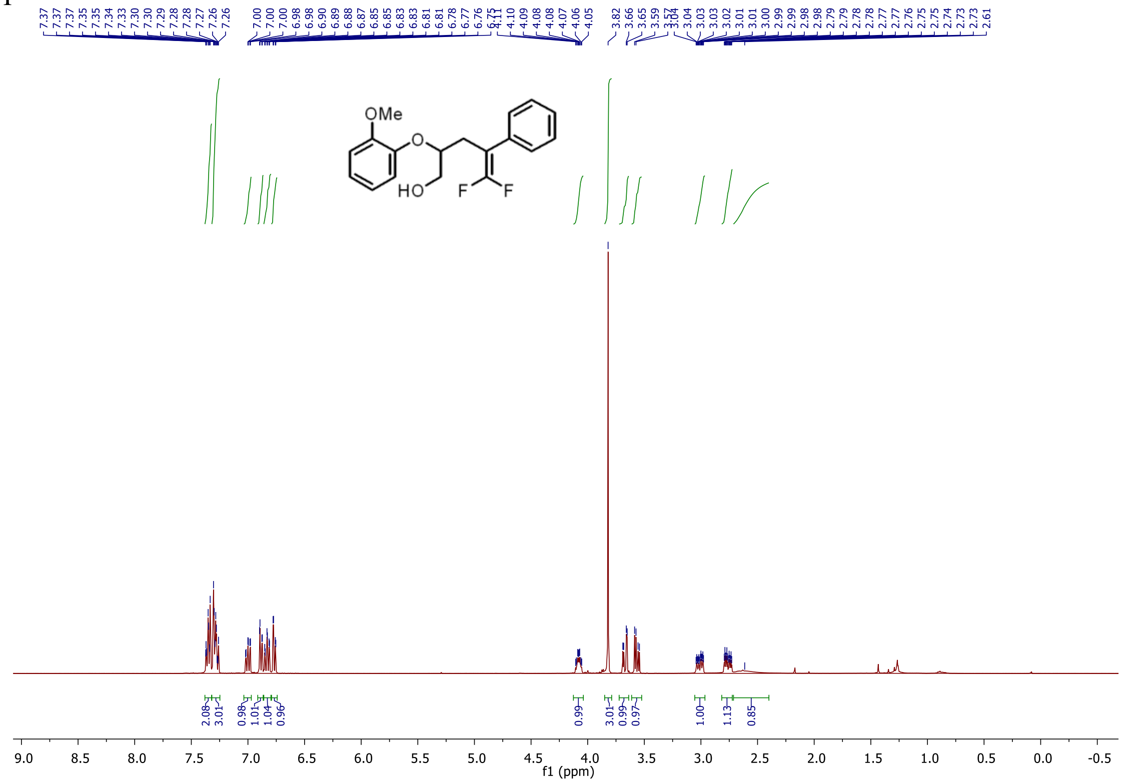
OMe
O
HO
F
F
2.08
3.01
0.98
1.01
1.04
0.96
0.99
3.01
0.99
0.97
1.00
1.13
0.85
f1 (ppm)

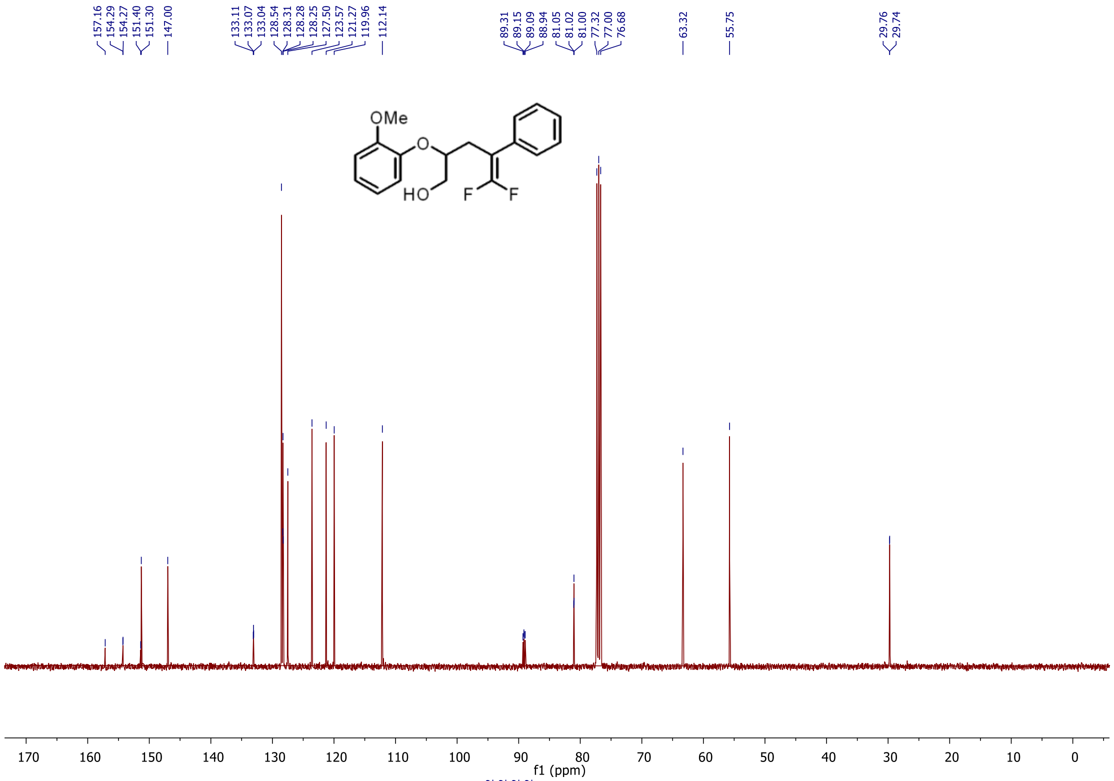
157.16
154.29
154.27
151.40
151.30
147.00
133.11
133.07
133.04
128.54
128.31
128.28
128.25
127.50
123.57
121.27
119.96
112.14
89.31
89.15
89.09
88.94
81.05
81.02
81.00
77.32
77.00
76.68
63.32
55.75
29.76
29.74
OMe
O
HO
F
F
170 160 150 140 130 120 110 100 90 80 70 60 50 40 30 20 10 0
f1 (ppm)

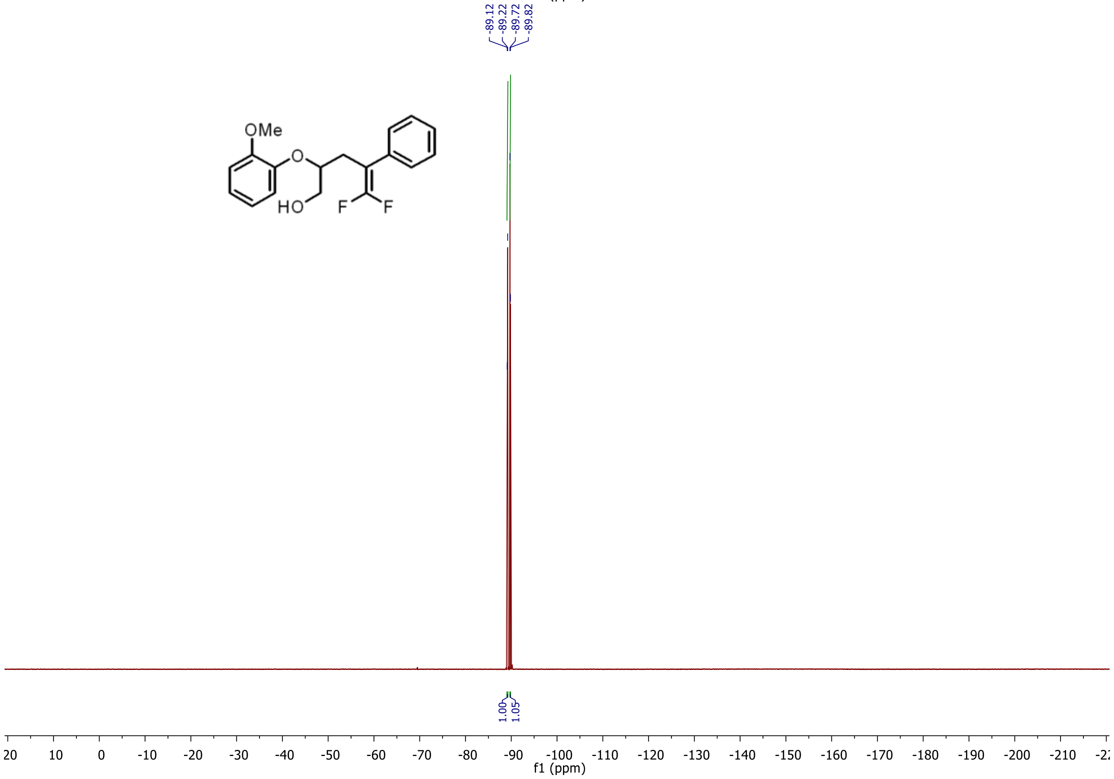
-89.12
-89.22
-89.72
-89.82
OMe
O
HO
F
F
1.00
1.05
20 10 0 -10 -20 -30 -40 -50 -60 -70 -80 -90 -100 -110 -120 -130 -140 -150 -160 -170 -180 -190 -200 -210
f1 (ppm)

Compound **7**

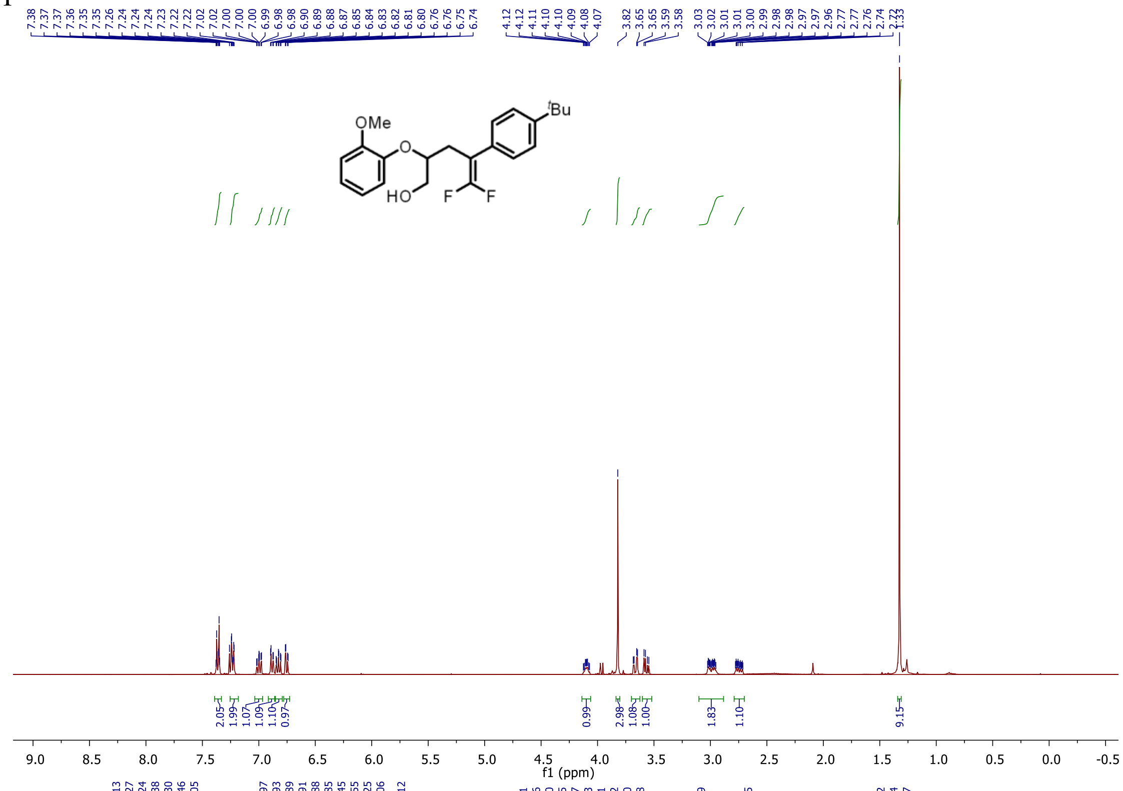

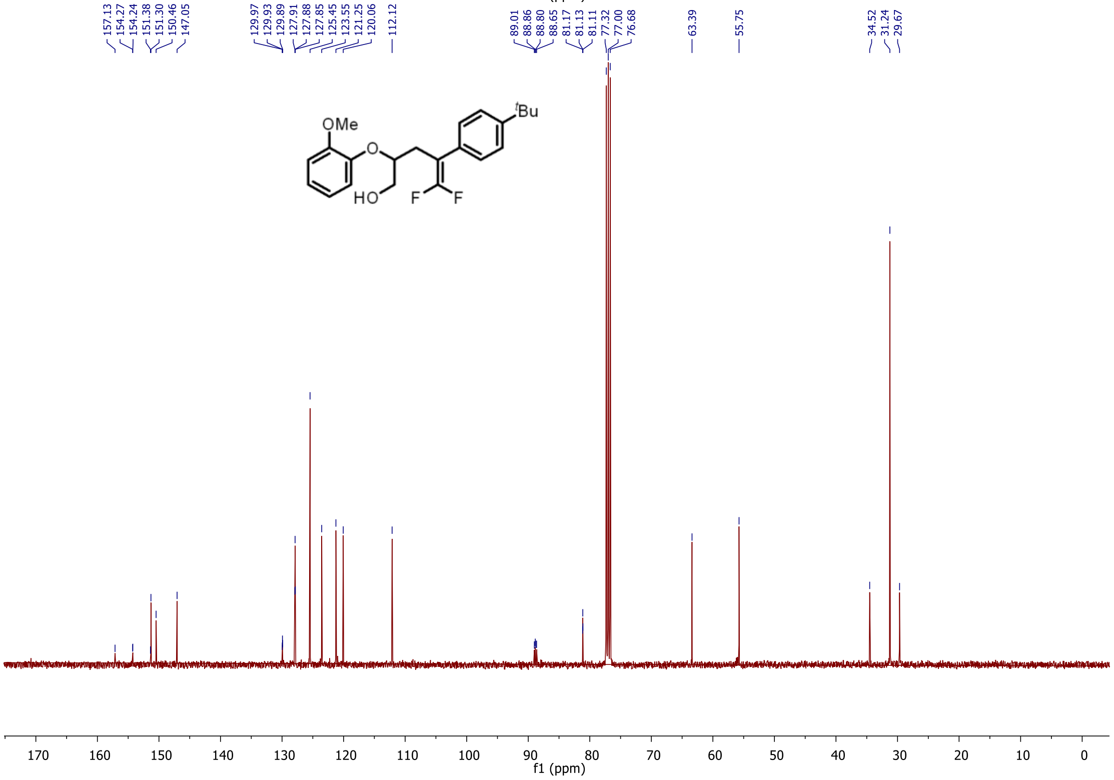

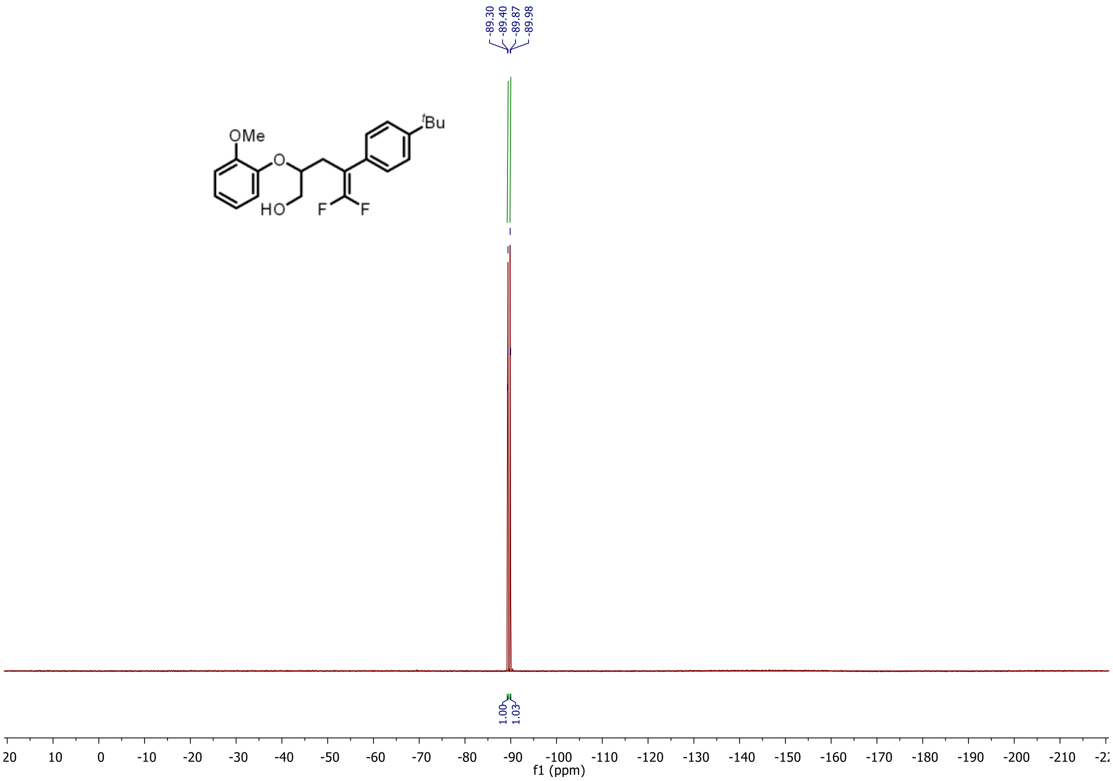
-89.30
-89.40
-89.87
-89.98
OMe
O
tBu
HO
F
F
1.00
1.03
20 10 0 -10 -20 -30 -40 -50 -60 -70 -80 -90 -100 -110 -120 -130 -140 -150 -160 -170 -180 -190 -200 -210
f1 (ppm)

Compound **8**

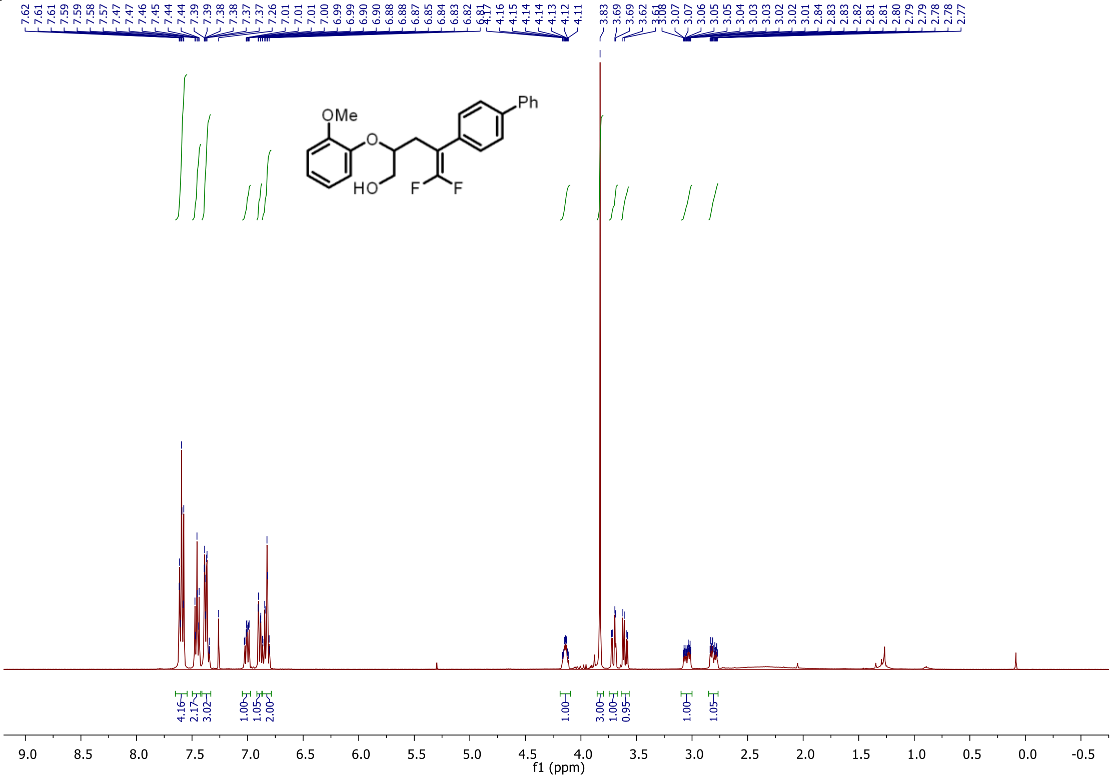
7.62 7.61 7.61 7.59 7.58 7.57 7.47 7.46 7.45 7.44 7.39 7.38 7.38 7.37 7.37 7.26 7.01 7.01 7.00 6.99 6.99 6.90 6.90 6.88 6.88 6.87 6.85 6.84 6.83 6.82 6.81 4.17 4.16 4.15 4.14 4.14 4.13 4.12 4.11 3.83 3.69 3.69 3.62 3.61 3.08 3.07 3.07 3.06 3.05 3.04 3.03 3.03 3.02 3.02 3.01 2.84 2.83 2.83 2.82 2.81 2.81 2.80 2.79 2.79 2.78 2.78 2.77
OMe
O
Ph
HO
F
F
4.16
2.17
3.02
1.00
1.05
2.00
1.00
3.00
1.00
0.95
1.00
1.05
9.0 8.5 8.0 7.5 7.0 6.5 6.0 5.5 5.0 4.5 4.0 3.5 3.0 2.5 2.0 1.5 1.0 0.5 0.0 -0.5
f1 (ppm)

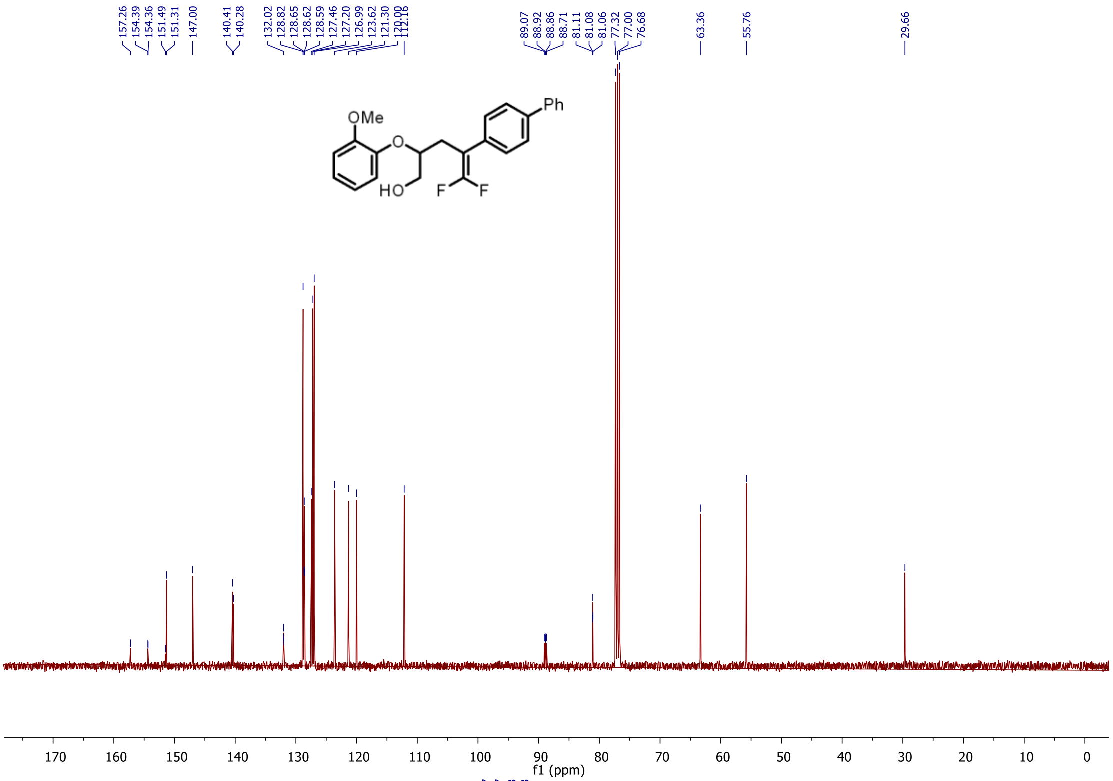

157.26
154.39
154.36
151.49
151.31
147.00
140.41
140.28
132.02
128.82
128.65
128.62
128.59
127.46
127.20
126.99
123.62
121.30
120.00
112.16
89.07
88.92
88.86
88.71
81.11
81.08
81.06
77.32
77.00
76.68
63.36
55.76
29.66
OMe
O
Ph
HO
F
F
f1 (ppm)

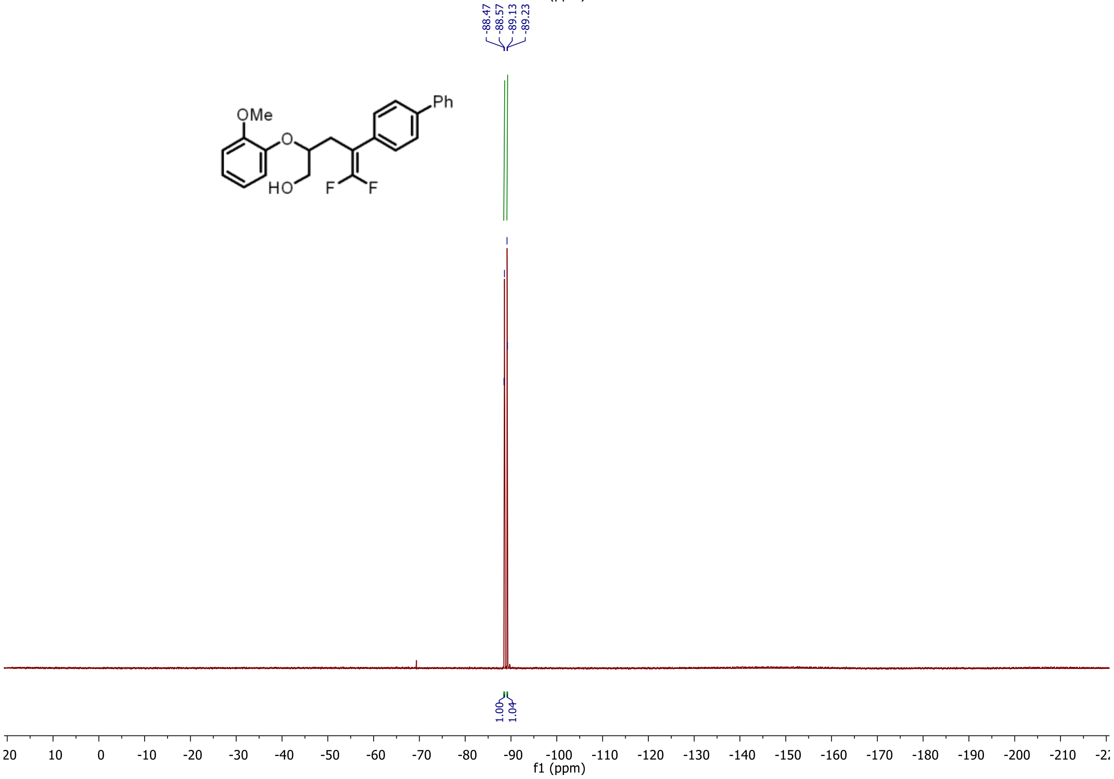

-88.47
-88.57
-89.13
-89.23
OMe
O
Ph
HO
F
F
1.00
1.04
f1 (ppm)

Compound **9**

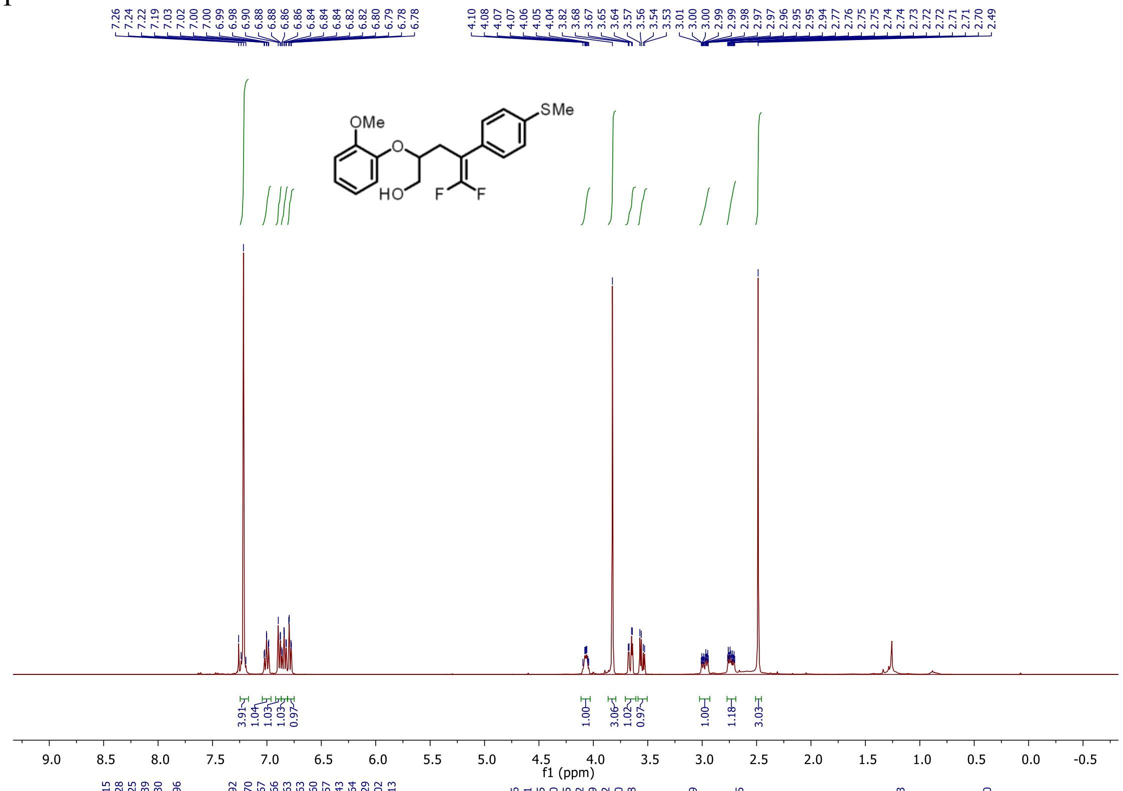

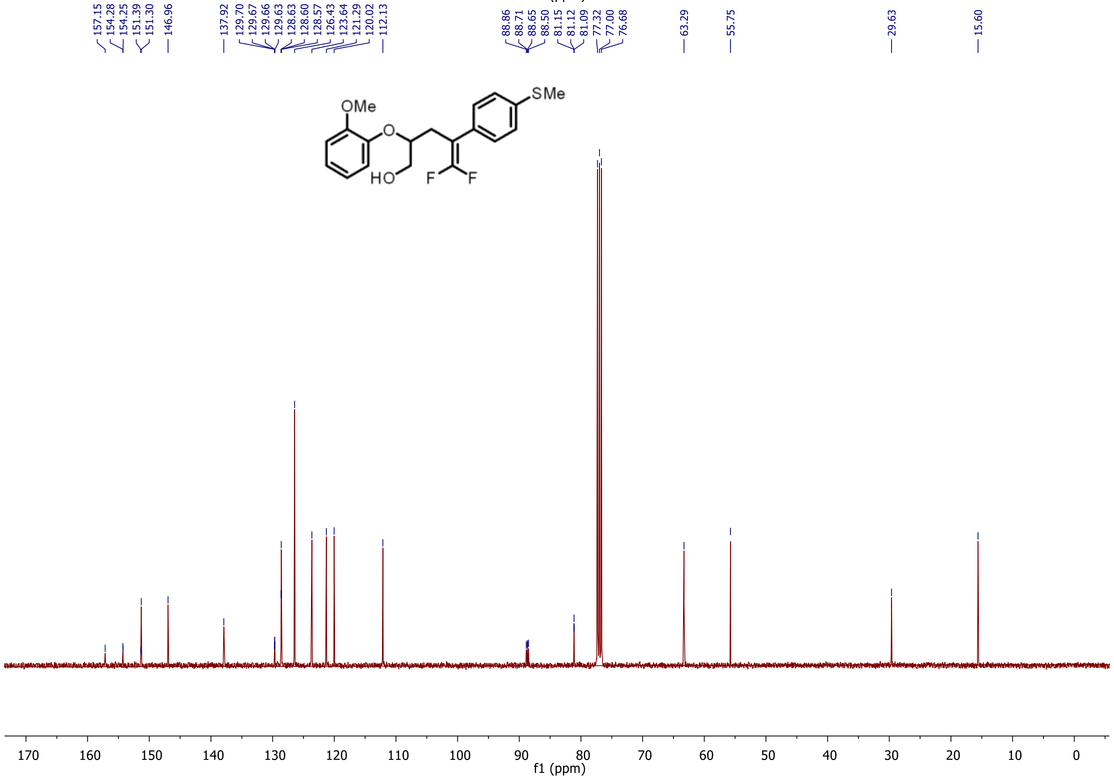

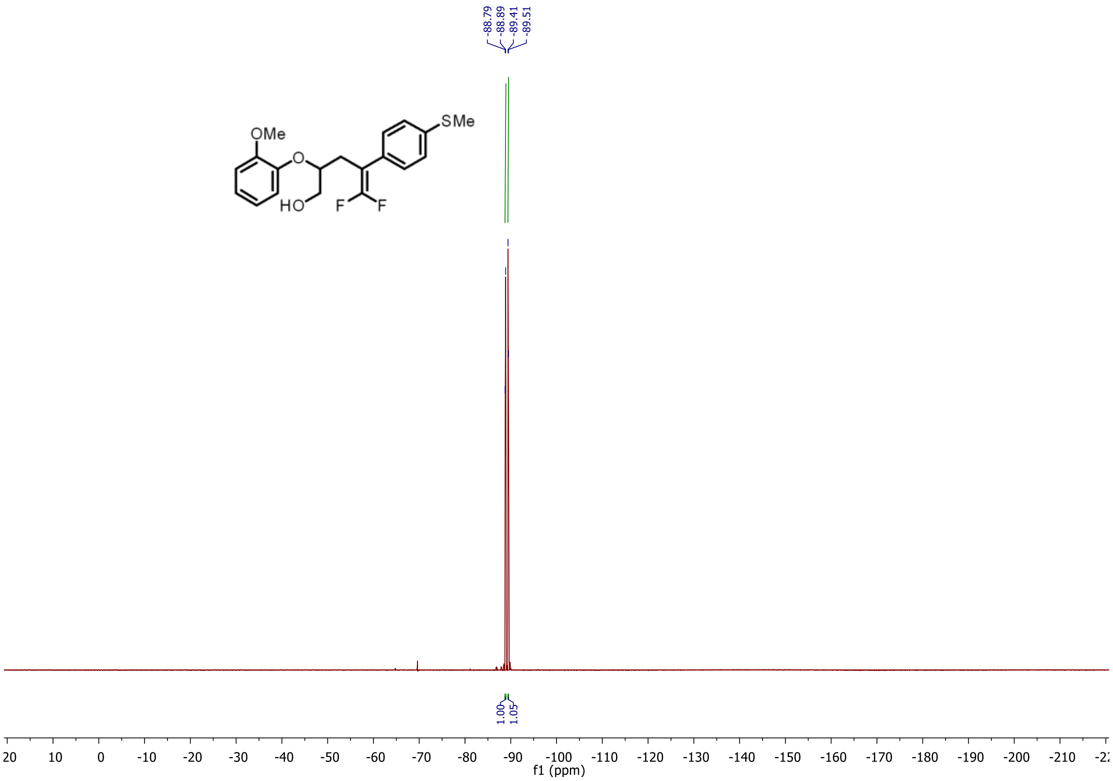

Compound **10**

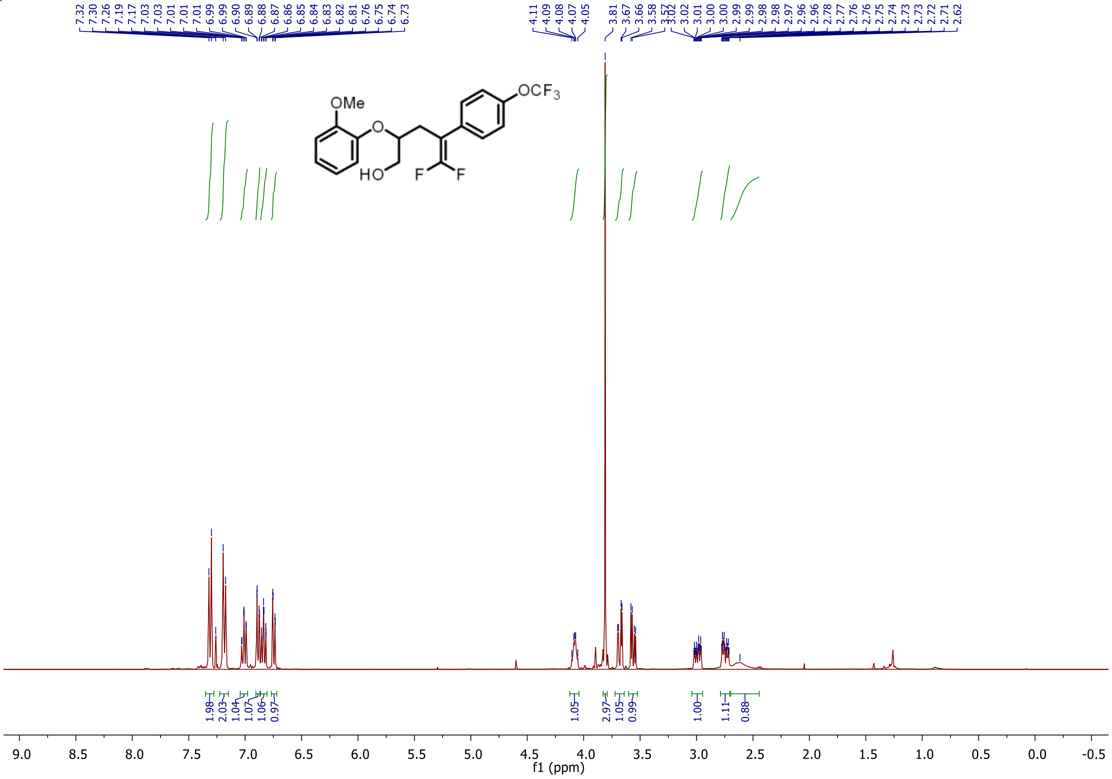

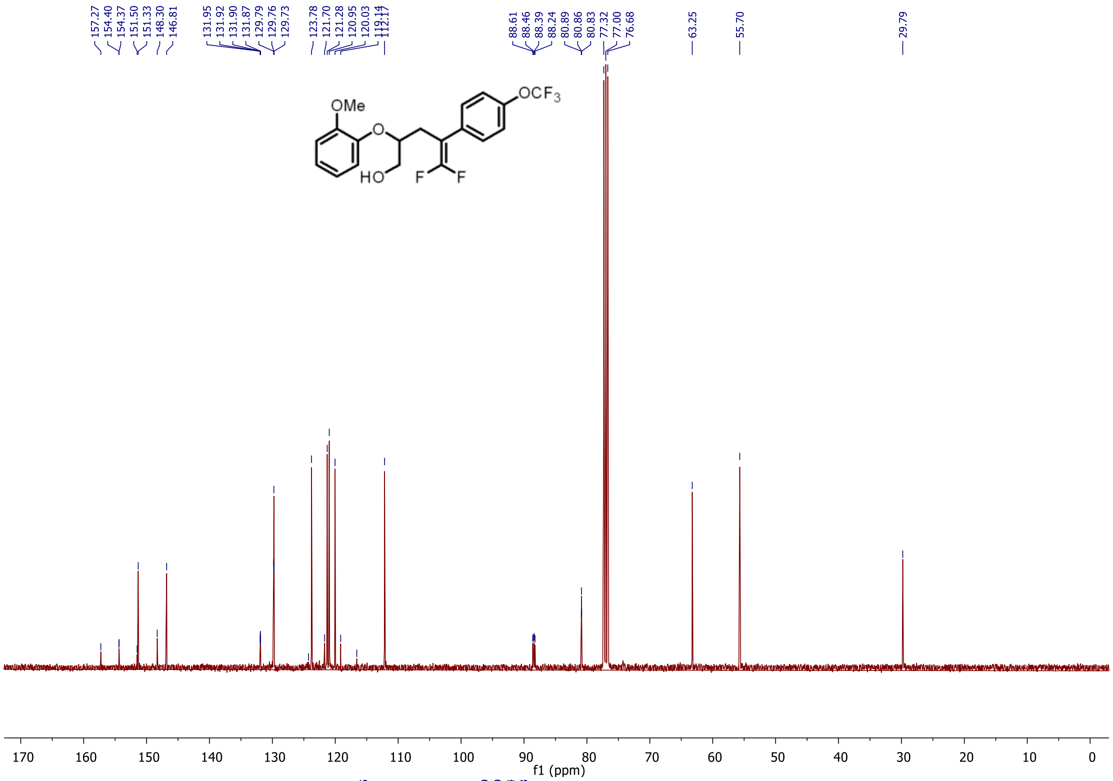

157.27
154.40
154.37
151.50
151.33
148.30
146.81
131.95
131.92
131.90
131.87
129.79
129.76
129.73
123.78
121.70
121.28
120.95
120.03
88.61
88.46
88.39
88.24
80.89
80.86
80.83
77.32
77.00
76.68
63.25
55.70
29.79
OMe
O
OCF$_3$
HO
F
F
f1 (ppm)

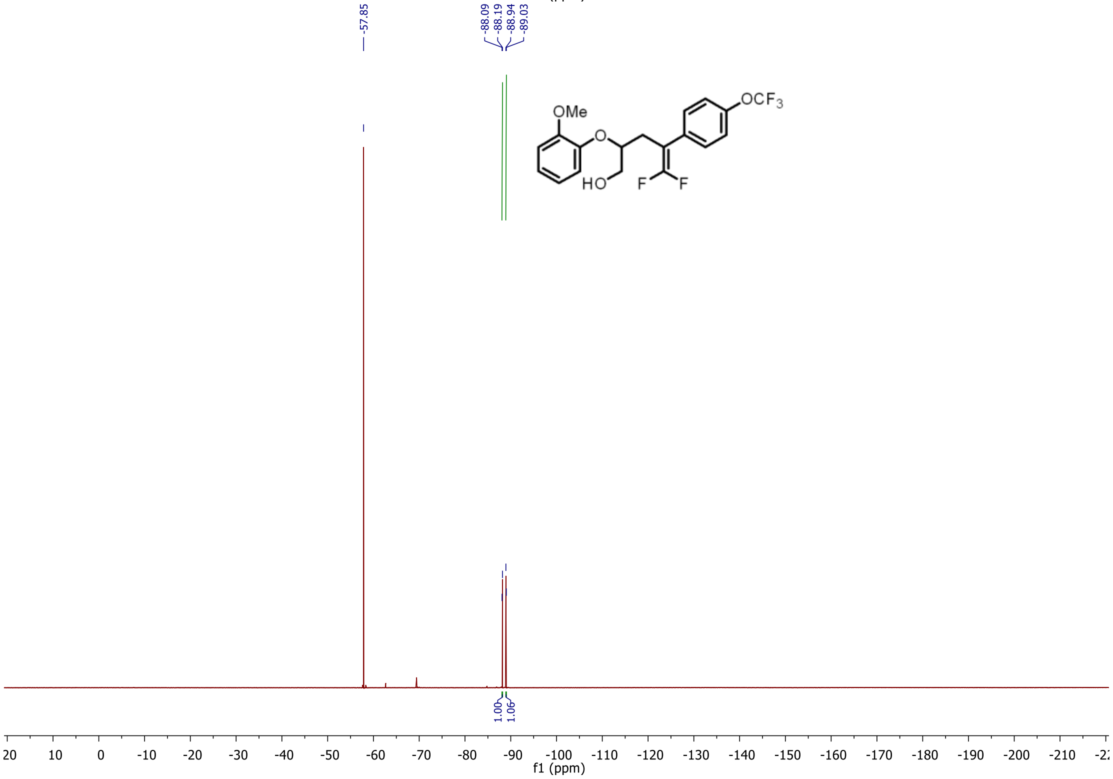

-57.85
-88.09
-88.19
-88.94
-89.03
OMe
O
OCF$_3$
HO
F
F
1.00
1.06
f1 (ppm)

Compound **11**

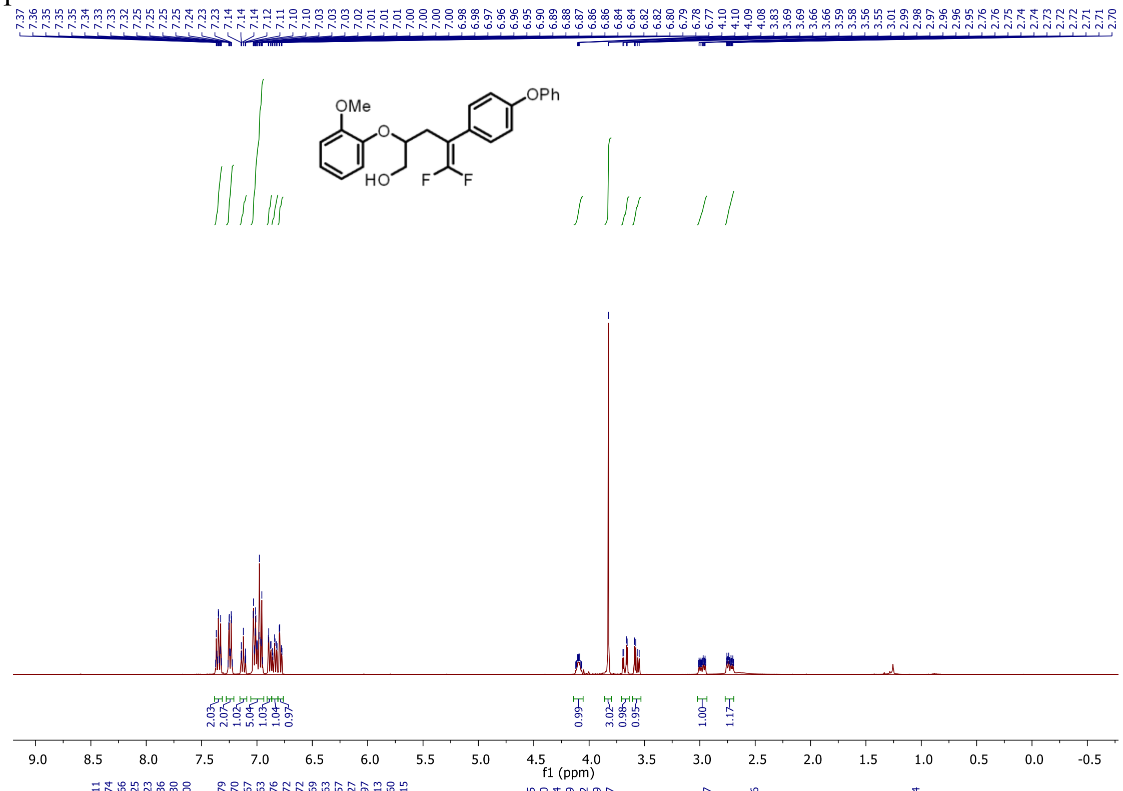

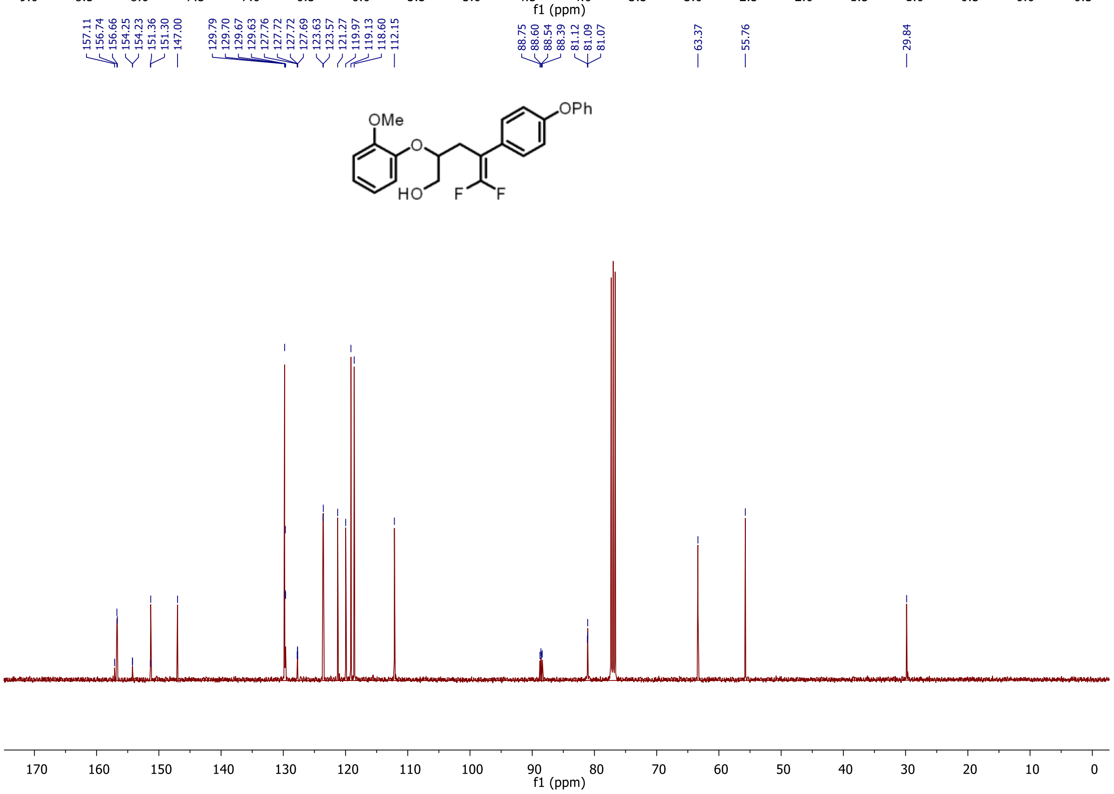

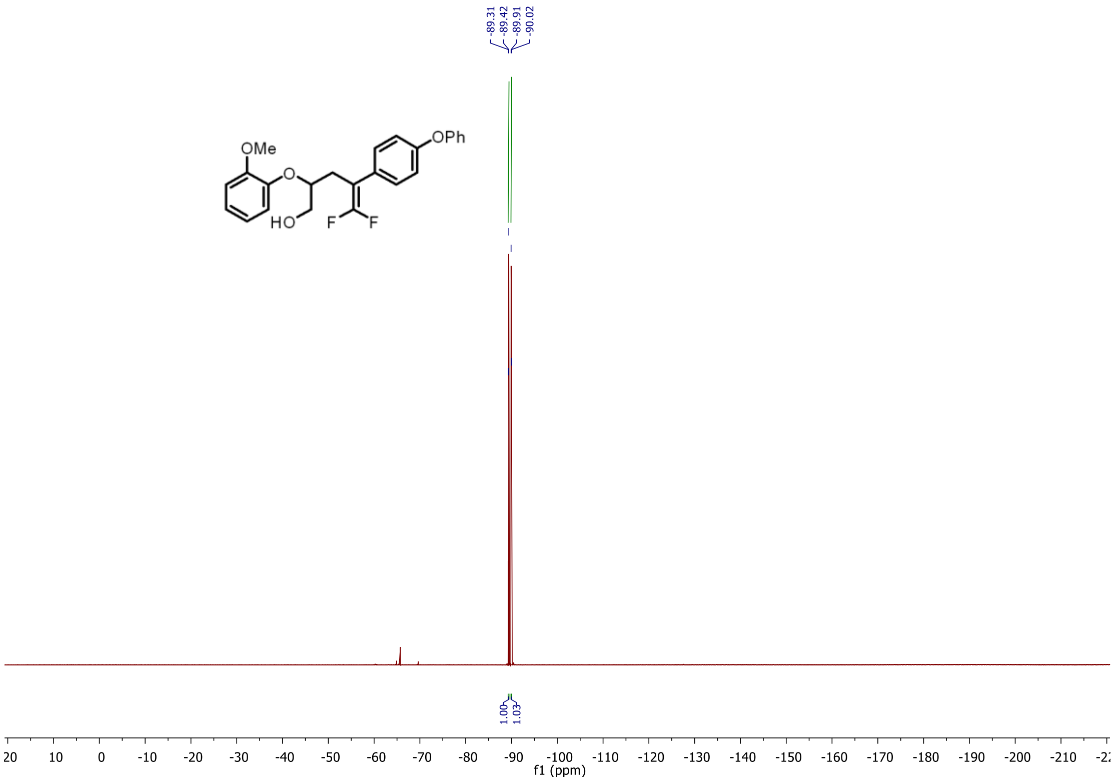

Compound **12**

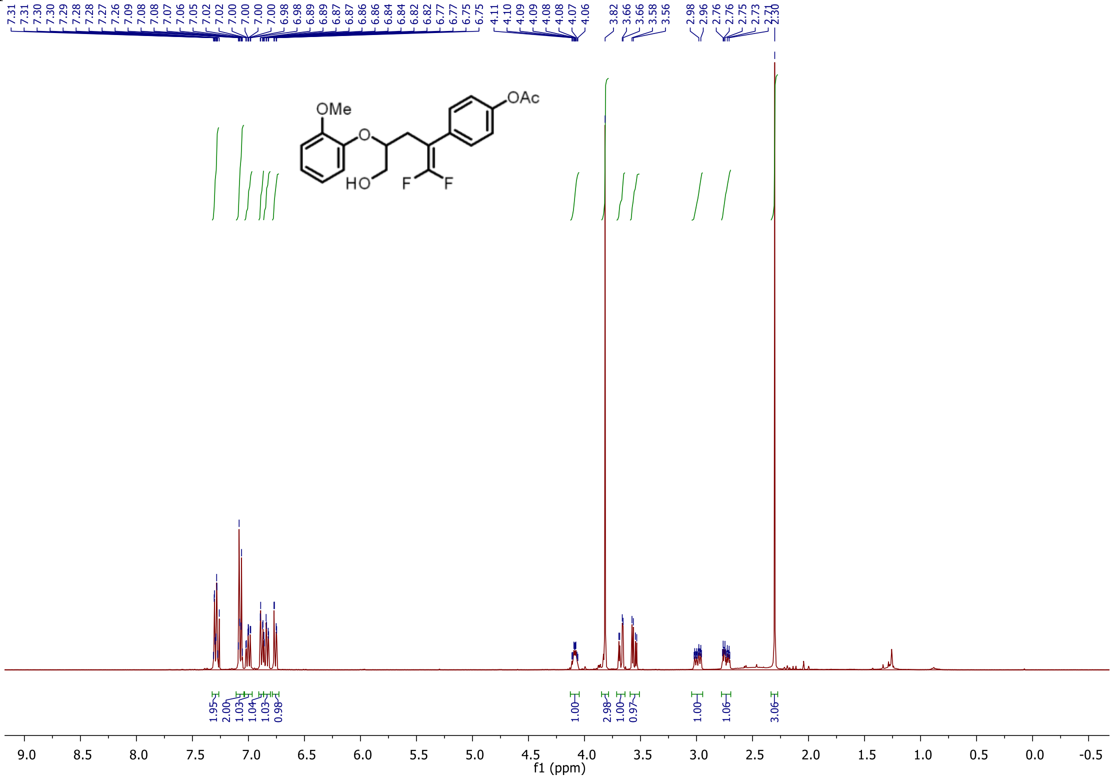

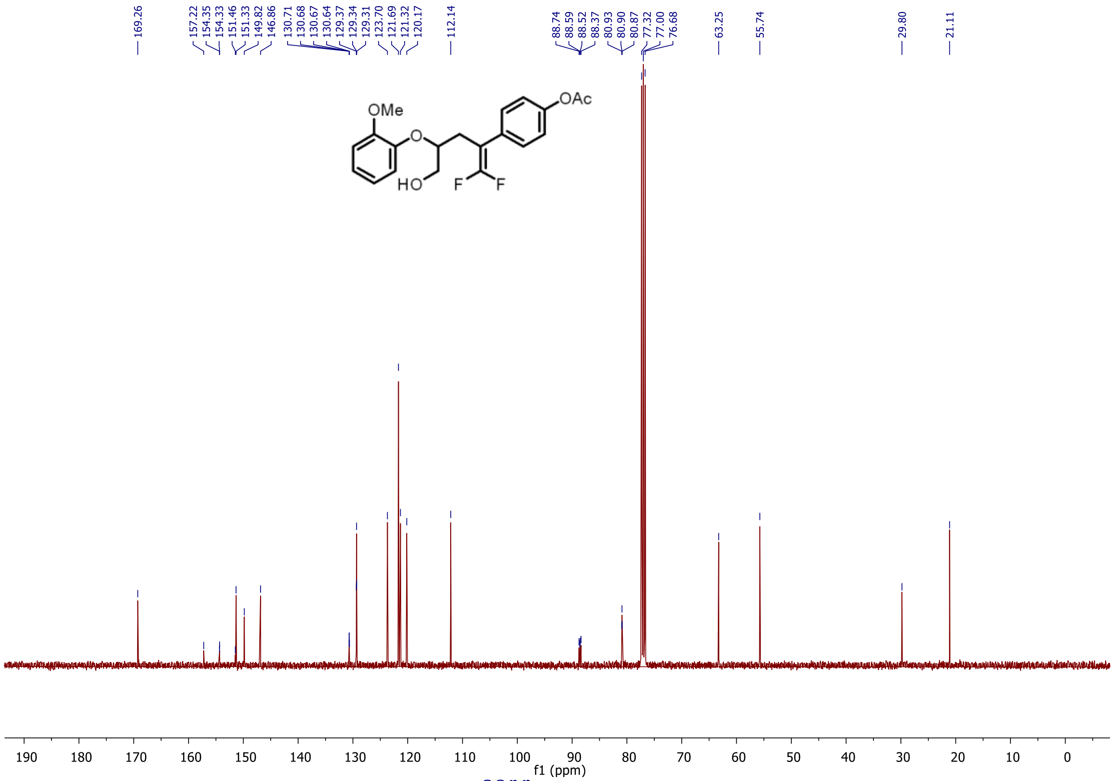

169.26
157.22
154.35
154.33
151.46
151.33
149.82
146.86
130.71
130.68
130.67
130.64
129.37
129.34
129.31
123.70
121.69
121.32
120.17
112.14
88.74
88.59
88.52
88.37
80.93
80.90
80.87
77.32
77.00
76.68
63.25
55.74
29.80
21.11
OMe
O
OAc
HO
F
F
190 180 170 160 150 140 130 120 110 100 90 80 70 60 50 40 30 20 10 0
f1 (ppm)

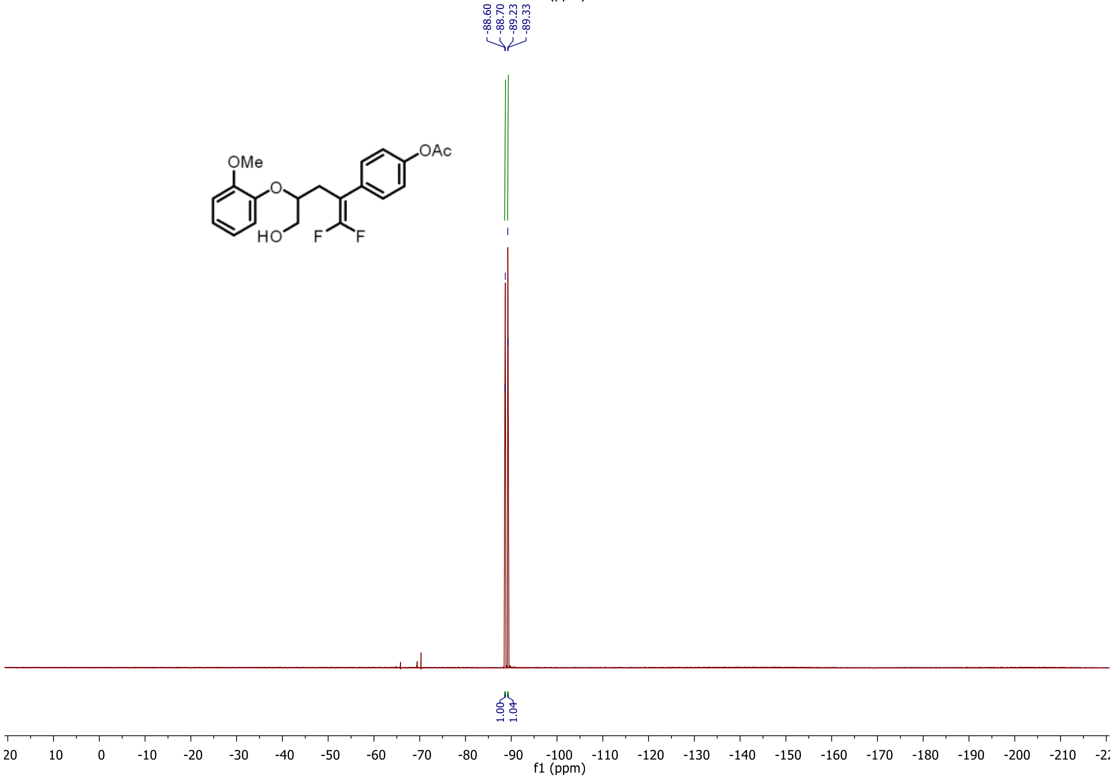

-88.60
-88.70
-89.23
-89.33
OMe
O
OAc
HO
F
F
1.00
1.04
20 10 0 -10 -20 -30 -40 -50 -60 -70 -80 -90 -100 -110 -120 -130 -140 -150 -160 -170 -180 -190 -200 -210
f1 (ppm)

Compound **13**

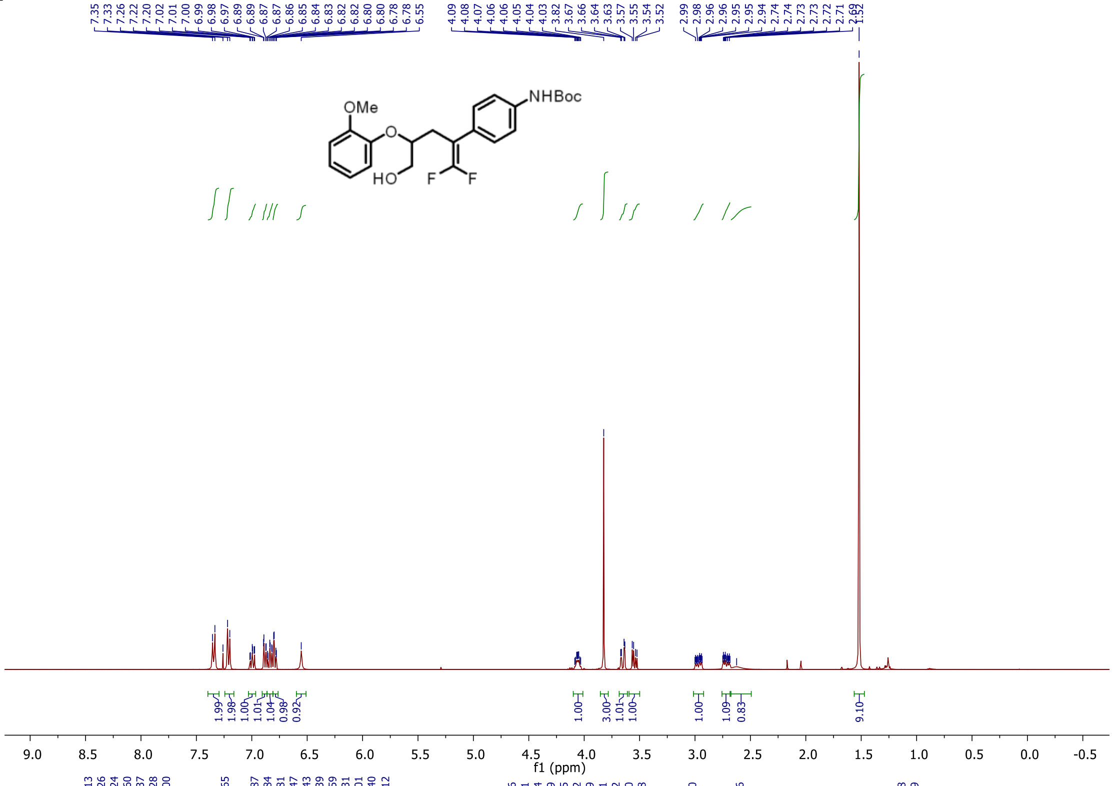

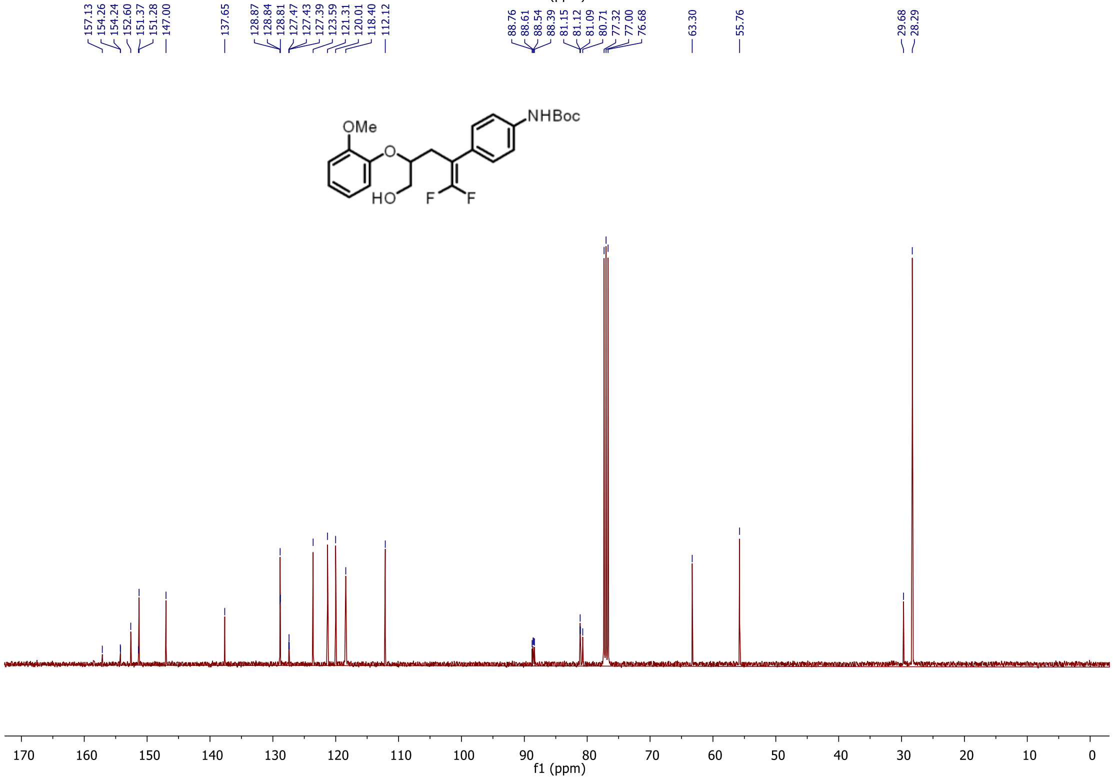

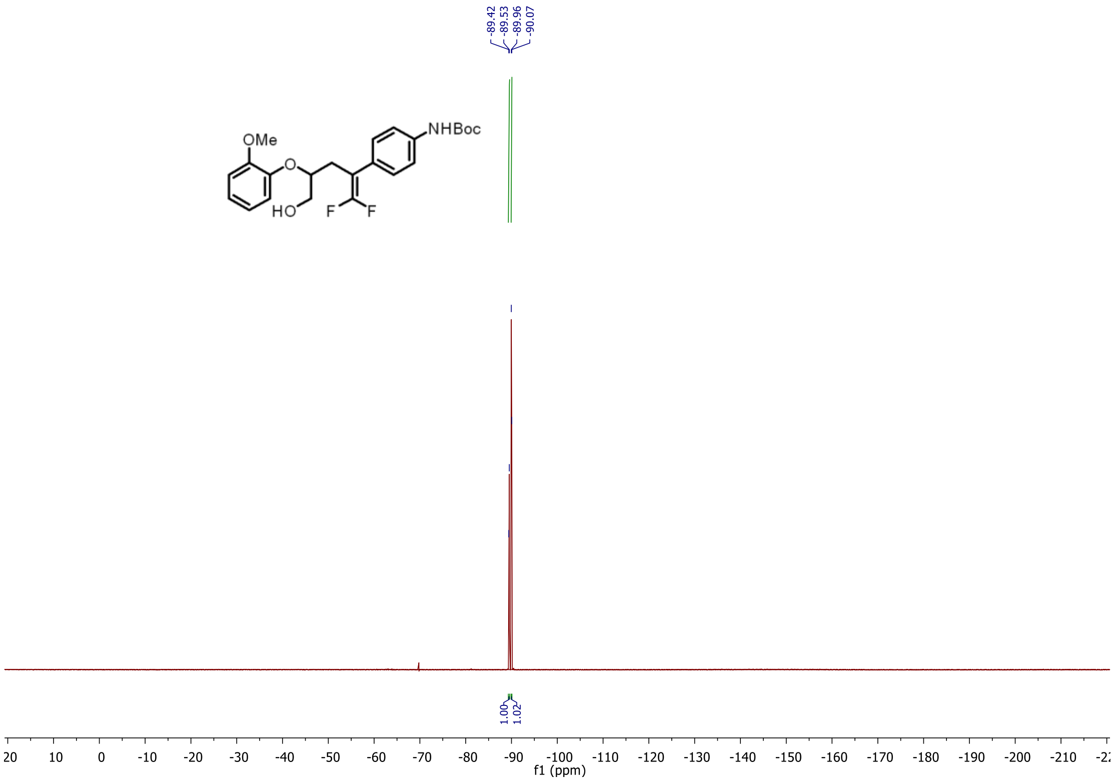

-89.42
-89.53
-89.96
-90.07
OMe
NHBoc
HO
F
F
1.00
1.02
f1 (ppm)

Compound **14**

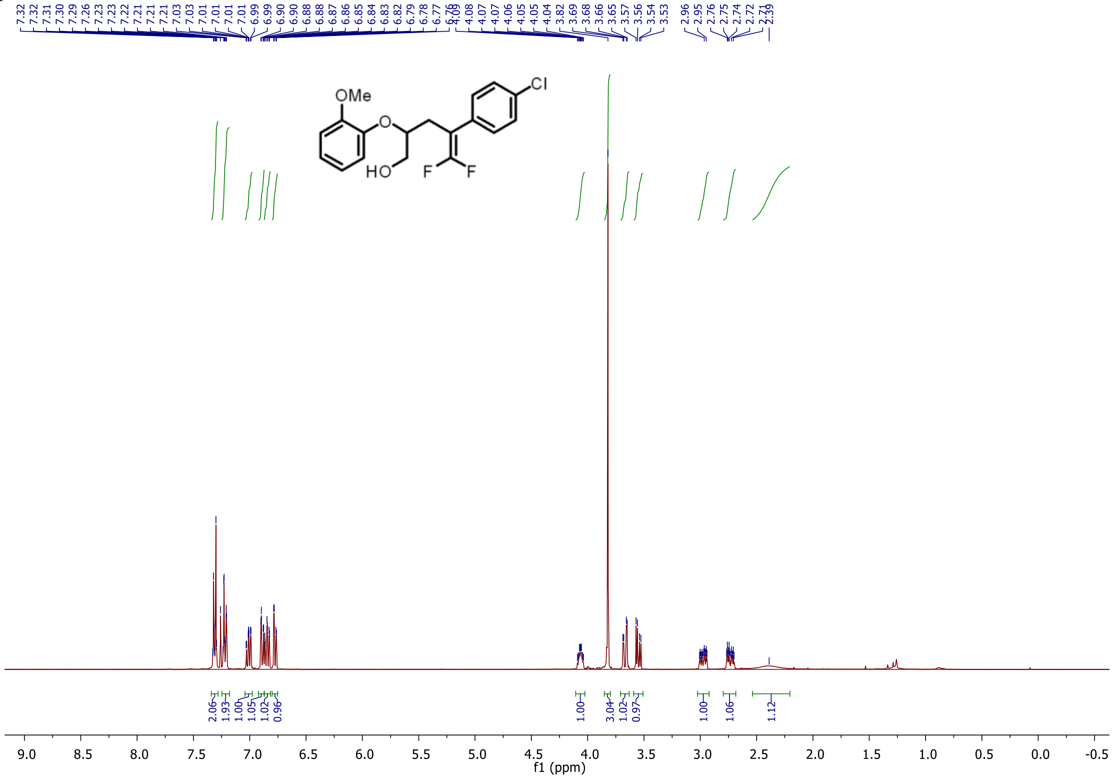

OMe
Cl
HO
F
F
2.06
1.93
1.00
1.05
1.02
0.96
1.00
3.04
1.02
0.97
1.00
1.06
1.12
f1 (ppm)

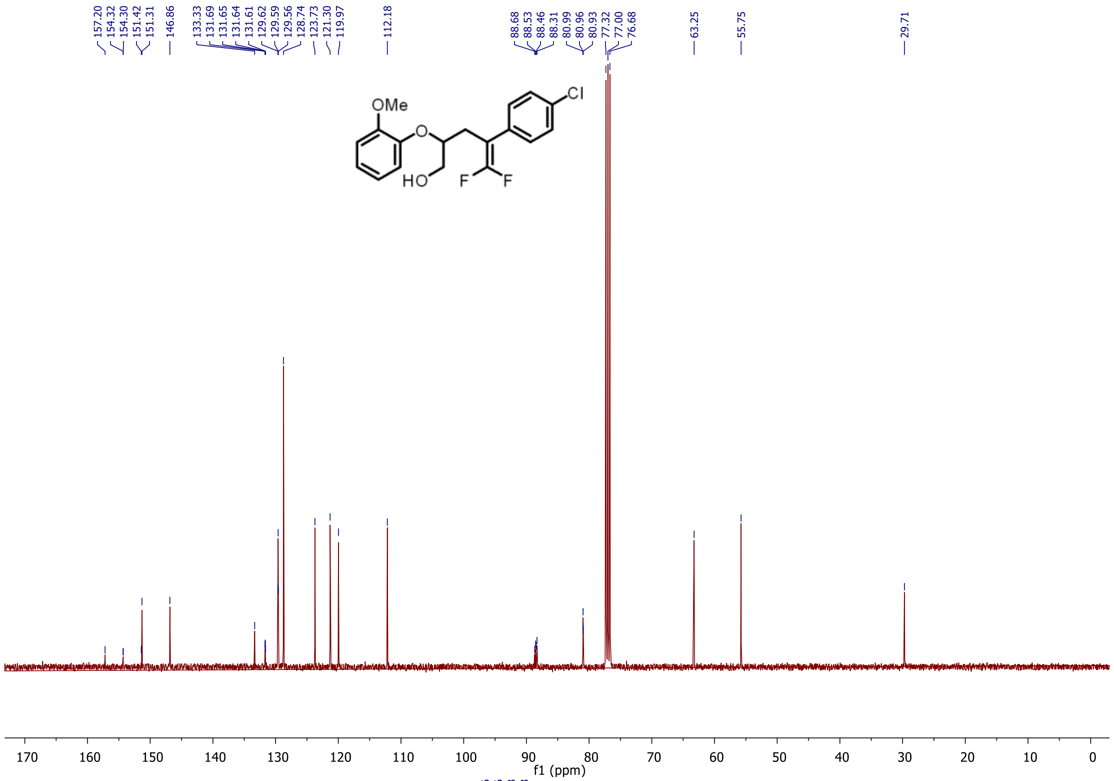
157.20
154.32
154.30
151.42
151.31
146.86
133.33
131.69
131.65
131.64
131.61
129.62
129.59
129.56
128.74
123.73
121.30
119.97
112.18
88.68
88.53
88.46
88.31
80.99
80.96
80.93
77.32
77.00
76.68
63.25
55.75
29.71
OMe
O
Cl
HO
F
F
f1 (ppm)

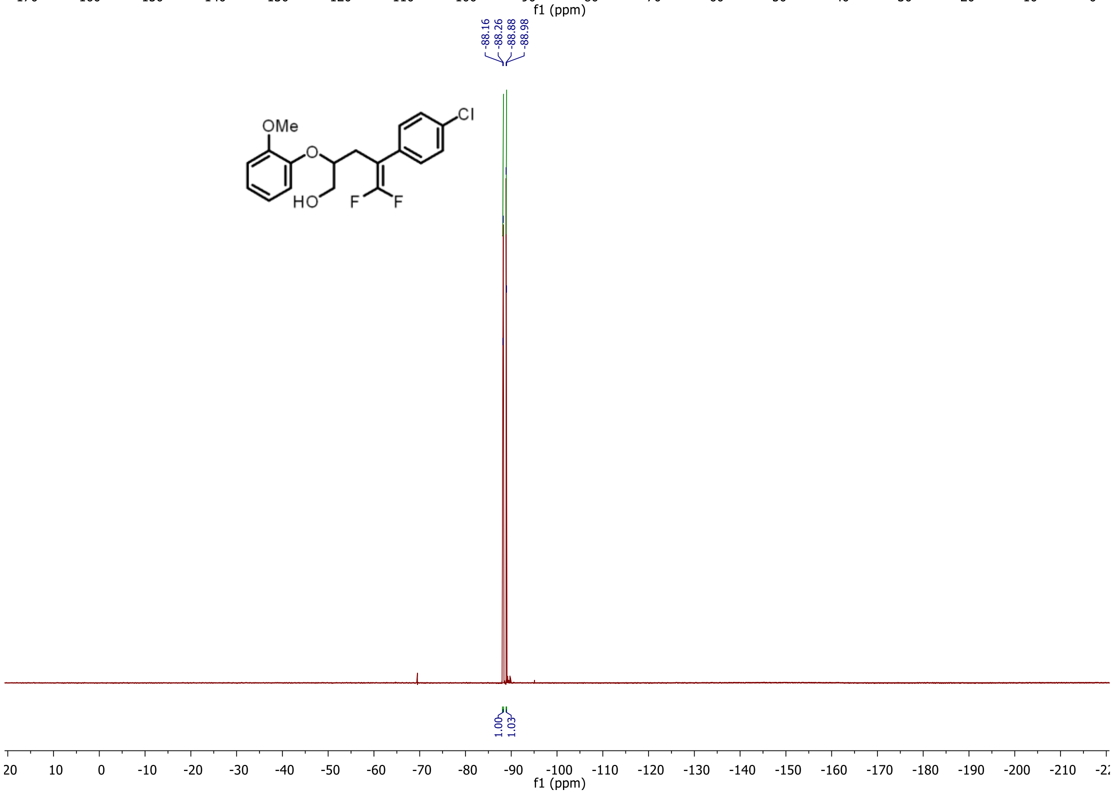
-88.16
-88.26
-88.88
-88.98
OMe
O
Cl
HO
F
F
1.00
1.03
f1 (ppm)

Compound **15**

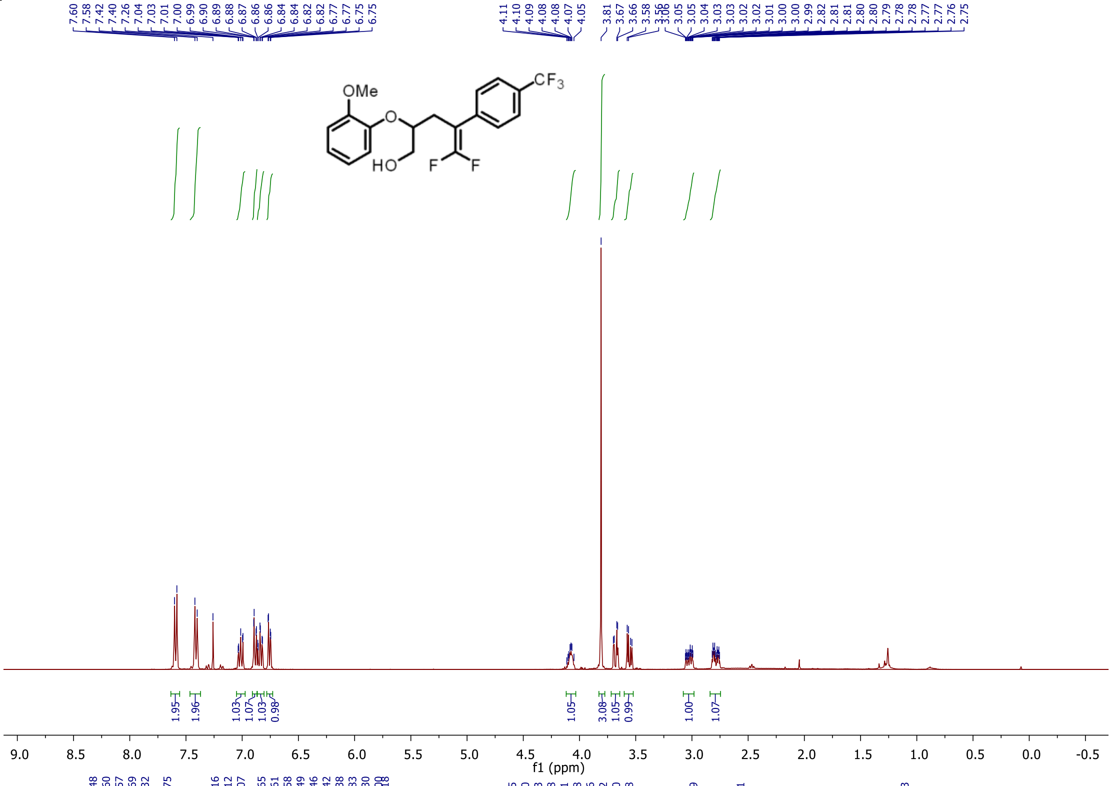

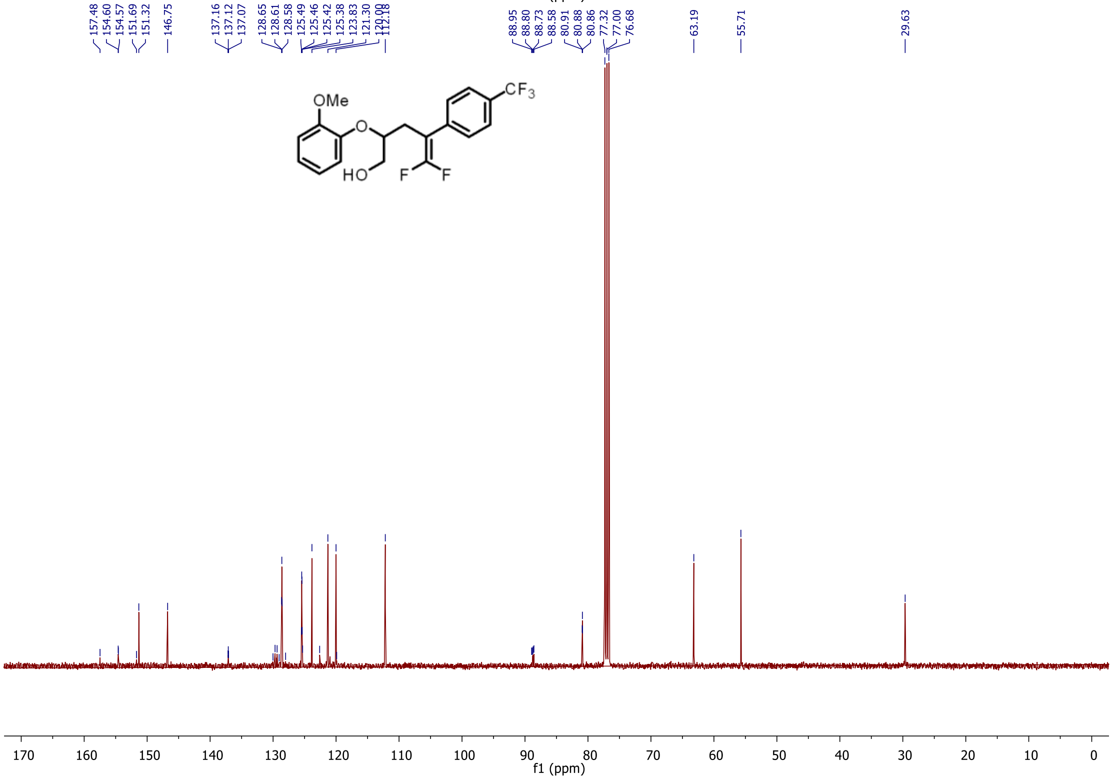

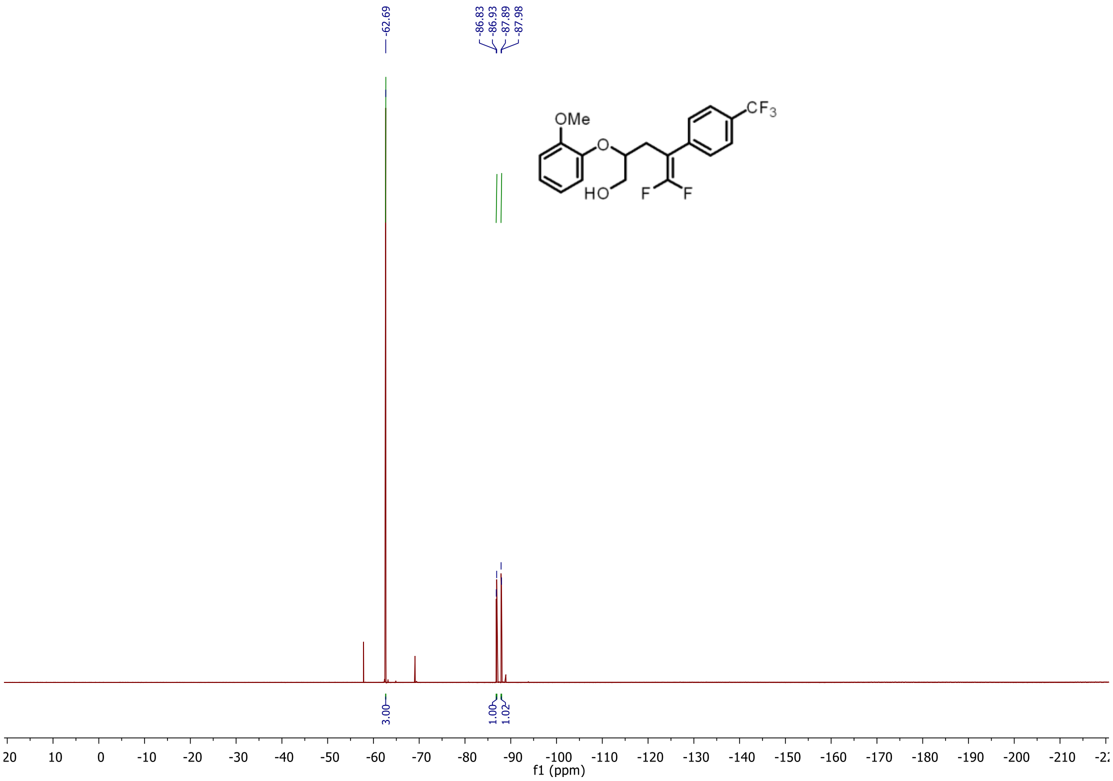
-62.69
-86.83
-86.93
-87.89
-87.98
OMe
O
HO
F
F
CF_3
3.00
1.00
1.02
20 10 0 -10 -20 -30 -40 -50 -60 -70 -80 -90 -100 -110 -120 -130 -140 -150 -160 -170 -180 -190 -200 -210
f1 (ppm)

Compound **16**

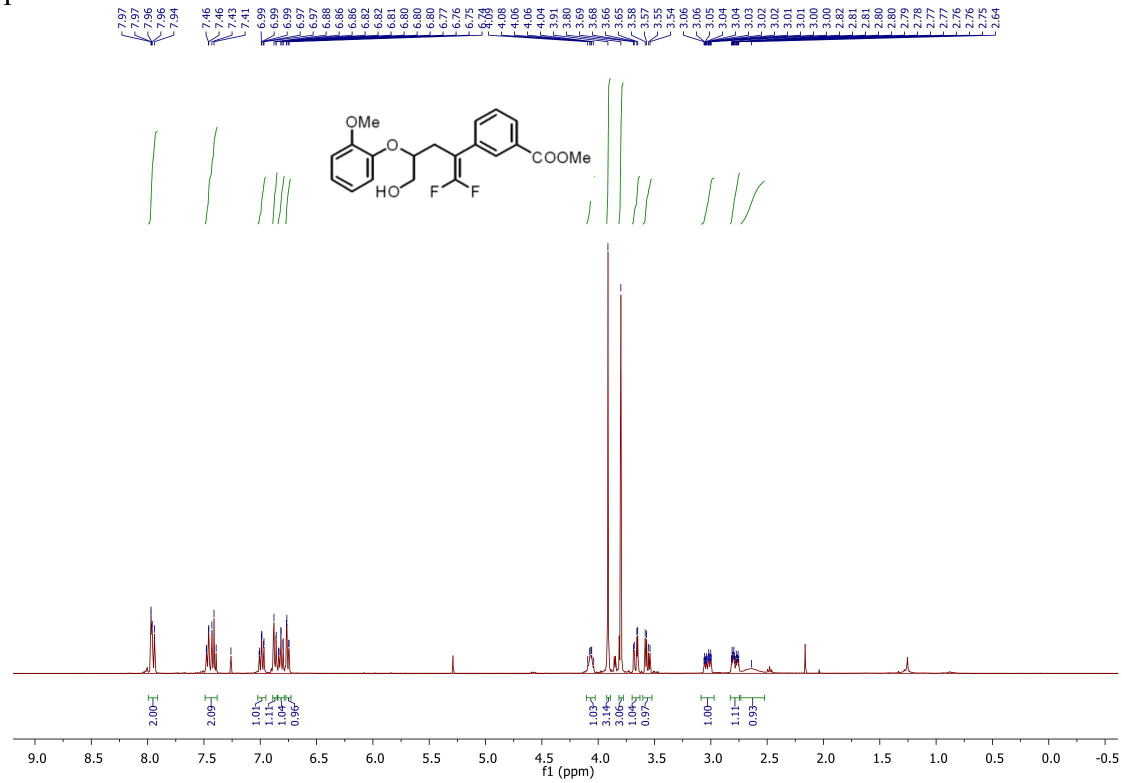
7.97 7.97 7.96 7.96 7.94 7.46 7.46 7.43 7.41 6.99 6.99 6.99 6.97 6.97 6.88 6.86 6.86 6.82 6.82 6.81 6.80 6.80 6.80 6.77 6.76 6.75 6.74 4.09 4.08 4.06 4.06 4.04 3.91 3.80 3.69 3.68 3.66 3.65 3.58 3.57 3.55 3.54 3.06 3.05 3.04 3.04 3.03 3.02 3.02 3.01 3.01 3.00 3.00 2.82 2.81 2.81 2.80 2.80 2.79 2.78 2.77 2.77 2.76 2.75 2.64
OMe
O
HO
F
F
COOMe
2.00 2.09 1.01 1.11 1.04 0.96 1.03 3.14 3.06 1.04 0.97 1.00 1.11 0.93
9.0 8.5 8.0 7.5 7.0 6.5 6.0 5.5 5.0 4.5 4.0 3.5 3.0 2.5 2.0 1.5 1.0 0.5 0.0 -0.5
f1 (ppm)

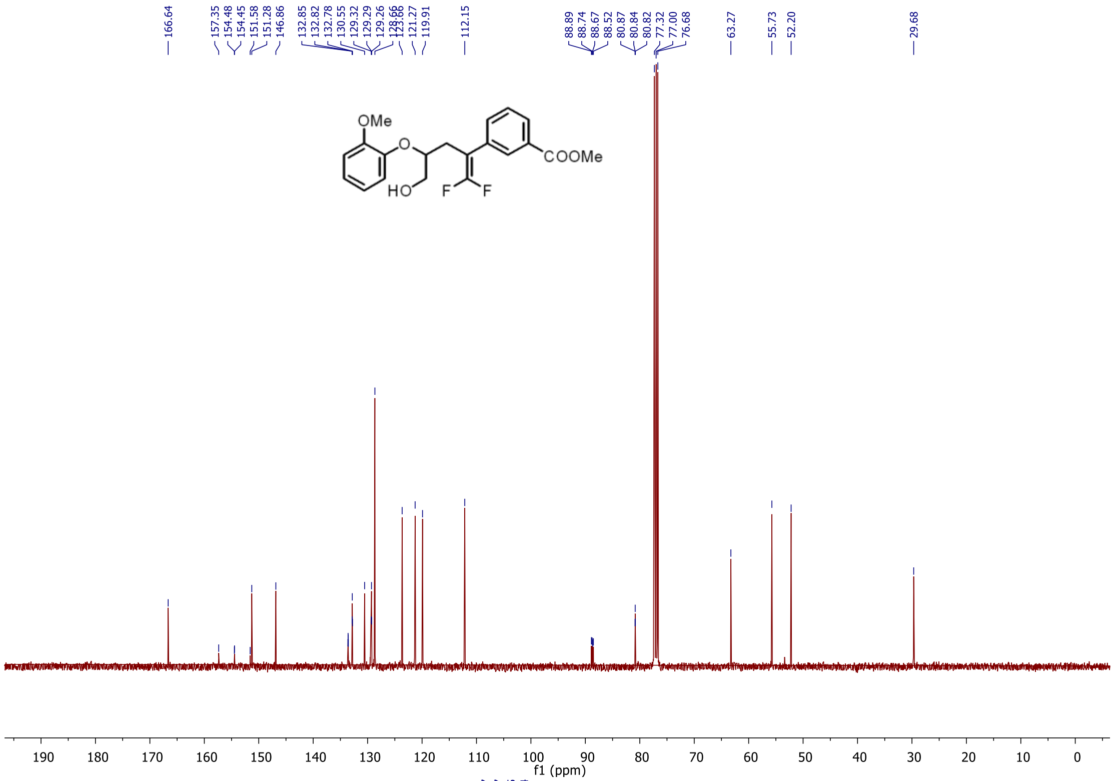
166.64
157.35
154.48
154.45
151.58
151.28
146.86
132.85
132.82
132.78
130.55
129.32
129.29
129.26
121.27
119.91
112.15
88.89
88.74
88.67
88.52
80.87
80.84
80.82
77.32
77.00
76.68
63.27
55.73
52.20
29.68
OMe
O
COOMe
HO
F
F
190 180 170 160 150 140 130 120 110 100 90 80 70 60 50 40 30 20 10 0
f1 (ppm)

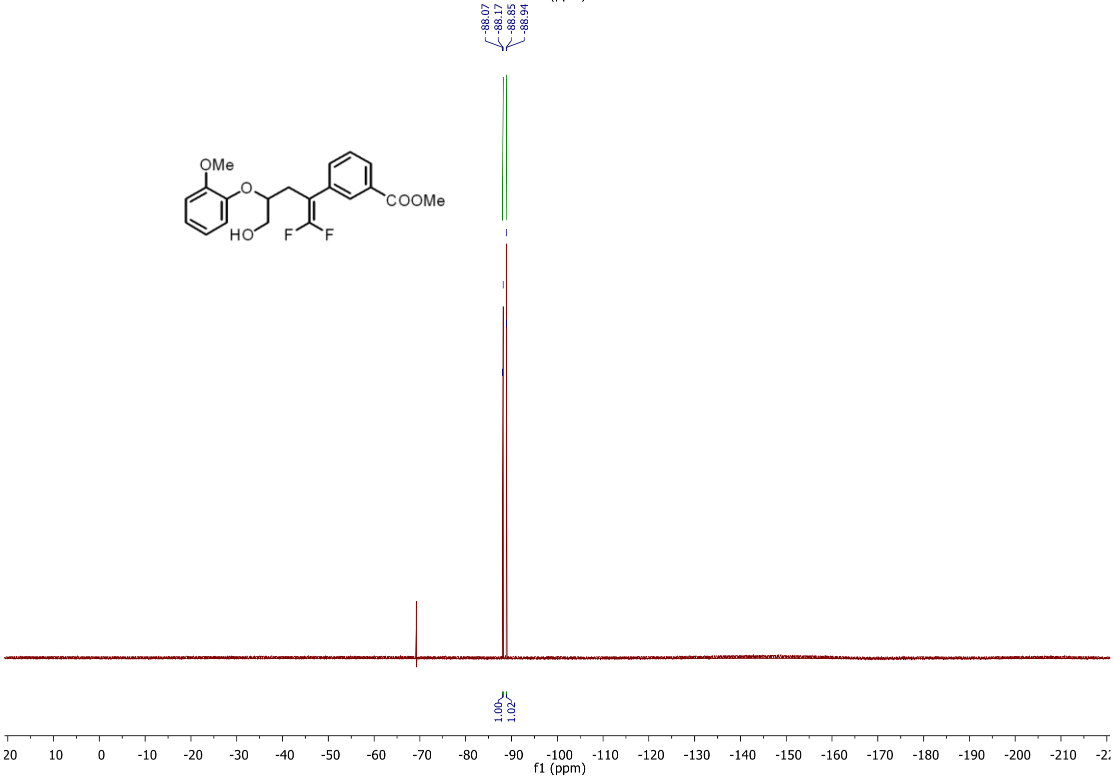
-88.07
-88.17
-88.85
-88.94
OMe
O
COOMe
HO
F
F
1.00
1.02
20 10 0 -10 -20 -30 -40 -50 -60 -70 -80 -90 -100 -110 -120 -130 -140 -150 -160 -170 -180 -190 -200 -210
f1 (ppm)

Compound **17**

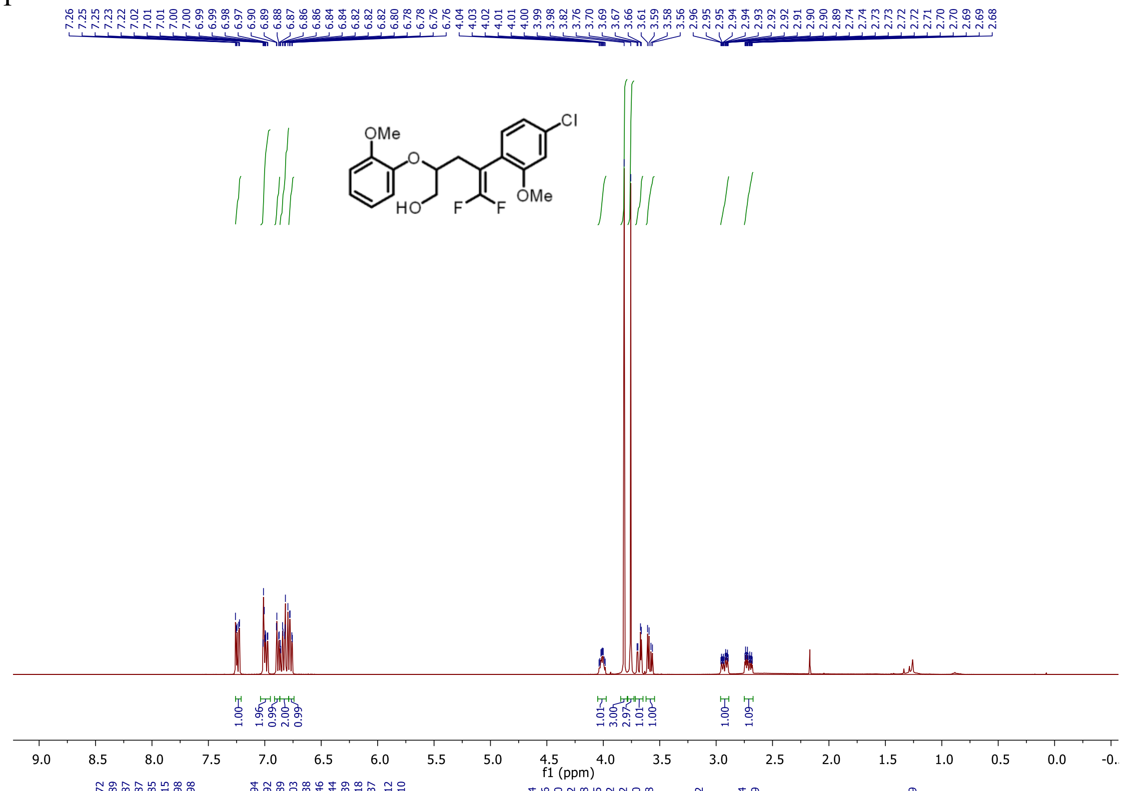

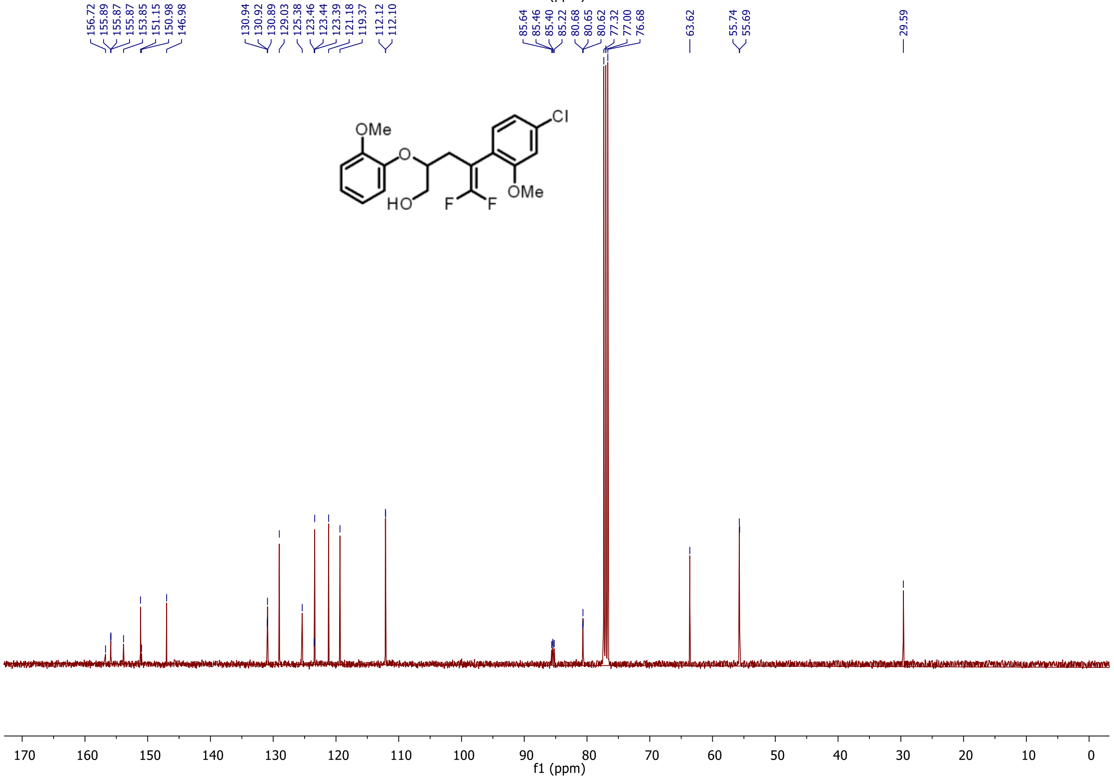

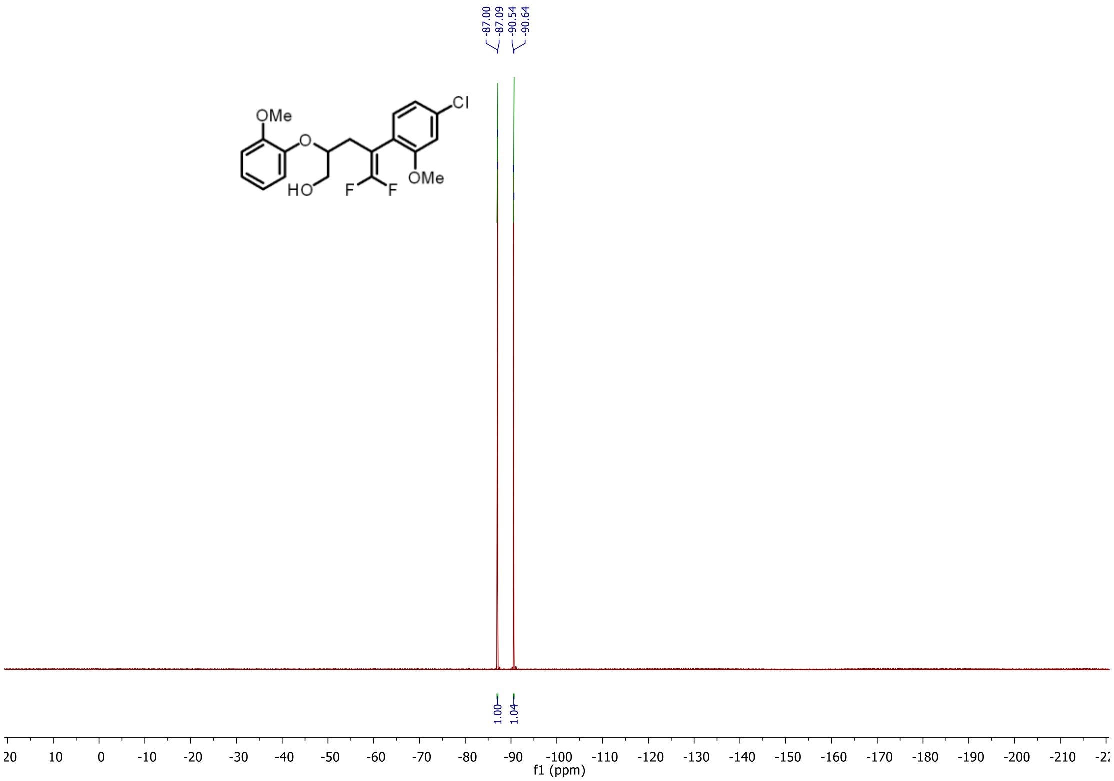

-87.00
-87.09
-90.54
-90.64
OMe
Cl
HO
F
F
OMe
1.00
1.04
f1 (ppm)

Compound **18**

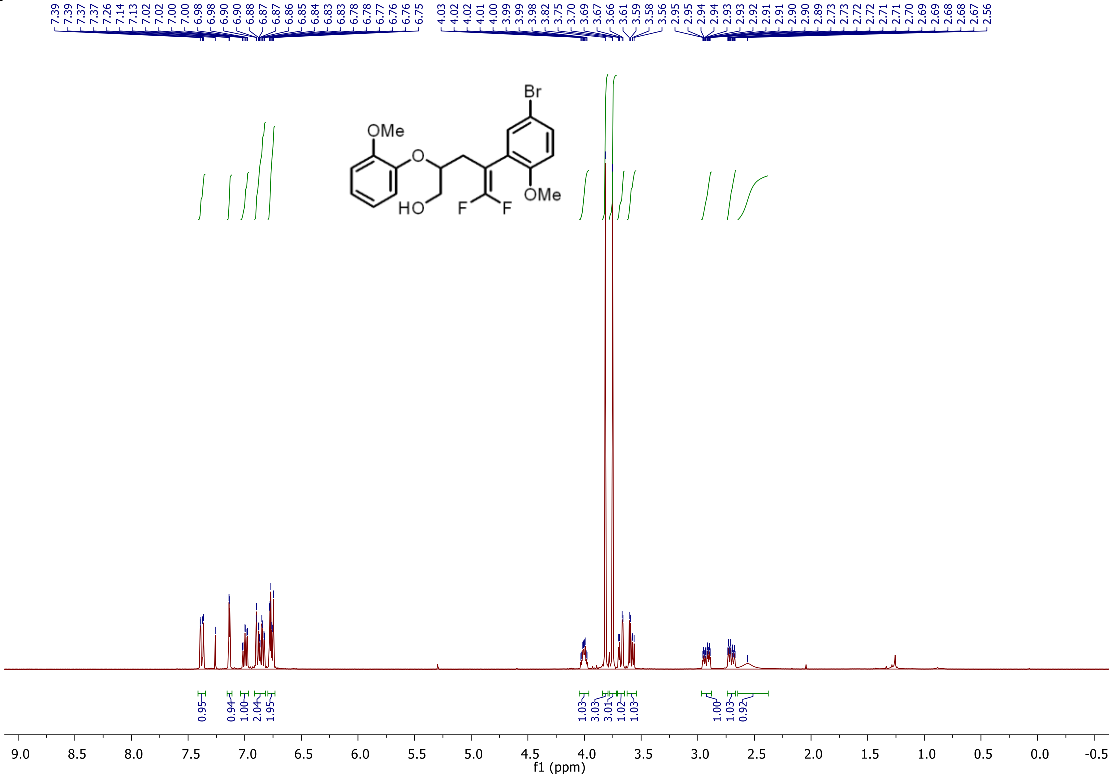

Br
OMe
HO
F
F
OMe
f1 (ppm)

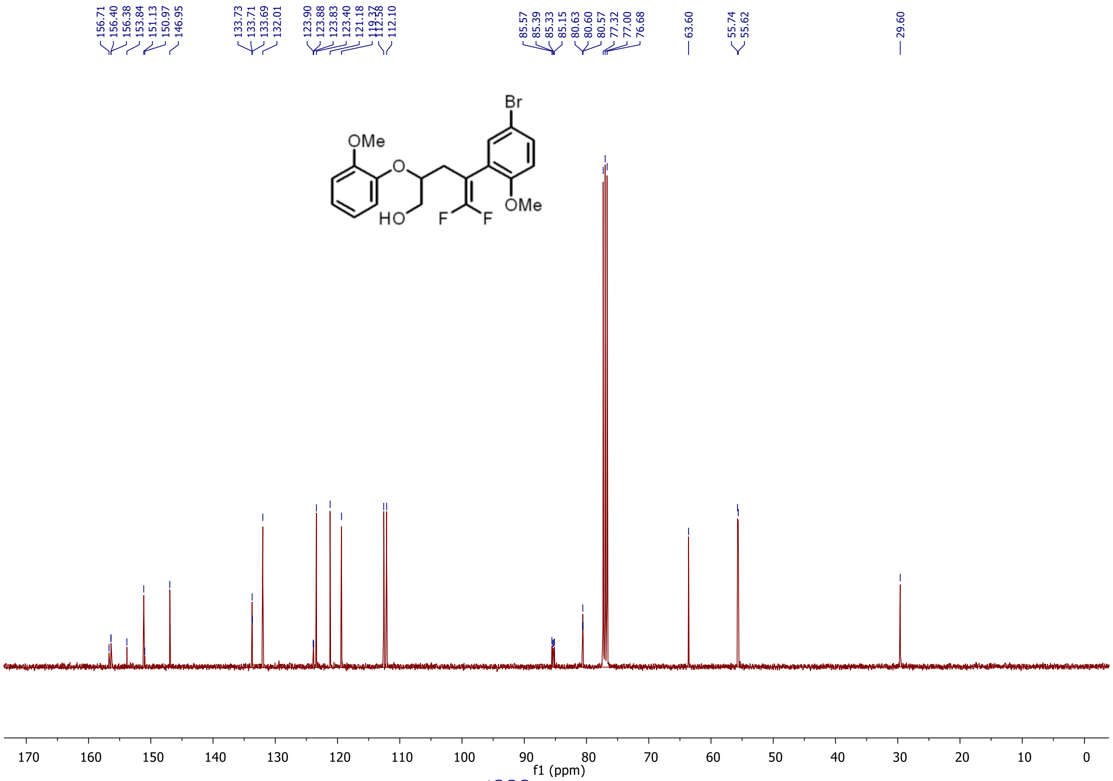
156.71
156.40
156.38
153.84
151.13
150.97
146.95
133.73
133.71
133.69
132.01
123.90
123.88
123.83
123.40
121.18
112.58
112.10
85.57
85.39
85.33
85.15
80.63
80.60
80.57
77.32
77.00
76.68
63.60
55.74
55.62
29.60
Br
OMe
O
HO
F
F
OMe
170 160 150 140 130 120 110 100 90 80 70 60 50 40 30 20 10 0
f1 (ppm)

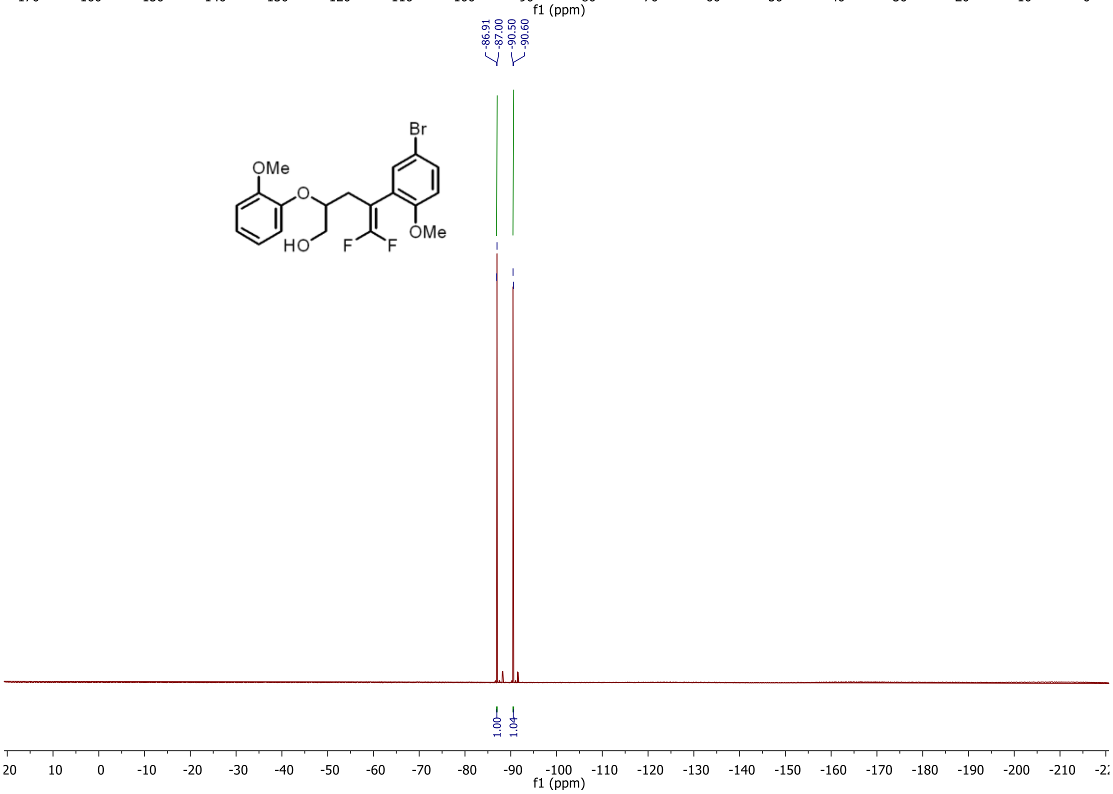
-86.91
-87.00
-90.50
-90.60
Br
OMe
O
HO
F
F
OMe
1.00
1.04
20 10 0 -10 -20 -30 -40 -50 -60 -70 -80 -90 -100 -110 -120 -130 -140 -150 -160 -170 -180 -190 -200 -210
f1 (ppm)

Compound **19**

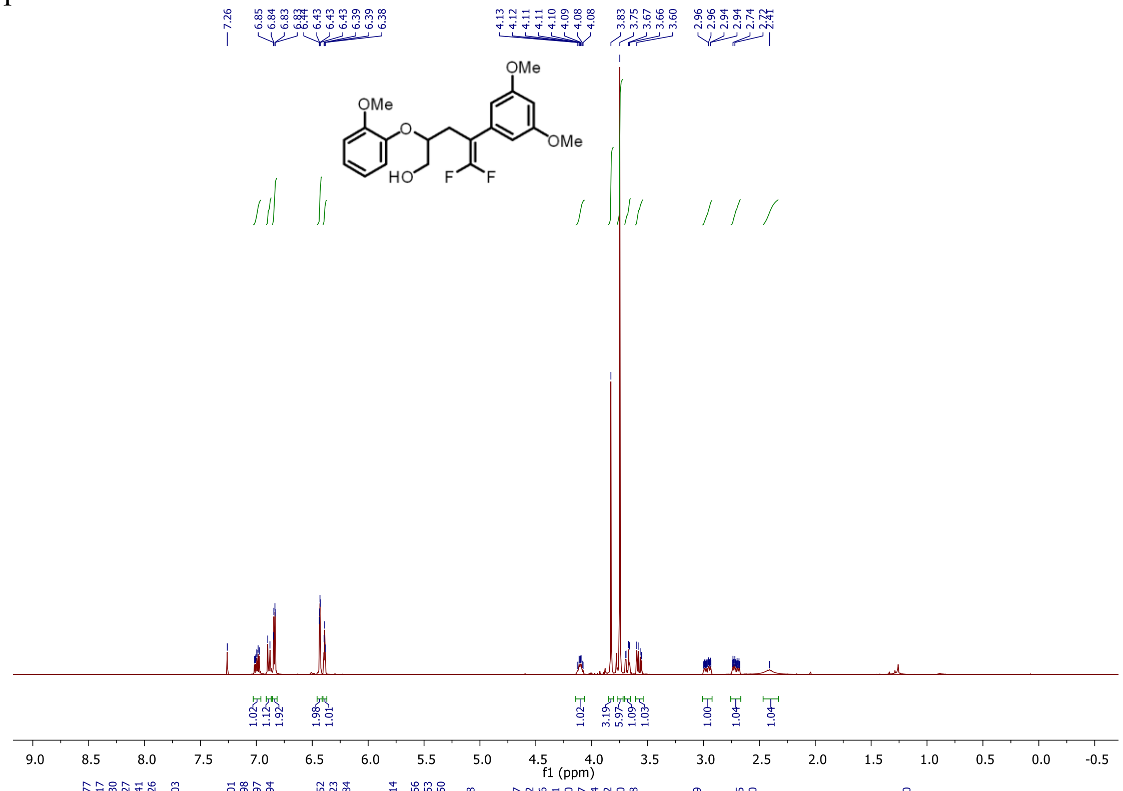

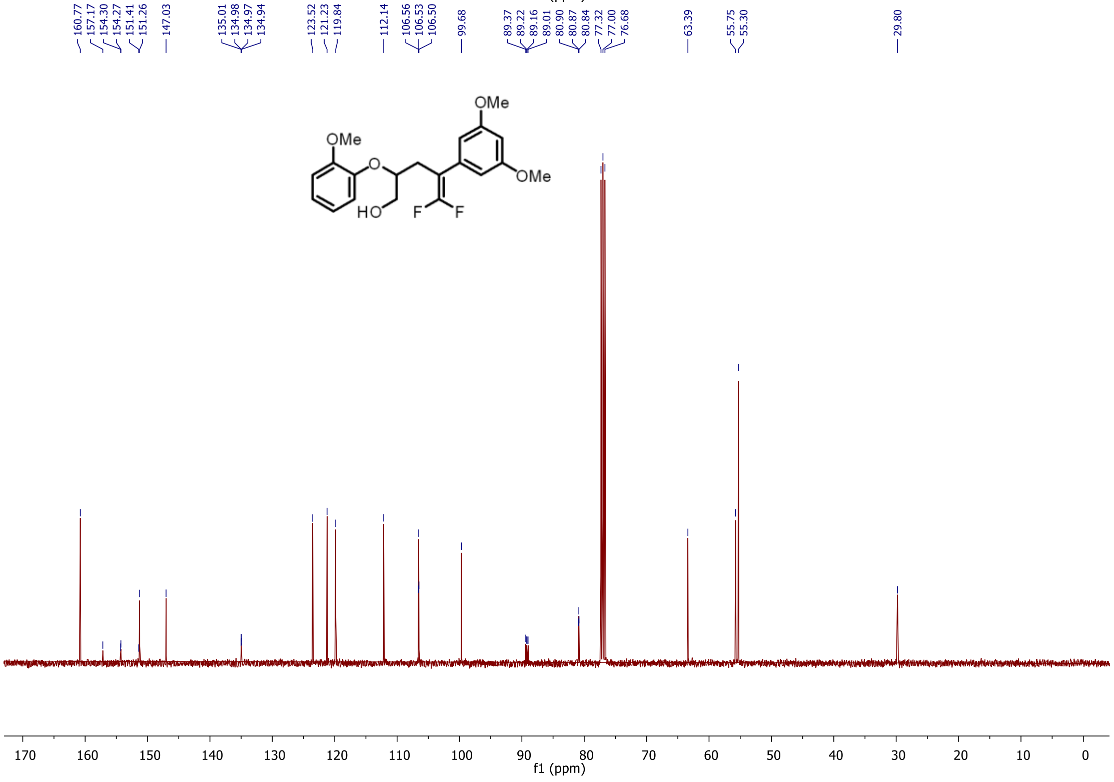

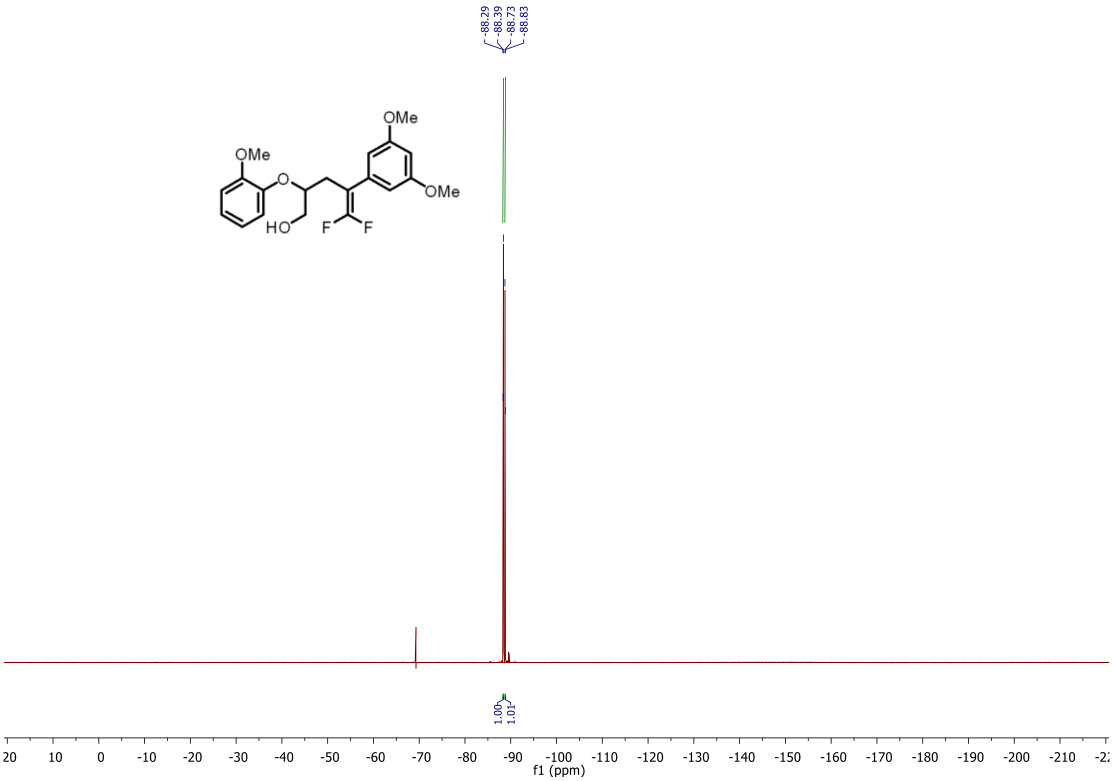

Compound **20**

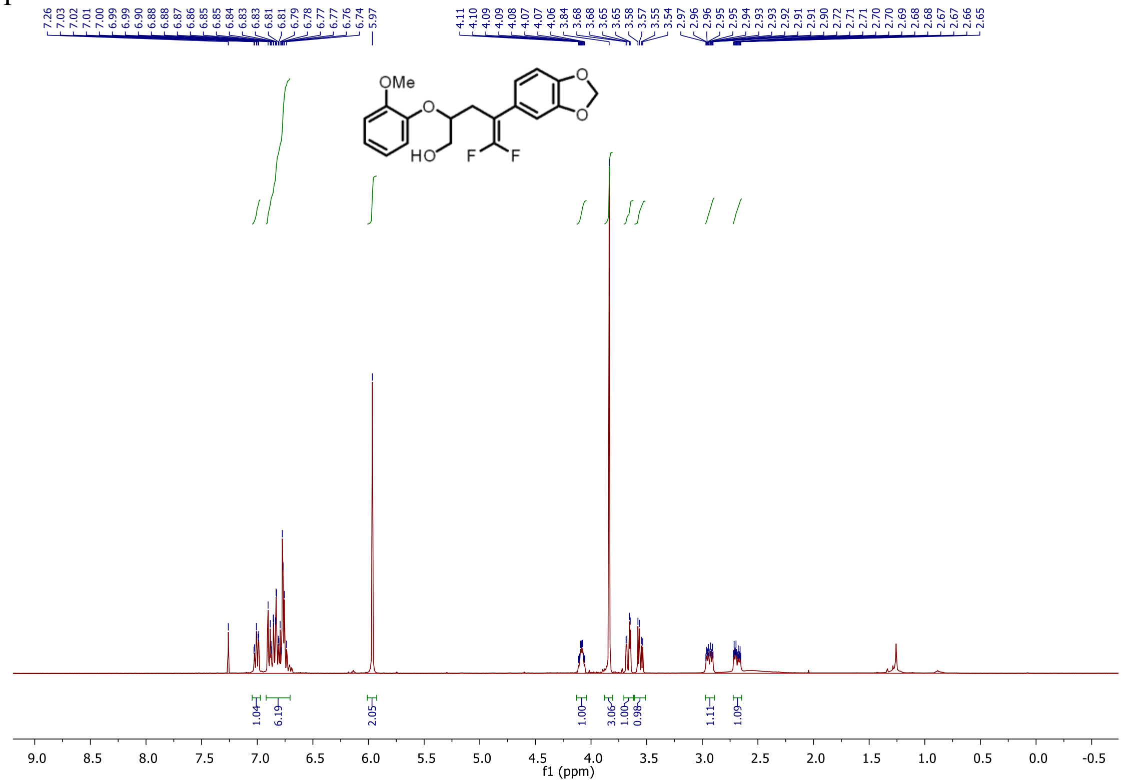

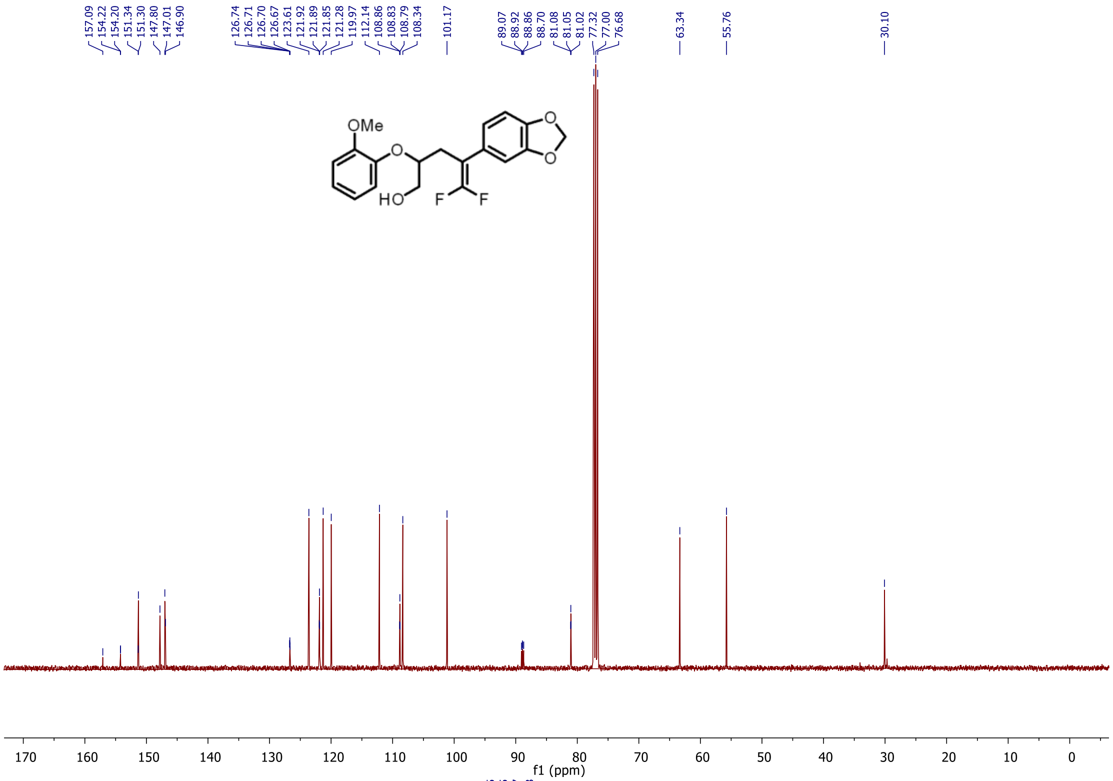
157.09
154.22
154.20
151.34
151.30
147.80
147.01
146.90
126.74
126.71
126.70
126.67
123.61
121.92
121.89
121.85
121.28
119.97
112.14
108.86
108.83
108.79
108.34
101.17
89.07
88.92
88.86
88.70
81.08
81.05
81.02
77.32
77.00
76.68
63.34
55.76
30.10
OMe
O
O
O
HO
F
F
170
160
150
140
130
120
110
100
90
80
70
60
50
40
30
20
10
0
f1 (ppm)

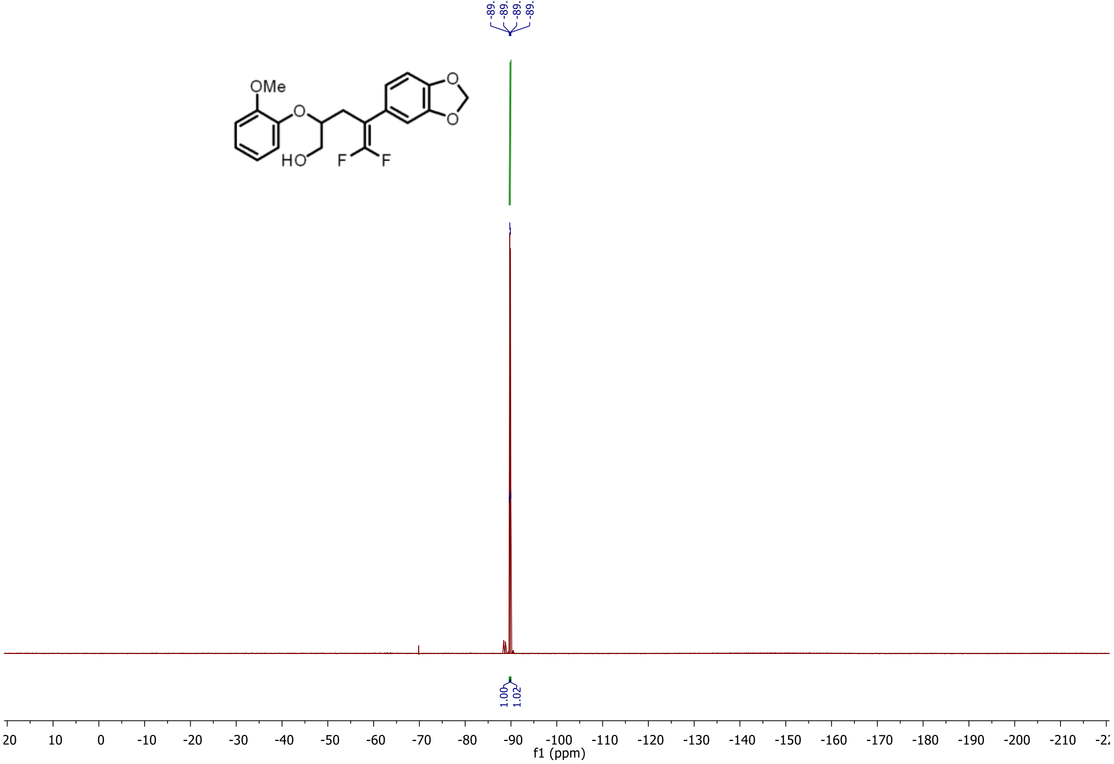
-89.65
-89.75
-89.87
-89.98
OMe
O
O
O
HO
F
F
1.00
1.02
20
10
0
-10
-20
-30
-40
-50
-60
-70
-80
-90
-100
-110
-120
-130
-140
-150
-160
-170
-180
-190
-200
-210
f1 (ppm)

Compound **21**

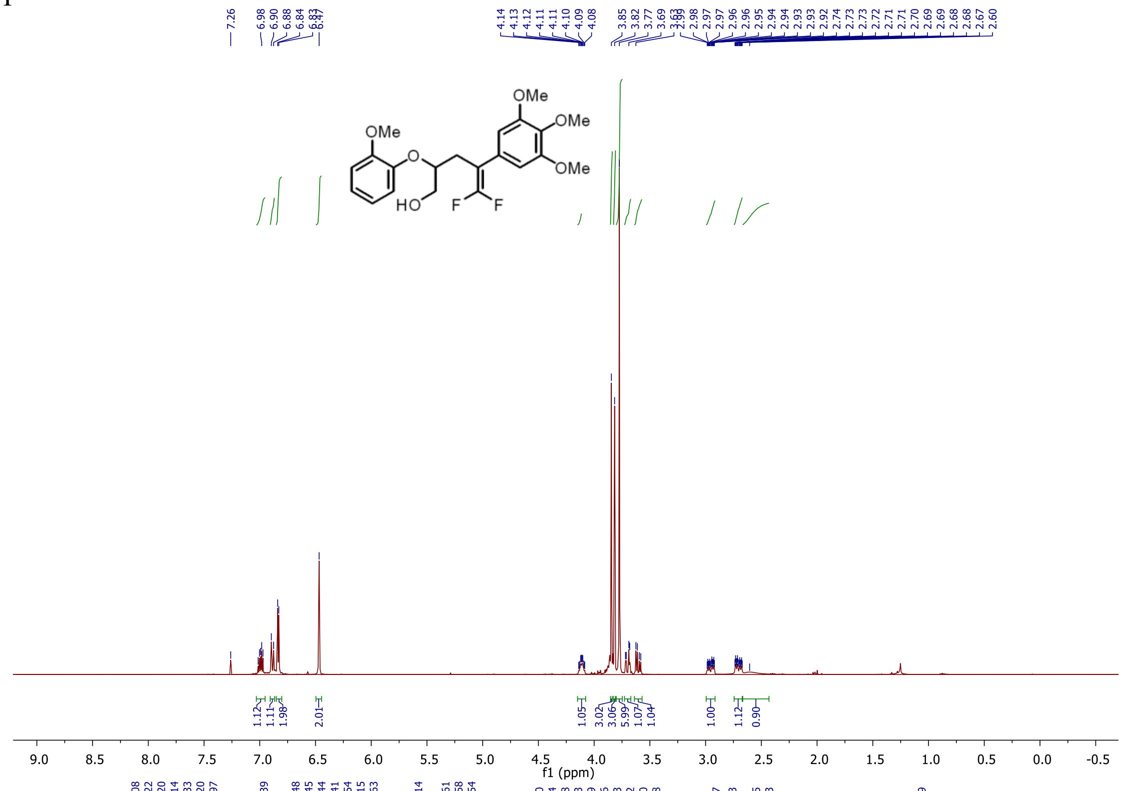

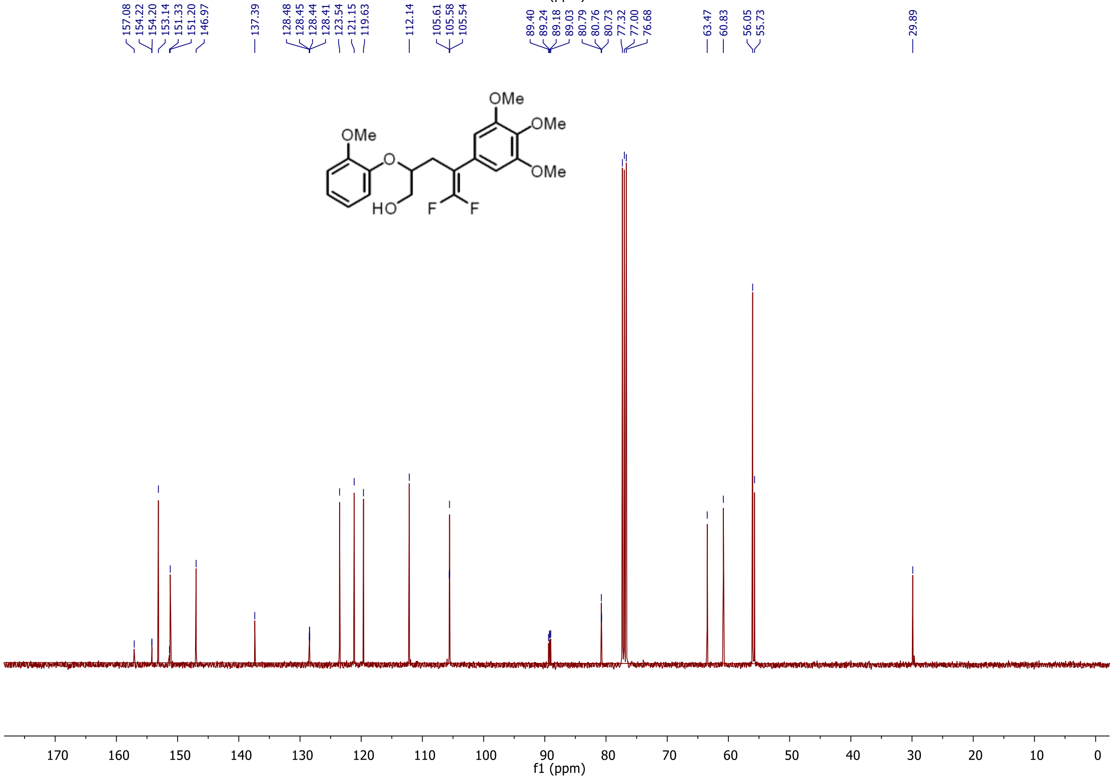

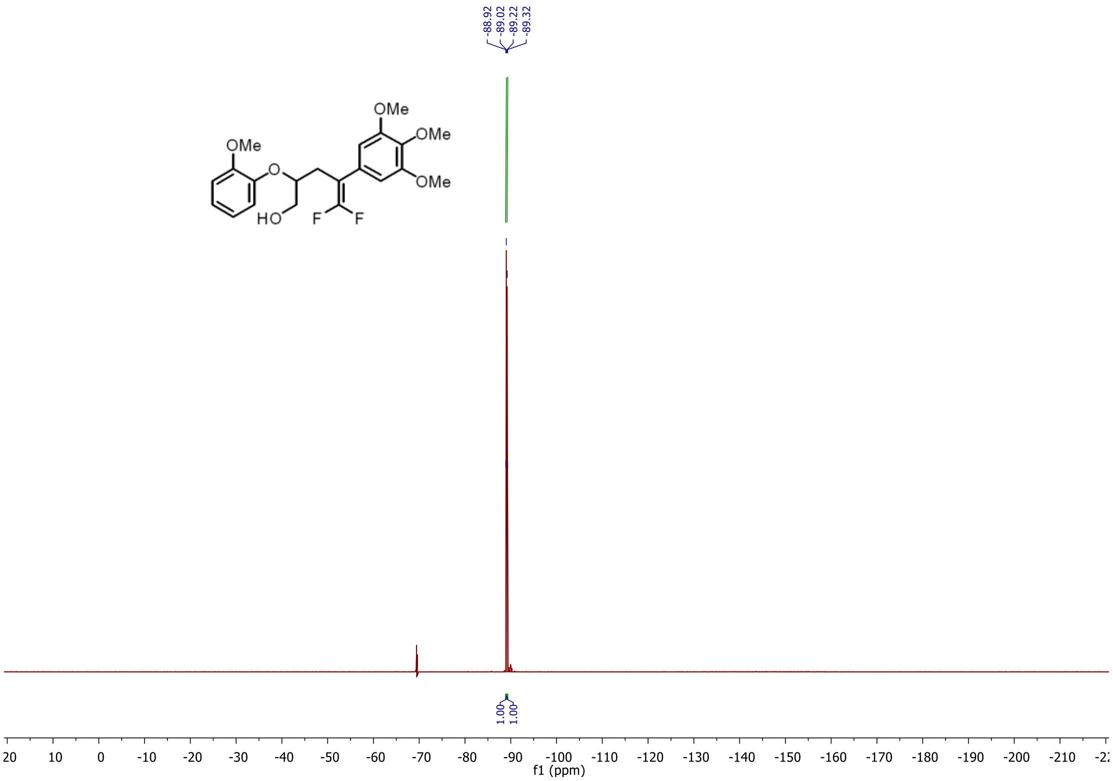
-88.92
-89.02
-89.22
-89.32
OMe
OMe
OMe
OMe
O
HO
F
F
1.00
1.00
20 10 0 -10 -20 -30 -40 -50 -60 -70 -80 -90 -100 -110 -120 -130 -140 -150 -160 -170 -180 -190 -200 -210
f1 (ppm)

Compound **22**

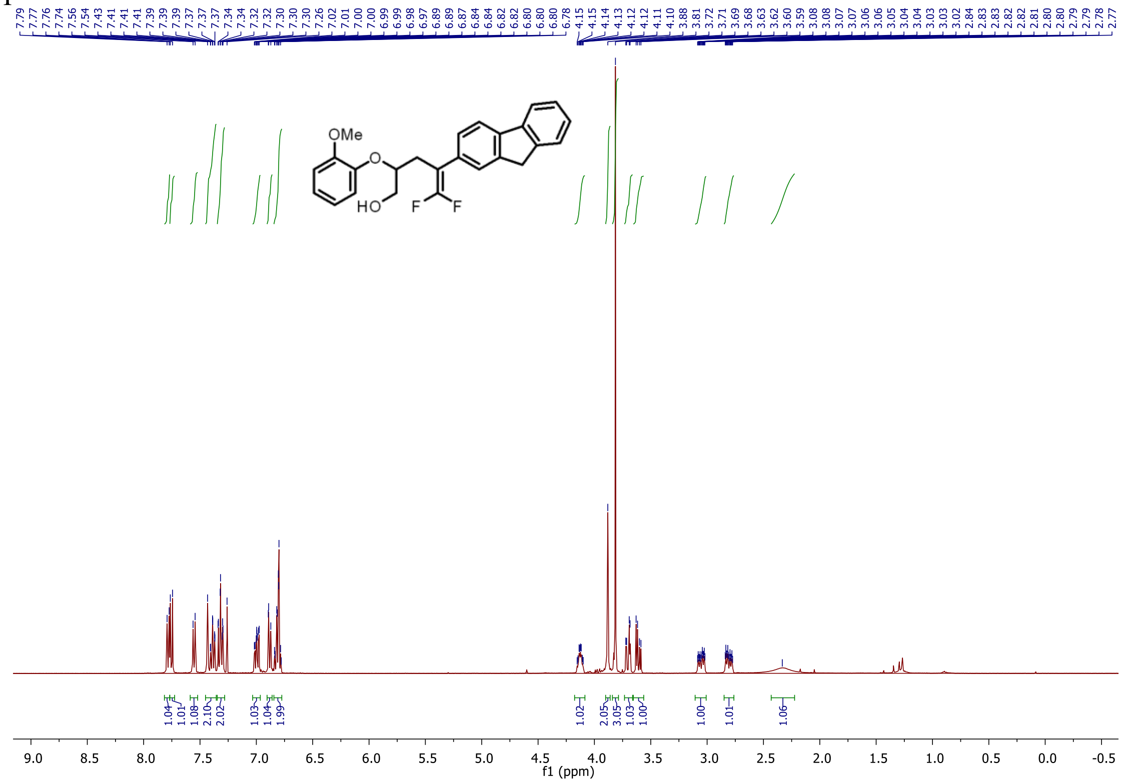
7.79 7.77 7.76 7.74 7.56 7.54 7.43 7.41 7.41 7.41 7.39 7.39 7.37 7.37 7.37 7.34 7.34 7.32 7.32 7.30 7.30 7.26 7.02 7.01 7.00 7.00 6.99 6.99 6.98 6.97 6.89 6.89 6.87 6.84 6.82 6.82 6.80 6.80 6.80 6.78 4.15 4.15 4.14 4.13 4.12 4.12 4.11 4.10 3.88 3.81 3.72 3.71 3.69 3.68 3.63 3.62 3.60 3.59 3.08 3.08 3.07 3.07 3.06 3.05 3.04 3.04 3.03 3.03 3.02 2.84 2.83 2.83 2.82 2.82 2.81 2.80 2.80 2.79 2.79 2.78 2.77
OMe
O
HO
F
F
1.04 1.01 1.08 2.10 2.02 1.03 1.04 1.99 1.02 2.05 3.05 1.03 1.00 1.00 1.01 1.06
9.0 8.5 8.0 7.5 7.0 6.5 6.0 5.5 5.0 4.5 4.0 3.5 3.0 2.5 2.0 1.5 1.0 0.5 0.0 -0.5
f1 (ppm)

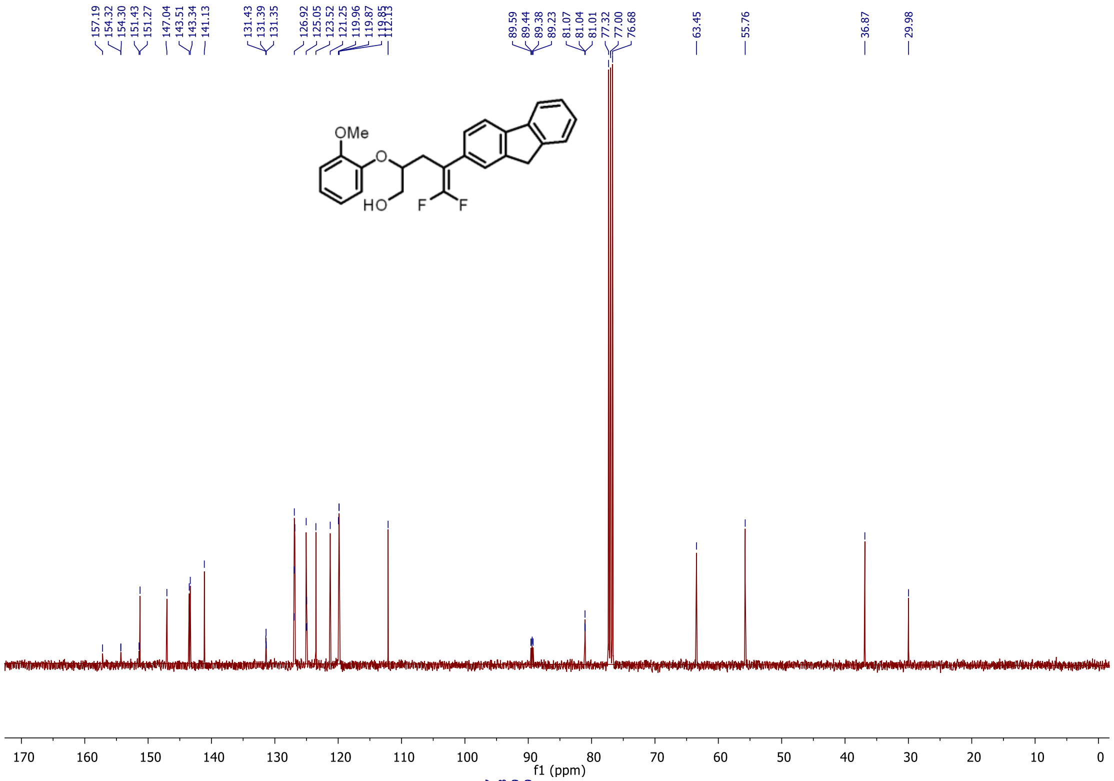
157.19
154.32
154.30
151.43
151.27
147.04
143.51
143.34
141.13
131.43
131.39
131.35
126.92
125.05
123.52
121.25
119.96
119.87
119.85
112.13
89.59
89.44
89.38
89.23
81.07
81.04
81.01
77.32
77.00
76.68
63.45
55.76
36.87
29.98
OMe
O
HO
F
F
f1 (ppm)

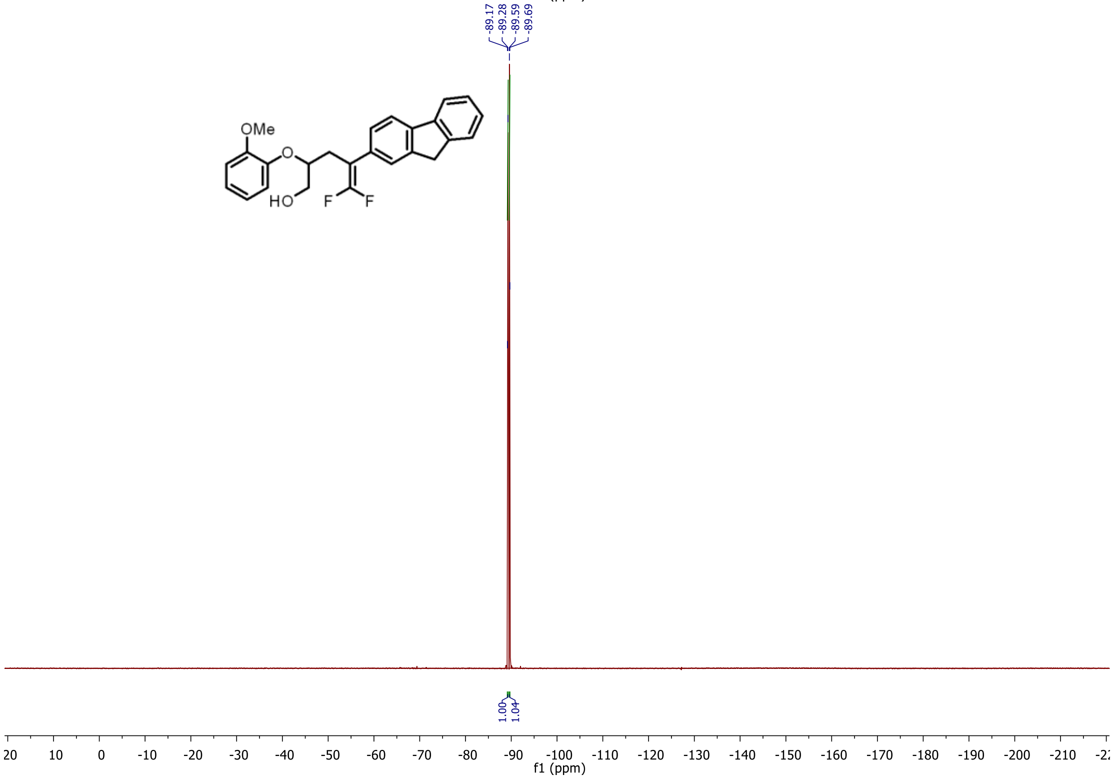
-89.17
-89.28
-89.59
-89.69
OMe
O
HO
F
F
1.00
1.04
f1 (ppm)

Compound **23**

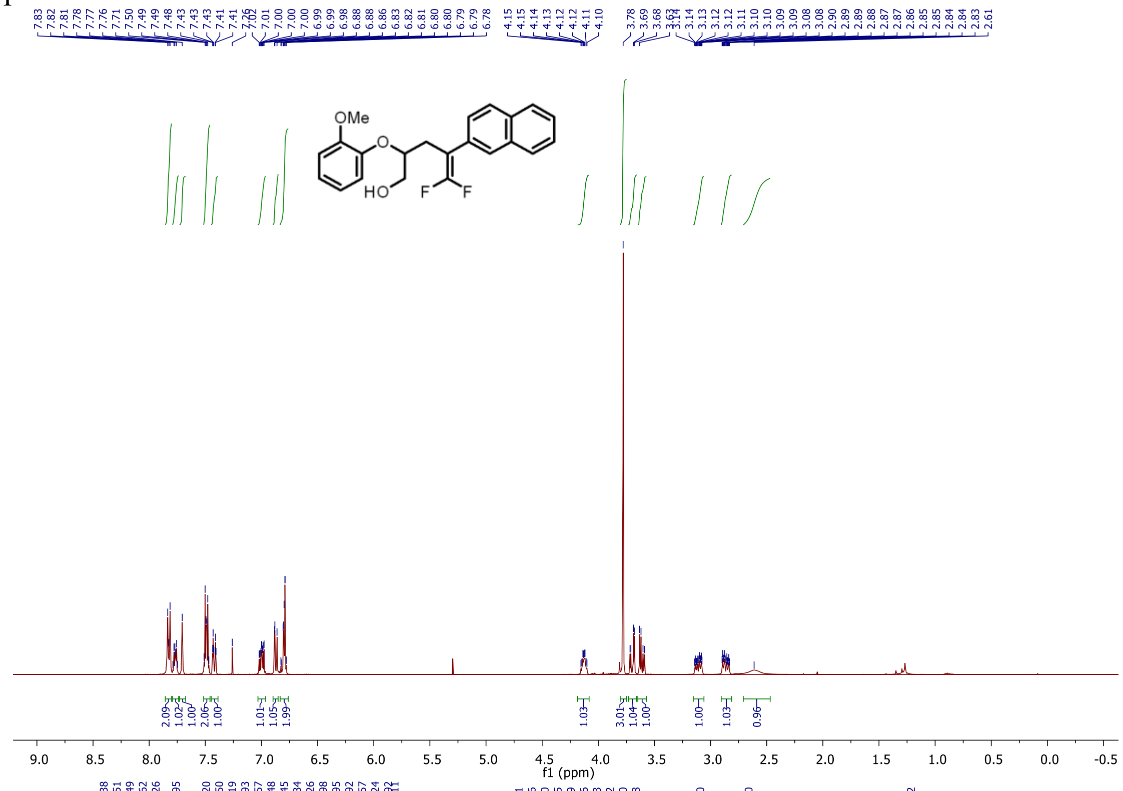

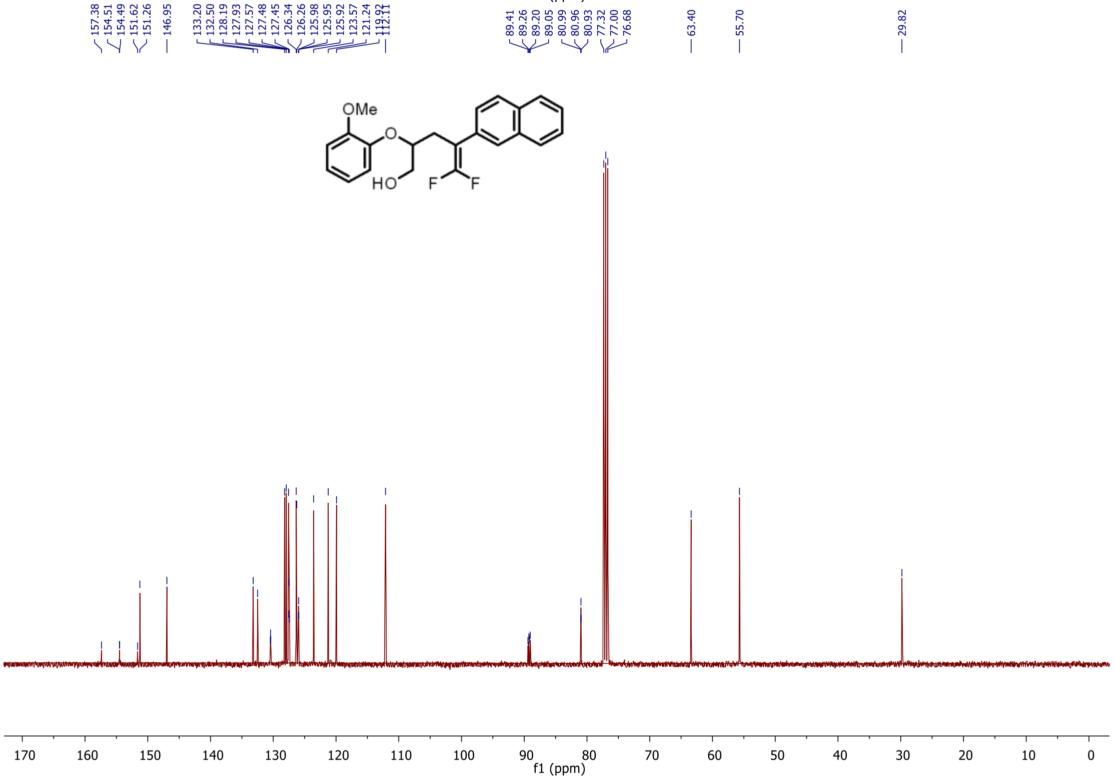

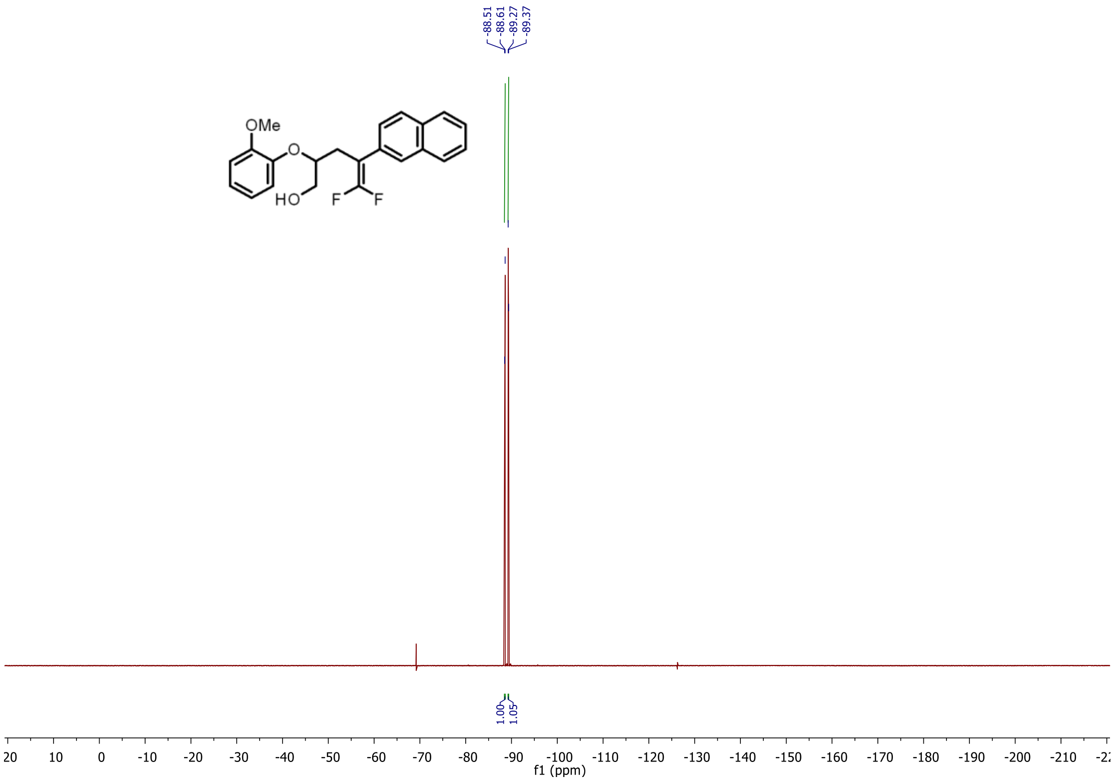

-88.51
-88.61
-89.27
-89.37
OMe
O
HO
F
F
1.00
1.05
f1 (ppm)

Compound **24**

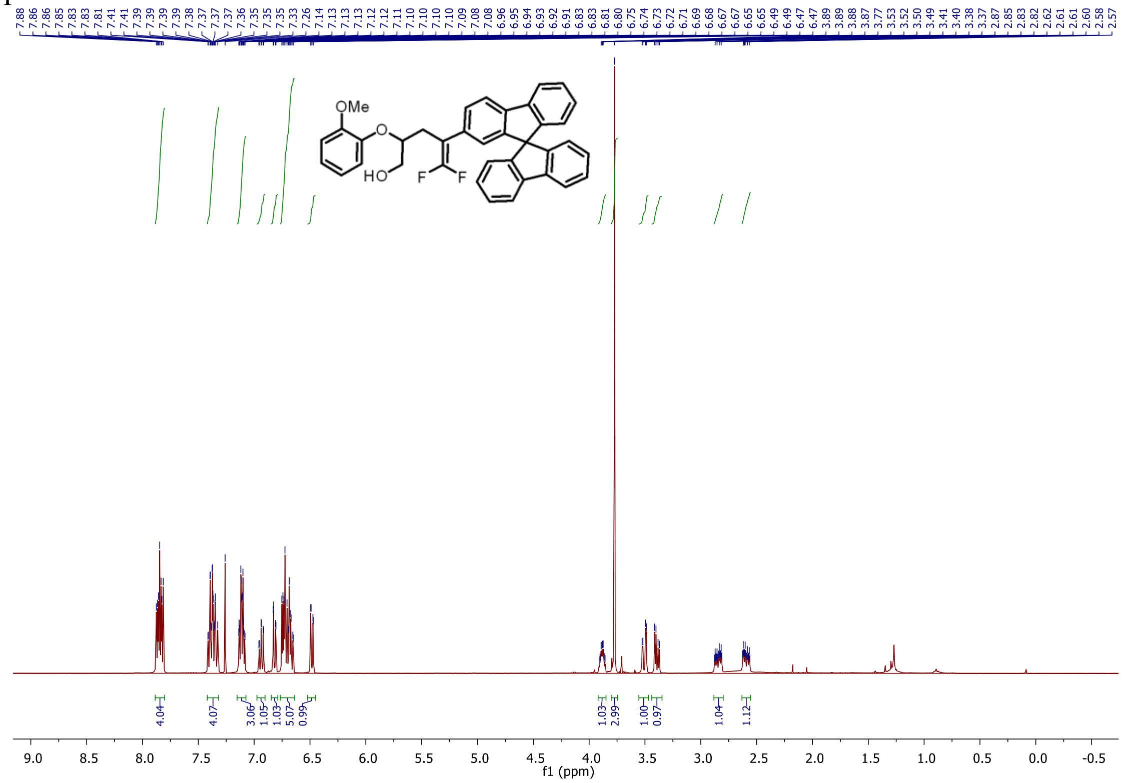

OMe
O
HO
F
F
4.04
4.07
3.06
1.05
1.03
5.07
0.99
1.03
2.99
1.00
0.97
1.04
1.12
f1 (ppm)

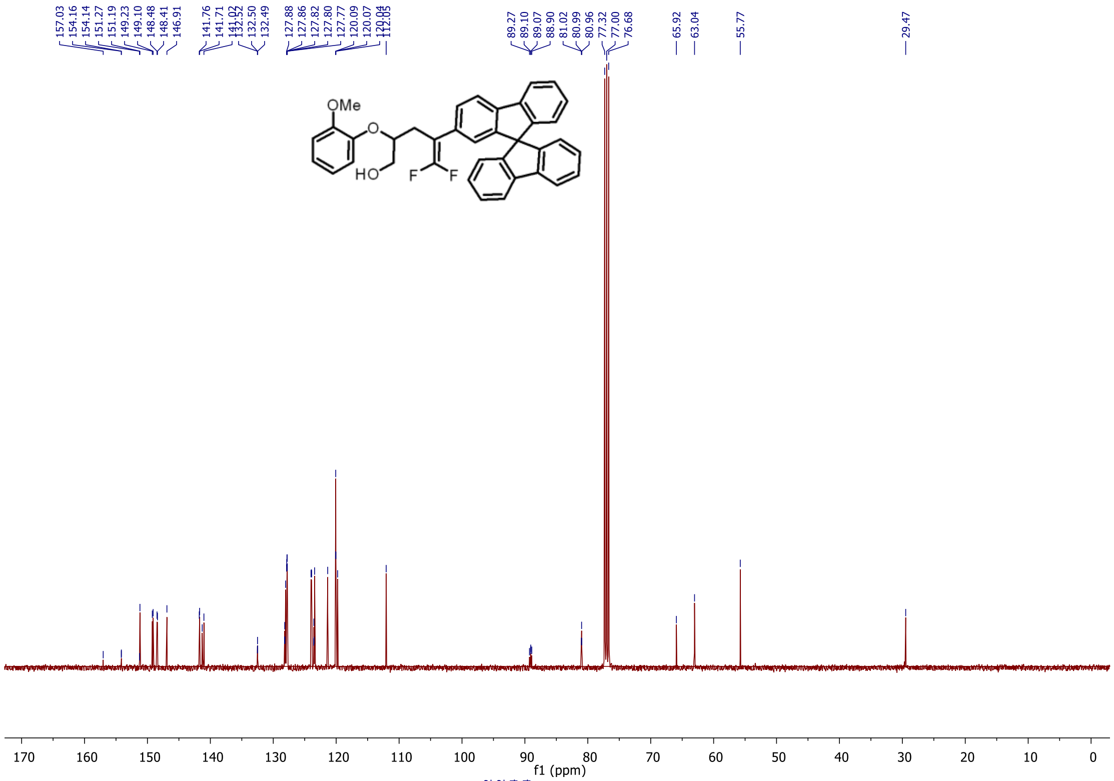
157.03
154.16
154.14
151.27
151.19
149.23
149.10
148.48
148.41
146.91
141.76
141.71
132.50
132.49
127.88
127.86
127.82
127.80
127.77
120.09
120.07
89.27
89.10
89.07
88.90
81.02
80.99
80.96
77.32
77.00
76.68
65.92
63.04
55.77
29.47
OMe
O
HO
F
F
f1 (ppm)

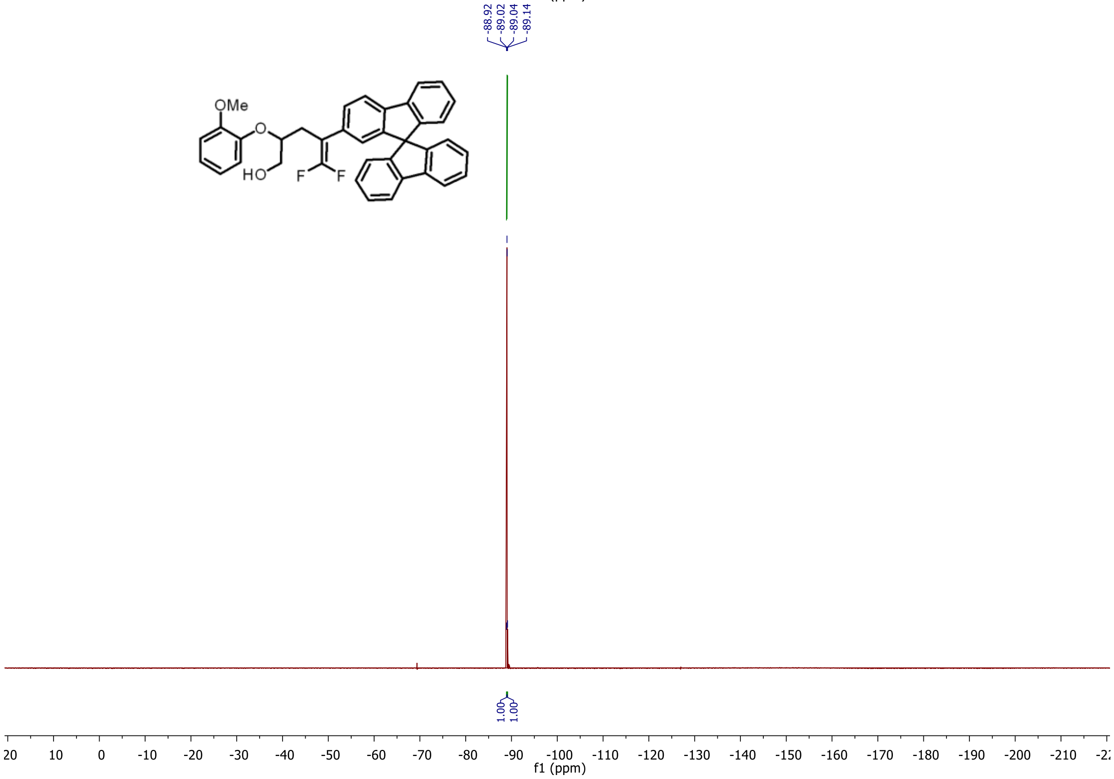
-88.92
-89.02
-89.04
-89.14
OMe
O
HO
F
F
1.00
1.00
f1 (ppm)

Compound **25**

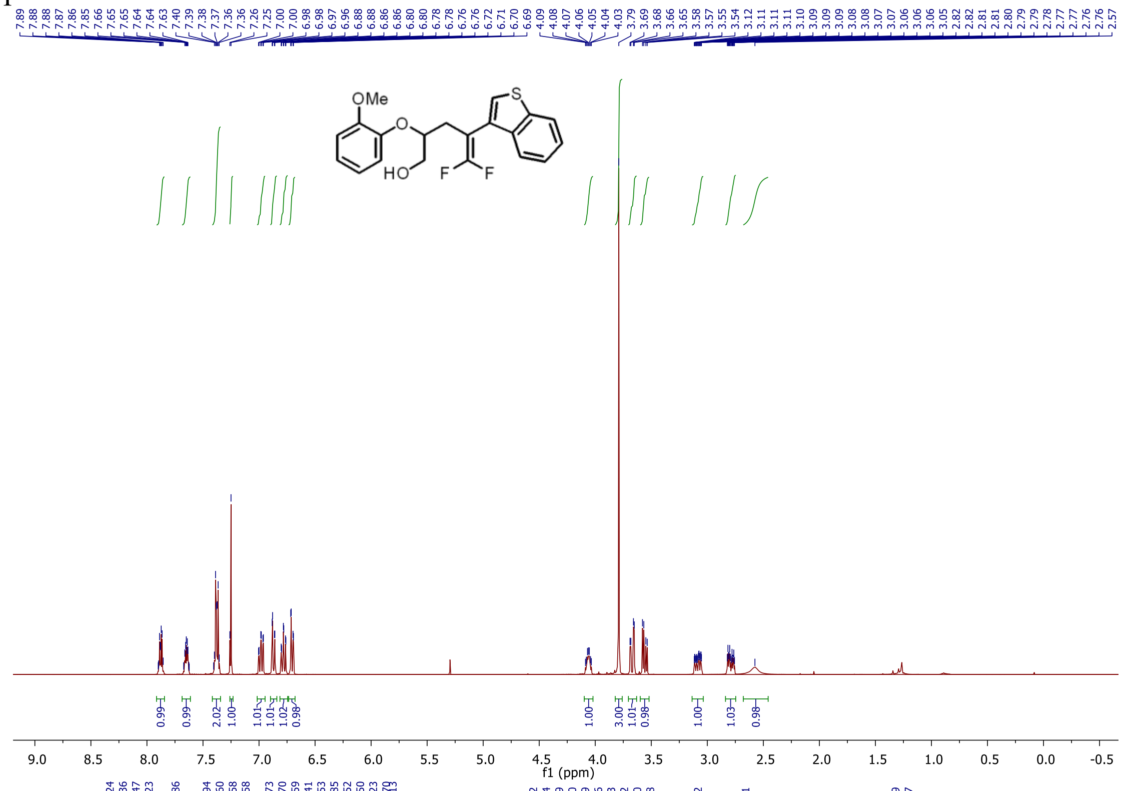

OMe
O
HO
F
F
S
f1 (ppm)

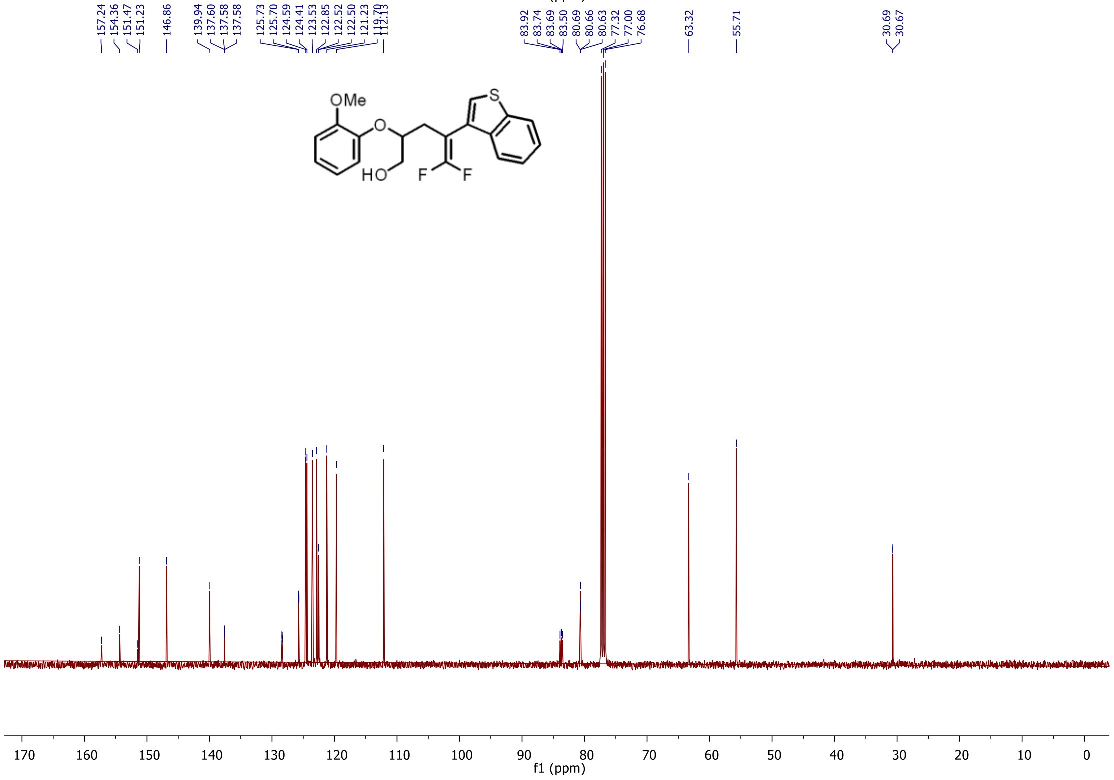

157.24
154.36
151.47
151.23
146.86
139.94
137.60
137.58
137.58
125.73
125.70
124.59
124.41
123.53
122.85
122.52
122.50
121.23
83.92
83.74
83.69
83.50
80.69
80.66
80.63
77.32
77.00
76.68
63.32
55.71
30.69
30.67
OMe
O
HO
F
F
S
f1 (ppm)

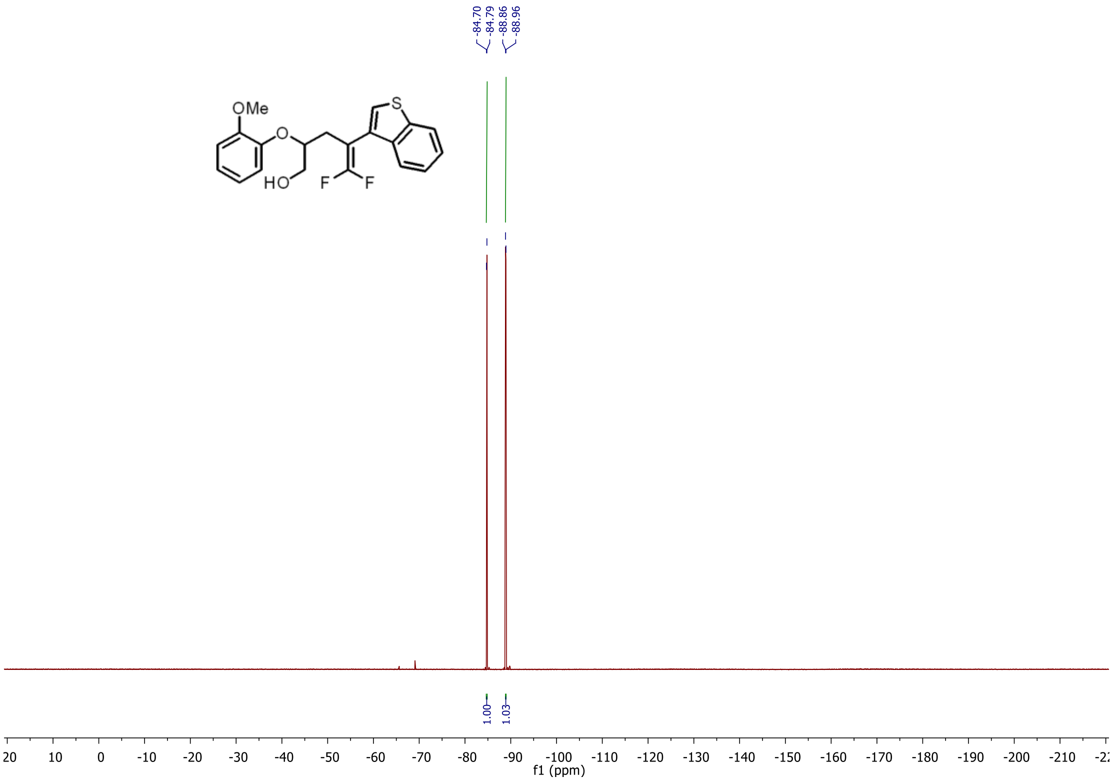

Compound **26**

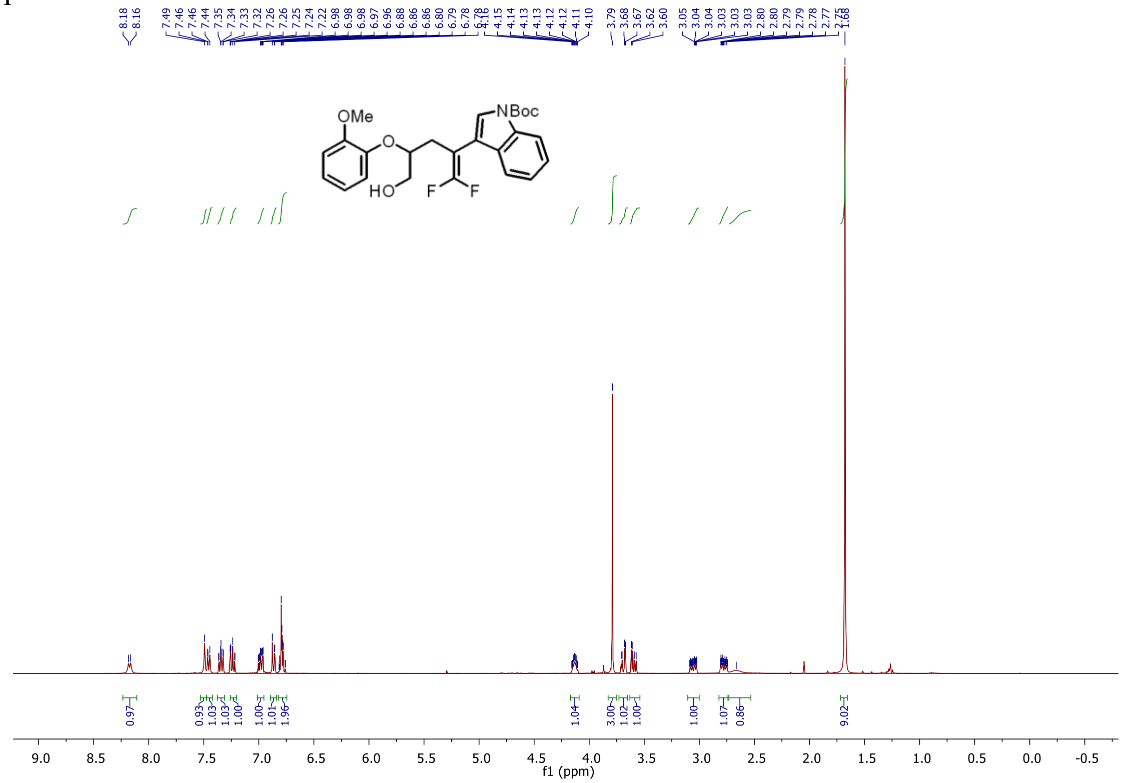

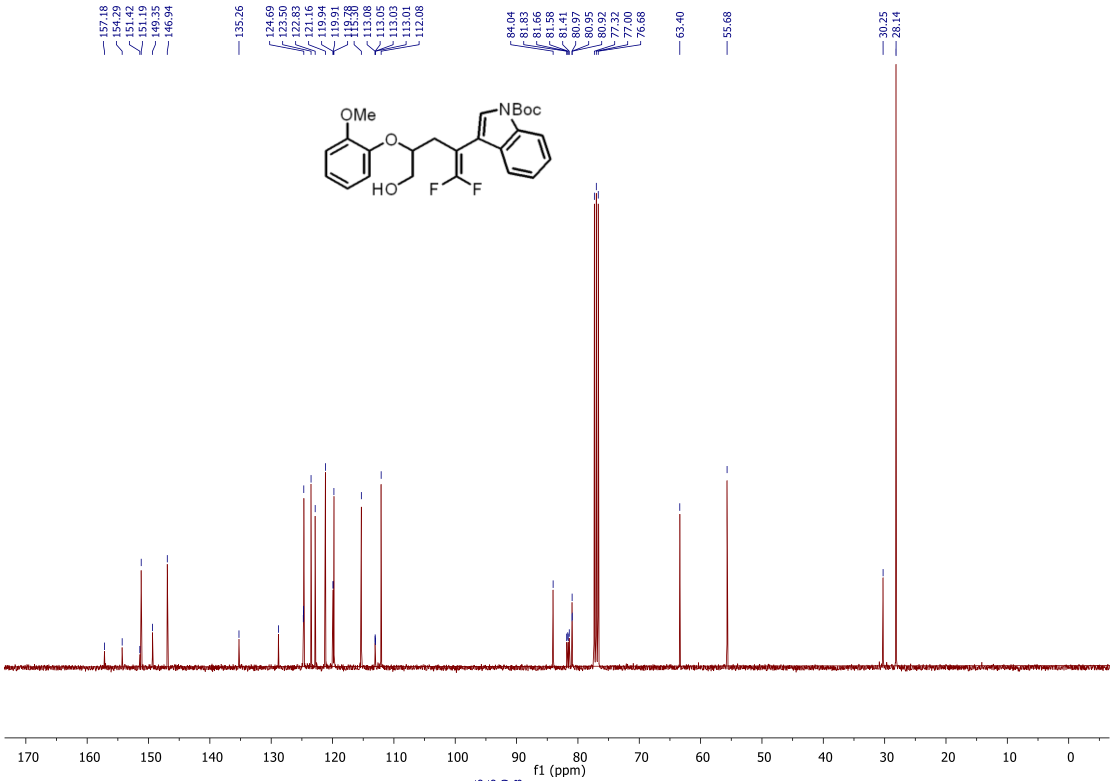

157.18
154.29
151.42
151.19
149.35
146.94
135.26
124.69
123.50
122.83
121.16
119.94
119.91
119.78
115.30
113.08
113.05
113.03
113.01
112.08
84.04
81.83
81.66
81.58
81.41
80.97
80.95
80.92
77.32
77.00
76.68
63.40
55.68
30.25
28.14
OMe
O
NBoc
HO
F
F
170 160 150 140 130 120 110 100 90 80 70 60 50 40 30 20 10 0
f1 (ppm)

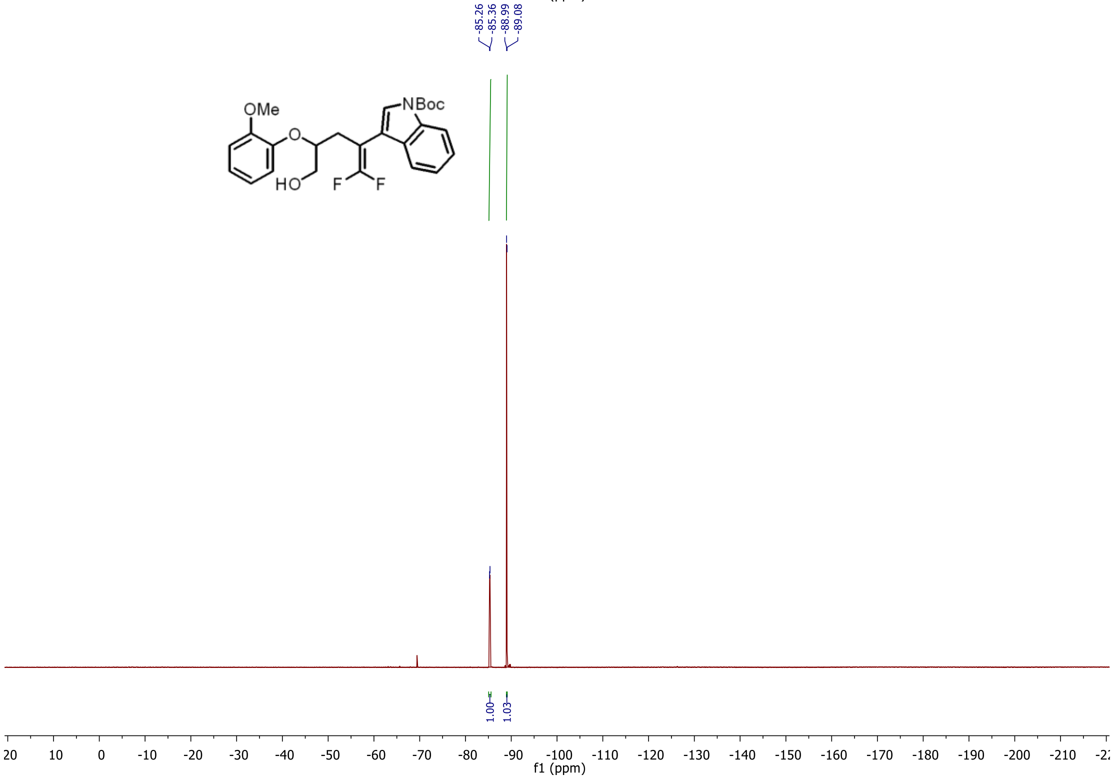

-85.26
-85.36
-88.99
-89.08
OMe
O
NBoc
HO
F
F
1.00
1.03
20 10 0 -10 -20 -30 -40 -50 -60 -70 -80 -90 -100 -110 -120 -130 -140 -150 -160 -170 -180 -190 -200 -210
f1 (ppm)

Compound **27**

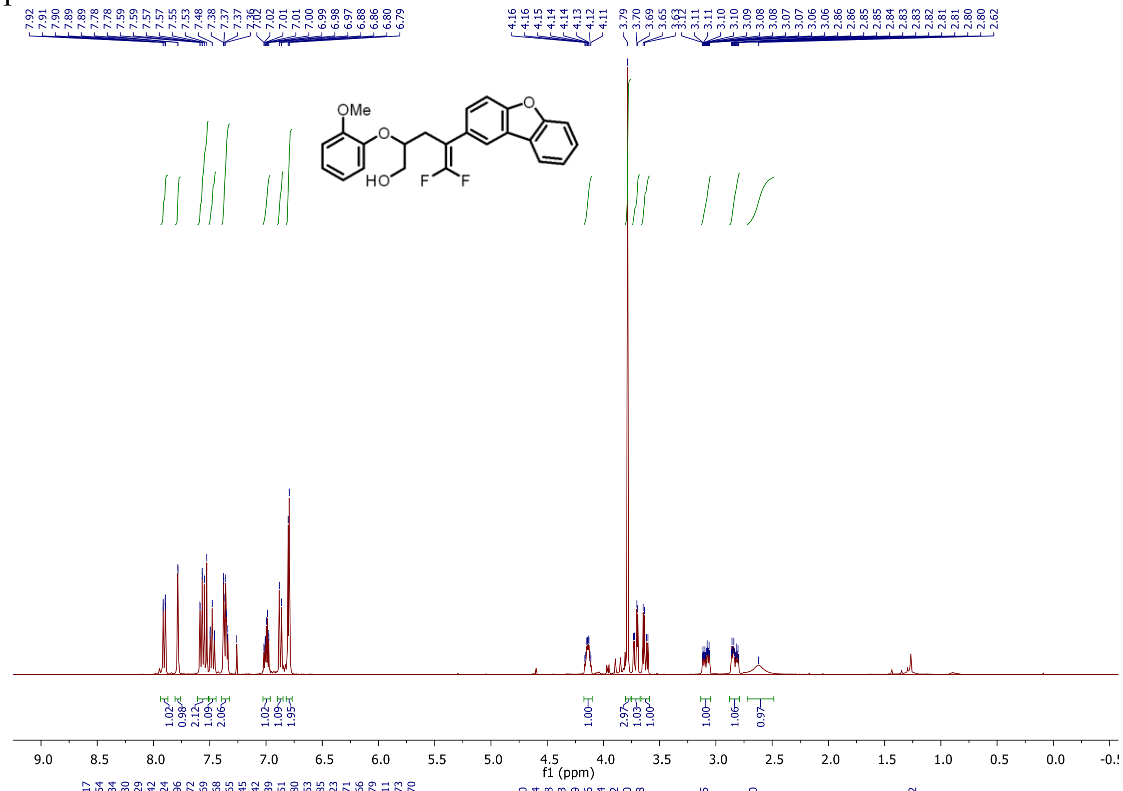

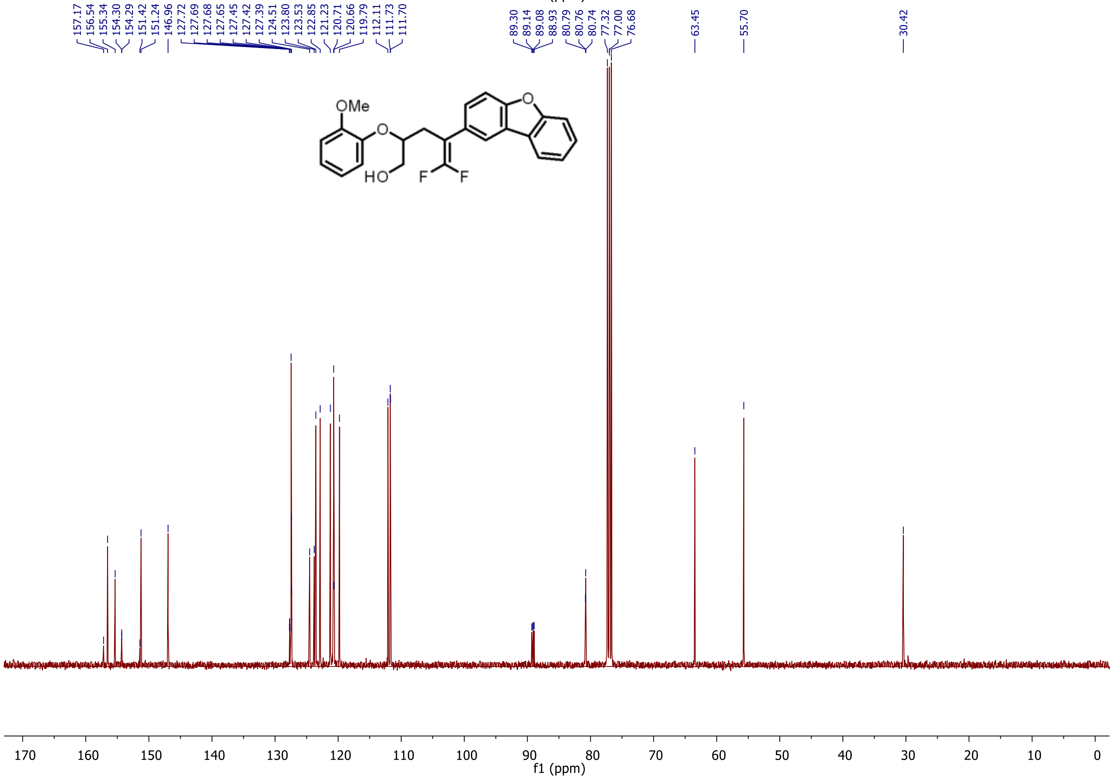

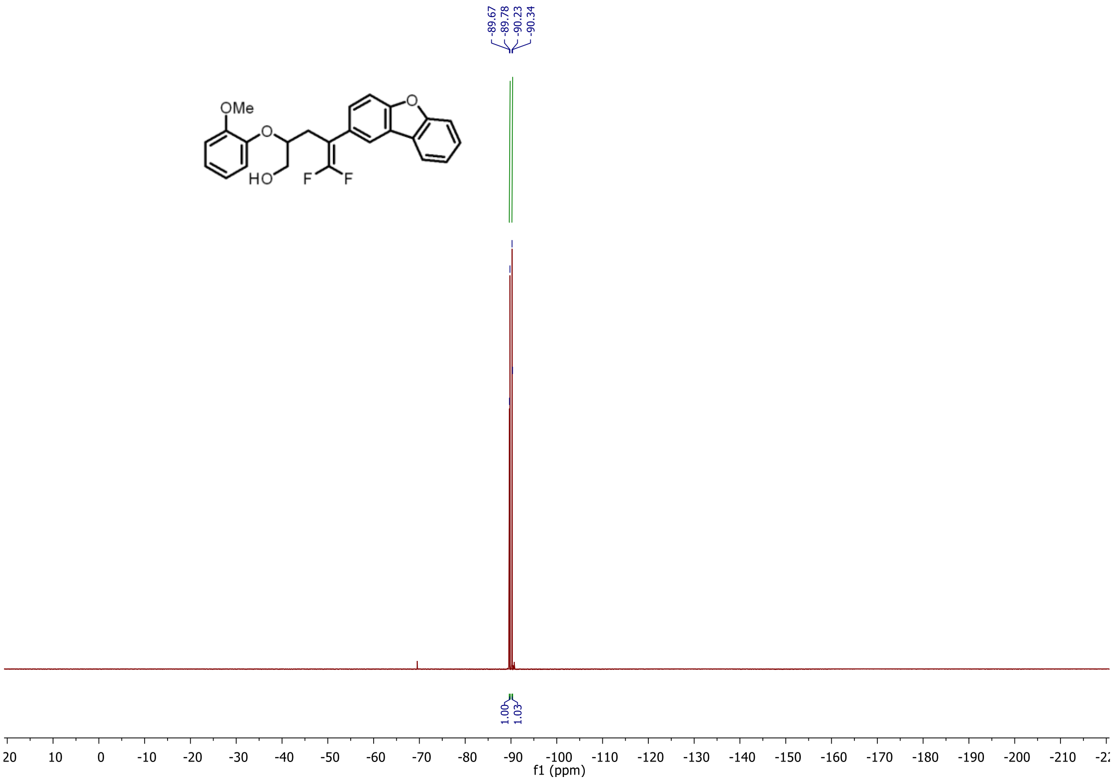

Compound **28**

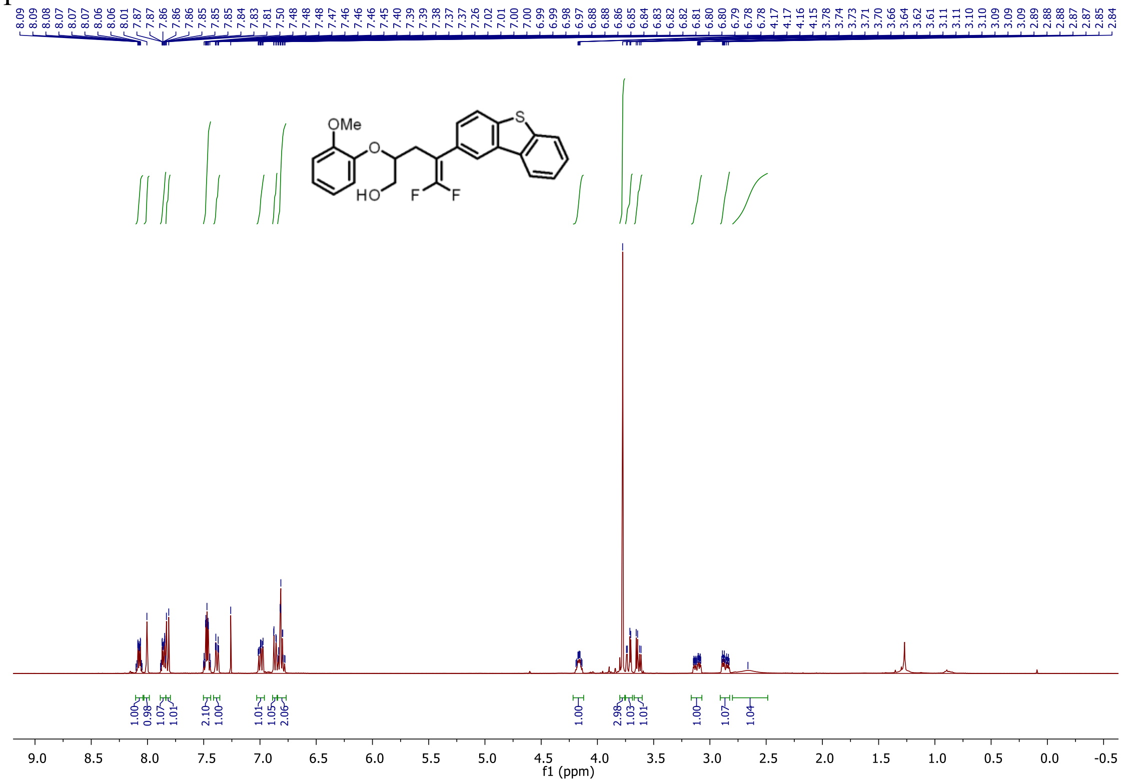

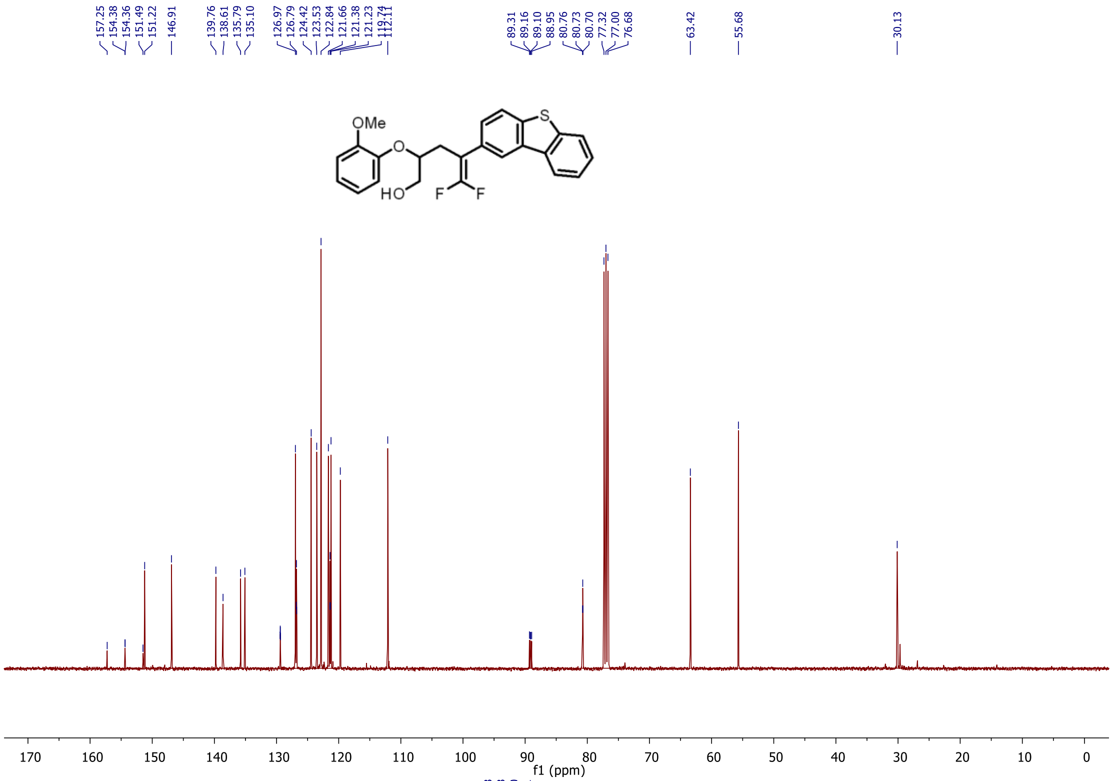
157.25
154.38
154.36
151.49
151.22
146.91
139.76
138.61
135.79
135.10
126.97
126.79
124.42
123.53
122.84
121.66
121.38
121.23
112.11
89.31
89.16
89.10
88.95
80.76
80.73
80.70
77.32
77.00
76.68
63.42
55.68
30.13
OMe
O
S
HO
F
F
f1 (ppm)

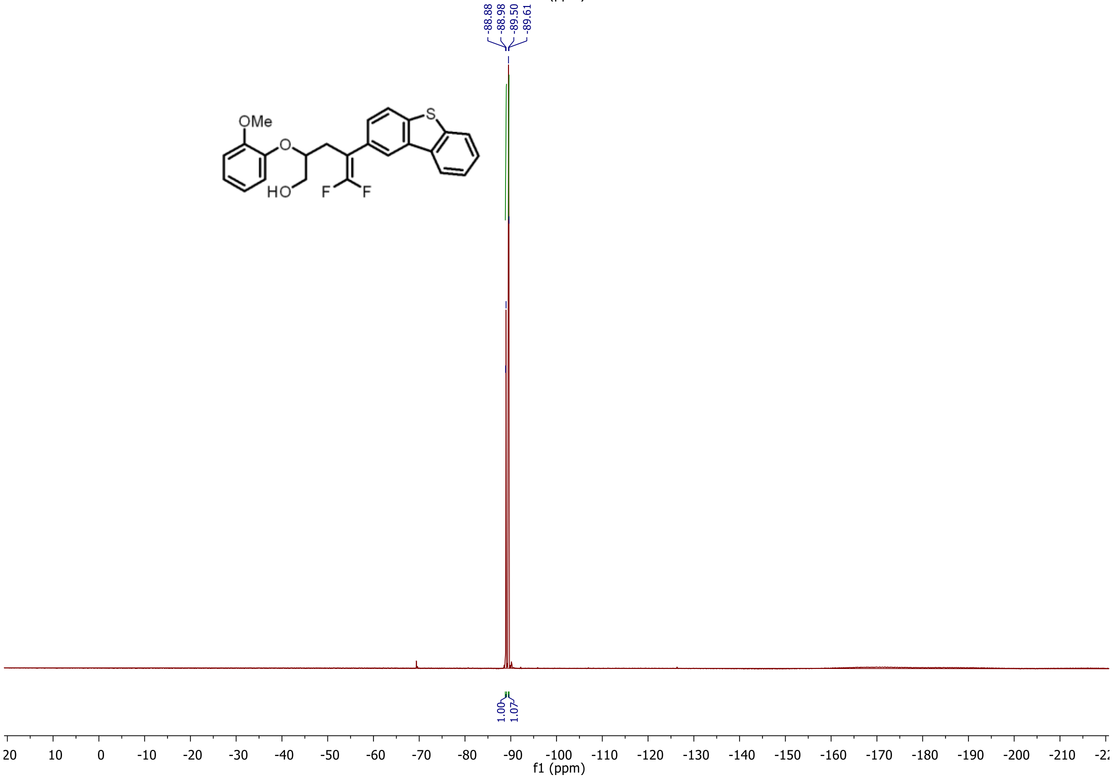
-88.88
-88.98
-89.50
-89.61
OMe
O
S
HO
F
F
1.00
1.07
f1 (ppm)

Compound **29**

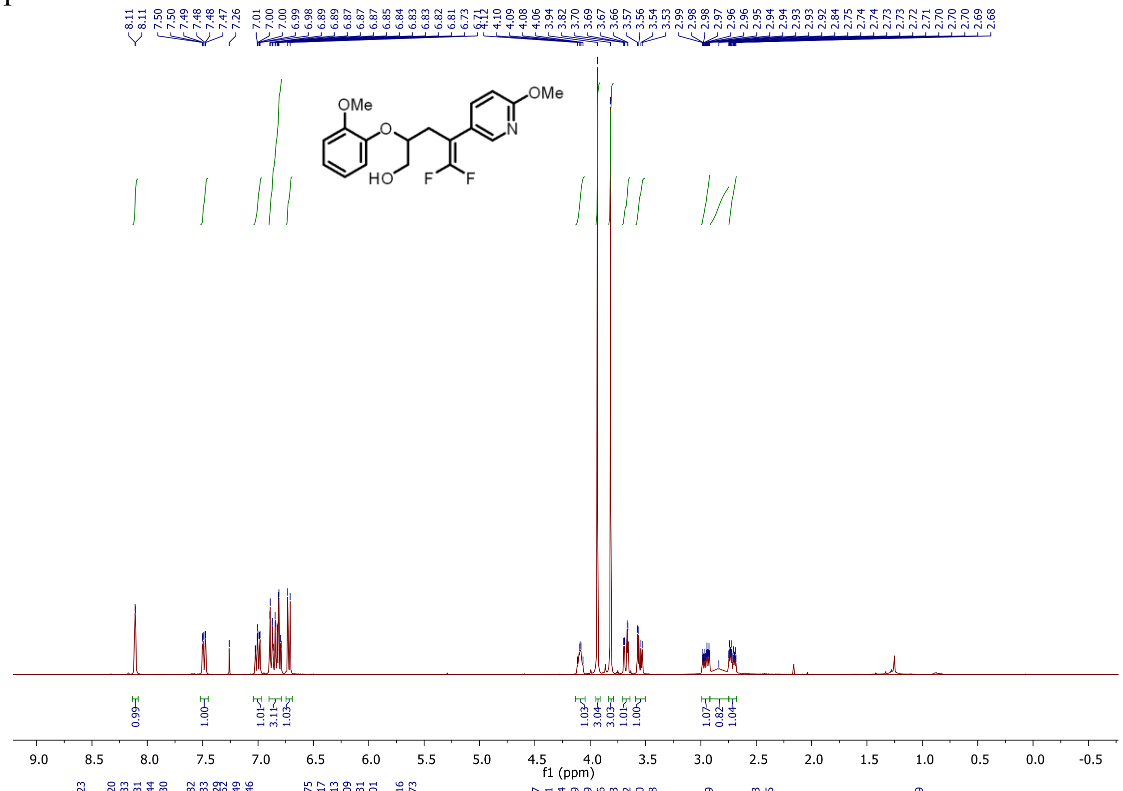

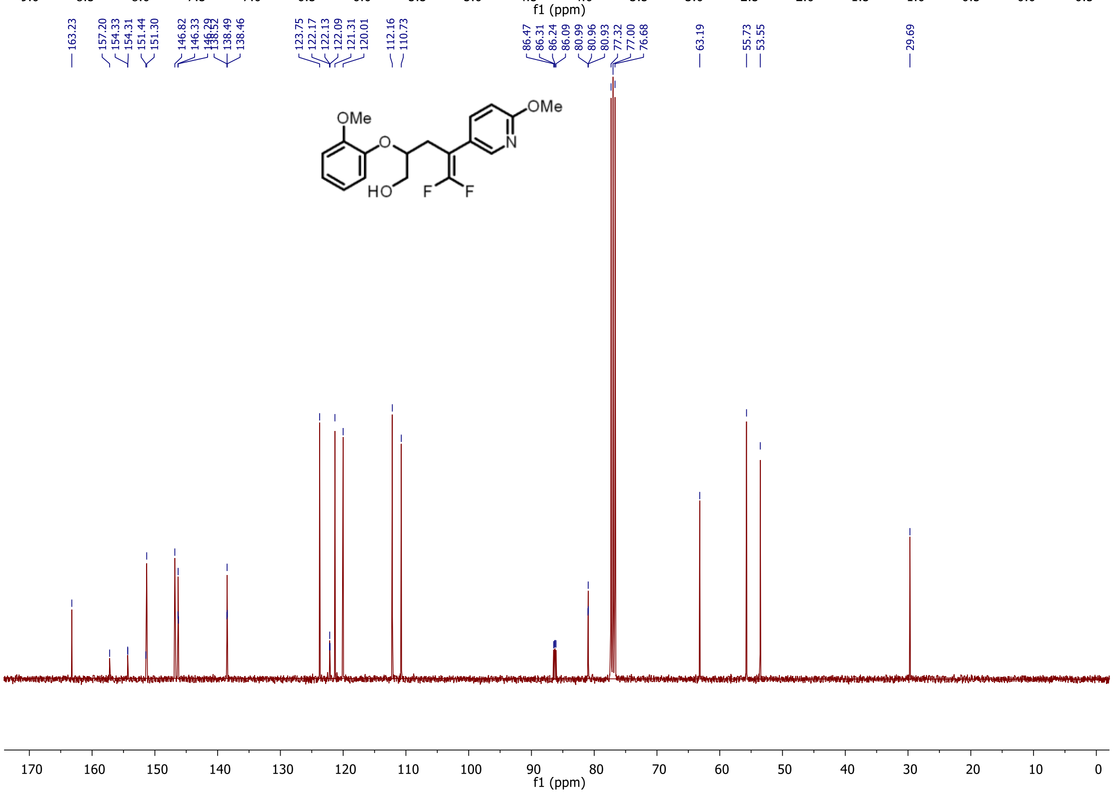

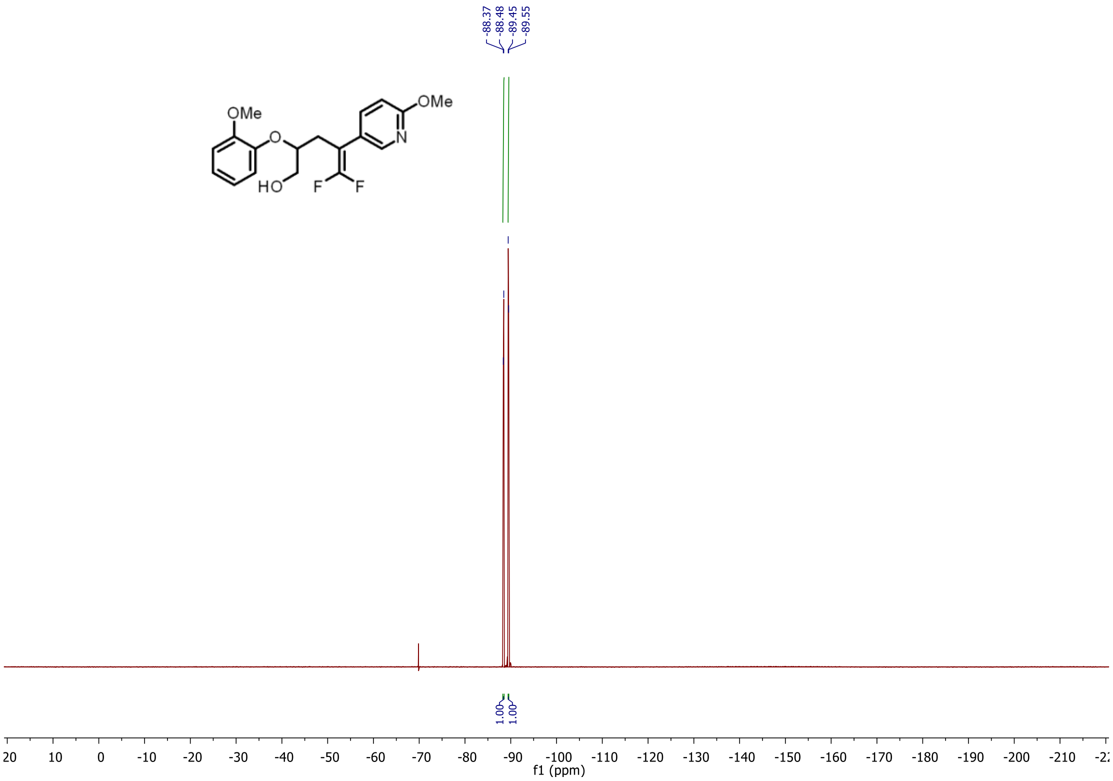

-88.37
-88.48
-89.45
-89.55
OMe
O
OMe
N
HO
F
F
1.00
1.00
f1 (ppm)

Compound **30**

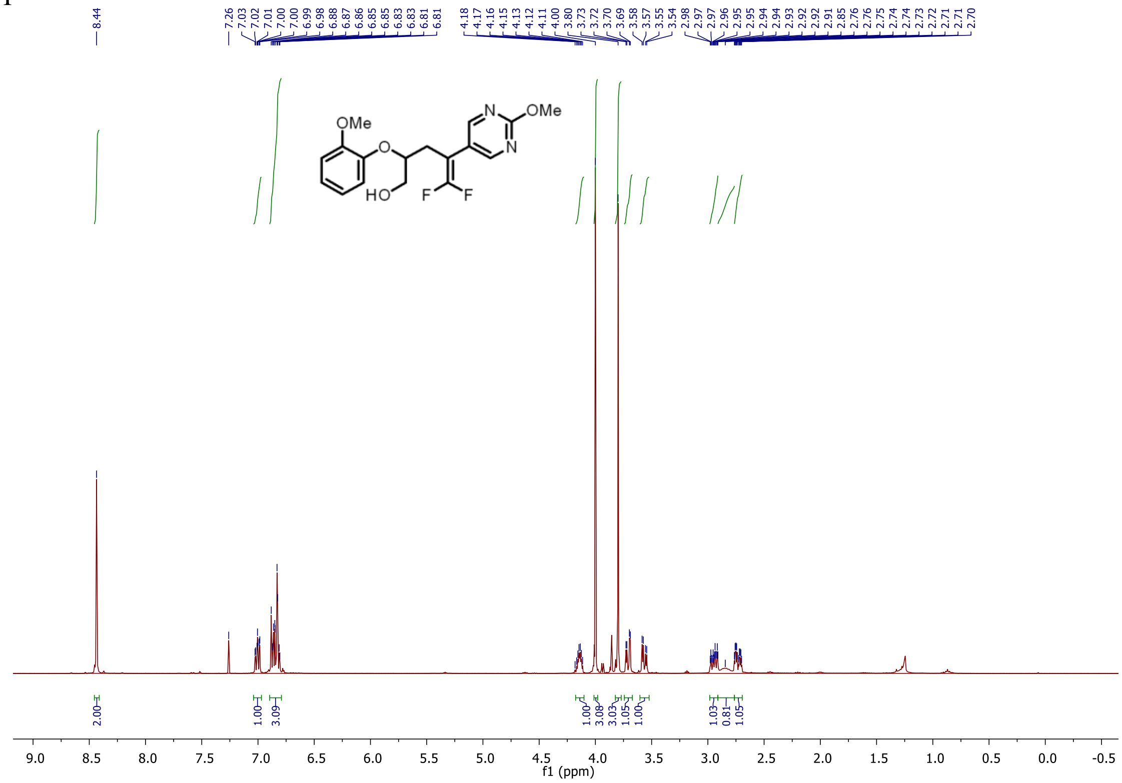

OMe
O
N
OMe
N
HO
F
F
2.00
1.00
3.09
1.00
3.08
3.03
1.05
1.00
1.03
0.81
1.05
f1 (ppm)

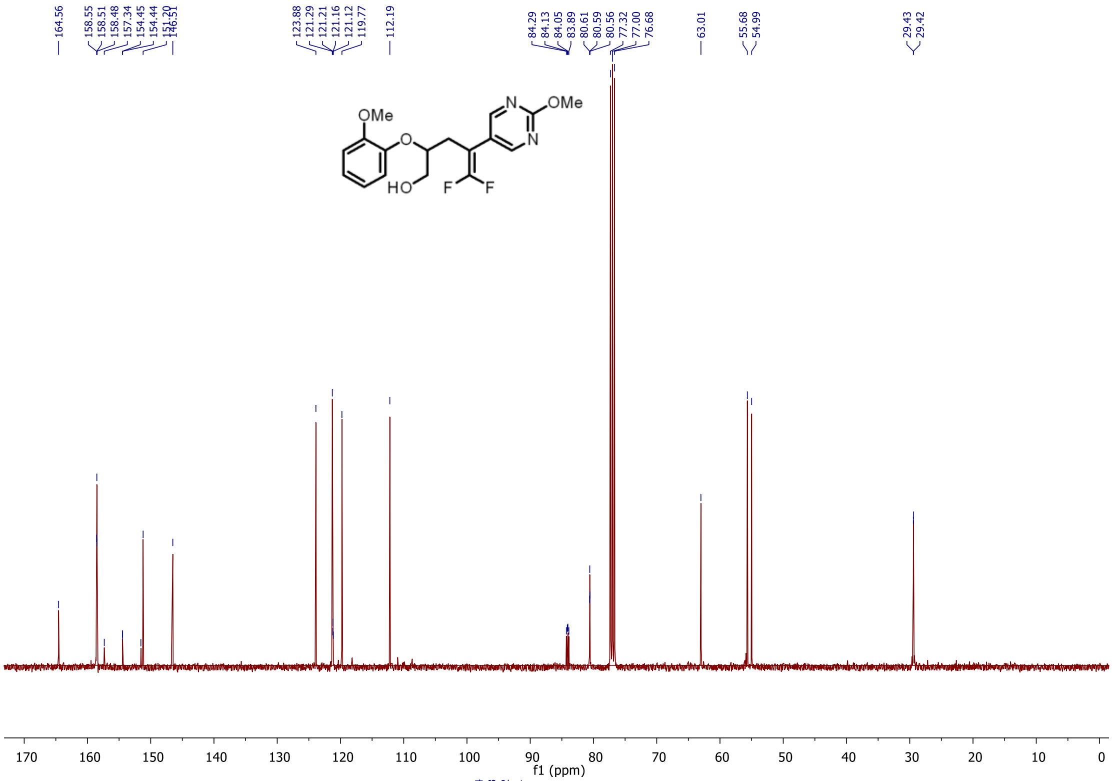
164.56
158.55
158.51
158.48
157.34
154.45
154.44
151.20
146.51
123.88
121.29
121.21
121.16
121.12
119.77
112.19
84.29
84.13
84.05
83.89
80.61
80.59
80.56
77.32
77.00
76.68
63.01
55.68
54.99
29.43
29.42
OMe
O
N
OMe
N
HO
F
F
170
160
150
140
130
120
110
100
90
80
70
60
50
40
30
20
10
0
f1 (ppm)

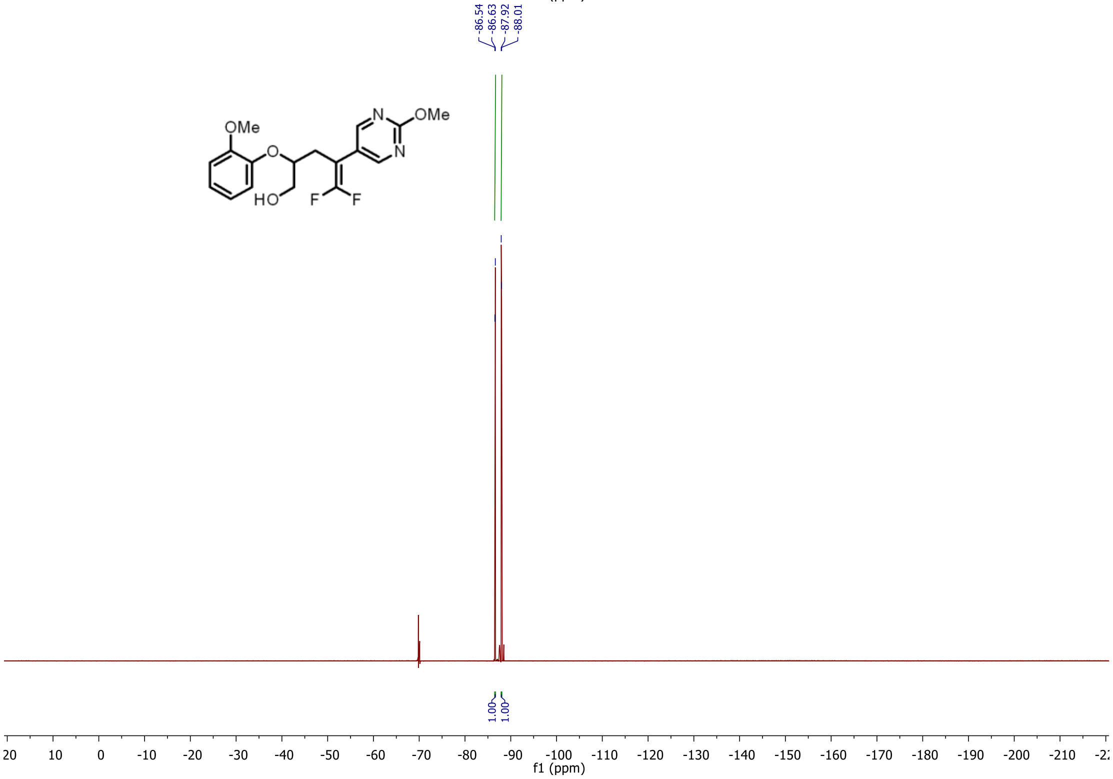
-86.54
-86.63
-87.92
-88.01
OMe
O
N
OMe
N
HO
F
F
1.00
1.00
20
10
0
-10
-20
-30
-40
-50
-60
-70
-80
-90
-100
-110
-120
-130
-140
-150
-160
-170
-180
-190
-200
-210
f1 (ppm)

Compound **31**

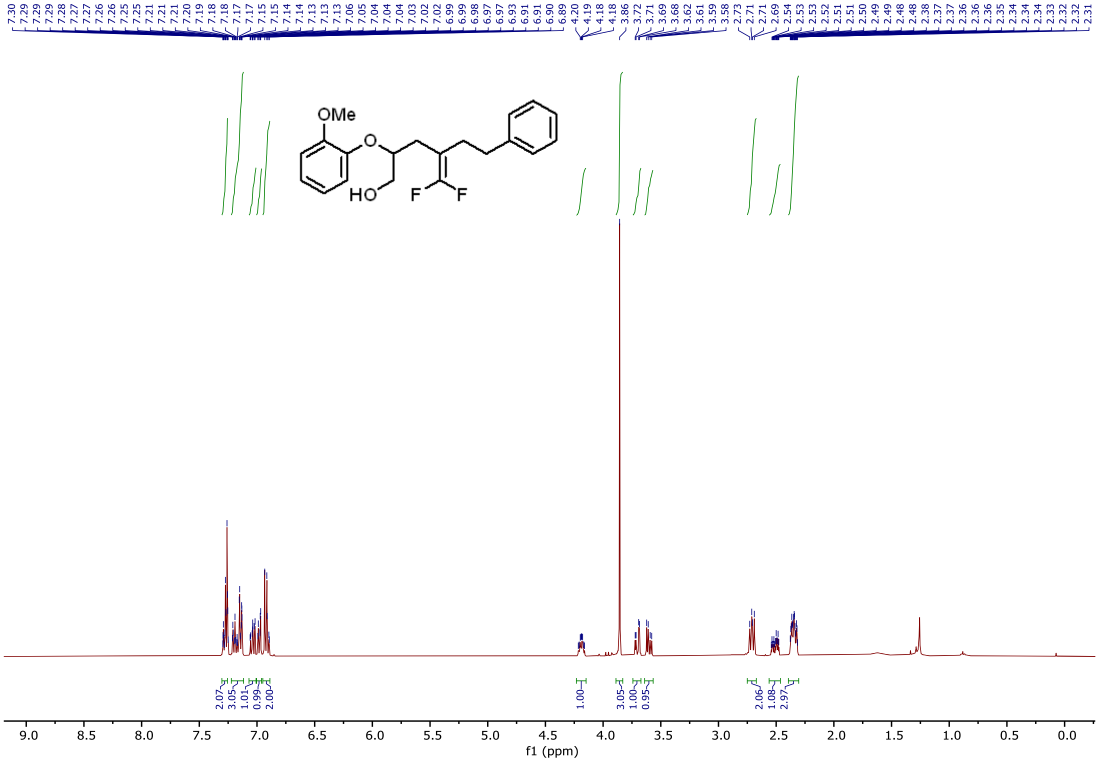

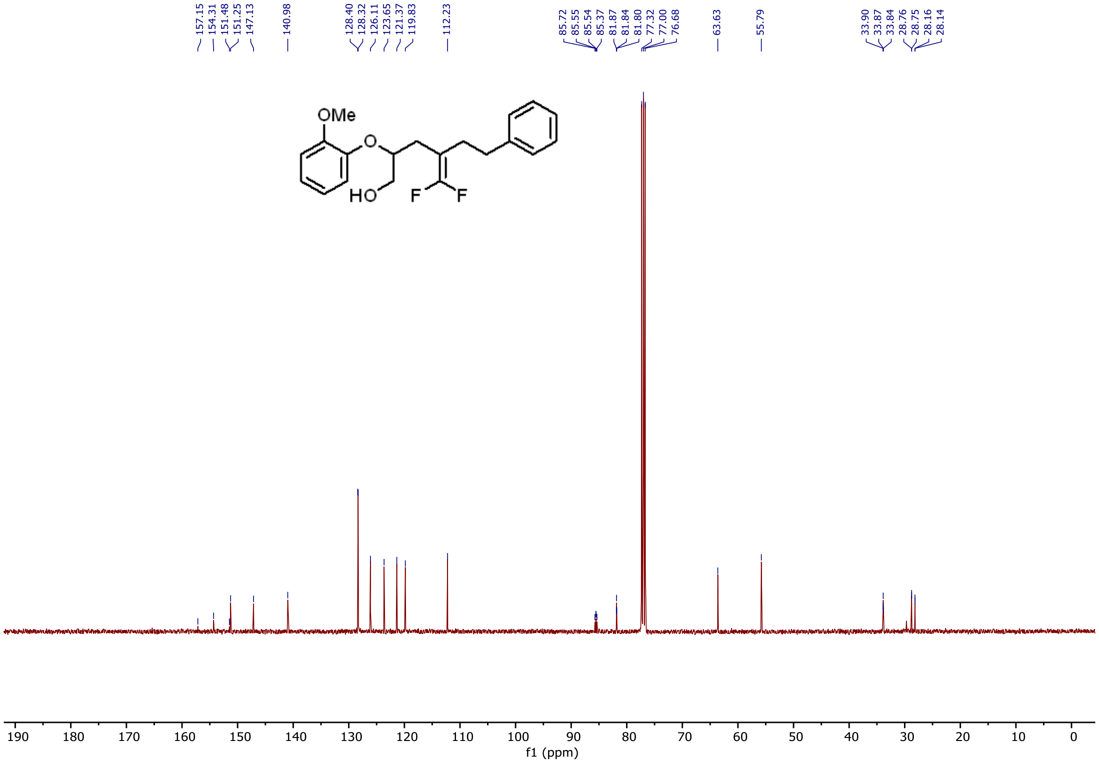

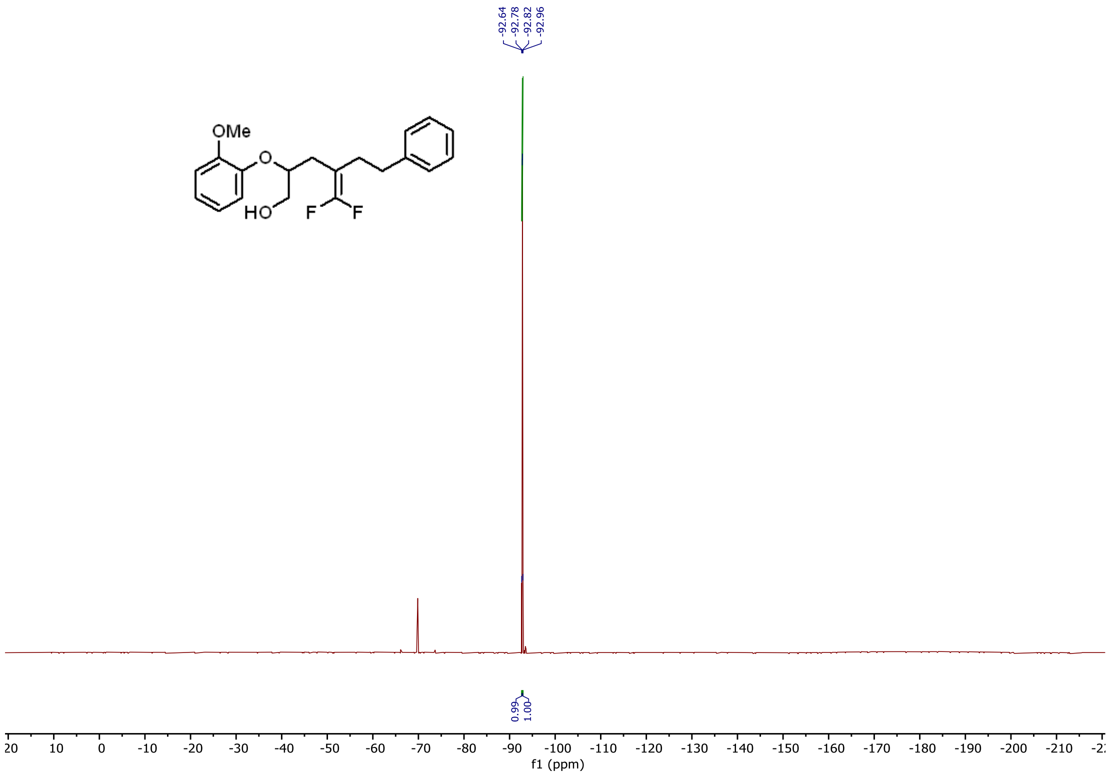
-92.64
-92.78
-92.82
-92.96
OMe
O
HO
F
F
0.99
1.00
f1 (ppm)

Compound **32**

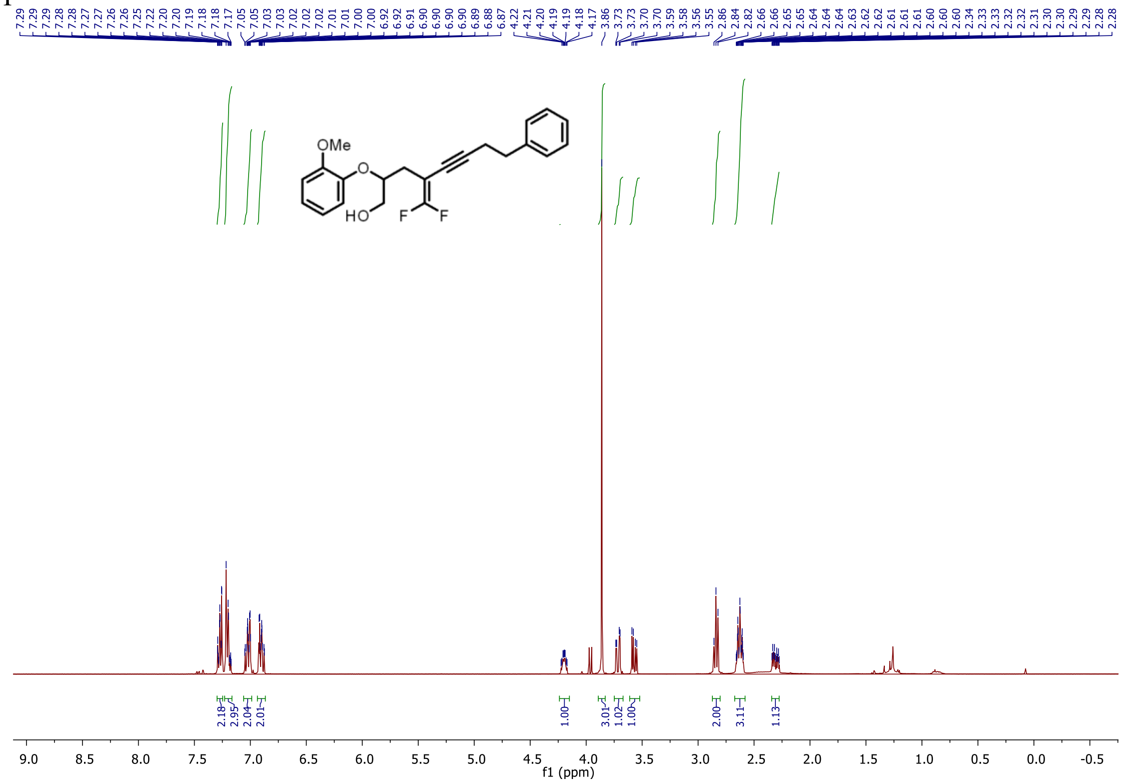
OMe
O
HO
F
F
2.18
2.95
2.04
2.01
1.00
3.01
1.02
1.00
2.00
3.11
1.13
f1 (ppm)

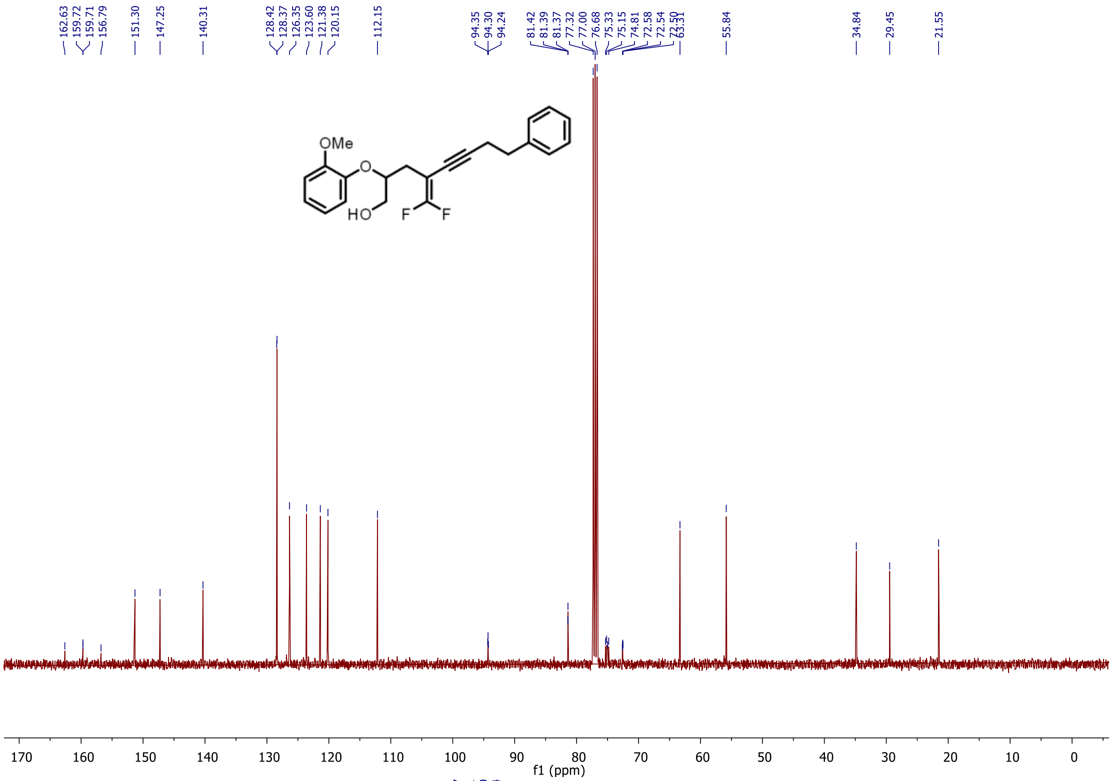

162.63
159.72
159.71
156.79
151.30
147.25
140.31
128.42
128.37
126.35
123.60
121.38
120.15
112.15
94.35
94.30
94.24
81.42
81.39
81.37
77.32
77.00
76.68
75.33
75.15
74.81
72.58
72.54
72.50
63.31
55.84
34.84
29.45
21.55
OMe
O
HO
F
F
170 160 150 140 130 120 110 100 90 80 70 60 50 40 30 20 10 0
f1 (ppm)

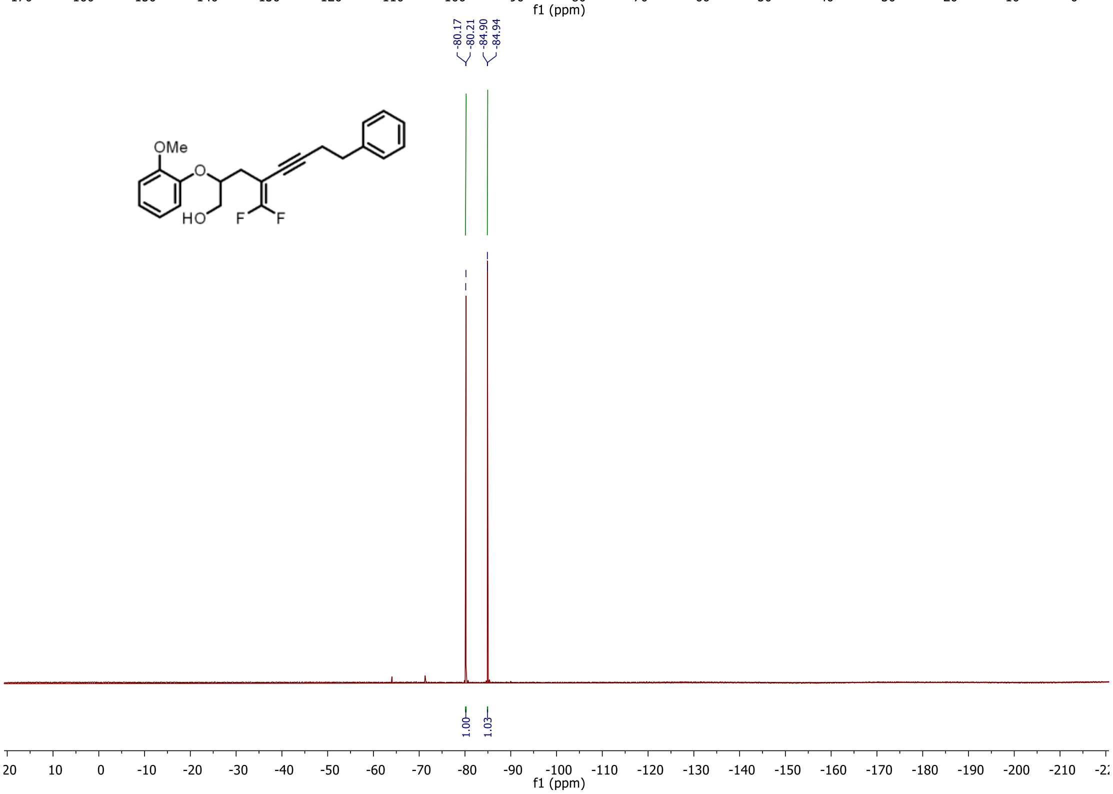

-80.17
-80.21
-84.90
-84.94
OMe
O
HO
F
F
1.00
1.03
20 10 0 -10 -20 -30 -40 -50 -60 -70 -80 -90 -100 -110 -120 -130 -140 -150 -160 -170 -180 -190 -200 -210
f1 (ppm)

Compound **33**

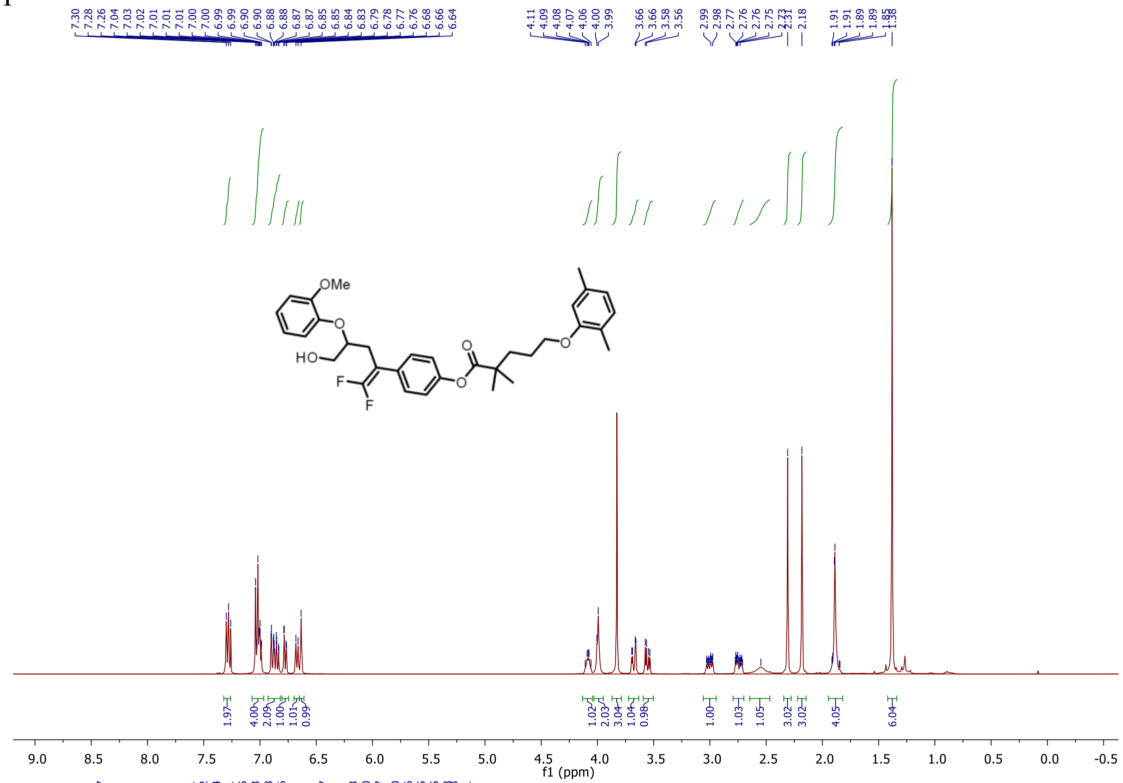

7.30 7.28 7.26 7.04 7.03 7.02 7.01 7.01 7.00 6.99 6.99 6.90 6.90 6.88 6.88 6.87 6.87 6.85 6.84 6.83 6.79 6.78 6.77 6.76 6.68 6.66 6.64
4.11 4.09 4.08 4.07 4.06 4.00 3.99
3.66 3.66 3.58 3.56
2.99 2.98 2.77 2.76 2.76 2.75
2.18
1.91 1.91
1.38
OMe
O
HO
F
F
O
O
O
1.97 4.00 2.09 1.00 1.01 0.99
1.02 2.03 3.04 1.04 0.98
1.00 1.03 1.05 3.02 3.02 4.05 6.04
9.0 8.5 8.0 7.5 7.0 6.5 6.0 5.5 5.0 4.5 4.0 3.5 3.0 2.5 2.0 1.5 1.0 0.5 0.0 -0.5
f1 (ppm)

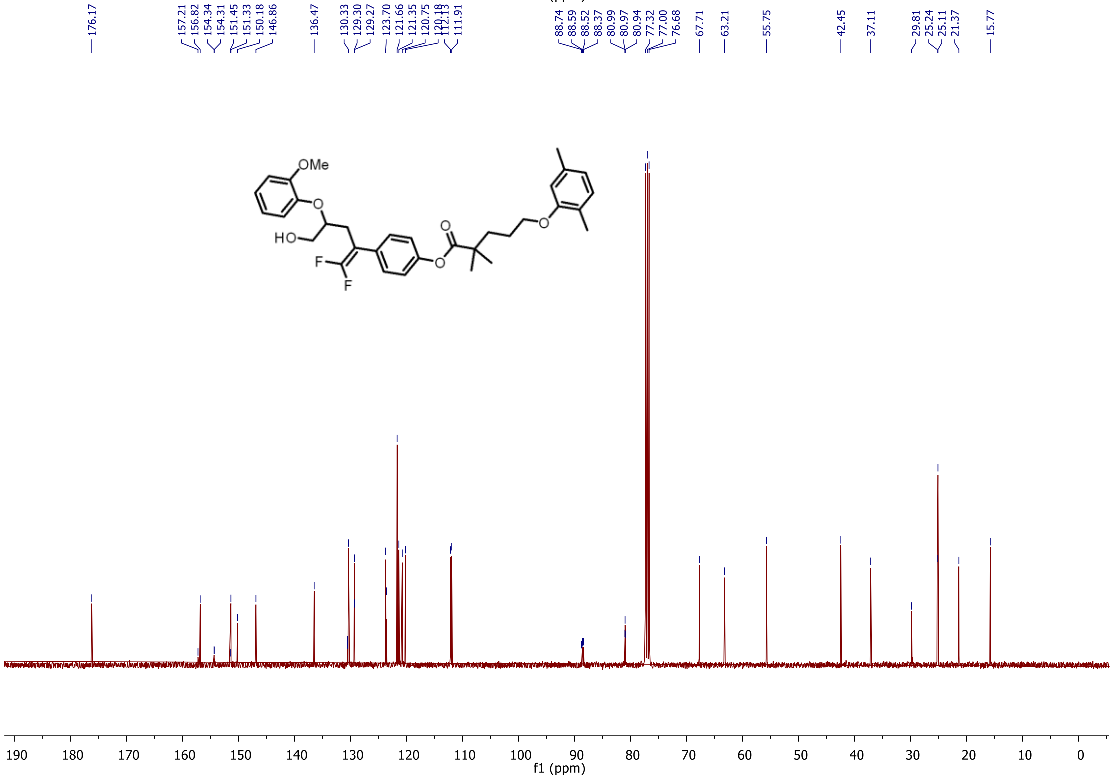

176.17
157.21 156.82 154.34 154.31 151.45 151.33 150.18 146.86
136.47
130.33 129.30 129.27
123.70 121.66 121.35 120.75 111.91
88.74 88.59 88.52 88.37 80.99 80.97 80.94 77.32 77.00 76.68
67.71
63.21
55.75
42.45
37.11
29.81 25.24 25.11 21.37
15.77
OMe
O
HO
F
F
O
O
O
190 180 170 160 150 140 130 120 110 100 90 80 70 60 50 40 30 20 10 0
f1 (ppm)

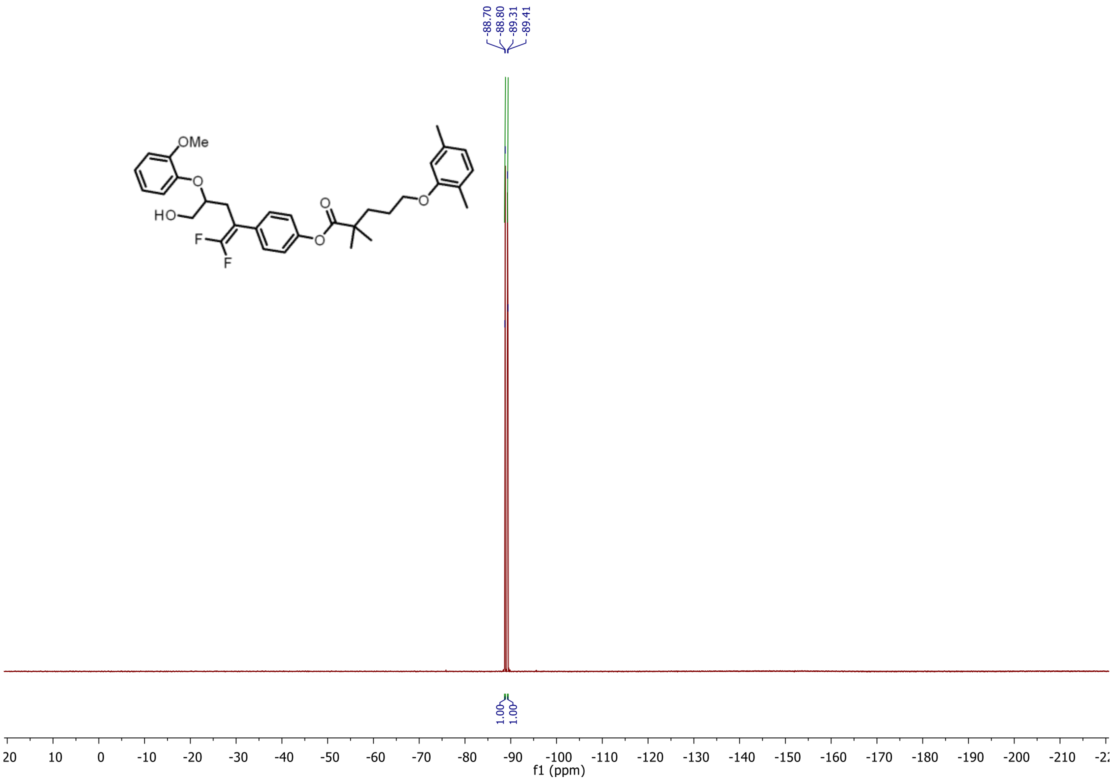

-88.70
-88.80
-89.31
-89.41
OMe
O
HO
F
F
O
O
O
1.00
1.00
f1 (ppm)

Compound **35**

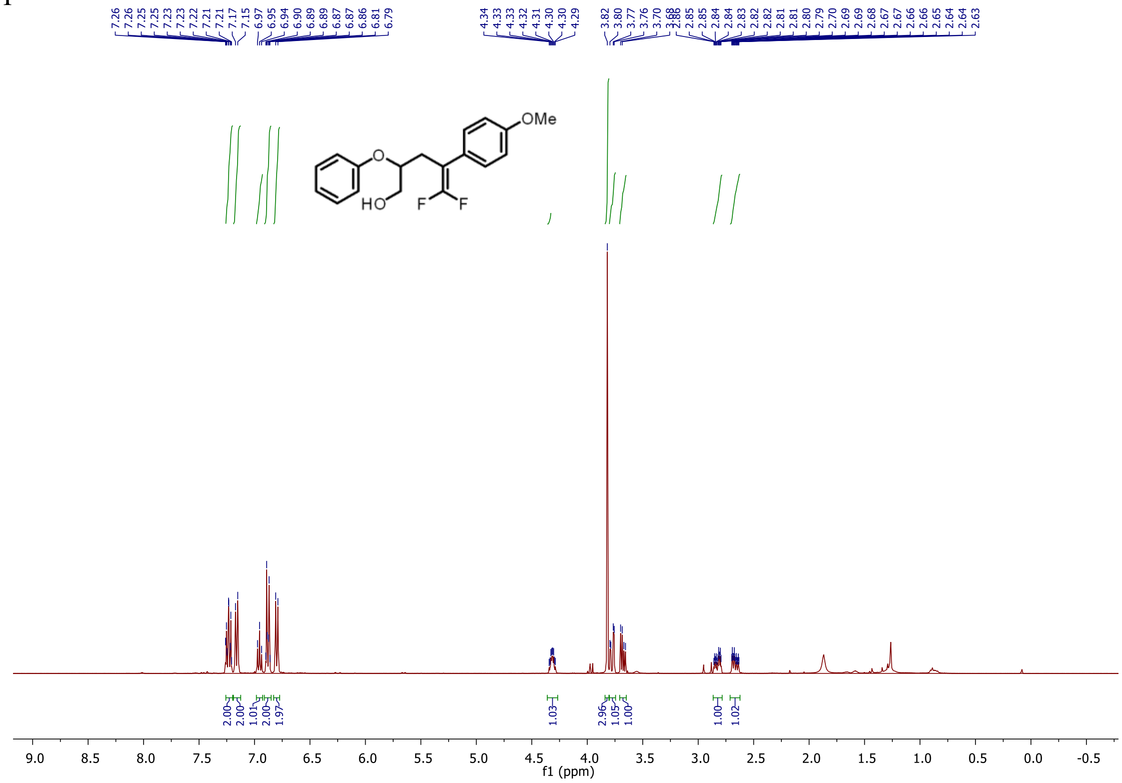

7.26 7.26 7.25 7.25 7.23 7.22 7.21 7.21 7.17 7.15 6.97 6.95 6.94 6.90 6.89 6.89 6.87 6.86 6.81 6.79
4.34 4.33 4.33 4.32 4.31 4.30 4.30 4.29
3.82 3.80 3.77 3.76 3.70 3.68 2.88 2.85 2.85 2.84 2.84 2.83 2.82 2.82 2.81 2.81 2.80 2.79 2.70 2.69 2.69 2.68 2.67 2.67 2.66 2.65 2.64 2.63
OMe
O
HO
F
F
2.00 2.00 1.01 2.00 1.97
1.03
2.96 1.05 1.00
1.00 1.02
f1 (ppm)

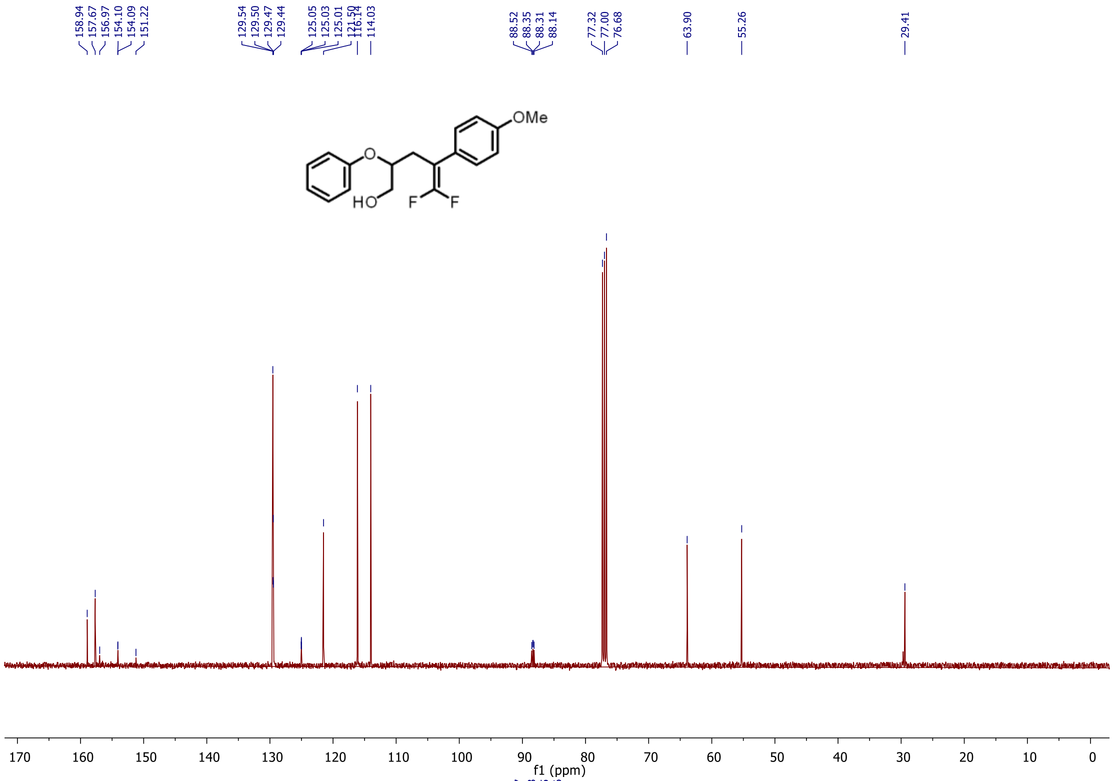
158.94
157.67
156.97
154.10
154.09
151.22
129.54
129.50
129.47
129.44
125.05
125.03
125.01
114.03
88.52
88.35
88.31
88.14
77.32
77.00
76.68
63.90
55.26
29.41
OMe
O
HO
F
F
170 160 150 140 130 120 110 100 90 80 70 60 50 40 30 20 10 0
f1 (ppm)

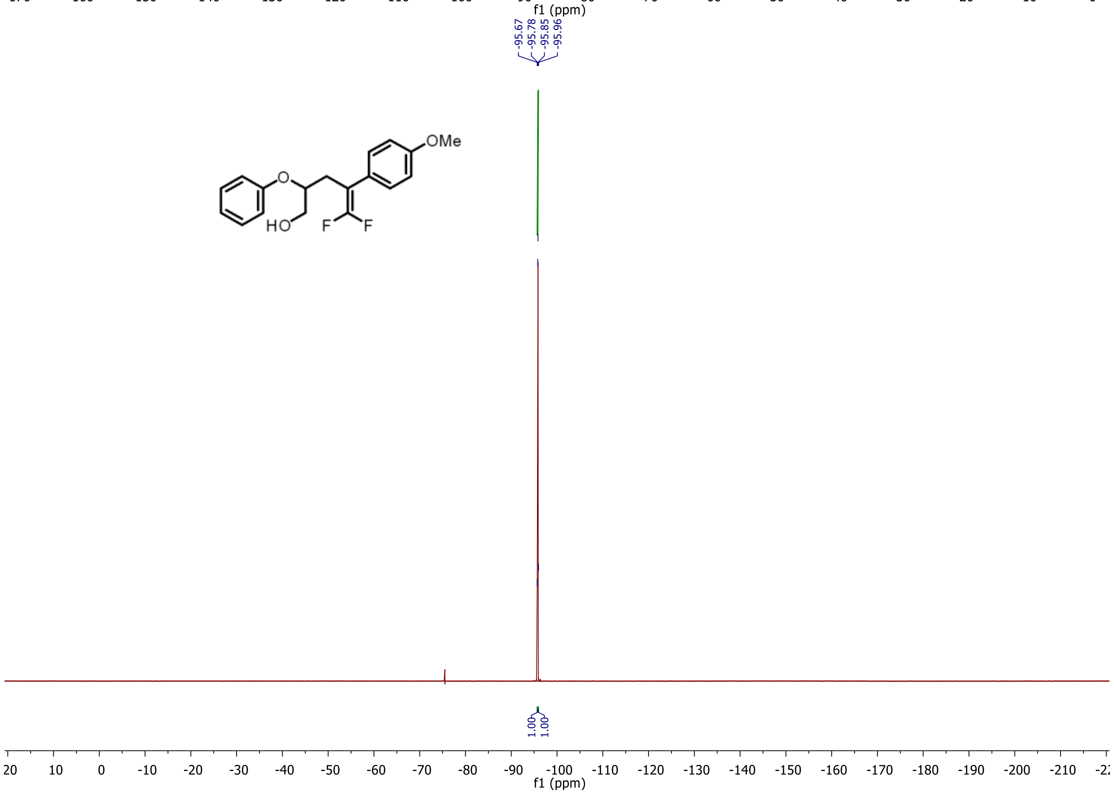
-95.67
-95.78
-95.85
-95.96
OMe
O
HO
F
F
1.00
1.00
20 10 0 -10 -20 -30 -40 -50 -60 -70 -80 -90 -100 -110 -120 -130 -140 -150 -160 -170 -180 -190 -200 -210
f1 (ppm)

Compound **37**

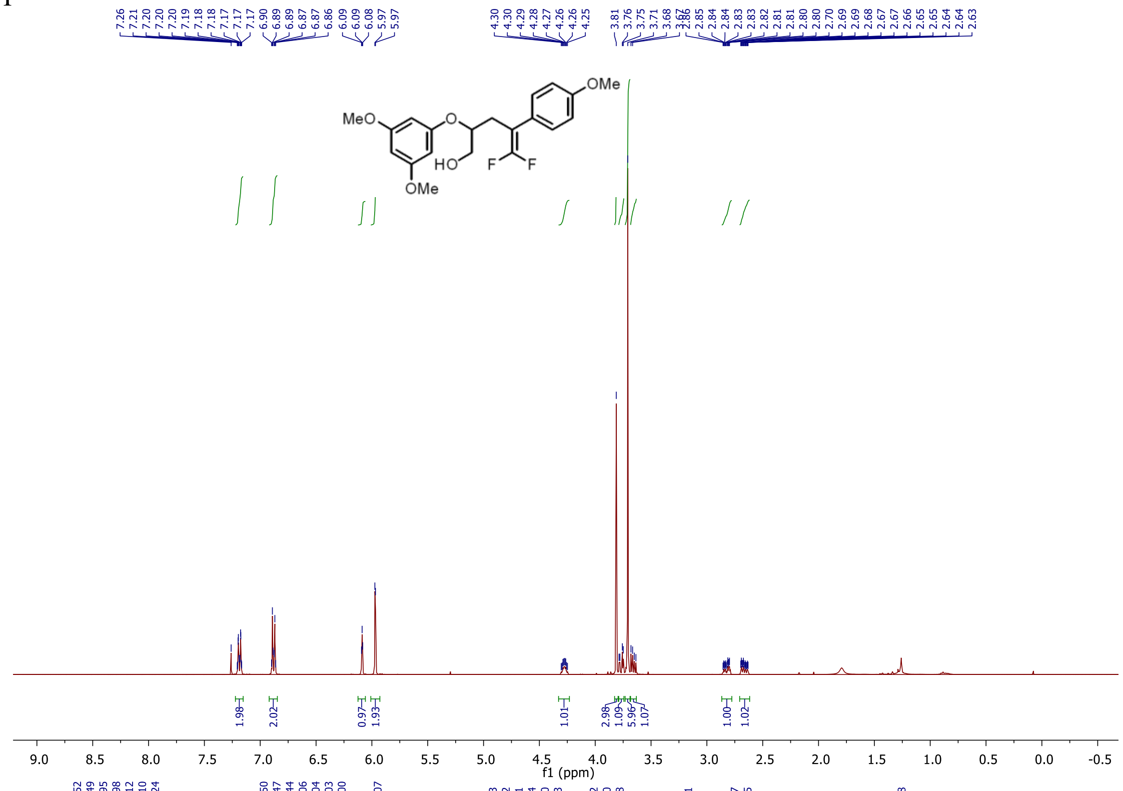

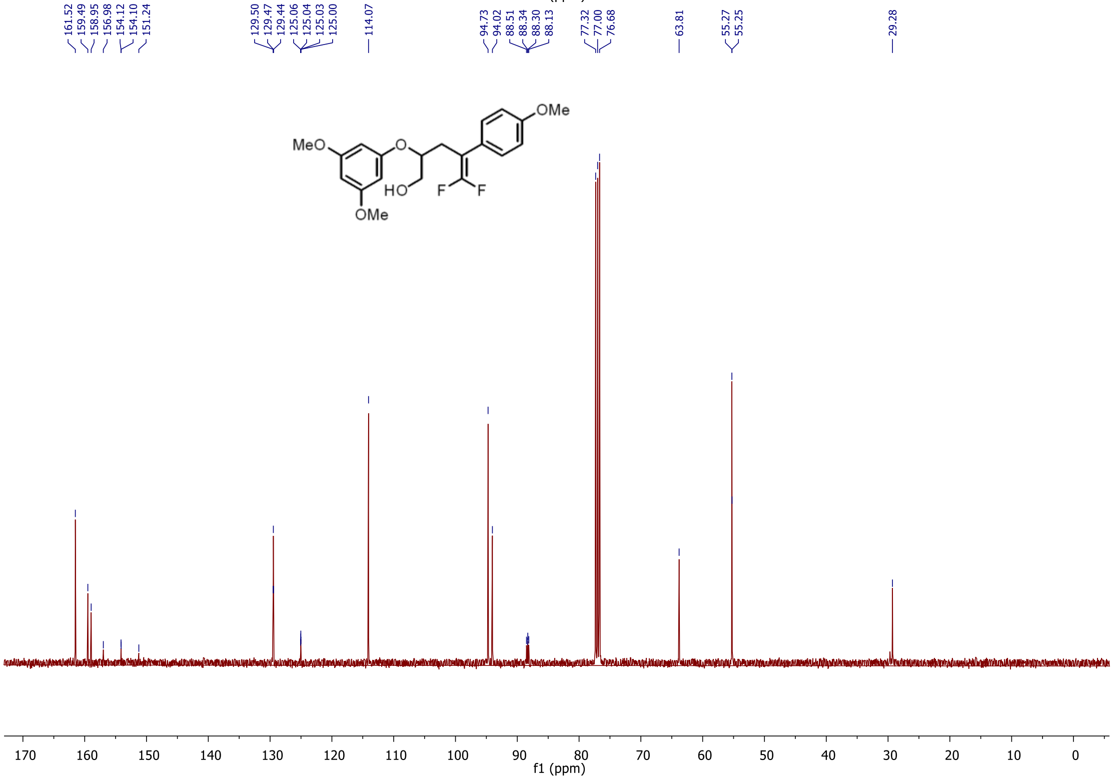

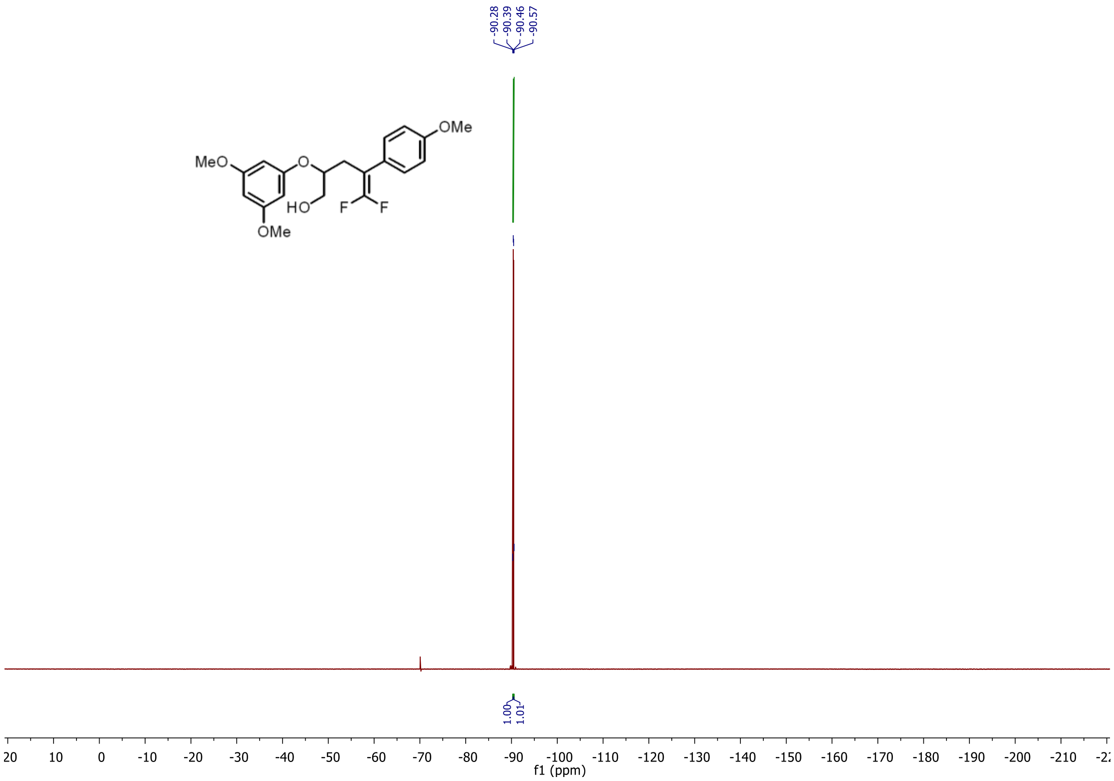

-90.28
-90.39
-90.46
-90.57
OMe
MeO
O
HO
F
F
OMe
1.00
1.01
20 10 0 -10 -20 -30 -40 -50 -60 -70 -80 -90 -100 -110 -120 -130 -140 -150 -160 -170 -180 -190 -200 -210
f1 (ppm)

Compound **39**

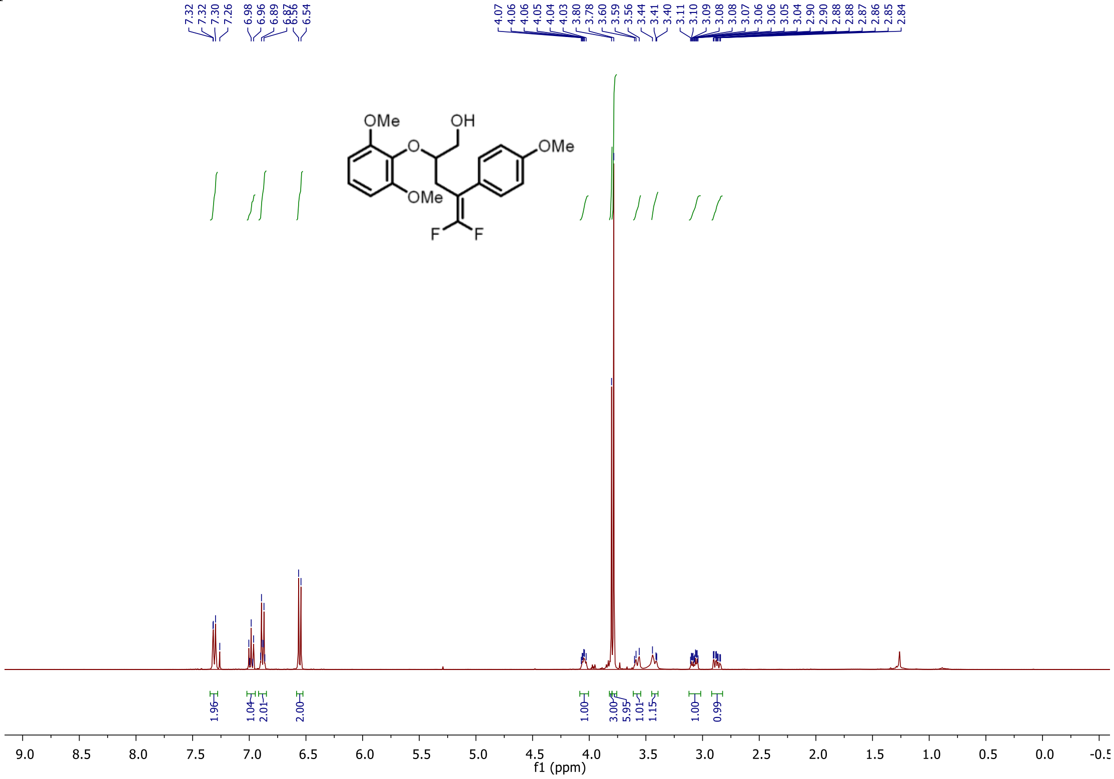

7.32
7.32
7.30
7.26
6.98
6.96
6.89
6.87
6.56
6.54
4.07
4.06
4.06
4.05
4.04
4.03
3.80
3.78
3.60
3.59
3.56
3.44
3.41
3.40
3.11
3.10
3.09
3.08
3.08
3.07
3.06
3.06
3.05
3.04
2.90
2.90
2.88
2.88
2.87
2.86
2.85
2.84
OMe
OH
O
OMe
OMe
F
F
1.96
1.04
2.01
2.00
1.00
3.00
5.95
1.01
1.15
1.00
0.99
9.0 8.5 8.0 7.5 7.0 6.5 6.0 5.5 5.0 4.5 4.0 3.5 3.0 2.5 2.0 1.5 1.0 0.5 0.0 -0.5
f1 (ppm)

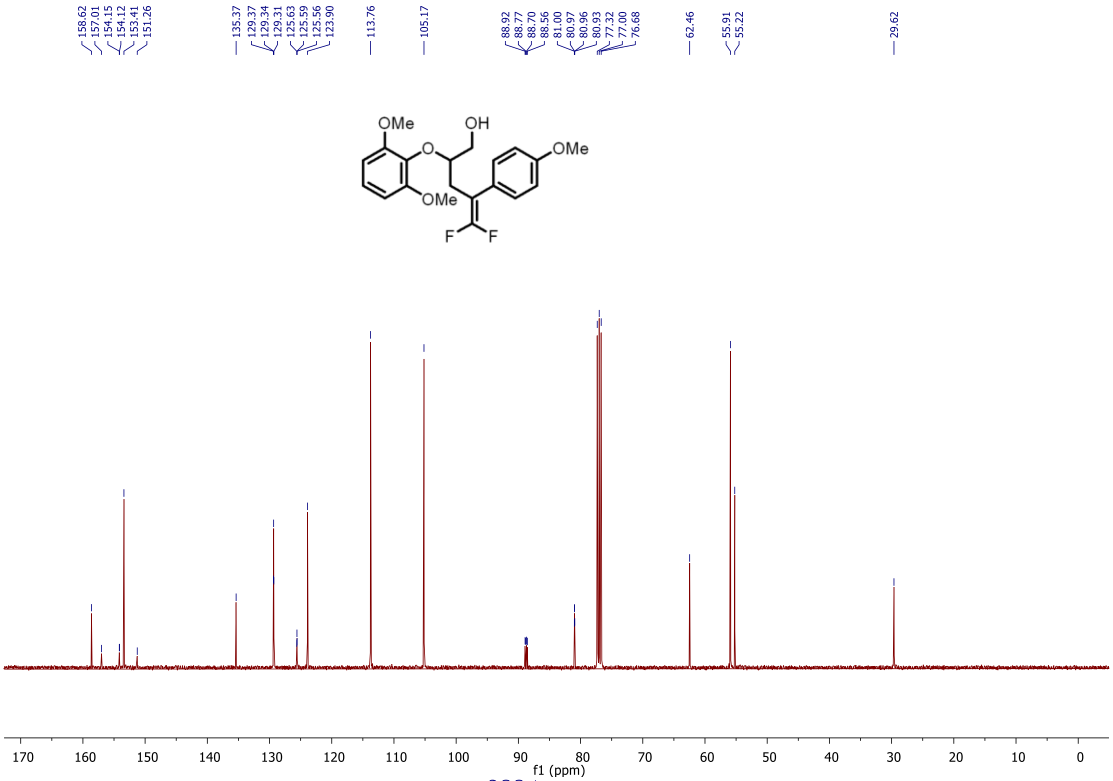
158.62
157.01
154.15
154.12
153.41
151.26
135.37
129.37
129.34
129.31
125.63
125.59
125.56
123.90
113.76
105.17
88.92
88.77
88.70
88.56
81.00
80.97
80.96
80.93
77.32
77.00
76.68
62.46
55.91
55.22
29.62
OMe
OH
O
OMe
OMe
F
F
170 160 150 140 130 120 110 100 90 80 70 60 50 40 30 20 10 0
f1 (ppm)

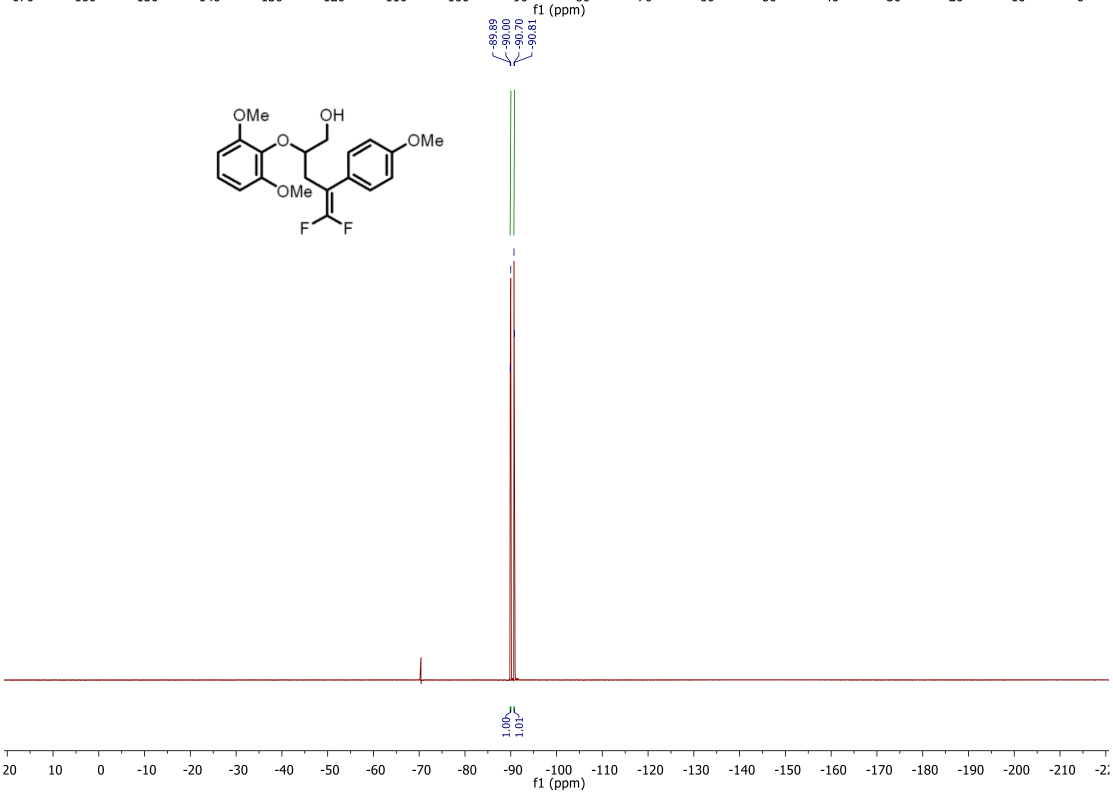
-89.89
-90.00
-90.70
-90.81
OMe
OH
O
OMe
OMe
F
F
1.00
1.01
20 10 0 -10 -20 -30 -40 -50 -60 -70 -80 -90 -100 -110 -120 -130 -140 -150 -160 -170 -180 -190 -200 -210
f1 (ppm)

Compound **41**

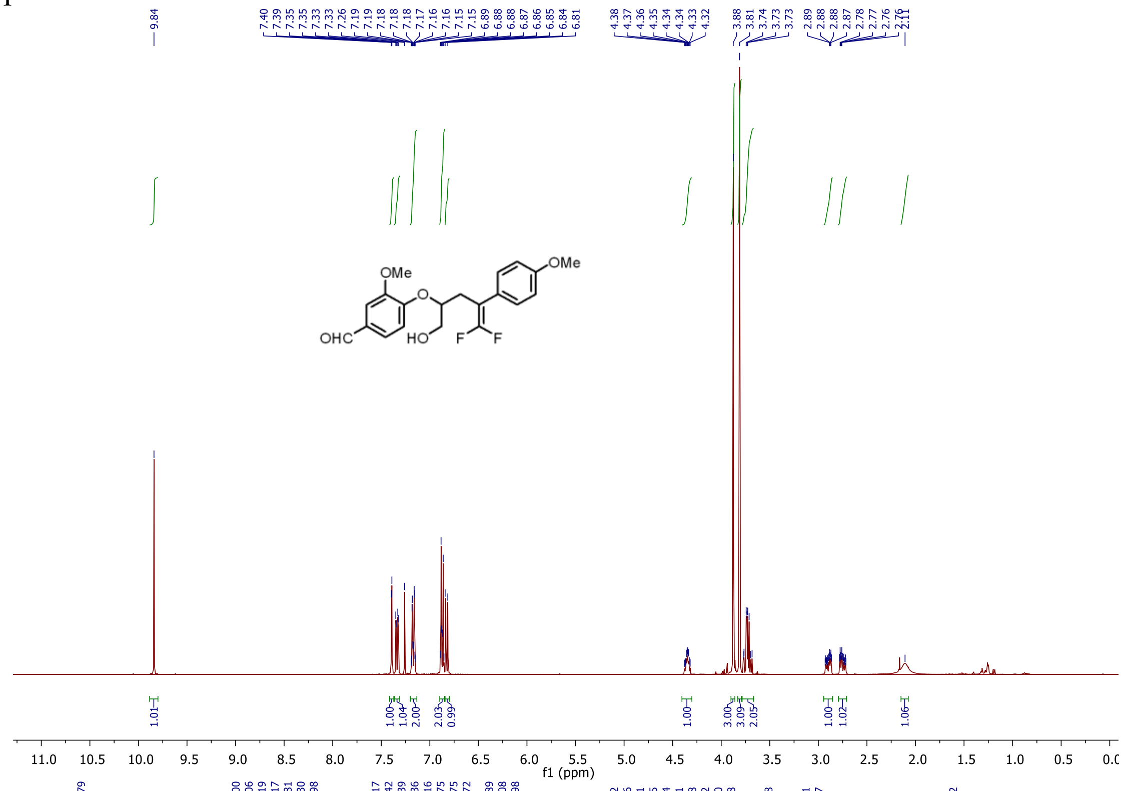

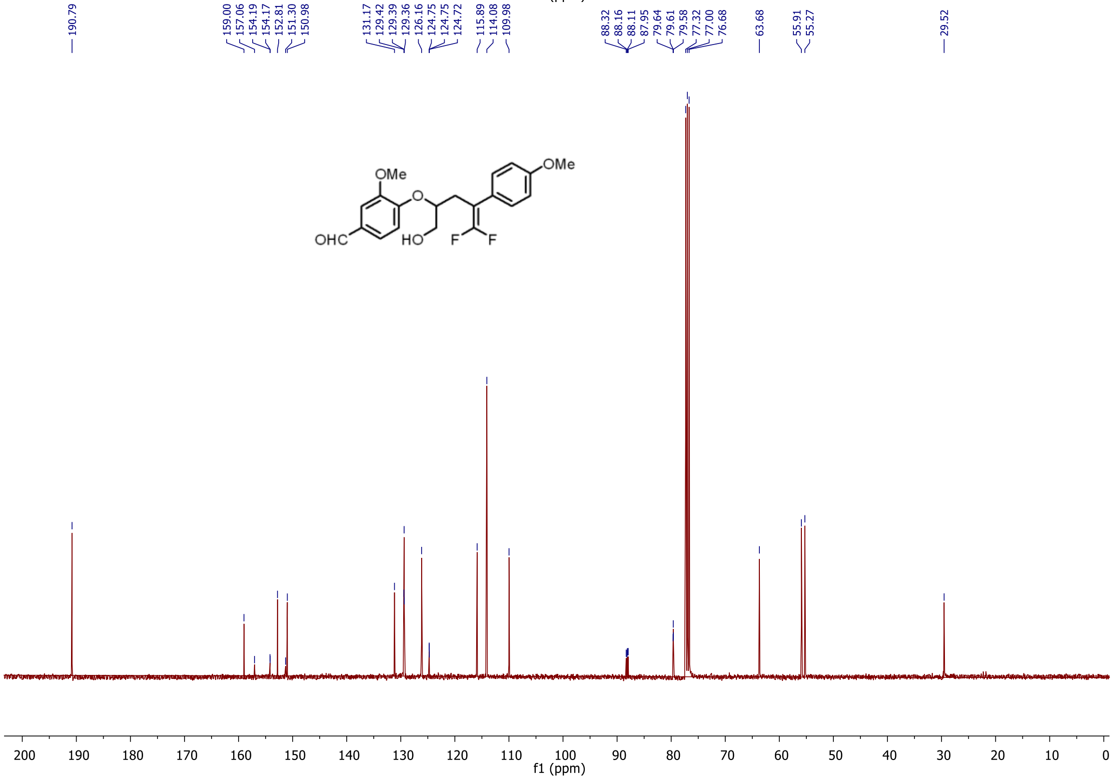

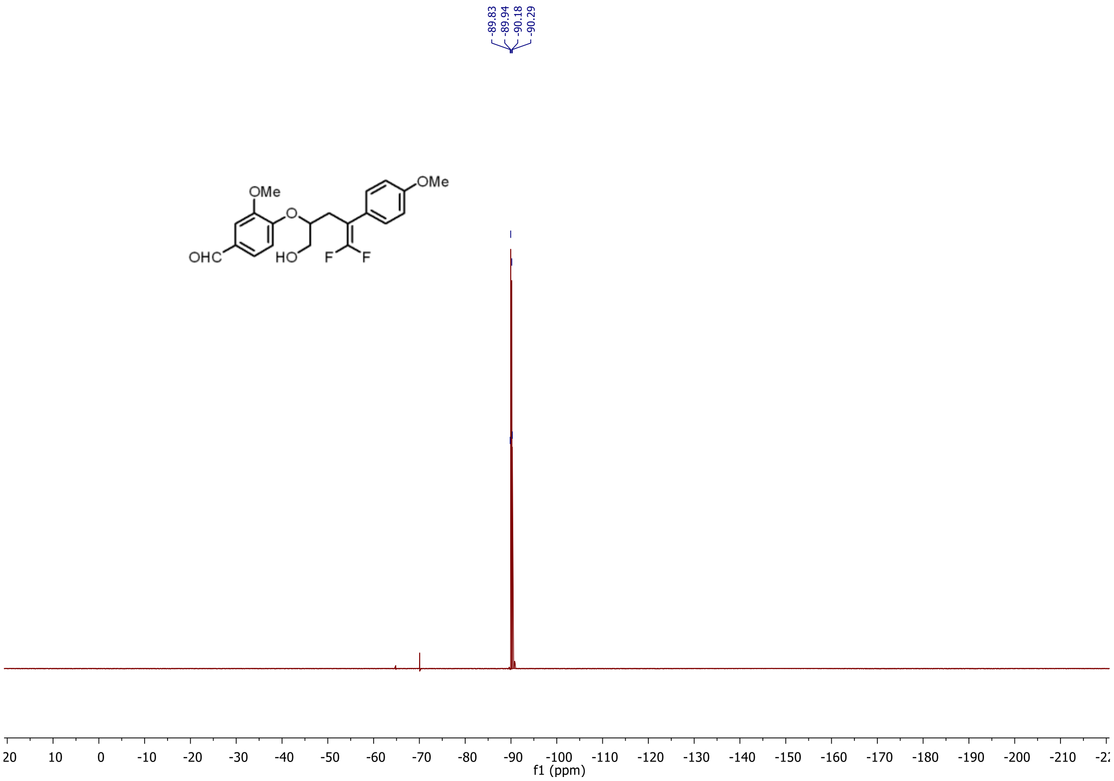
-89.83
-89.94
-90.18
-90.29
OMe
O
OMe
OHC
HO
F
F
f1 (ppm)

Compound **47**

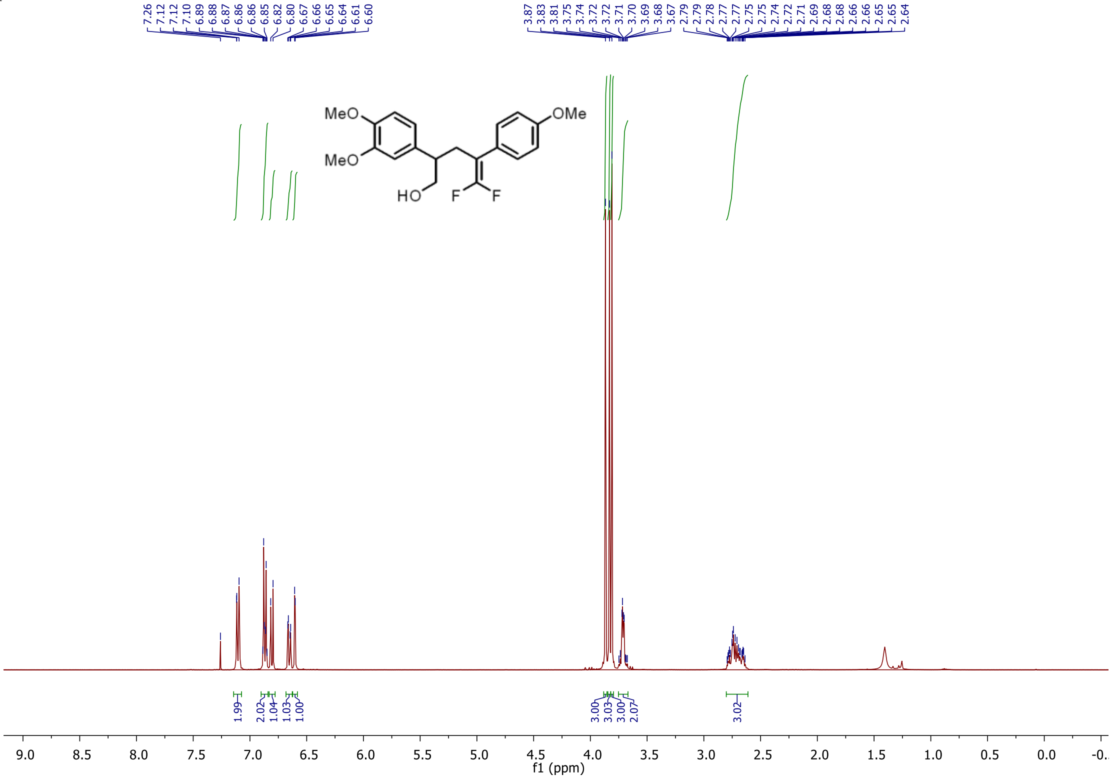
7.26 7.12 7.12 7.10 6.89 6.88 6.87 6.86 6.86 6.85 6.82 6.80 6.67 6.66 6.65 6.64 6.61 6.60
3.87 3.83 3.81 3.75 3.74 3.72 3.72 3.71 3.70 3.69 3.68 3.67 2.79 2.79 2.78 2.77 2.77 2.75 2.75 2.74 2.72 2.71 2.69 2.68 2.68 2.66 2.66 2.65 2.65 2.64
MeO
MeO
OMe
HO
F
F
1.99
2.02
1.04
1.03
1.00
3.00
3.03
3.00
2.07
3.02
f1 (ppm)

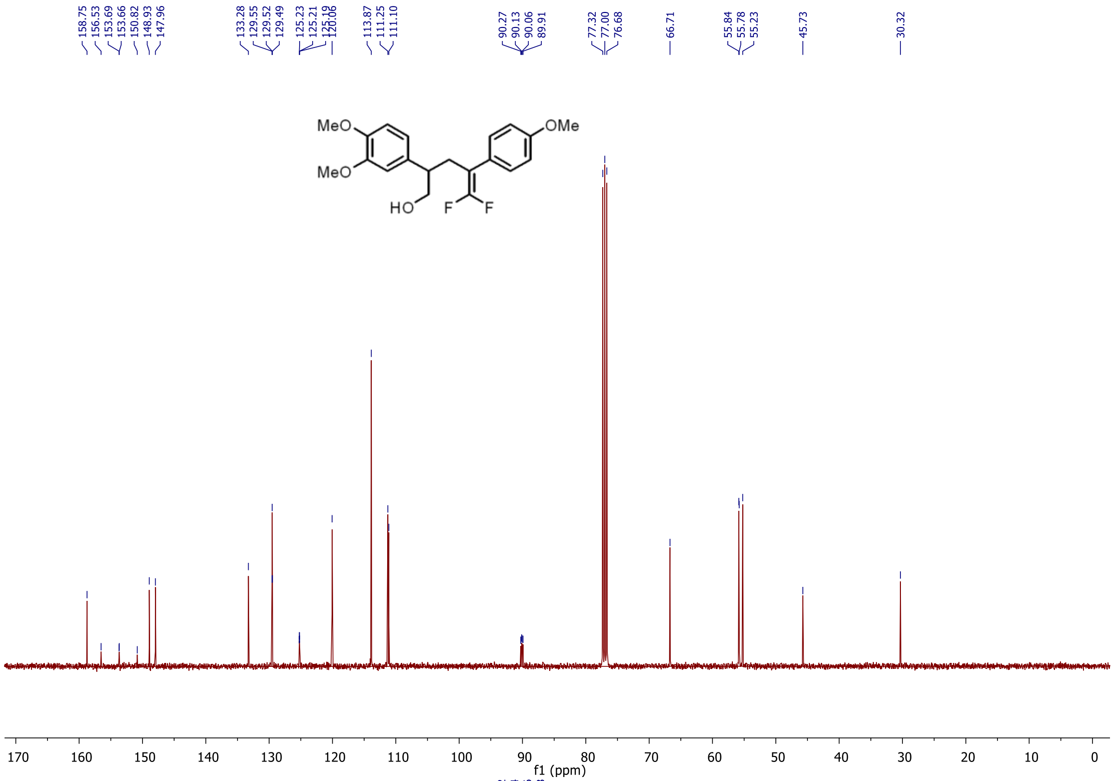
158.75
156.53
153.69
153.66
150.82
148.93
147.96
133.28
129.55
129.52
129.49
125.23
125.21
120.08
113.87
111.25
111.10
90.27
90.13
90.06
89.91
77.32
77.00
76.68
66.71
55.84
55.78
55.23
45.73
30.32
MeO
MeO
OMe
HO
F
F
170 160 150 140 130 120 110 100 90 80 70 60 50 40 30 20 10 0
f1 (ppm)

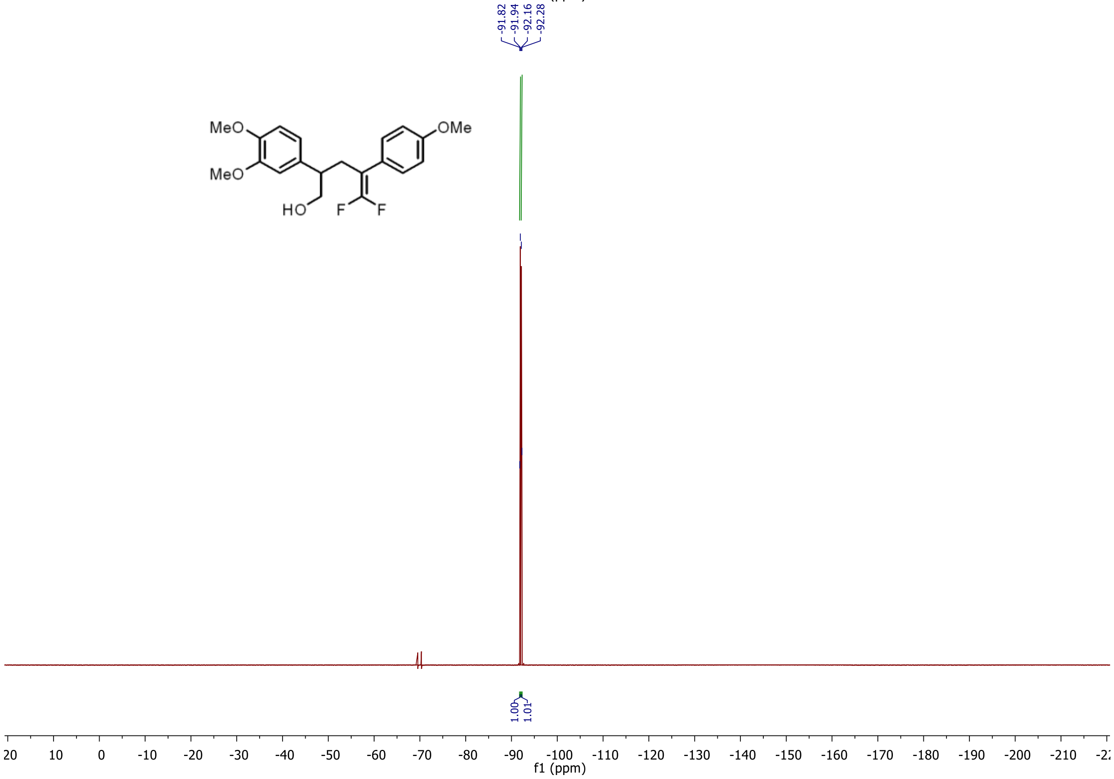
-91.82
-91.94
-92.16
-92.28
MeO
MeO
OMe
HO
F
F
1.00
1.01
20 10 0 -10 -20 -30 -40 -50 -60 -70 -80 -90 -100 -110 -120 -130 -140 -150 -160 -170 -180 -190 -200 -210
f1 (ppm)

Compound **52**

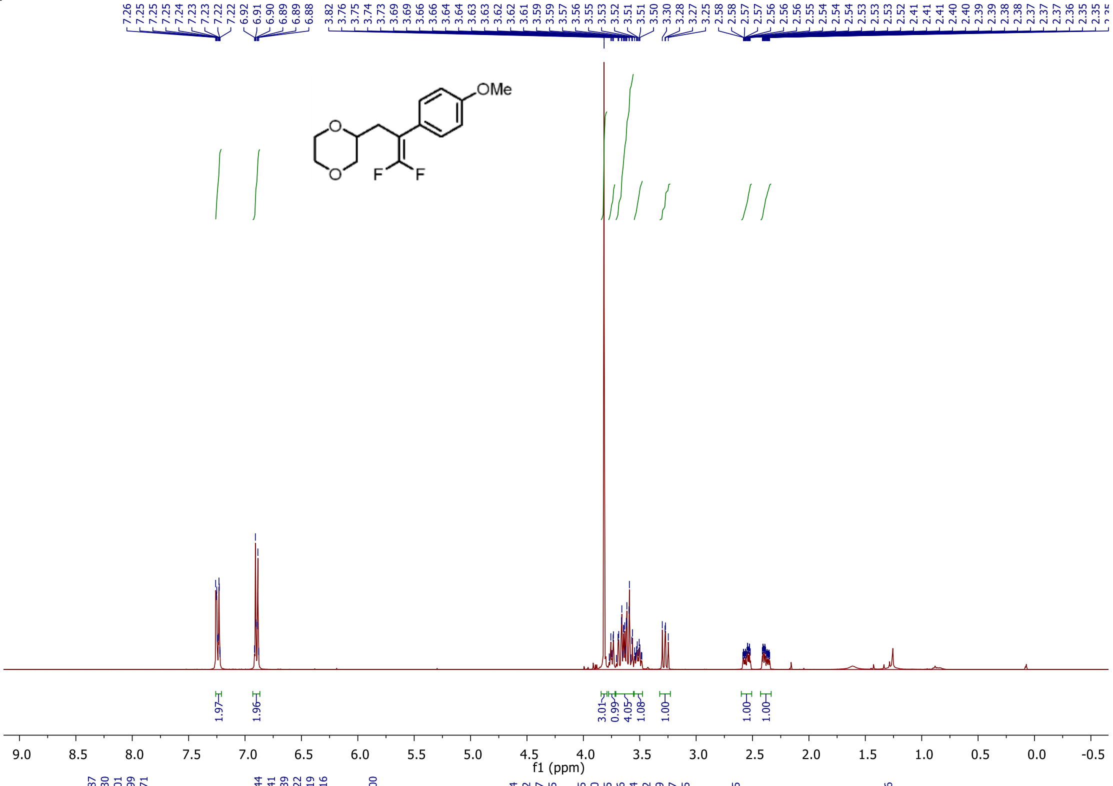

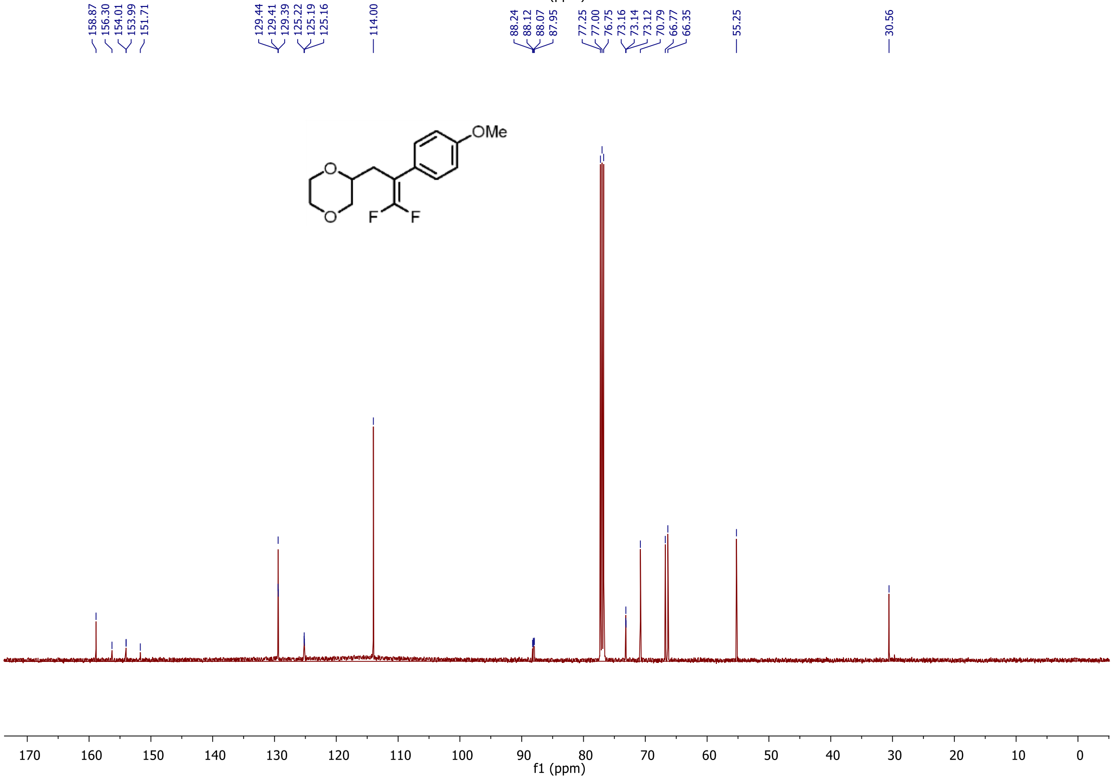

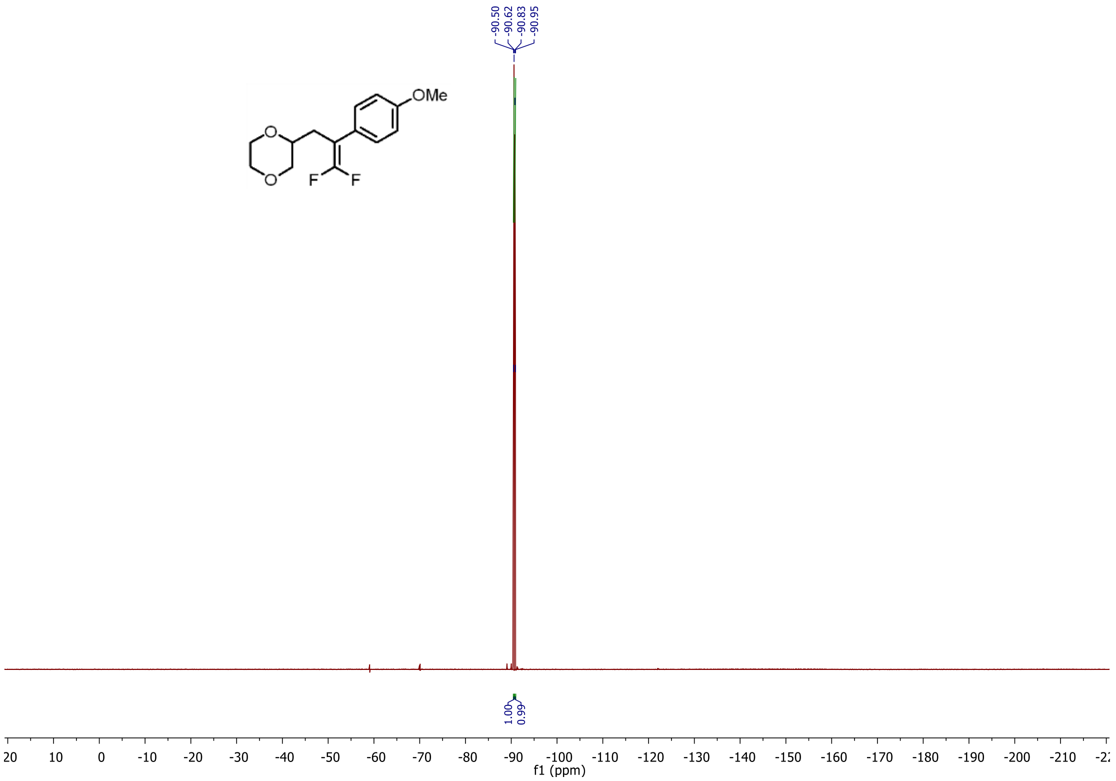
-90.50
-90.62
-90.83
-90.95
OMe
O
O
F
F
1.00
0.99
f1 (ppm)

Compound **53**

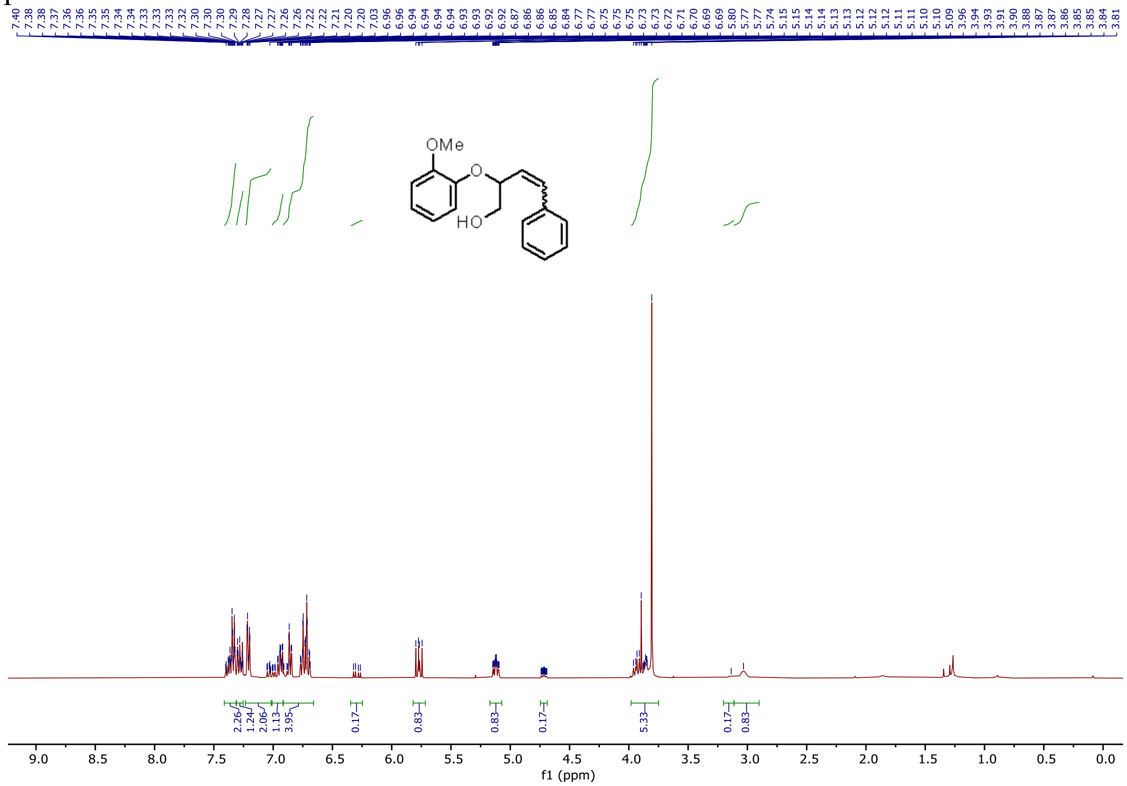
OMe
O
HO
2.26
1.24
2.06
1.13
3.95
0.17
0.83
0.83
0.17
5.33
0.17
0.83
f1 (ppm)

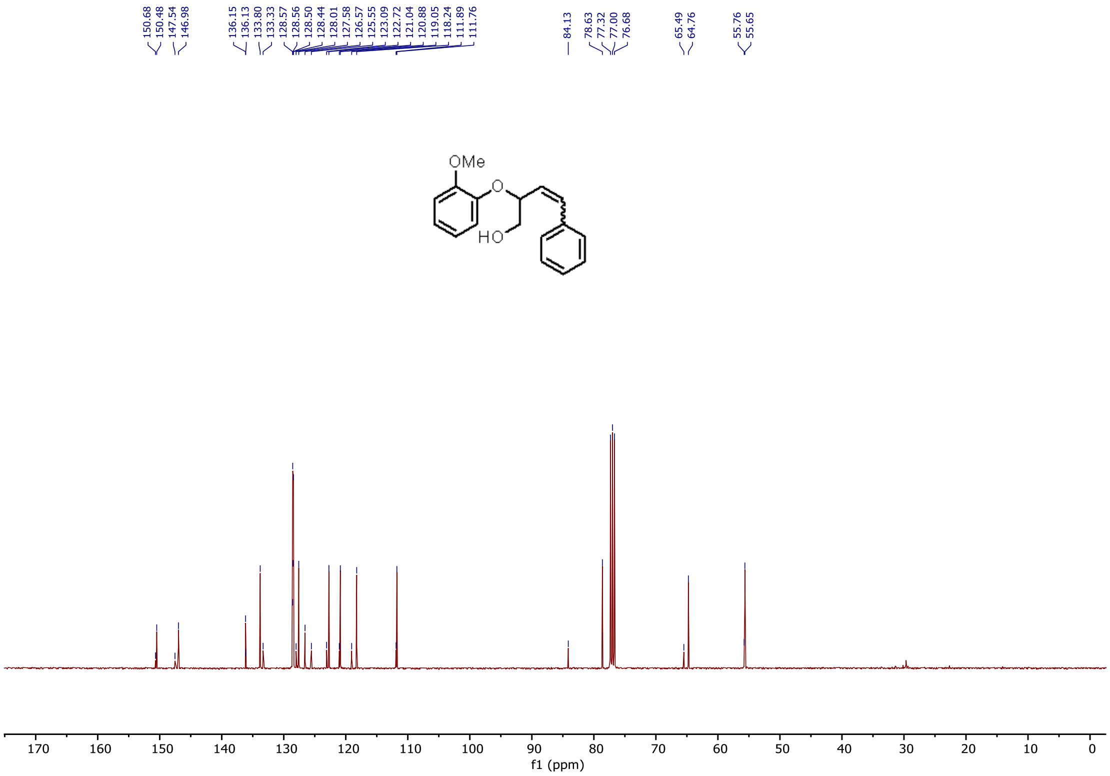

150.68
150.48
147.54
146.98
136.15
136.13
133.80
133.33
128.57
128.56
128.50
128.44
128.01
127.58
126.57
125.55
123.09
122.72
121.04
120.88
119.05
118.24
111.89
111.76
84.13
78.63
77.32
77.00
76.68
65.49
64.76
55.76
55.65
OMe
O
HO
f1 (ppm)

Compound **54**

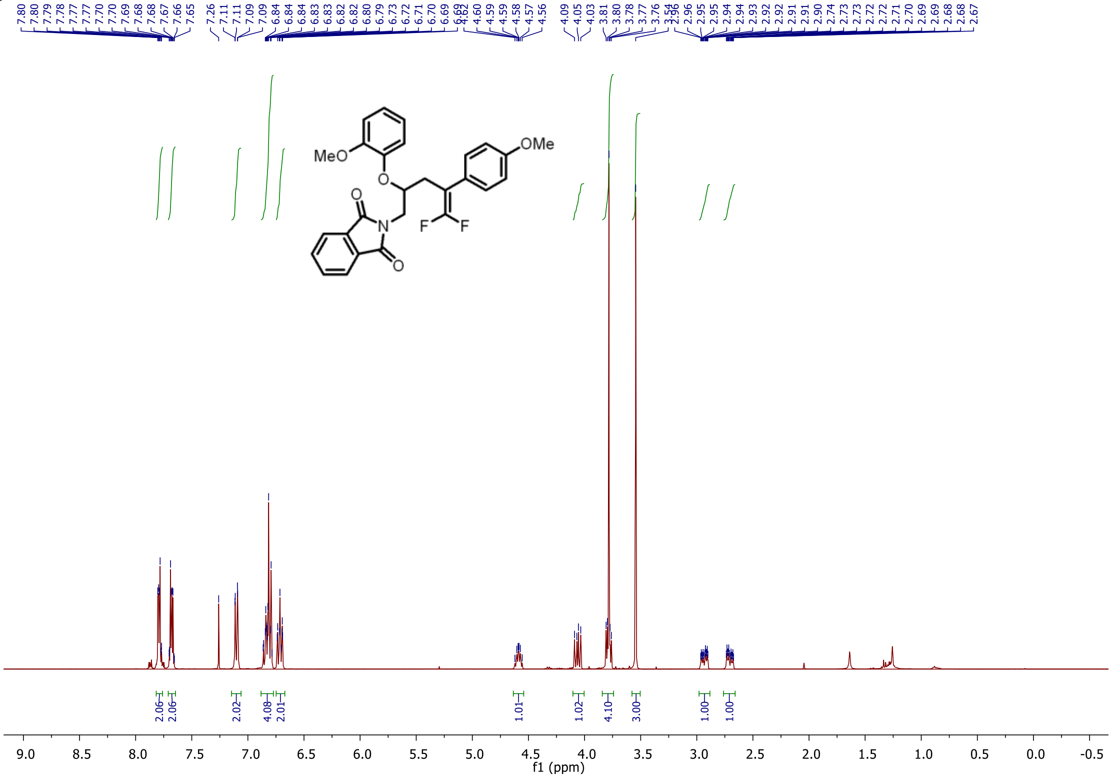

7.80
7.80
7.79
7.78
7.77
7.77
7.70
7.70
7.69
7.68
7.68
7.67
7.66
7.65
7.26
7.11
7.11
7.09
7.09
6.84
6.84
6.84
6.83
6.83
6.82
6.82
6.80
6.79
6.73
6.72
6.71
6.70
6.69
6.69
4.62
4.60
4.59
4.59
4.58
4.57
4.56
4.09
4.05
4.03
3.81
3.80
3.78
3.77
3.76
3.54
2.96
2.95
2.95
2.94
2.94
2.93
2.92
2.92
2.91
2.91
2.90
2.74
2.73
2.73
2.72
2.72
2.71
2.70
2.69
2.69
2.68
2.68
2.67
MeO
O
OMe
N
O
O
F
F
2.06
2.06
2.02
4.08
2.01
1.01
1.02
4.10
3.00
1.00
1.00
f1 (ppm)

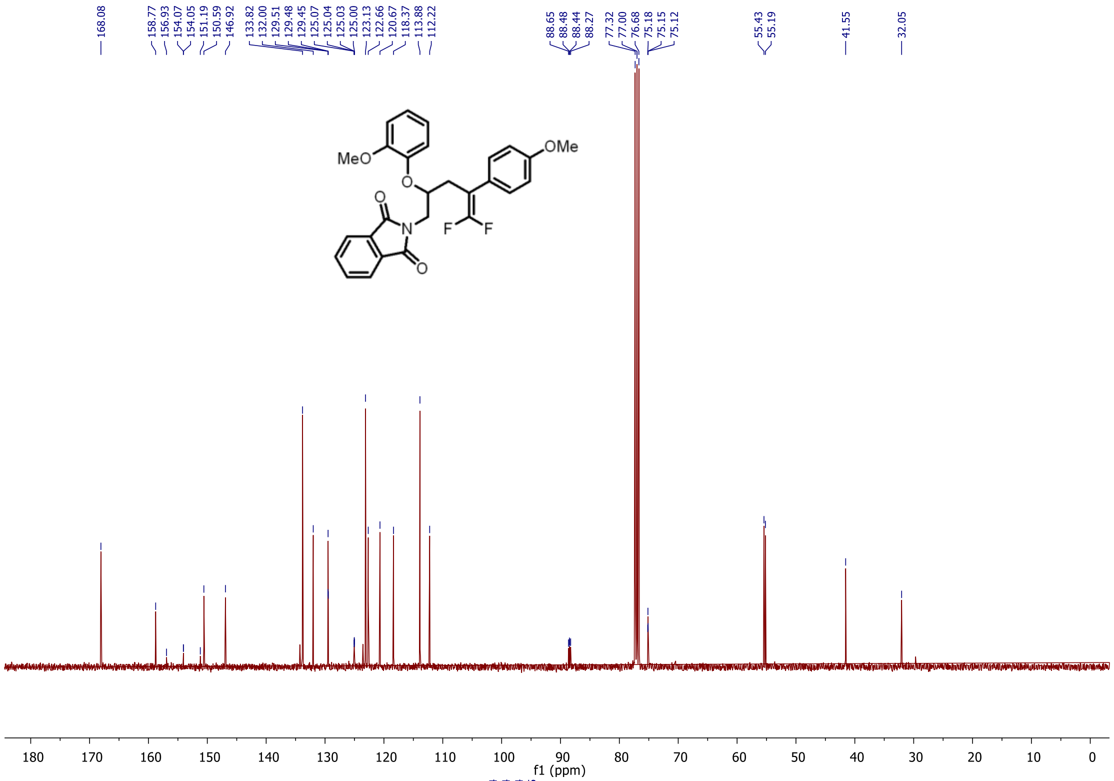

168.08
158.77
156.93
154.07
154.05
151.19
150.59
146.92
133.82
132.00
129.51
129.48
129.45
125.07
125.04
125.03
125.00
123.13
122.66
120.67
118.37
113.88
112.22
88.65
88.48
88.44
88.27
77.32
77.00
76.68
75.18
75.15
75.12
55.43
55.19
41.55
32.05
MeO
OMe
O
N
F
F
f1 (ppm)

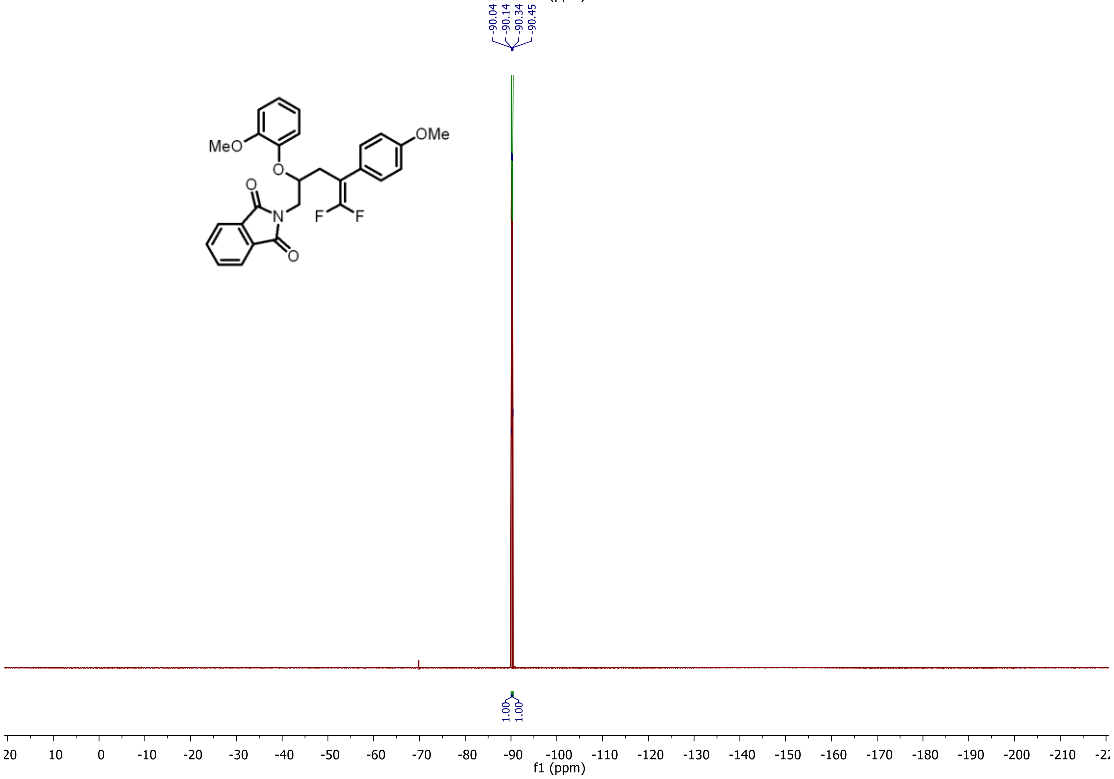

-90.04
-90.14
-90.34
-90.45
MeO
OMe
O
N
F
F
1.00
1.00
f1 (ppm)

Compound **55**

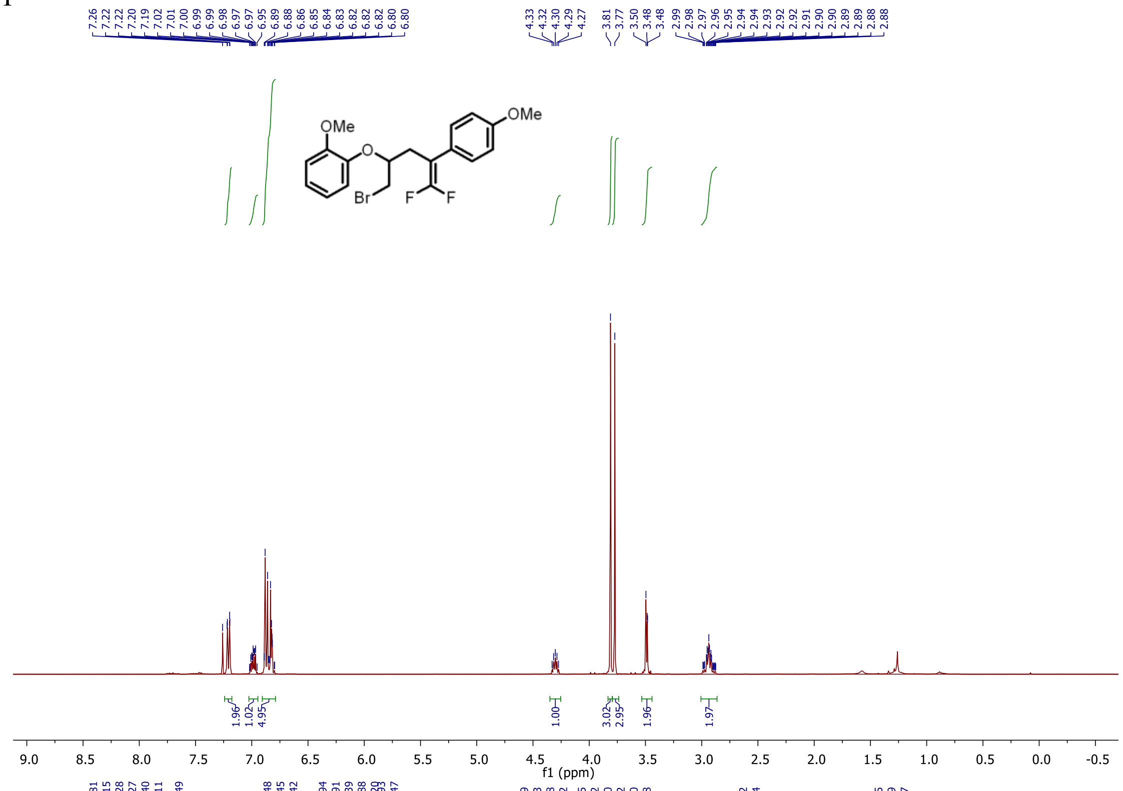

7.26
7.22
7.22
7.20
7.19
7.02
7.01
7.00
6.99
6.99
6.98
6.97
6.95
6.89
6.88
6.86
6.85
6.84
6.83
6.82
6.82
6.80
6.80
4.33
4.32
4.30
4.29
4.27
3.81
3.77
3.50
3.48
3.48
2.99
2.98
2.97
2.96
2.95
2.94
2.94
2.93
2.92
2.92
2.91
2.90
2.89
2.89
2.88
2.88
OMe
O
OMe
Br
F
F
1.96
1.02
4.95
1.00
3.02
2.95
1.96
1.97
9.0
8.5
8.0
7.5
7.0
6.5
6.0
5.5
5.0
4.5
4.0
3.5
3.0
2.5
2.0
1.5
1.0
0.5
0.0
-0.5
f1 (ppm)

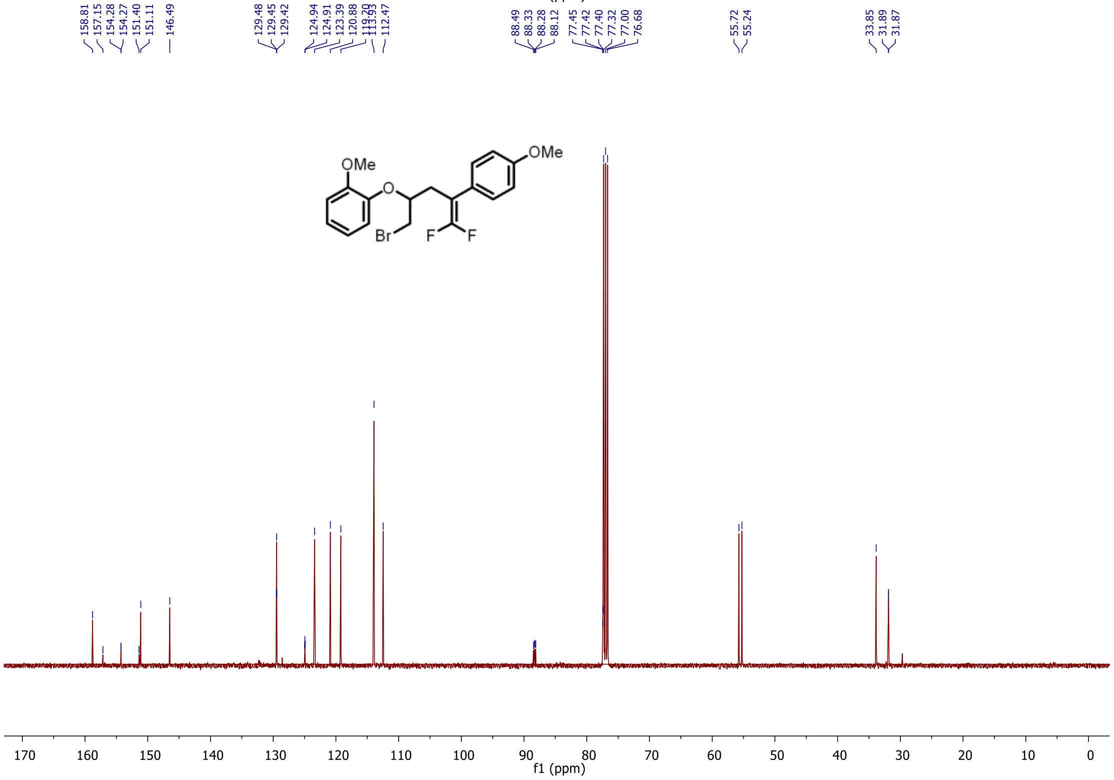

158.81
157.15
154.28
154.27
151.40
151.11
146.49
129.48
129.45
129.42
124.94
124.91
123.39
120.88
112.47
88.49
88.33
88.28
88.12
77.45
77.42
77.40
77.32
77.00
76.68
55.72
55.24
33.85
31.89
31.87
OMe
O
OMe
Br
F
F
170
160
150
140
130
120
110
100
90
80
70
60
50
40
30
20
10
0
f1 (ppm)

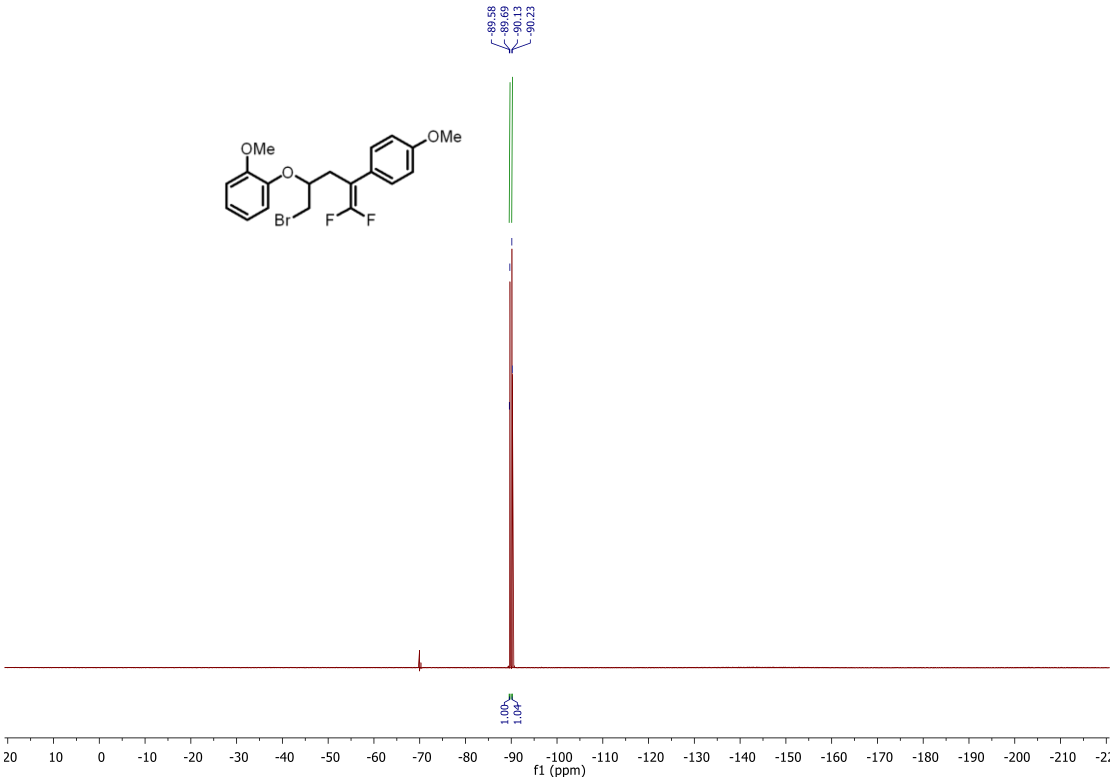
-89.58
-89.69
-90.13
-90.23
OMe
O
OMe
Br
F
F
1.00
1.04
f1 (ppm)

Compound **56**

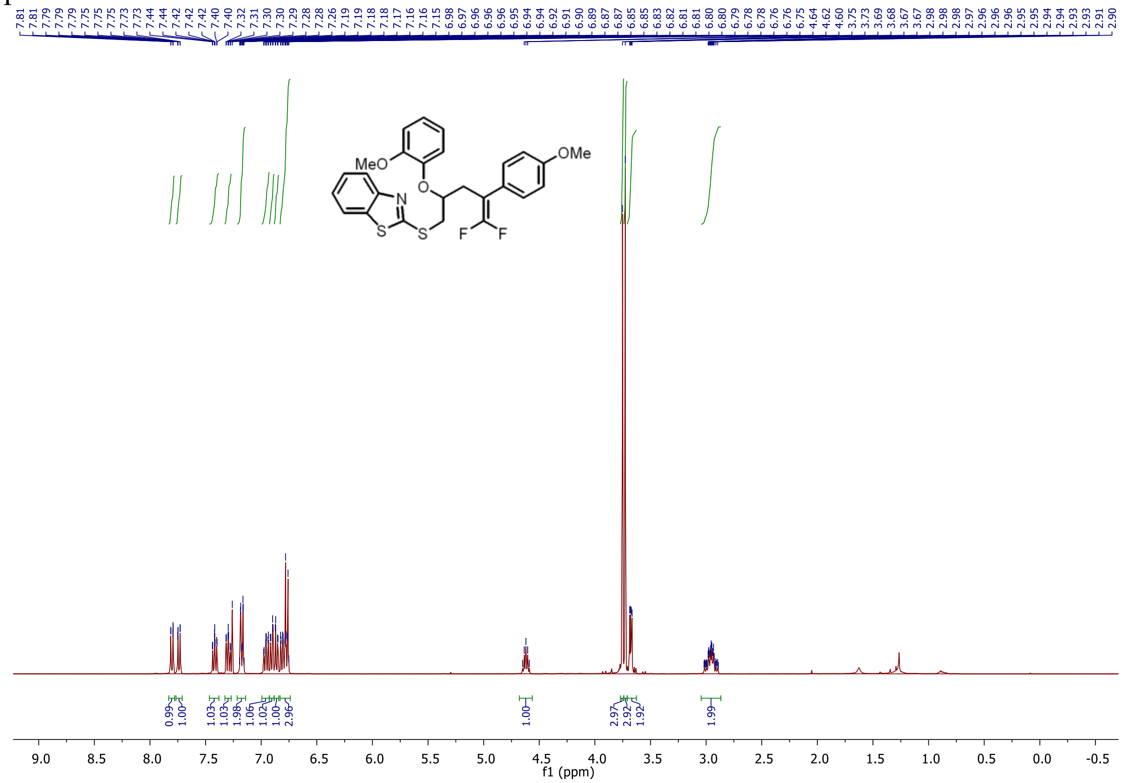
MeO
O
OMe
N
S
S
F
F
f1 (ppm)

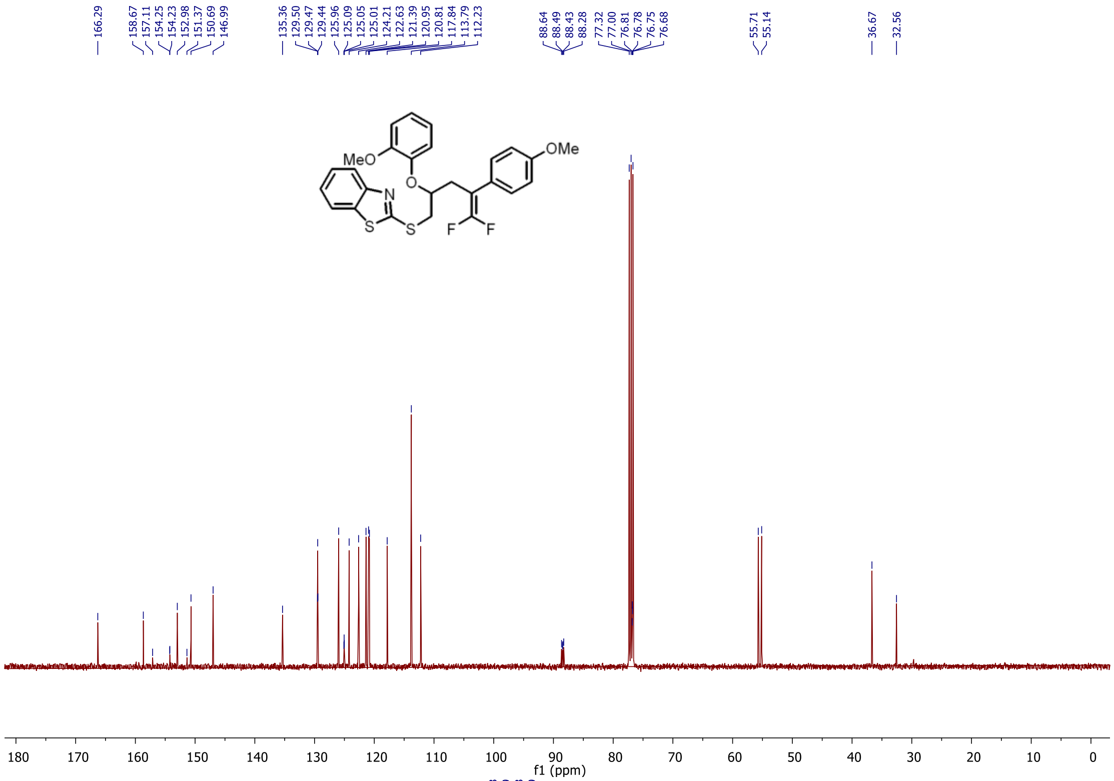
166.29
158.67
157.11
154.25
154.23
152.98
151.37
150.69
146.99
135.36
129.50
129.47
129.44
125.96
125.09
125.05
125.01
124.21
122.63
121.39
120.95
120.81
117.84
113.79
112.23
88.64
88.49
88.43
88.28
77.32
77.00
76.81
76.78
76.75
76.68
55.71
55.14
36.67
32.56
MeO
O
OMe
N
S
S
F
F
f1 (ppm)

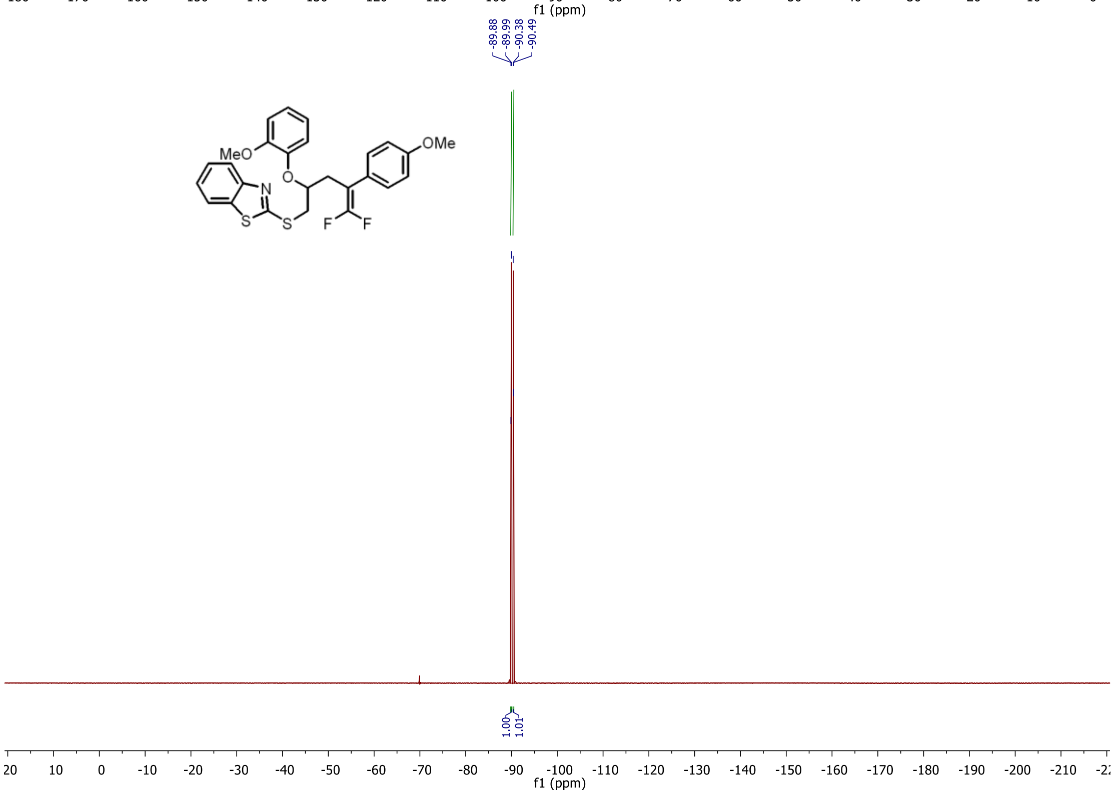
-89.88
-89.99
-90.38
-90.49
MeO
O
OMe
N
S
S
F
F
1.00
1.01
f1 (ppm)

Compound **57**

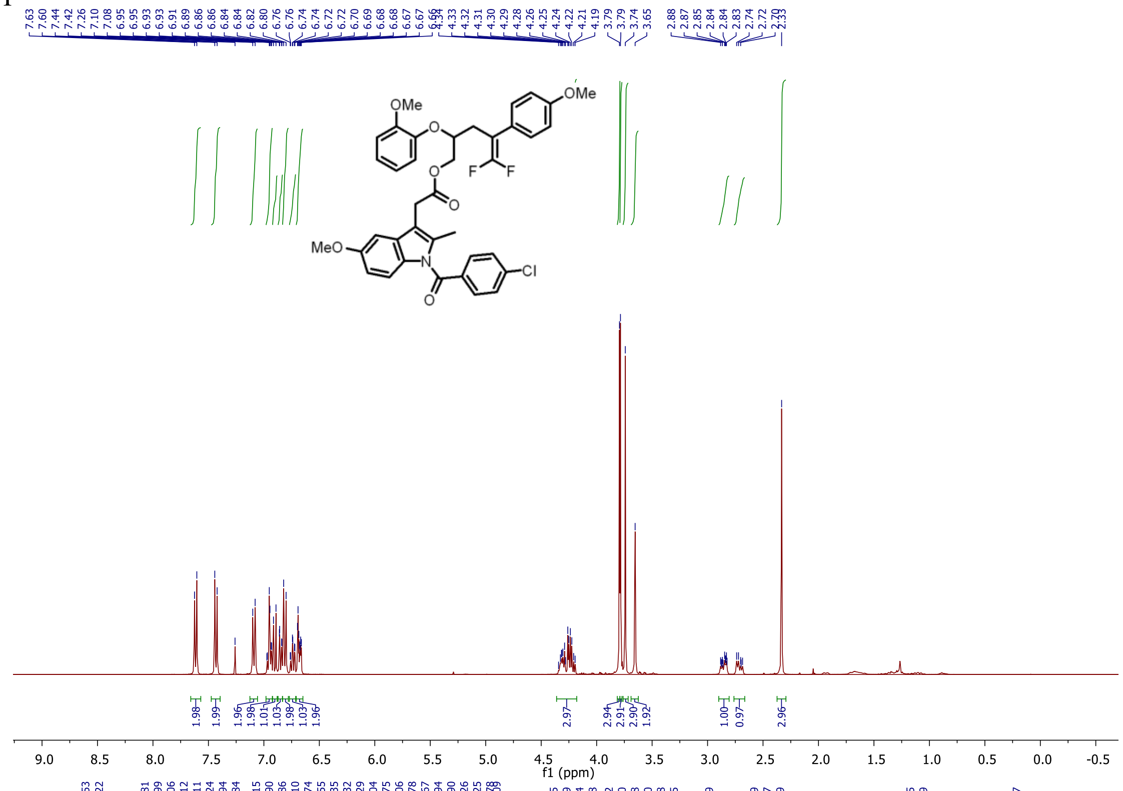

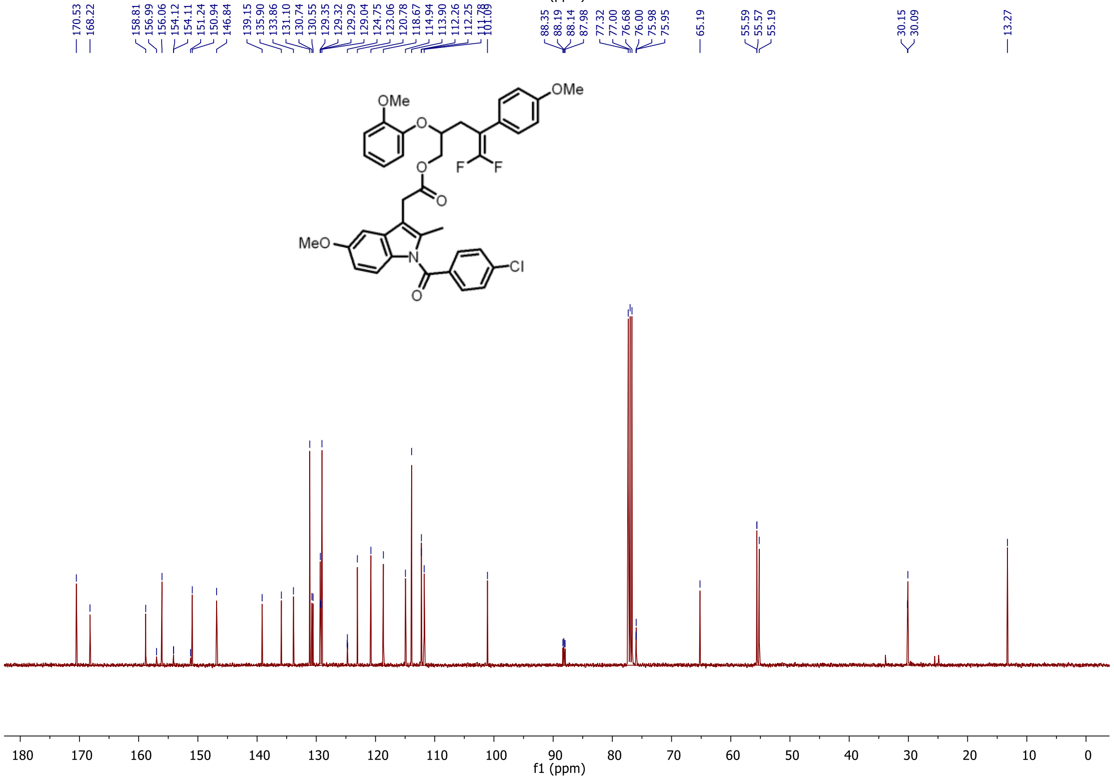

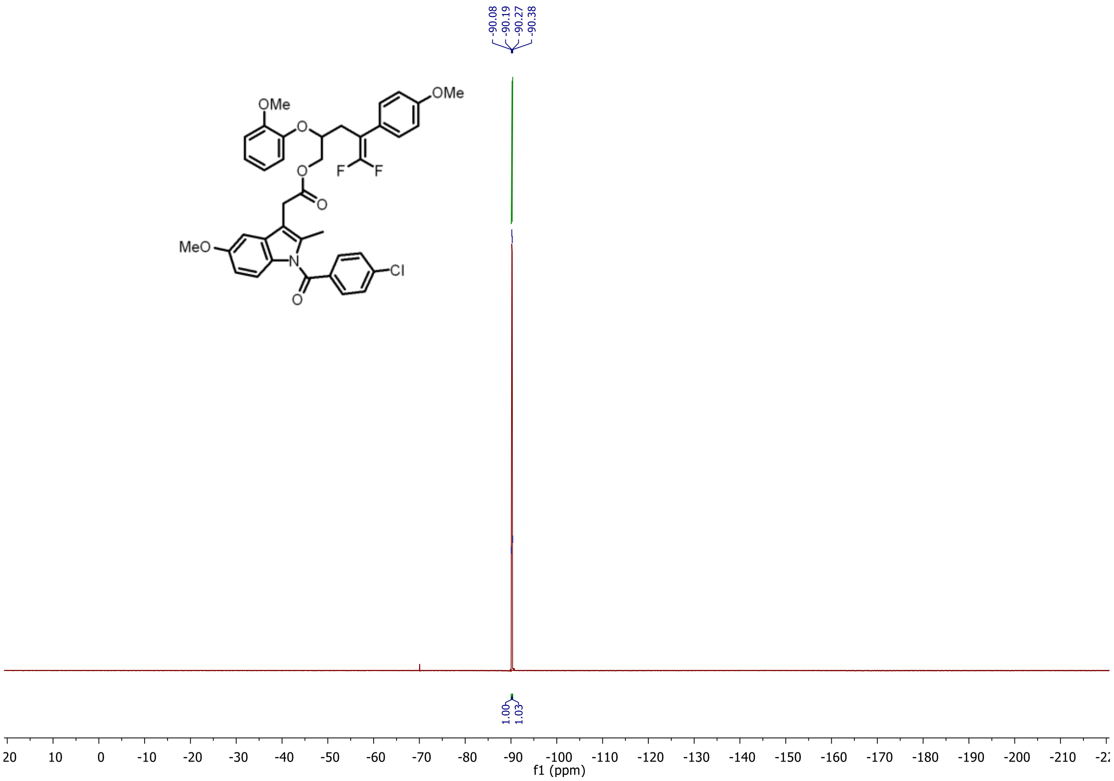
-90.08
-90.19
-90.27
-90.38
OMe
OMe
F
F
O
O
O
O
MeO
N
Cl
O
1.00
1.03
f1 (ppm)

Compound **58**

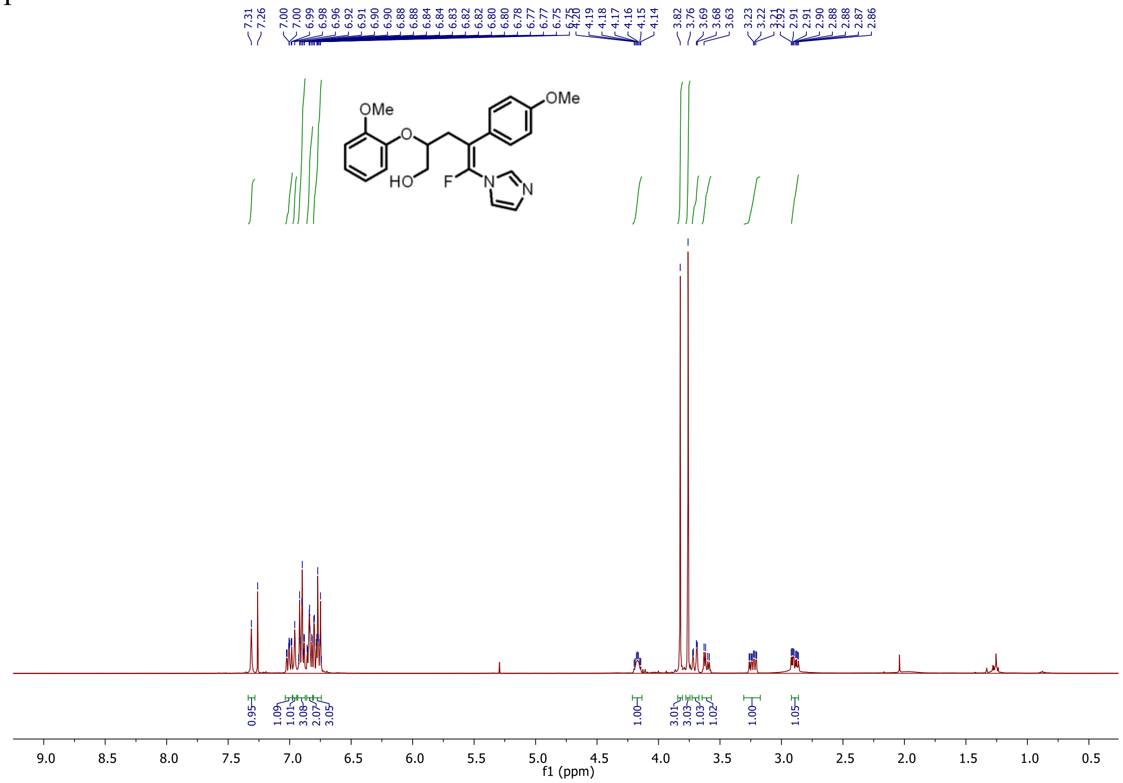
7.31
7.26
7.00
7.00
6.99
6.98
6.96
6.92
6.91
6.90
6.90
6.88
6.88
6.84
6.83
6.82
6.82
6.80
6.80
6.78
6.77
6.77
6.75
6.75
4.20
4.19
4.18
4.17
4.16
4.15
4.14
3.82
3.76
3.69
3.68
3.63
3.23
3.22
3.21
2.92
2.91
2.91
2.90
2.88
2.88
2.87
2.86
OMe
OMe
O
HO
F
N
N
0.95
1.09
1.01
3.08
2.07
3.05
1.00
3.01
3.03
1.03
1.02
1.00
1.05
f1 (ppm)

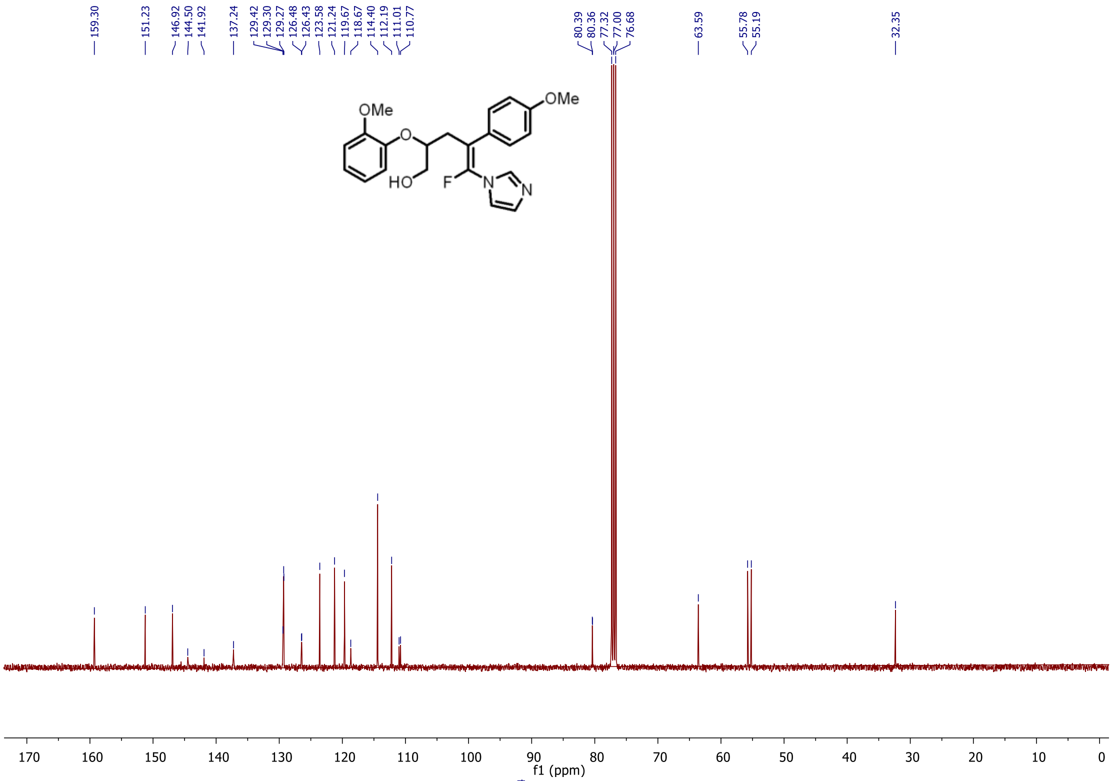
159.30
151.23
146.92
144.50
141.92
137.24
129.42
129.30
129.27
126.48
126.43
123.58
121.24
119.67
118.67
114.40
112.19
111.01
110.77
80.39
80.36
77.32
77.00
76.68
63.59
55.78
55.19
32.35
OMe
O
OMe
HO
F
N
N
170 160 150 140 130 120 110 100 90 80 70 60 50 40 30 20 10 0
f1 (ppm)

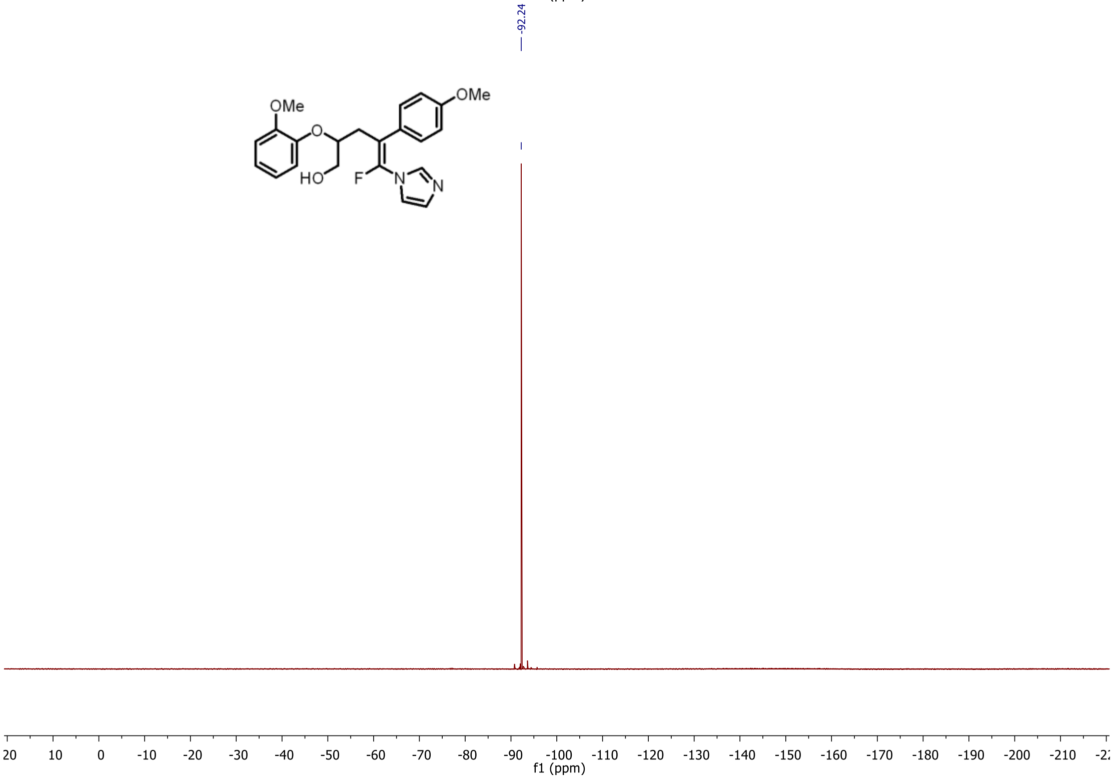
-92.24
OMe
O
OMe
HO
F
N
N
20 10 0 -10 -20 -30 -40 -50 -60 -70 -80 -90 -100 -110 -120 -130 -140 -150 -160 -170 -180 -190 -200 -210
f1 (ppm)

Compound **59**

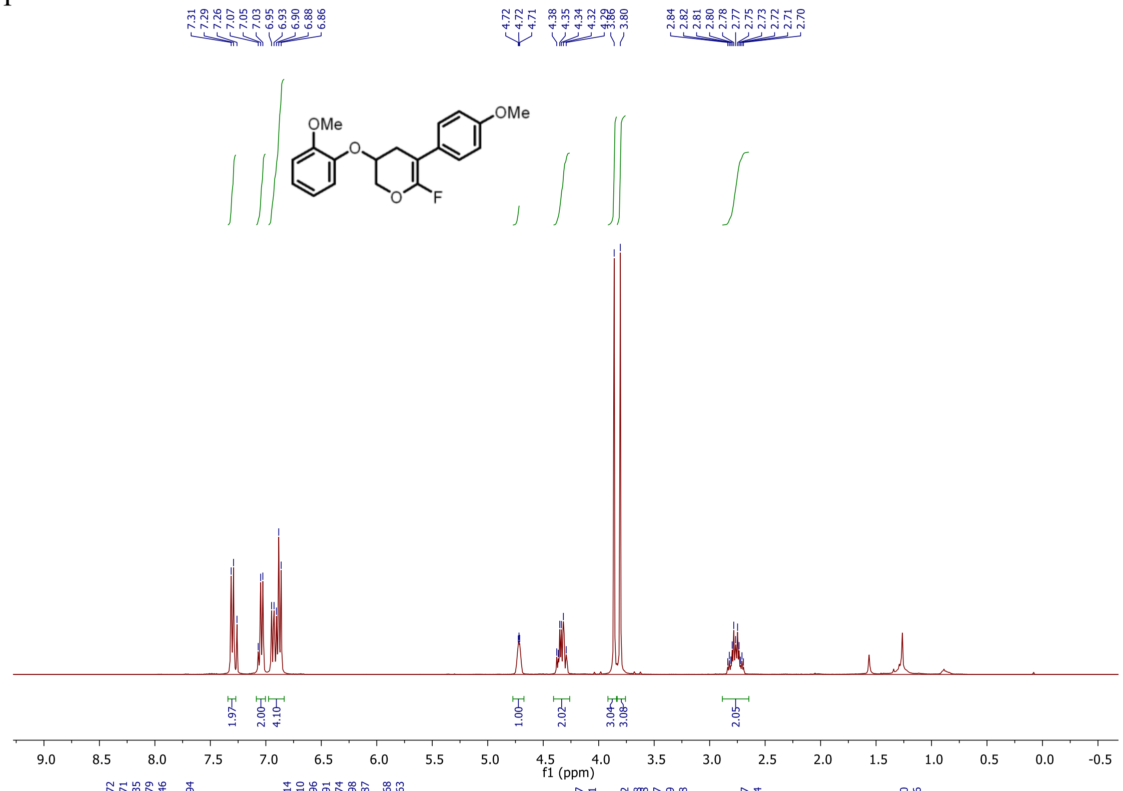

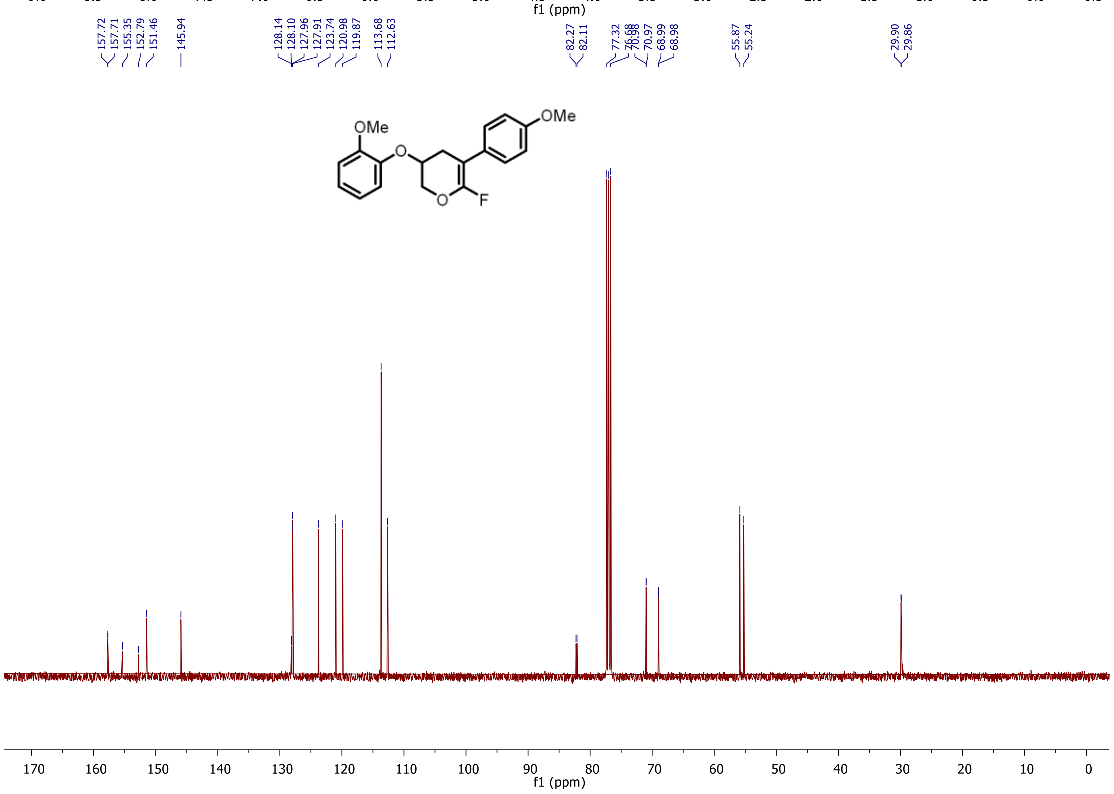

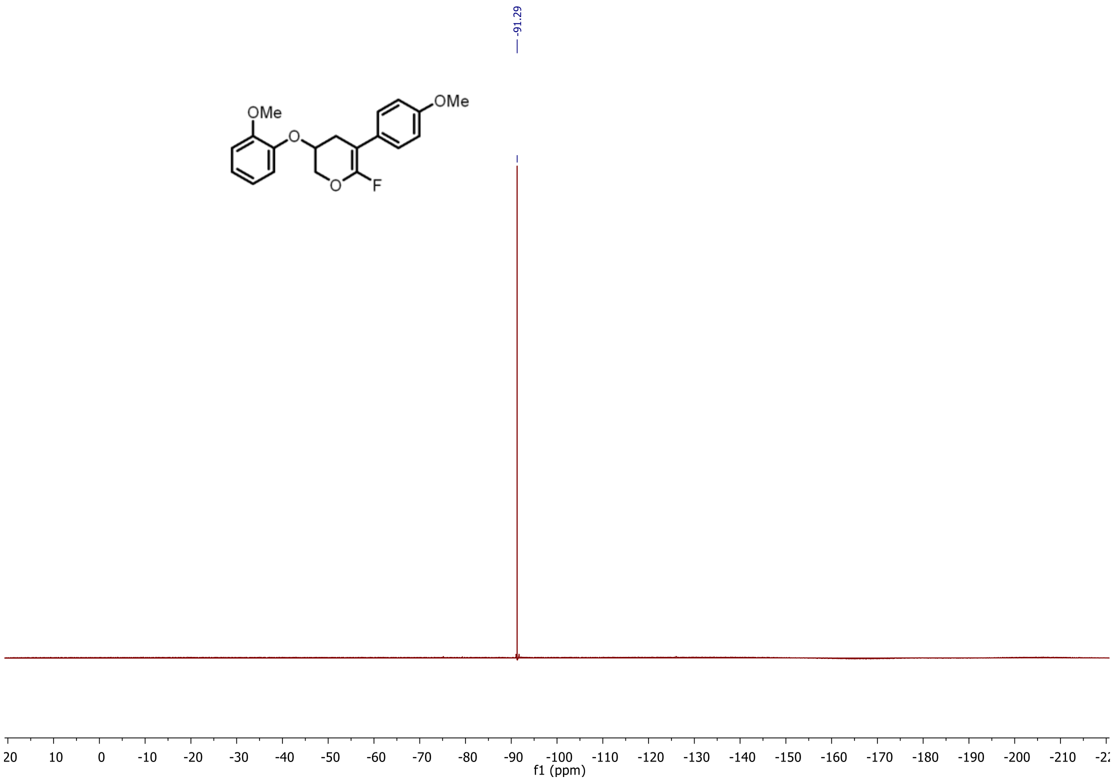

-91.29
OMe
OMe
O
O
F
20
10
0
-10
-20
-30
-40
-50
-60
-70
-80
-90
-100
-110
-120
-130
-140
-150
-160
-170
-180
-190
-200
-210
f1 (ppm)

Compound **60**

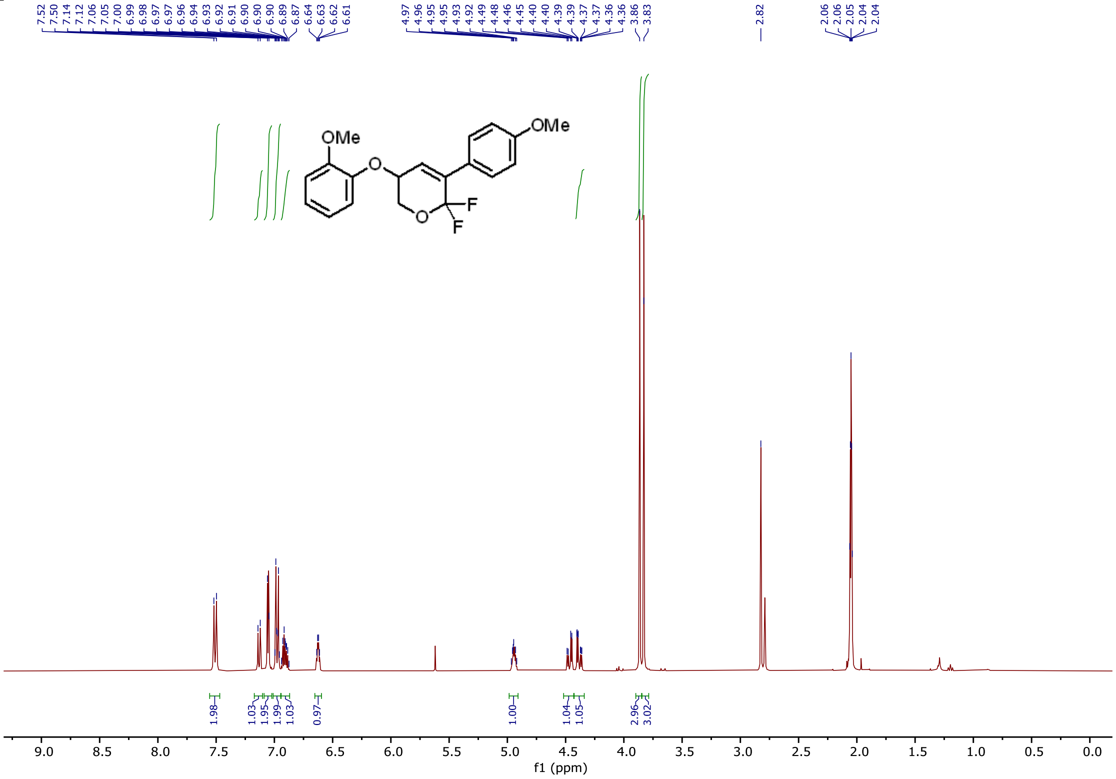

7.52
7.50
7.14
7.12
7.06
7.05
7.00
6.99
6.98
6.97
6.97
6.96
6.94
6.93
6.92
6.91
6.90
6.90
6.90
6.89
6.87
6.64
6.63
6.62
6.61
4.97
4.96
4.95
4.95
4.93
4.92
4.49
4.48
4.46
4.45
4.40
4.40
4.39
4.37
4.37
4.36
3.86
3.83
2.82
2.06
2.06
2.05
2.04
2.04
OMe
OMe
O
O
F
F
1.98
1.03
1.95
1.99
1.03
0.97
1.00
1.04
1.05
2.96
3.02
9.0
8.5
8.0
7.5
7.0
6.5
6.0
5.5
5.0
4.5
4.0
3.5
3.0
2.5
2.0
1.5
1.0
0.5
0.0
f1 (ppm)

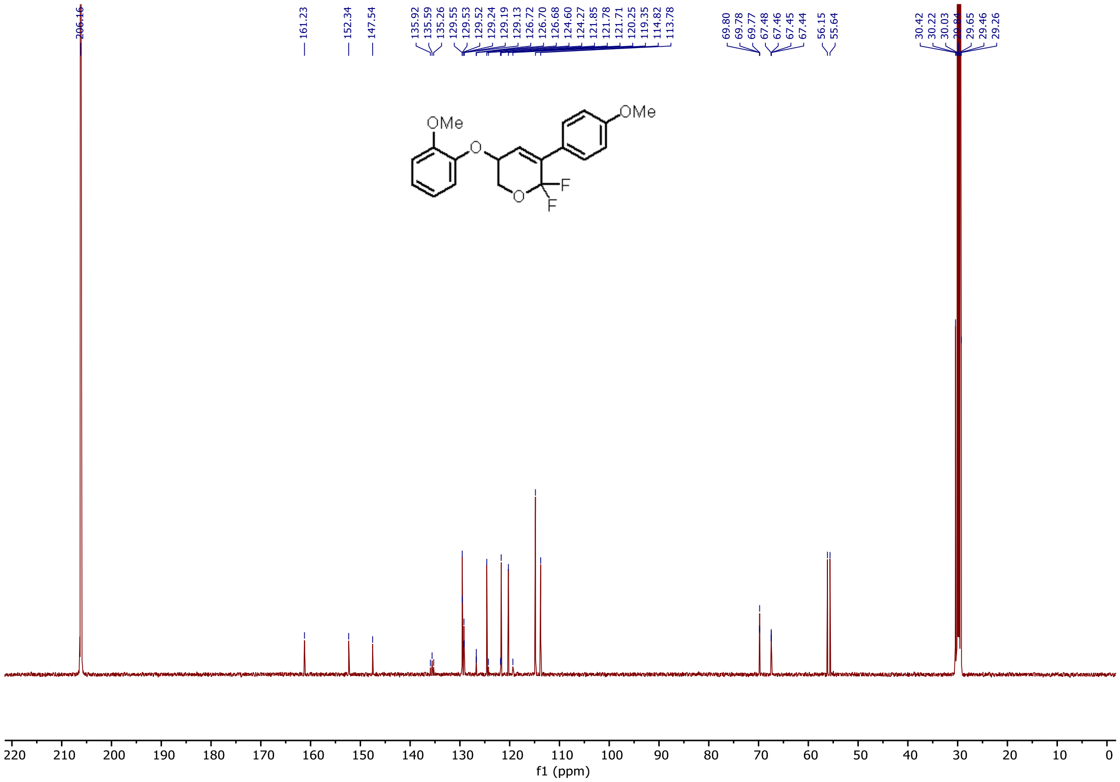
206.16
161.23
152.34
147.54
135.92
135.59
135.26
129.55
129.53
129.52
129.24
129.19
129.13
126.72
126.70
126.68
124.60
124.27
121.85
121.78
121.71
120.25
119.35
114.82
113.78
69.80
69.78
69.77
67.48
67.46
67.45
67.44
56.15
55.64
30.42
30.22
30.03
29.65
29.46
29.26
OMe
O
OMe
F
F
O
220
210
200
190
180
170
160
150
140
130
120
110
100
90
80
70
60
50
40
30
20
10
0
f1 (ppm)

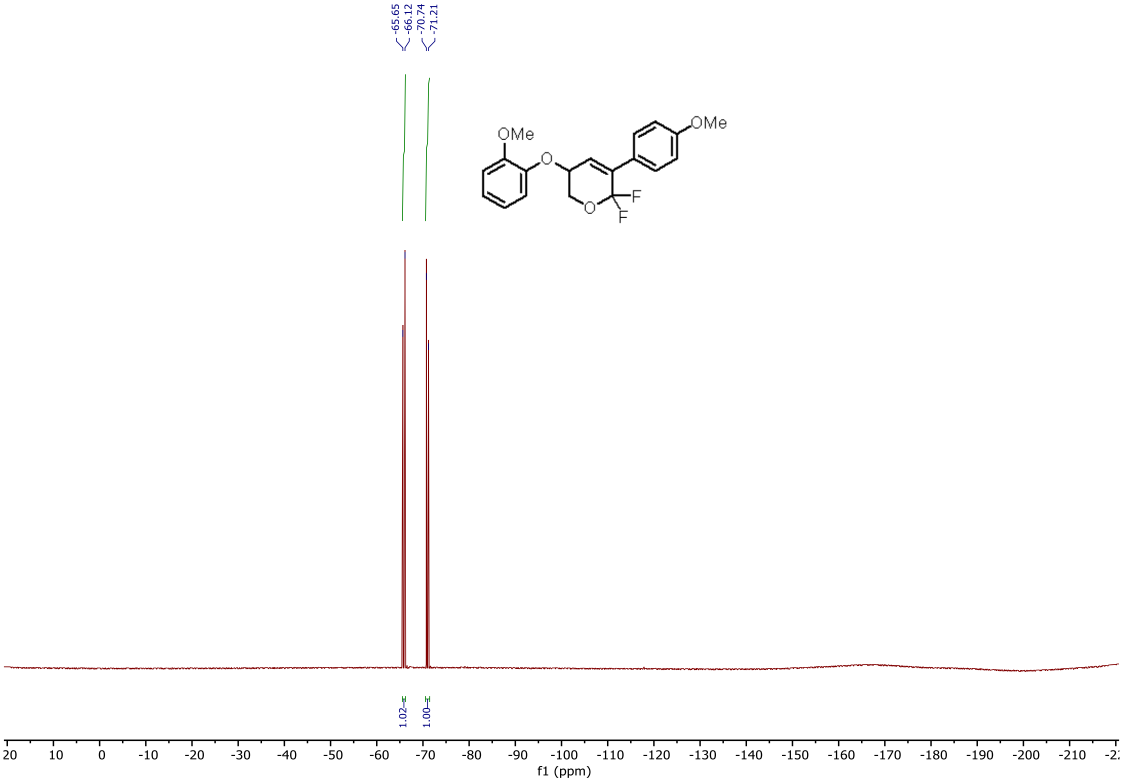
-65.65
-66.12
-70.74
-71.21
OMe
O
OMe
F
F
O
1.02
1.00
20
10
0
-10
-20
-30
-40
-50
-60
-70
-80
-90
-100
-110
-120
-130
-140
-150
-160
-170
-180
-190
-200
-210
f1 (ppm)

Compound **61**

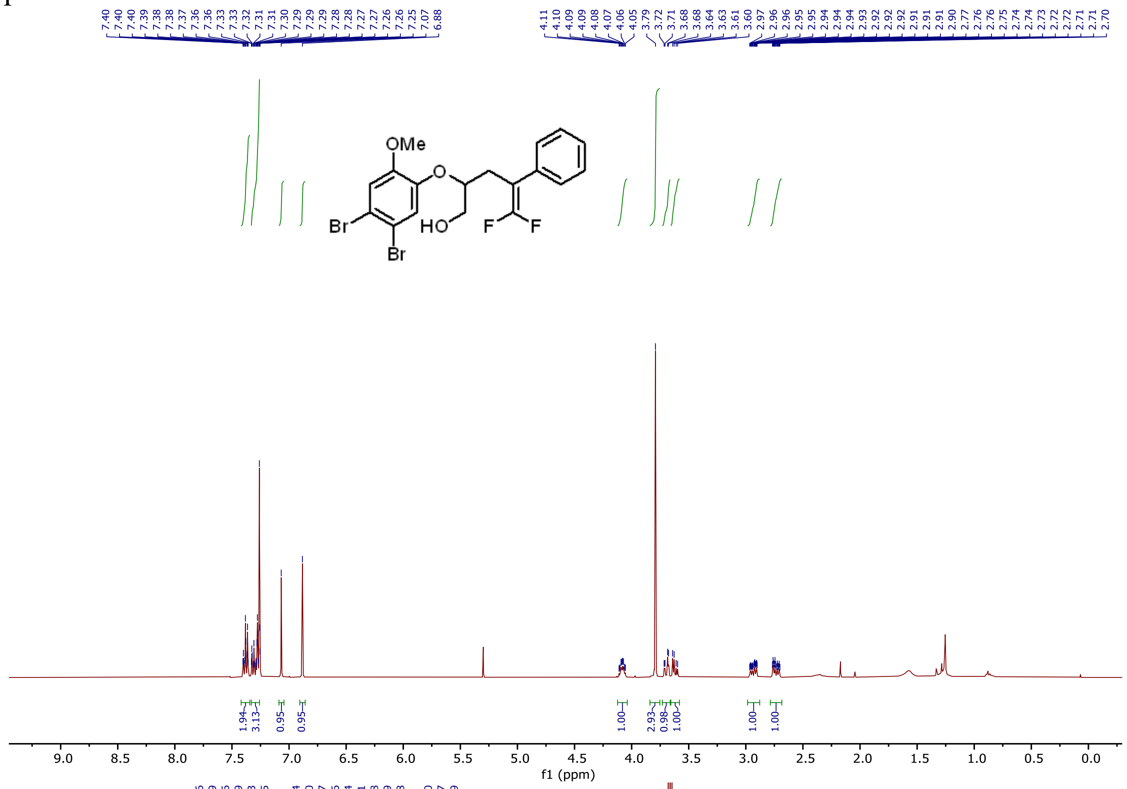

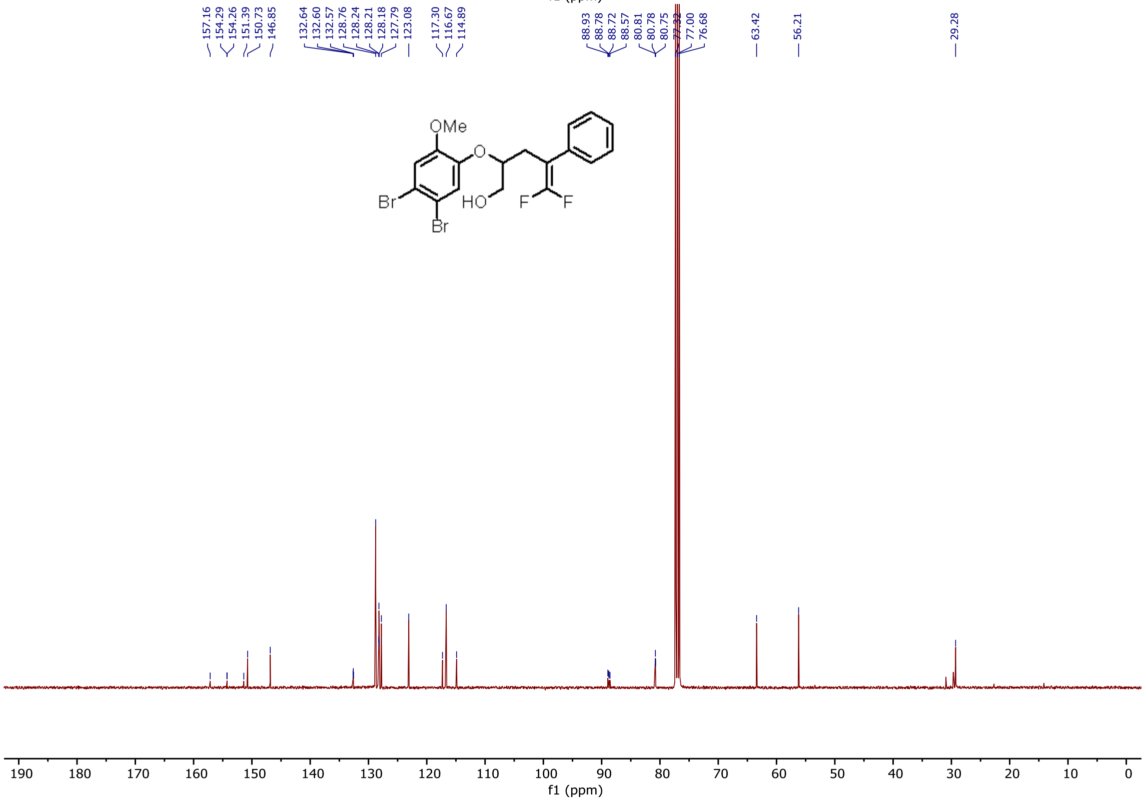

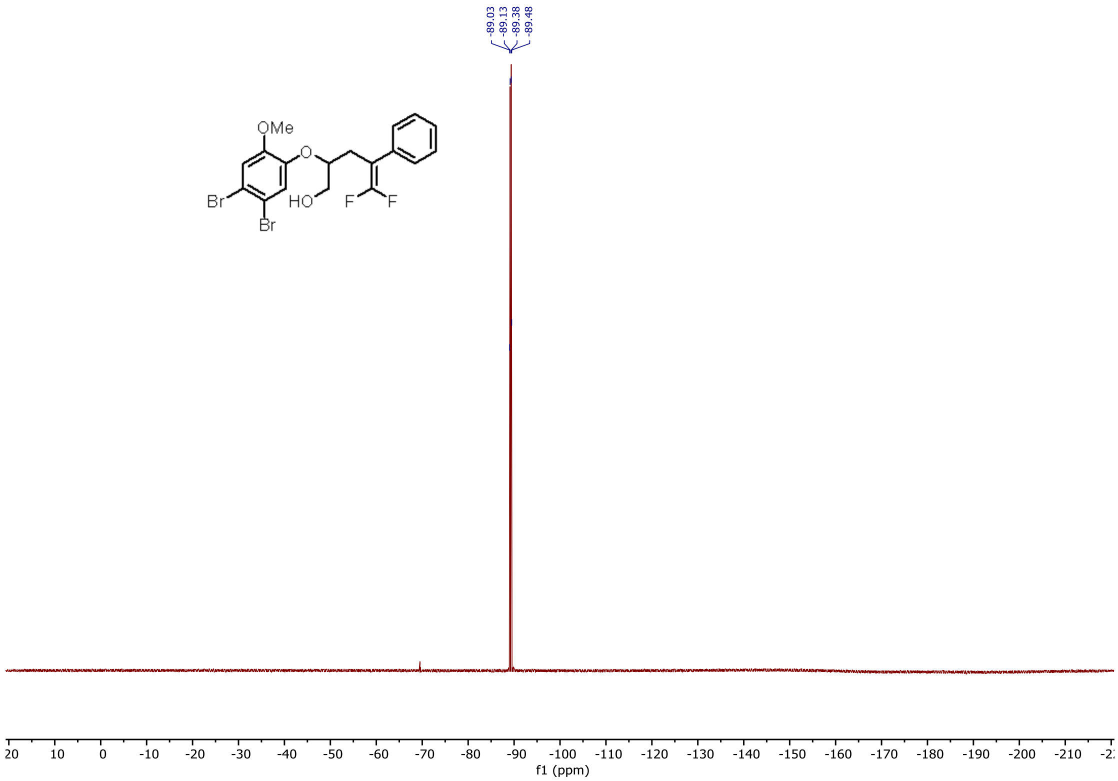
-89.03
-89.13
-89.38
-89.48
OMe
O
Br
Br
HO
F
F
f1 (ppm)

Compound **62**

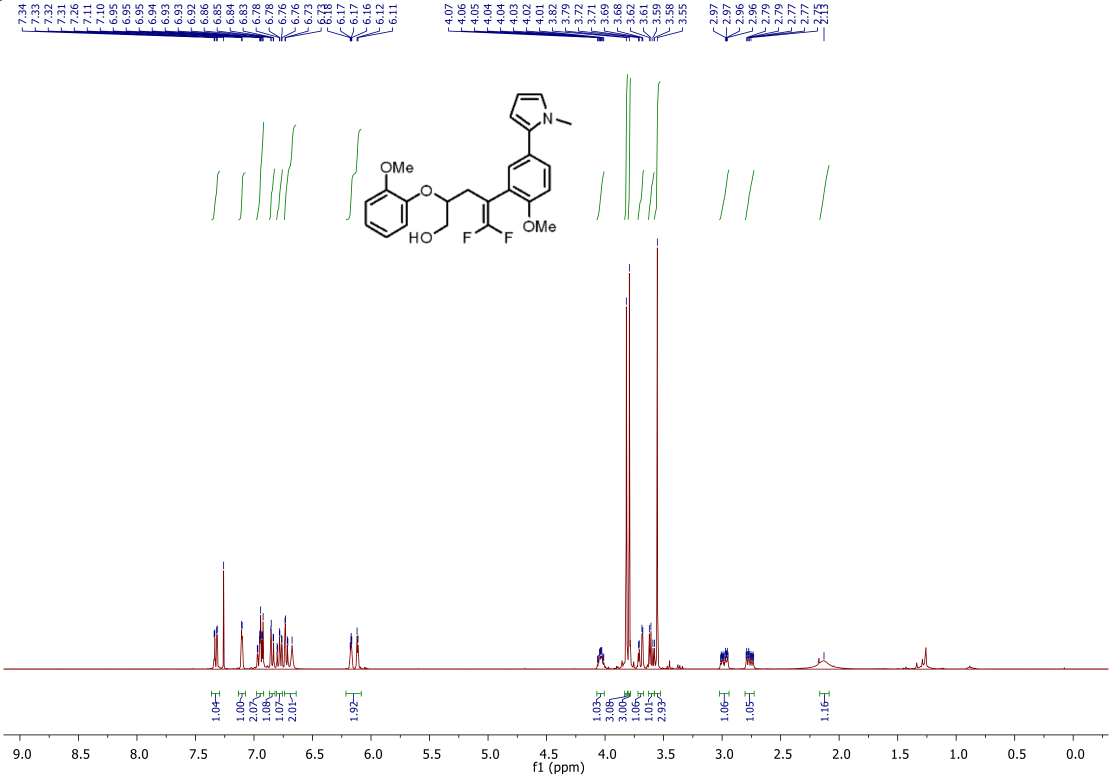
7.34 7.33 7.32 7.31 7.26 7.11 7.10 6.95 6.95 6.95 6.94 6.93 6.93 6.92 6.86 6.85 6.84 6.83 6.78 6.78 6.76 6.76 6.73 6.18 6.17 6.17 6.16 6.12 6.11
4.07 4.06 4.05 4.04 4.04 4.03 4.02 4.01 3.82 3.79 3.72 3.71 3.69 3.68 3.62 3.61 3.59 3.58 3.55
2.97 2.97 2.96 2.96 2.79 2.79 2.77 2.77 2.75 2.13
OMe
O
HO
F
F
OMe
N
1.04 1.00 2.07 1.08 1.07 2.01 1.92 1.03 3.08 3.00 1.06 1.01 2.93 1.06 1.05 1.16
f1 (ppm)

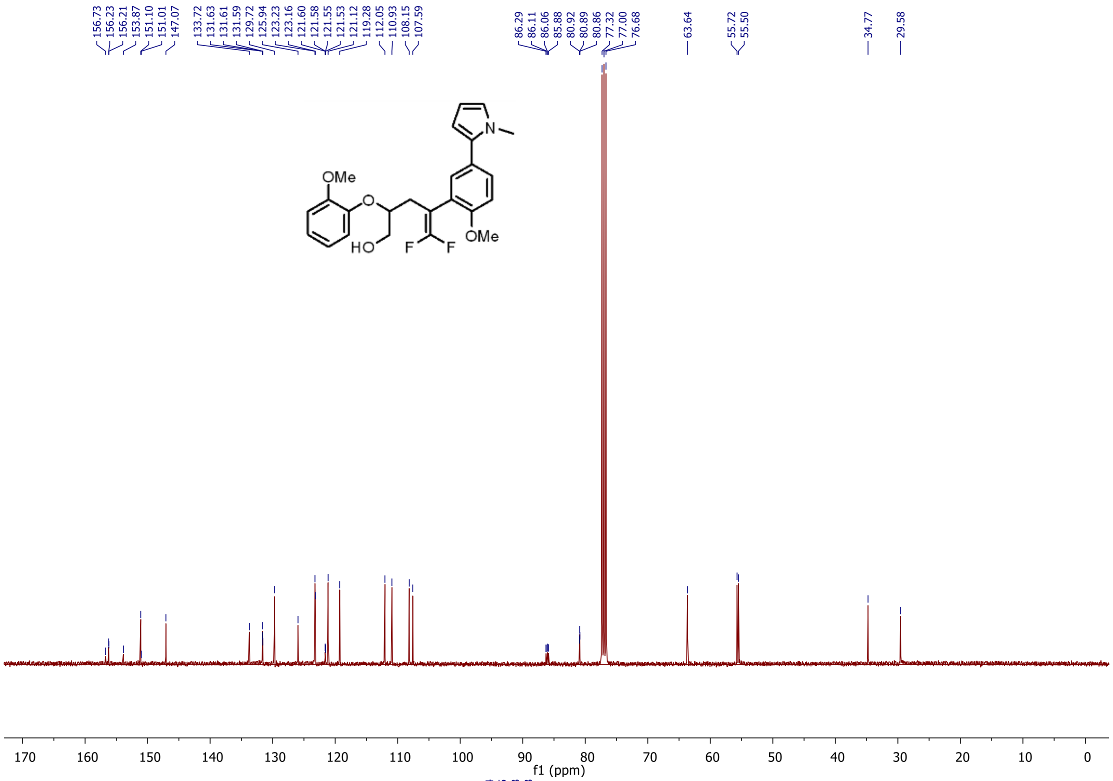

156.73
156.23
156.21
153.87
151.10
151.01
147.07
133.72
131.63
131.61
131.59
129.72
125.94
123.23
123.16
121.60
121.58
121.55
121.53
121.12
119.28
112.05
110.93
108.15
107.59
86.29
86.11
86.06
85.88
80.92
80.89
80.86
77.32
77.00
76.68
63.64
55.72
55.50
34.77
29.58
OMe
O
N
HO
F
F
OMe
170
160
150
140
130
120
110
100
90
80
70
60
50
40
30
20
10
0
f1 (ppm)

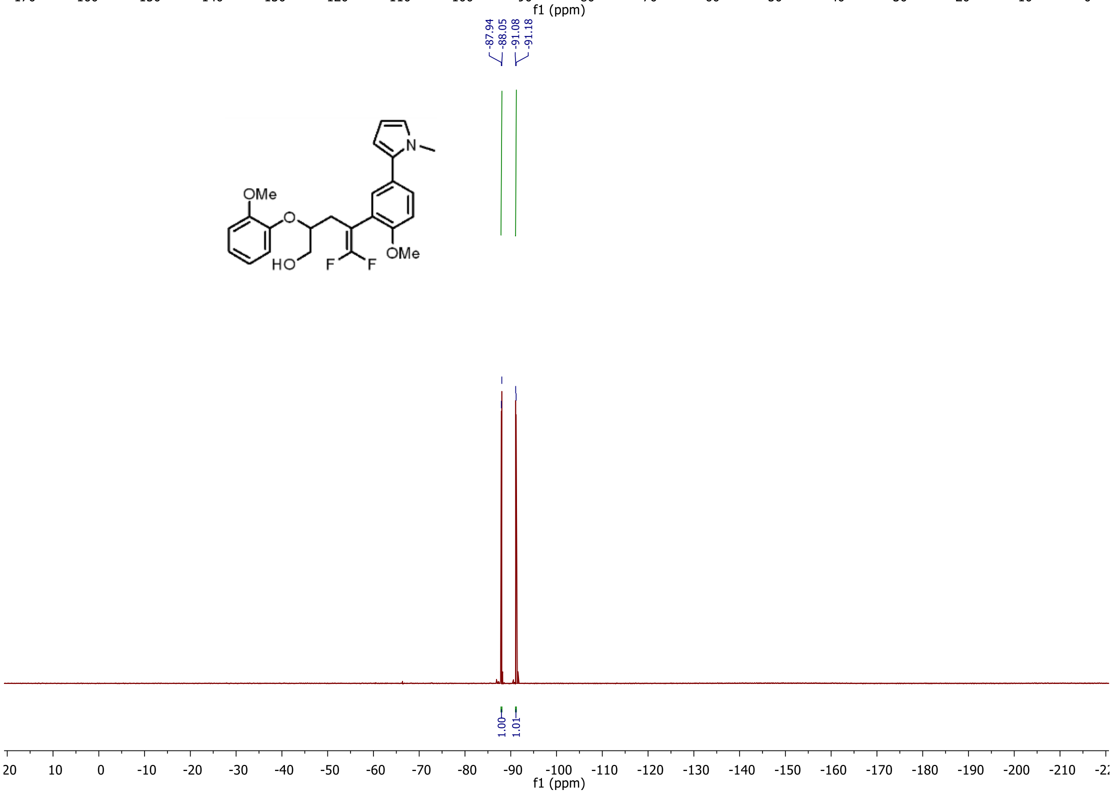

-87.94
-88.05
-91.08
-91.18
OMe
O
N
HO
F
F
OMe
1.00
1.01
20
10
0
-10
-20
-30
-40
-50
-60
-70
-80
-90
-100
-110
-120
-130
-140
-150
-160
-170
-180
-190
-200
-210
f1 (ppm)

REFERENCES AND NOTES

1. J. Zakzeski, P. C. Bruijnincx, A. L. Jongerius, B. M. Weckhuysen, The catalytic valorization of lignin for the production of renewable chemicals. *Chem. Rev.* **110**, 3552–3599 (2010).

2. C. Xu, R. A. Arancon, J. Labidi, R. Luque, Lignin depolymerisation strategies: Towards valuable chemicals and fuels. *Chem. Soc. Rev.* **43**, 7485–7500 (2014).

3. C. Li, X. Zhao, A. Wang, G. W. Huber, T. Zhang, Catalytic transformation of lignin for the production of chemicals and fuels. *Chem. Rev.* **115**, 11559–11624 (2015).

4. Z. Sun, B. Fridrich, A. de Santi, S. Elangovan, K. Barta, Bright side of lignin depolymerization: Toward new platform chemicals. *Chem. Rev.* **118**, 614–678 (2018).

5. M. D. Karkas, B. S. Matsuura, T. M. Monos, G. Magallanes, C. R. Stephenson, Transition-metal catalyzed valorization of lignin: The key to a sustainable carbon-neutral future. *Org. Biomol. Chem.* **14**, 1853–1914 (2016).

6. P. J. Deuss, K. Barta, From models to lignin: Transition metal catalysis for selective bond cleavage reactions. *Coord. Chem. Rev.* **306**, 510–532 (2016).

7. C. Zhang, X. Shen, Y. Jin, J. Cheng, C. Cai, F. Wang, Catalytic strategies and mechanism analysis orbiting the center of critical intermediates in lignin depolymerization. *Chem. Rev.* **123**, 4510–4601 (2023).

8. R. Rinaldi, R. Jastrzebski, M. T. Clough, J. Ralph, M. Kennema, P. C. Bruijnincx, B. M. Weckhuysen, Paving the way for lignin valorisation: Recent advances in bioengineering, biorefining and catalysis. *Angew. Chem. Int. Ed. Engl.* **55**, 8164–8215 (2016).

9. W. Schutyser, T. Renders, S. Van den Bosch, S. F. Koelewijn, G. T. Beckham, B. F. Sels, Chemicals from lignin: An interplay of lignocellulose fractionation, depolymerisation, and upgrading. *Chem. Soc. Rev.* **47**, 852–908 (2018).

10. X. Liu, F. P. Bouxin, J. Fan, V. L. Budarin, C. Hu, J. H. Clark, Recent advances in the catalytic depolymerization of lignin towards phenolic chemicals: A review. *ChemSusChem* **13**, 4296–4317 (2020).

11. A. J. Ragauskas, G. T. Beckham, M. J. Biddy, R. Chandra, F. Chen, M. F. Davis, B. H. Davison, R. A. Dixon, P. Gilna, M. Keller, P. Langan, A. K. Naskar, J. N. Saddler, T. J. Tschaplinski, G. A. Tuskan, C. E. Wyman, Lignin valorization: Improving lignin processing in the biorefinery. *Science* **344**, 1246843 (2014).

12. J. Ralph, K. Lundquist, G. Brunow, F. Lu, H. Kim, P. F. Schatz, J. M. Marita, R. D. Hatfield, S. A. Ralph, J. H. Christensen, W. Boerjan, Lignins: Natural polymers from oxidative coupling of 4-hydroxyphenyl- propanoids. *Phytochem. Rev.* **3**, 29–60 (2004).

13. A. Rahimi, A. Ulbrich, J. J. Coon, S. S. Stahl, Formic-acid-induced depolymerization of oxidized lignin to aromatics. *Nature* **515**, 249–252 (2014).

14. M. Liu, P. J. Dyson, Direct conversion of lignin to functionalized diaryl ethers via oxidative cross-coupling. *Nat. Commun.* **14**, 2830 (2023).

15. M. Rafiee, M. Alherech, S. D. Karlen, S. S. Stahl, Electrochemical aminoxyl-mediated oxidation of primary alcohols in lignin to carboxylic acids: Polymer modification and depolymerization. *J. Am. Chem. Soc.* **141**, 15266–15276 (2019).

16. M. Wang, L. H. Li, J. M. Lu, H. J. Li, X. C. Zhang, H. F. Liu, N. C. Luo, F. Wang, Acid promoted C–C bond oxidative cleavage of β-O-4 and β-1 lignin models to esters over a copper catalyst. *Green Chem.* **19**, 702–706 (2017).

17. S. Gazi, W. K. Hung Ng, R. Ganguly, A. M. Putra Moeljadi, H. Hirao, H. S. Soo, Selective photocatalytic C–C bond cleavage under ambient conditions with earth abundant vanadium complexes. *Chem. Sci.* **6**, 7130–7142 (2015).

18. R. Behling, S. Valange, G. Chatel, Heterogeneous catalytic oxidation for lignin valorization into valuable chemicals: What results? What limitations? What trends? *Green Chem.* **18**, 1839–1854 (2016).

19. C. Yang, S. Maldonado, C. R. J. Stephenson, Electrocatalytic lignin oxidation. *ACS Catal.* **11**, 10104–10114 (2021).

20. A. G. Sergeev, J. F. Hartwig, Selective, nickel-catalyzed hydrogenolysis of aryl ethers. *Science* **332**, 439–443 (2011).

21. M. V. Galkin, J. S. Samec, Lignin valorization through catalytic lignocellulose fractionation: A fundamental platform for the future biorefinery. *ChemSusChem* **9**, 1544–1558 (2016).

22. X. Shen, C. Zhang, B. Han, F. Wang, Catalytic self-transfer hydrogenolysis of lignin with endogenous hydrogen: Road to the carbon-neutral future. *Chem. Soc. Rev.* **51**, 1608–1628 (2022).

23. L. Shuai, M. T. Amiri, Y. M. Questell-Santiago, F. Heroguel, Y. Li, H. Kim, R. Meilan, C. Chapple, J. Ralph, J. S. Luterbacher, Formaldehyde stabilization facilitates lignin monomer production during biomass depolymerization. *Science* **354**, 329–333 (2016).

24. Z. Huang, Z. Yu, Z. Guo, P. Shi, J. Hu, H. Deng, Z. Huang, Selective cleavage of C_{β}–O–4 bond for lignin depolymerization via paired-electrolysis in an undivided cell. *Angew. Chem. Int. Ed. Engl.* **e202407750** (2024).

25. S. Son, F. D. Toste, Non-oxidative vanadium-catalyzed C—O bond cleavage: Application to degradation of lignin model compounds. *Angew. Chem. Int. Ed. Engl.* **49**, 3791–3794 (2010).

26. J. M. Nichols, L. M. Bishop, R. G. Bergman, J. A. Ellman, Catalytic C–O bond cleavage of 2-aryloxy-1-arylethanols and its application to the depolymerization of lignin-related polymers. *J. Am. Chem. Soc.* **132**, 12554–12555 (2010).

27. C. S. Lancefield, O. S. Ojo, F. Tran, N. J. Westwood, Isolation of functionalized phenolic monomers through selective oxidation and C—O bond cleavage of the β-O-4 linkages in lignin. *Angew. Chem. Int. Ed. Engl.* **54**, 258–262 (2015).

28. T. vom Stein, T. den Hartog, J. Buendia, S. Stoychev, J. Mottweiler, C. Bolm, J. Klankermayer, W. Leitner, Ruthenium-catalyzed C—C bond cleavage in lignin model substrates. *Angew. Chem. Int. Ed. Engl.* **54**, 5859–5863 (2015).

29. M. V. Galkin, S. Sawadjoon, V. Rohde, M. Dawange, J. S. M. Samec, Mild heterogeneous palladium-catalyzed cleavage of β-*O*-4′-ether linkages of lignin model compounds and native lignin in air. *ChemCatChem* **6**, 179–184 (2013).

30. X. Wu, X. Fan, S. Xie, J. Lin, J. Cheng, Q. Zhang, L. Chen, Y. Wang, Solar energy-driven lignin-first approach to full utilization of lignocellulosic biomass under mild conditions. *Nat. Catal.* **1**, 772–780 (2018).

31. C. Bertin, C. Cruché, F. Chacón-Huete, P. Forgione, S. K. Collins, Decomposition of lignin models enabled by copper-based photocatalysis under biphasic conditions. *Green Chem.,* **24**, 4414–4419 (2022).

32. J. D. Nguyen, B. S. Matsuura, C. R. Stephenson, A photochemical strategy for lignin degradation at room temperature. *J. Am. Chem. Soc.* **136**, 1218–1221 (2014).

33. I. Bosque, G. Magallanes, M. Rigoulet, M. D. Karkas, C. R. J. Stephenson, Redox catalysis facilitates lignin depolymerization. *ACS Cent. Sci.* **3**, 621–628 (2017).

34. G. Magallanes, M. D. Kärkäs, I. Bosque, S. Lee, S. Maldonado, C. R. J. Stephenson, Selective C–O bond cleavage of lignin systems and polymers enabled by sequential palladium-catalyzed aerobic oxidation and visible-light photoredox catalysis. *ACS Catal.* **9**, 2252–2260 (2019).

35. Q. Zhu, D. G. Nocera, Catalytic C(β)–O bond cleavage of lignin in a one-step reaction enabled by a spin-center shift. *ACS Catal.* **11**, 14181–14187 (2021).

36. S. T. Nguyen, P. R. D. Murray, R. R. Knowles, Light-driven depolymerization of native lignin enabled by proton-coupled electron transfer. *ACS Catal.* **10**, 800–805 (2019).

37. Y. Wang, Y. Liu, J. He, Y. Zhang, Redox-neutral photocatalytic strategy for selective C–C bond cleavage of lignin and lignin models via PCET process. *Sci. Bull.* **64**, 1658–1666 (2019).

38. Y. Wang, J. He, Y. Zhang, $CeCl_3$-promoted simultaneous photocatalytic cleavage and amination of Cα–Cβ bond in lignin model compounds and native lignin. *CCS Chem.* **2**, 107–117 (2020).

39. Y. Li, J. Wen, S. Wu, S. Luo, C. Ma, S. Li, Z. Chen, S. Liu, B. Tian, Photocatalytic conversion of lignin models into functionalized aromatic molecules initiated by the proton-coupled electron transfer process. *Org. Lett.* **26**, 1218–1223 (2024).

40. Y. Patehebieke, R. Charaf, H. P. Bryce-Rogers, K. Ye, M. Ahlquist, L. Hammarström, C.-J. Wallentin, β-scission of secondary alcohols via photosensitization: Synthetic utilization and mechanistic insights. *ACS Catal.* **14**, 585–593 (2024).

41. Y. Li, S. Wu, Y. Liu, Z. He, W. Li, S. Li, Z. Chen, S. Liu, B. Tian, Photoinduced lignin C_α−C_β bond cleavage and chemodivergent functionalization via iron catalysis. *ChemSusChem* **18**, e202401087 (2025).

42. D. Aboagye, R. Djellabi, F. Medina, S. Contreras, Radical-mediated photocatalysis for lignocellulosic biomass conversion into value-added chemicals and hydrogen: Facts, opportunities and challenges. *Angew. Chem. Int. Ed. Engl.* **62**, e202301909 (2023).

43. Z. Huang, N. Luo, C. Zhang, F. Wang, Radical generation and fate control for photocatalytic biomass conversion. *Nat. Rev. Chem.* **6**, 197–214 (2022).

44. Q. Zhang, N. K. Gupta, M. Rose, X. Gu, P. W. Menezes, Z. Chen, Mechanistic insights into the photocatalytic valorization of lignin models via C−O/C−C cleavage or C−C/C−N coupling. *Chem. Catal.* **3**, 100470 (2023).

45. N. A. Meanwell, Synopsis of some recent tactical application of bioisosteres in drug design. *J. Med. Chem.* **54**, 2529–2591 (2011).

46. G. Magueur, B. Crousse, M. Ourévitch, D. Bonnet-Delpon, J.-P. Bégué, Fluoro-artemisinins: When a *gem*-difluoroethylene replaces a carbonyl group. *J. Fluor. Chem.* **127**, 637–642 (2006).

47. C. Leriche, X. He, C.-w. T. Chang, H. w. Liu, Reversal of the apparent regiospecificity of NAD(P)H-dependent hydride transfer: The properties of the difluoromethylene group, a carbonyl mimic. *J. Am. Chem. Soc.* **125**, 6348–6349 (2003).

48. F. Tian, G. Yan, J. Yu, Recent advances in the synthesis and applications of α-(trifluoromethyl)styrenes in organic synthesis. *Chem. Commun.* **55**, 13486–13505 (2019).

49. S. B. Lang, R. J. Wiles, C. B. Kelly, G. A. Molander, Photoredox generation of carbon-centered radicals enables the construction of 1,1-difluoroalkene carbonyl mimics. *Angew. Chem. Int. Ed. Engl.* **56**, 15073–15077 (2017).

50. J. P. Phelan, S. B. Lang, J. Sim, S. Berritt, A. J. Peat, K. Billings, L. Fan, G. A. Molander, Open-air alkylation reactions in photoredox-catalyzed DNA-encoded library synthesis. *J. Am. Chem. Soc.* **141**, 3723–3732 (2019).

51. W. J. Yue, C. S. Day, R. Martin, Site-selective defluorinative sp^3 C–H alkylation of secondary amides. *J. Am. Chem. Soc.* **143**, 6395–6400 (2021).

52. Y. Zhang, Y. Zhang, Y. Guo, S. Liu, X. Shen, Reductive quenching-initiated catalyst-controlled divergent alkylation of α-CF_3-olefins. *Chem Catal.* **2**, 1380–1393 (2022).

53. W. Xu, H. Jiang, J. Leng, H. W. Ong, J. Wu, Visible-light-induced selective defluoroborylation of polyfluoroarenes, *gem*-difluoroalkenes, and trifluoromethylalkenes. *Angew. Chem. Int. Ed. Engl.* **59**, 4009–4016 (2020).

54. Y. Liu, X. Tao, Y. Mao, X. Yuan, J. Qiu, L. Kong, S. Ni, K. Guo, Y. Wang, Y. Pan, Electrochemical C–N bond activation for deaminative reductive coupling of Katritzky salts. *Nat. Commun.* **12**, 6745 (2021).

55. F. Li, C. Pei, R. M. Koenigs, Photocatalytic gem-difluoroolefination reactions by a formal C–C coupling/defluorination reaction with diazoacetates. *Angew. Chem. Int. Ed. Engl.* **61**, e202111892 (2022).

56. Y. Zhao, C. Empel, W. Liang, R. M. Koenigs, F. W. Patureau, Gem-difluoroallylation of aryl sulfonium salts. *Org. Lett.* **24**, 8753–8758 (2022).

57. Y. Q. Guo, R. Wang, H. Song, Y. Liu, Q. Wang, Visible-light-induced deoxygenation/defluorination protocol for synthesis of γ,γ-difluoroallylic ketones. *Org. Lett.* **22**, 709–713 (2020).

58. Y. He, D. Anand, Z. Sun, L. Zhou, Visible-light-promoted redox neutral γ,γ-difluoroallylation of cycloketone oxime ethers with trifluoromethyl alkenes via C–C and C–F bond cleavage. *Org. Lett.* **21**, 3769–3773 (2019).

59. Y. Lan, F. Yang, C. Wang, Synthesis of *gem*-difluoroalkenes via nickel-catalyzed allylic defluorinative reductive cross-coupling. *ACS Catal.* **8**, 9245–9251 (2018).

60. Z. Lin, Y. Lan, C. Wang, Synthesis of *gem*-difluoroalkenes via nickel-catalyzed reductive C–F and C–O bond cleavage. *ACS Catal.* **9**, 775–780 (2019).

61. C. Zhang, Z. Lin, Y. Zhu, C. Wang, Chromium-catalyzed allylic defluorinative ketyl olefin coupling. *J. Am. Chem. Soc.* **143**, 11602–11610 (2021).

62. X. Lu, X. X. Wang, T. J. Gong, J. J. Pi, S. J. He, Y. Fu, Nickel-catalyzed allylic defluorinative alkylation of trifluoromethyl alkenes with reductive decarboxylation of redox-active esters. *Chem. Sci.* **10**, 809–814 (2019).

63. C. Zhu, Z. Y. Liu, L. Tang, H. Zhang, Y. F. Zhang, P. J. Walsh, C. Feng, Migratory functionalization of unactivated alkyl bromides for construction of all-carbon quaternary centers via transposed *tert*-C-radicals. *Nat. Commun.* **11**, 4860 (2020).

64. Z. Li, K. F. Wang, X. Zhao, H. Ti, X. G. Liu, H. Wang, Manganese-mediated reductive functionalization of activated aliphatic acids and primary amines. *Nat. Commun.* **11**, 5036 (2020).

65. C. Yao, S. Wang, J. Norton, M. Hammond, Catalyzing the hydrodefluorination of CF_3-substituted alkenes by $PhSiH_3$. H• transfer from a nickel hydride. *J. Am. Chem. Soc.* **142**, 4793–4799 (2020).

66. F. Chen, X. Xu, Y. He, G. Huang, S. Zhu, NiH-catalyzed migratory defluorinative olefin cross-coupling: Trifluoromethyl-substituted alkenes as acceptor olefins to form *gem*-difluoroalkenes. *Angew. Chem. Int. Ed. Engl.* **59**, 5398–5402 (2020).

67. K. Wang, W. Kong, Synthesis of fluorinated compounds by nickel-catalyzed defluorinative cross-coupling reactions. *ACS Catal.* **13**, 12238–12268 (2023).

68. J. J. Warren, T. A. Tronic, J. M. Mayer, Thermochemistry of proton-coupled electron transfer reagents and its implications. *Chem. Rev.* **110**, 6961–7001 (2010).

69. G. Chelucci, Synthesis and metal-catalyzed reactions of *gem*-dihalovinyl systems. *Chem. Rev.* **112**, 1344–1462 (2012).

70. X. Zhang, S. Cao, Recent advances in the synthesis and C–F functionalization of *gem*-difluoroalkenes. *Tetrahedron Lett.* **58**, 375–392 (2017).

71. S. Couve-Bonnaire, D. Cahard, X. Pannecoucke, Chiral dipeptide mimics possessing a fluoroolefin moiety: A relevant tool for conformational and medicinal studies. *Org. Biomol. Chem.* **5**, 1151–1157 (2007).

72. W. Zhang, W. Huang, J. Hu, Highly stereoselective synthesis of monofluoroalkenes from α-fluorosulfoximines and nitrones. *Angew. Chem. Int. Ed. Engl.* **48**, 9858–9861 (2009).

73. X. Hu, G. Zhang, F. Bu, A. Lei, Selective oxidative [4+2] imine/Alkene annulation with H_2 liberation induced by photo-oxidation. *Angew. Chem. Int. Ed. Engl.* **57**, 1286–1290 (2018).

74. R. D. Van Rijn, H. J. J. Loozen, C. M. Timmers, L. A. Van Der Veen, W. F. J. Karstens (N.V. Organon), (dihydro)pyrrolo[2,1-a]isoquinolines, WO2009098283 (2009).

75. S. Alunni, F. De Angelis, L. Ottavi, M. Papavasileiou, F. Tarantelli, Evidence of a borderline region between E1cb and E2 elimination reaction mechanisms: A combined experimental and theoretical study of systems activated by the pyridine ring. *J. Am. Chem. Soc.* **127**, 15151–15160 (2005).

76. T. M. Monos, R. C. McAtee, C. R. J. Stephenson, Arylsulfonylacetamides asbifunctional reagents foralkene aminoarylation, *Science* **361**, 1369–1373 (2018).

77. J. Xiao, J. Montgomery, Nickel-catalyzed defluorinative coupling of aliphatic aldehydes with trifluoromethyl alkenes. *ACS Catal.* **12**, 2463–2471 (2022).

78. M. Hu, B. B. Tan, S. Ge, Enantioselective cobalt-catalyzed hydroboration of fluoroalkyl-substituted alkenes to access chiral fluoroalkylboronates. *J. Am. Chem. Soc.* **144**, 15333–15338 (2022).

79. J. P. Phelan, S. B. Lang, J. S. Compton, C. B. Kelly, R. Dykstra, O. Gutierrez, G. A. Molander, Redox-neutral photocatalytic cyclopropanation via radical/polar crossover. *J. Am. Chem. Soc.* **140**, 8037–8047 (2018).

80. X.-L. Chen, D.-S. Yang, B.-C. Tang, C.-Y. Wu, H.-Y. Wang, J.-T. Ma, S.-Y. Zhuang, Z.-C. Yu, Y.-D. Wu, A.-X. Wu, Direct hydrodefluorination of CF_3-alkenes via a Mild S_N2' process using rongalite as a masked proton reagent. *Org. Lett.* **25**, 2294–2299 (2023).

81. Y.-Q. Guo, Y. Wu, R. Wang, H. Song, Y. Liu, Q. Wang, Photoredox/hydrogen atom transfer cocatalyzed C–H difluoroallylation of amides, ethers, and alkyl aldehydes. *Org. Lett.* **23**, 2353–2358 (2021).

82. K. Fuchibe, H. Jyono, M. Fujiwara, T. Kudo, M. Yokota, I. Ichikawa, Domino Friedel–Crafts-type cyclizations of difluoroalkenes promoted by the α-cation-stabilizing effect of fluorine: An efficient method for synthesizing angular PAHs. *Chem. A Eur. J.* **17**, 12175–12185 (2011).

83. C. M. Zhu, R.-B. Liang, Y. Xiao, W. Zhou, Q.-X. Tong, J.-J. Zhong, Metal-free and site-selective α-C–H functionalization of tetrahydrofuran enabled by the photocatalytic generation of bromine radicals. *Green Chem.* **25**, 960–965 (2023).

84. C. Sun, L. Zheng, W. Xu, A. V. Dushkin, W. Su, Mechanochemical cleavage of lignin models and lignin via oxidation and a subsequent base-catalyzed strategy. *Green Chem.* **22**, 3489–3494 (2020).

85. J. Buendia, J. Mottweiler, C. Bolm, Preparation of diastereomerically pure dilignol model compounds. *Chem. A Eur. J.* **17**, 13877–13882 (2011).

86. J. Bomon, E. Van Den Broeck, M. Bal, Y. Liao, S. Sergeyev, V. Van Speybroeck, B. F. Sels, B. U. W. Maes, Brønsted acid catalyzed tandem defunctionalization of biorenewable ferulic acid and derivates into bio-catechol. *Angew. Chem. Int. Ed. Engl.* **59**, 3063–3068 (2020).

87. E. Baciocchi, C. Fabbri, O. Lanzalunga, Lignin peroxidase-catalyzed oxidation of nonphenolic trimeric lignin model compounds: Fragmentation reactions in the intermediate radical cations. *J. Org. Chem.* **68**, 9061–9069 (2003).

88. D. W. Cho, R. Parthasarathi, A. S. Pimentel, G. D. Maestas, H. J. Park, U. C. Yoon, D. Dunaway-Mariano, S. Gnanakaran, P. Langan, P. S. Mariano, Nature and kinetic analysis of carbon−carbon bond fragmentation reactions of cation radicals derived from SET-oxidation of lignin model compounds. *J. Org. Chem.* **75**, 6549–6562 (2010).

89. S. H. Lim, W. S. Lee, Y.-I. Kim, Y. Sohn, D. W. Cho, C. Kim, E. Kim, J. A. Latham, D. Dunaway-Mariano, P. S. Mariano, Photochemical and enzymatic SET promoted C–C bond cleavage reactions of lignin β-1 model compounds containing varying number of methoxy substituents on their arene rings. *Tetrahedron* **71**, 4236–4247 (2015).

90. E. M. Espinoza, J. A. Clark, J. Soliman, J. B. Derr, M. Morales, V. I. Vullev, Practical aspects of cyclic voltammetry: How to estimate reduction potentials when irreversibility prevails. *J. Electrochem. Soc.* **166**, H3175–H3187 (2019).
